# Genital tract infections, the vaginal microbiome and gestational age at birth among pregnant women in South Africa: a cohort study protocol

**DOI:** 10.1136/bmjopen-2023-081562

**Published:** 2023-12-28

**Authors:** Ranjana M S Gigi, Mandisa M Mdingi, Hyunsul Jung, Shantelle Claassen-Weitz, Lukas Bütikofer, Jeffrey D Klausner, Christina A Muzny, Christopher M Taylor, Janneke H H M van de Wijgert, Remco P H Peters, Nicola Low

**Affiliations:** 1 Institute of Social and Preventive Medicine, University of Bern, Bern, Switzerland; 2 Research Unit, Foundation for Professional Development, East London, South Africa; 3 Department of Medical Microbiology, University of Pretoria, Pretoria, South Africa; 4 Department of Pathology, University of Cape Town, Cape Town, South Africa; 5 CTU Bern, Department of Clinical Research, University of Bern, Bern, Switzerland; 6 Department of Population and Public Health Sciences, Keck School of Medicine, University of Southern California, Los Angeles, California, USA; 7 Division of Infectious Diseases, University of Alabama at Birmingham, Birmingham, Alabama, USA; 8 Department of Microbiology, Immunology, and Parasitology, Louisiana State University Health Sciences Center, New Orleans, Louisiana, USA; 9 Julius Center for Health Sciences and Primary Care, University Medical Center Utrecht, Utrecht University, Utrecht, Netherlands

**Keywords:** epidemiologic studies, sexually transmitted disease, diagnostic microbiology, maternal medicine, follow-up studies

## Abstract

**Introduction:**

Preterm birth complications are the most common cause of death in children under 5 years. The presence of multiple microorganisms and genital tract inflammation could be the common mechanism driving early onset of labour. South Africa has high levels of preterm birth, genital tract infections and HIV infection among pregnant women. We plan to investigate associations between the presence of multiple lower genital tract microorganisms in pregnancy and gestational age at birth.

**Methods and analysis:**

This cohort study enrols around 600 pregnant women at one public healthcare facility in East London, South Africa. Eligible women are ≥18 years and at <27 weeks of gestation, confirmed by ultrasound. At enrolment and 30–34 weeks of pregnancy, participants receive on-site tests for *Chlamydia trachomatis* and *Neisseria gonorrhoeae*, with treatment if test results are positive. At these visits, additional vaginal specimens are taken for: PCR detection and quantification of *Trichomonas vaginalis*, *Candida* spp., *Mycoplasma genitalium, M. hominis*, *Ureaplasma urealyticum* and *U. parvum*; microscopy and Nugent scoring; and for 16S ribosomal RNA gene sequencing and quantification. Pregnancy outcomes are collected from a postnatal visit and birth registers. The primary outcome is gestational age at birth. Statistical analyses will explore associations between specific microorganisms and gestational age at birth. To explore the association with the quantity of microorganisms, we will construct an index of microorganism load and use mixed-effects regression models and classification and regression tree analysis to examine which combinations of microorganisms contribute to earlier gestational age at birth.

**Ethics and dissemination:**

This protocol has approvals from the University of Cape Town Research Ethics Committee and the Canton of Bern Ethics Committee. Results from this study will be uploaded to preprint servers, submitted to open access peer-reviewed journals and presented at regional and international conferences.

**Trial registration number:**

NCT06131749; Pre-results.

**STRENGTHS AND LIMITATIONS OF THIS STUDY**

- This cohort study takes a holistic approach, investigating both the presence and quantity of multiple lower genital tract microorganisms, including vaginal microbiota, in pregnancy and their associations with gestational age at birth.
- The study is set in a location where the prevalence of genital tract infections and adverse pregnancy outcomes is high, uses ultrasound scans to assess gestational age at enrolment accurately, and uses state-of-the-art molecular diagnostic methods.
- The study setting is limited to one research site, which may affect the generalisability of the findings.
- The use of gestational age at birth as a continuous outcome, instead of preterm birth as a dichotomous outcome, might limit comparability with other studies, but we will also examine the binary outcome preterm birth in secondary analyses.

## Introduction

Preterm birth complications are the most common cause of death in children under 5 years.^1^ Close to 1 million infants die every year because they are born preterm (before 37 completed weeks of gestation), mainly from infectious, respiratory and neurological complications, and those that survive can experience long-term morbidity.^1 2^ South Africa has a high incidence of preterm birth at around 10%,^3^ around 30% of women have one or more curable sexually transmitted infections during pregnancy^4 5^ and about 30% of pregnant women are living with HIV.^6^

Microbial colonisation or infection during pregnancy, in the lower or upper genital tract, has been reported to predispose to preterm birth, as do anatomical, biochemical, endocrinological, immunological, nutritional, environmental and psychosocial factors.^7 8^ The presence of microorganisms may contribute to early onset of labour directly, through presumed ascension from the lower to the upper genital tract, or indirectly, through a pathway of inflammatory response, or a combination of both.^7 9^ Inflammation may be the common pathway, even if infection has not reached the amniotic cavity.^10^

Much of the research reporting on the role of sexually transmitted infections in pregnancy and preterm birth has focused on single infections, such as *Chlamydia trachomatis,*^11^ *Neisseria gonorrhoeae*^12^ and *Trichomonas vaginalis*.^13^ *Mycoplasma genitalium* is the most recently recognised bacterial sexually transmitted infection and, while an association with preterm birth has been reported, there are few studies with prospective data collection.^14^ Bacterial vaginosis is the most common vaginal microbiota dysbiosis and is associated with adverse pregnancy outcomes, either alone or in combination with other sexually transmitted infections.^15–17^ Associations with adverse birth outcomes have also been observed for other genital mycoplasmas, *M. hominis, Ureaplasma urealyticum* and *U. parvum*.^18^ For individual sexually transmitted infections, bacterial vaginosis and colonisation by other genital mycoplasmas, summary ORs for the association with adverse birth outcomes in meta-analyses of univariable data are generally around 1.3–2.0.^11–14 16 18^ *Candida* spp have not been found to be associated with preterm birth, but an association with more inflammatory, symptomatic yeast infection cannot be ruled out.^19^ Most studies about these microorganisms do not present analyses that examine the role of co-occurrence or control for confounding factors, so the presence or strength of the causal association cannot be assessed.^20^ It is also important to include women living with HIV, among whom there are fewer studies about associations between genital tract infections and adverse birth outcomes than among women without HIV infection.^21 22^

The importance of the quantity of different microorganisms as a driver of preterm birth has not been extensively studied,^23–25^ but might be as, or more, relevant than their presence.^23^ Together with inflammation or immune activation in the genital tract during pregnancy, organism load could be an important driver of the early onset of labour and preterm birth.^8 23 24 26 27^ This calls for a holistic approach to research studies, which combines information about the presence of different microorganisms, the quantified load and the microbiome, sociodemographic factors and HIV among women living with infection, most of whom are receiving antiretroviral therapy. The overall aim of this study is to investigate associations between the presence of lower genital tract microorganisms in pregnancy and preterm birth and other adverse pregnancy outcomes. This will be achieved through three objectives to explore: (1) the association between the presence of specific lower genital tract microorganisms and gestational age at birth (primary outcome), as well as secondary adverse pregnancy outcomes; (2) the association between quantified load of vaginal and sexually transmitted microorganisms and gestational age at birth (primary outcome) as well as secondary adverse pregnancy outcomes; and (3) the combinations of microorganisms that are most strongly associated with earlier gestational age at birth.

## Methods and analysis

### Study design and setting

**Figure 1:**
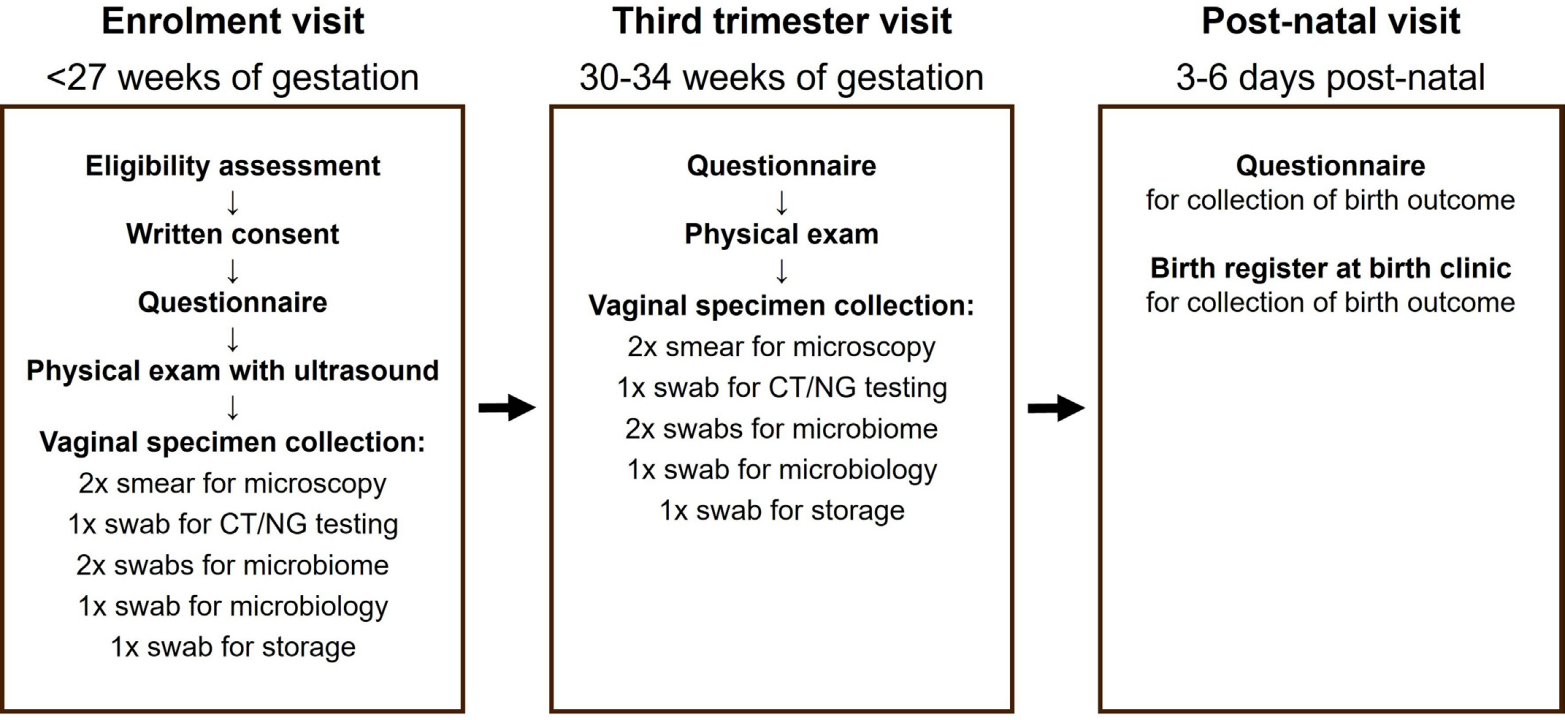
Study visits and specimen collection. CT, *Chlamydia trachomatis*; NG, *Neisseria gonorrhoeae*.

This prospective closed cohort study follows women enrolled during pregnancy until after they give birth (figure 1). The study is conducted at the antenatal clinic of one primary healthcare facility in Buffalo City Metropolitan Municipality, Eastern Cape Province, South Africa. This cohort study is part of a larger project, called Philani Ndiphile (meaning ‘be healthy and I will be healthy’ in isiXhosa), which includes a randomised implementation-effectiveness trial of screening strategies for sexually transmitted infections in pregnancy^28^ and a case–control study about persistent *C. trachomatis* infection.

### Participants

Inclusion criteria: Pregnant women aged 18 years or older, who live in Buffalo City Metropolitan Municipality, intend to deliver in the same municipality and provide written informed consent to take part in the study. The eligible gestational age at enrolment, confirmed by ultrasound, was below 20 weeks at the start of the study in March 2021 and was increased to 27 weeks in September 2021 to increase enrolment and to align with another trial.^29^

Exclusion criteria: Participation in any other research study or inability to understand and speak a local language (English, Afrikaans or isiXhosa).

### Enrolment

A trained study field worker approaches all pregnant women attending an antenatal care visit at the clinic and individually informs them about the study. If a potential participant shows interest in the study, the study field worker checks for eligibility. The date of the last menstrual period is used initially to estimate gestational age. If all eligibility criteria are met, a study field worker obtains written informed consent from the participant.

### Study procedures and visits

At the enrolment visit, study field workers administer a questionnaire to record sociodemographic, behavioural and clinical information in an online Research Electronic Data Capture (REDCap)^30^ software database. The study nurse examines the woman, according to the South African government standard of care.^31^ As an additional procedure, a study nurse with training in obstetric ultrasound performs an abdominal ultrasound to estimate the gestational age. If this is later than the eligibility criterion, the participant is excluded from any further study activity. A study nurse collects vaginal samples (figure 1) for on-site testing for *C. trachomatis* and *N. gonorrhoeae* using the Xpert CT/NG assay on the Gene Xpert platform (Cepheid, Sunnyvale, California, USA) and for further off-site laboratory testing (see ‘Specimen collection and analysis’).

If the test result for *C. trachomatis* or *N. gonorrhoeae* is positive, the woman receives immediate antibiotic treatment if still on-site or is contacted by telephone and asked to return to the clinic for treatment. Antibiotic treatments are first-line regimens according to South African guidelines: for *C. trachomatis*, 1 g oral azithromycin and for *N. gonorrhoeae*, 500 mg intramuscular ceftriaxone (250 mg until South African treatment guidelines for sexually transmitted infections changed in December 2022).^32^ Women with vaginal discharge syndrome but with negative Xpert test results for *C. trachomatis* and *N. gonorrhoeae* receive empirical treatment for trichomoniasis with metronidazole 400 mg twice a day for 7 days. The study nurse gives advice to women with *C. trachomatis* or *N. gonorrhoeae* on safe disclosure of her diagnosis to her partner(s) and gives her a notification slip(s) to request her partner(s) to attend a clinic for treatment.

A follow-up visit at 30–34 weeks (third trimester visit) is scheduled at which clinical and obstetric information, as well as the same vaginal specimens, is collected and treatment given, if indicated.

A postnatal visit is scheduled for 3–6 days after giving birth, according to the South African government standard.^31^ A study nurse collects information about the birth outcome and perinatal period through a questionnaire with the mother, a patient-held medical record of the baby (the Road to Health card) and/or the birth register from the public birth clinics within the study area. If the participant does not attend the postnatal visit, study staff telephone her to ask her to return to the clinic. If the participant is not able to return to the clinic, the study physician collects the information by telephone or from the birth register.

### Outcomes

The primary outcome is gestational age at birth, measured in days, based on the ultrasound assessment at the enrolment visit. Secondary outcomes are preterm birth (<37 completed weeks of gestation), low birth weight (birth weight <2500 g), miscarriage (dead fetus delivered before 28 completed weeks of pregnancy or with birth weight below 1000 g) and stillbirth (dead fetus delivered at or after 28 completed weeks of pregnancy or with birth weight above 1000 g).^33 34^ We chose gestational age at birth as the primary outcome because, while the cut-off of 37 weeks is the standard definition of preterm birth, dichotomisation of a continuous variable results in a loss of statistical power.^35^

### Specimen collection and analysis

#### Data sources and variables

The source data are case report forms recording questionnaire data for the enrolment, third trimester and postnatal visits, and forms for laboratory and specimen results, which are stored in REDCap, a secure web-based database^30^ (online supplemental file), hosted by the Foundation for Professional Development, Pretoria, South Africa.

10.1136/bmjopen-2023-081562.supp1 Supplementary data

#### Specimen collection

At the enrolment and the third trimester visits, a study nurse collects two vaginal smears using inoculation loops and air dries them on glass slides. She then collects vaginal specimens by inserting swabs into the vagina up to a mark at 4 cm and rotating around the vaginal wall. Five swabs are collected in the following order: one Cepheid GeneXpert Xpert Vaginal/Endocervical Swab in a tube with Xpert Swab Transport Reagent (Cepheid); two Qiagen digene Female Swabs in a single tube with digene Specimen Transport Medium (Qiagen, Hilden, Germany); and two dry FLOQSwabs (COPAN, Brescia, Italy) each in a separate sterile tube (figure 1).

#### Transport and storage of specimens

Vaginal smear glass slides are stored and transported in plastic slide carriers at room temperature. All vaginal swabs are initially stored at the clinic in a refrigerator (2–8°C with daily temperature checks). All vaginal swabs, except the Xpert swab, which is tested on-site, are transported on ice packs once a week by overnight road courier to the laboratory at the Department of Medical Microbiology, University of Pretoria, where they are also stored in a refrigerator until DNA extraction.

#### Microbiological analyses

The Xpert vaginal swabs are tested on-site using the Xpert CT/NG assay (Cepheid) to detect *C. trachomatis* and *N. gonorrhoeae*, as per manufacturer’s instructions. At the University of Pretoria, air-dried vaginal smears are heat fixed and Gram stained.^36^ Two qualified people record the Nugent scores (0–3: normal; 4–6: intermediate; 7–10: bacterial vaginosis) and the presence of yeasts.^37^ In case of discrepancies, a third person assesses the slide and consensus is reached by discussion. At the University of Pretoria, one vaginal FLOQSwabs is used for PCRs. The genomic DNA is extracted using the High Pure PCR Template Preparation Kit (Roche Diagnostics, Mannheim, Germany) as per manufacturer’s instructions. Real-time PCR assays are then performed using the LightCycler 480 Probes Master Kit (Roche Diagnostics) on the LightCycler 480 II instrument (Roche Diagnostics). Previously published primer and hydrolysis probe sequences and cycling conditions are used for detection and quantification of *M. genitalium*,^38^ *M. hominis*,^39^ *U. parvum*,^40^ *U. urealyticum,*^40^ *T. vaginalis*^41^ and *Candida* spp.^42 43^ The load for each assayed microorganism detected in vaginal swab specimens by real-time PCR or GeneXpert is obtained from the cycle threshold value.

#### Vaginal microbiome laboratory analyses

The vaginal swabs stored in Qiagen digene Specimen Transport Medium will be used for DNA extraction and subsequent 16S ribosomal RNA (rRNA) amplicon sequencing targeting the V3–V4 hypervariable regions for vaginal microbiota analyses at the Division of Medical Microbiology, University of Cape Town.

A commercial DNA extraction kit will be used and a bead-beating step included.^44^ A DNA isolation control will be prepared from an unused vaginal swab specimen during this process. Two PCR rounds will be conducted to prepare amplicon libraries.^45^ The aim of the first PCR round is to amplify 16S rRNA gene V3–V4 regions using the 319F 5′-ACTCCTACGGGAGGCAGCAG-3′ forward primer and 806R 5′-GGACTACHVGGGTWTCTAAT-3′ reverse primer. The aim of the second PCR round is to barcode the V3–V4 amplicons by a dual-index approach, permitting multiplexing of up to 384 samples (including controls). Amplicon concentrations for all sample libraries are measured and normalised to form a mixed loading library. The libraries will be sequenced on an Illumina MiSeq instrument (Illumina, San Diego, California, USA), 2×300 bp. To quantify the number of 16S rDNA copies per swab, a quantitative PCR using the same forward and reverse primers as described above will be used. Samples from enrolment and third trimester visits from the same woman will be processed in the same run.

#### Vaginal microbiota bioinformatics

Raw sequencing reads will be processed using an established bioinformatics pipeline.^46^ Taxonomic assignment of amplicon sequence variants will be done in DADA2^47^ with SILVA^48^ as the reference database. Vaginal microbiome composition data will be visualised in heatmaps and diagrams. For each vaginal sample, we will calculate diversity measures (alpha diversity), relative abundances and estimated concentrations of key vaginal bacteria and bacterial groups, as described.^46^ We will use the entire sequencing dataset to design vaginal microbiota types by hierarchical clustering, and each sample will be assigned to one vaginal microbiota type.

### Sample size calculation

**Figure 2:**
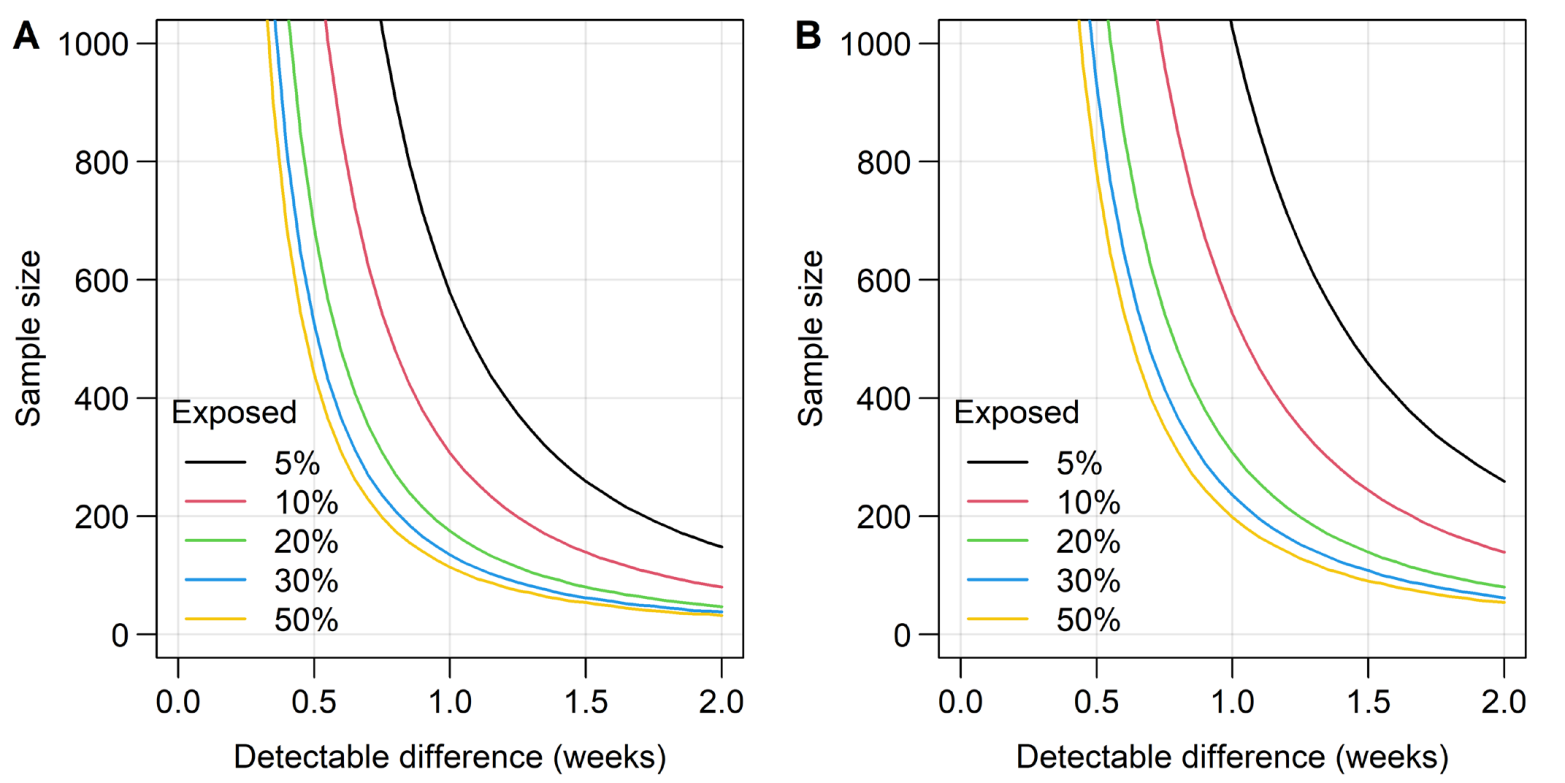
Sample size requirements at different levels of exposure prevalence with power of 80% and alpha 0.83% based on Student’s t-test. (A) SD 1.5. (B) SD 2.0. The curves for % exposed are symmetrical around a prevalence of 50%, that is, curve for 10% exposed is same as that for 90% exposed.

The sample size has been calculated for objective 1, with a univariable comparison between the presence of a genital tract microorganism in the mother and gestational age at birth. Figure 2 shows that, for any vaginal or sexually transmitted microorganism, or vaginal microbiota type that has a prevalence of 10% or more among all enrolled women, about 500–600 patients provide adequate power (80%) to detect a 1-week difference (with SD 2) in mean gestational age between the two groups using Student’s t-test. Specifying an alpha of 0.83% allows for multiple hypothesis testing (six hypotheses, using a Bonferroni correction). We enrol around 600 women and aim to have complete follow-up and outcome data on at least 550 women.

#### Study timeline

Enrolment began on 28 March 2021, with an estimated date for reaching the target sample size in August 2023. Follow-up of all participants until the postnatal visit is expected to be completed by March 2024.

### Statistical analysis

This description gives an overview of the statistical methods for each objective. A detailed statistical analysis plan will be published separately and made publicly available.

We will describe the numbers of women enrolled and available at each follow-up visit in a flow chart. We will present descriptive tables of sociodemographic, behavioural and clinical characteristics and compare women with complete follow-up with those lost to follow-up.

#### Objective 1: association between specified exposures and pregnancy outcomes

1. We will examine a primary set of microorganisms as exposures, detected at either enrolment or at the third trimester visit: *M. genitalium, M. hominis*, *U. urealyticum, U. parvum, T. vaginalis* and *Candida* spp are the microorganisms for which women did not receive diagnostic tests and treatment during study visits. We will use the mean and SD for the continuous outcome (gestational age) and absolute and relative frequencies for the binary outcomes (all secondary outcomes). Gestational age at birth for each exposure will be compared using Student’s t-test and a mean difference with 95% CIs (continuous outcome) and Fisher’s exact test and a risk difference with 95% CIs (binary outcomes).
  To control for confounding, multivariable regression models will be fitted to all outcomes with a set of prespecified potential confounders (age, educational level, alcohol consumption, HIV infection, prior preterm birth) for all organisms. For analyses of *M. genitalium*, *T. vaginalis* and *Candida* spp, we will also control for bacterial vaginosis (Nugent score 7–10). The other genital mycoplasmas can be identified from 16S rRNA amplicon sequencing in women with vaginal dysbiosis, so are sometimes considered part of bacterial vaginosis. For these organisms, we will conduct descriptive analyses, stratified by the presence of bacterial vaginosis. For continuous confounders, a linear relationship will be assumed by default but transformations (eg, log) or more flexible approaches (eg, splines or fractional polynomials) will be considered if there is evidence for non-linearity. For the continuous outcome, we will use linear mixed-effects regression models (including data from either visit and the participant as random effect) and report the result as mean difference with 95% CIs. For the binary outcome, we will use logistic mixed-effects regression and report the result as ORs with 95% CIs.
2. Comparisons for associations with timing of detection, other microorganism exposures and birth outcomes will be considered secondary analyses. Associations between vaginal microbiota composition and pregnancy outcomes will be assessed. We will use compositional multivariable analysis methods to identify bacterial taxa that are differentially abundant between binary pregnancy outcome groups at the level of individual taxon relative abundances. We will use mixed-effects models (with the individual participant as the random effect and including data from both visits) to assess associations between continuous and binary pregnancy outcome and the following fixed effects derived from the vaginal microbiota data: alpha diversity, vaginal microbiota types and absolute abundances of predefined bacterial groups.^46^ These models will be adjusted for confounding as described in the previous paragraph.

#### Objective 2: association between quantified microorganism load and pregnancy outcomes

We will investigate the hypothesis that the quantity of microorganisms with inflammatory potential is associated with gestational age at birth. For this, we will analyse the vaginal microbiota data jointly with sexually transmitted infections and *Candida* spp diagnostic test results during pregnancy (these will be considered as additional covariates in the above-mentioned regression models). We will develop a ‘vaginal inflammation index’ based on quantification of the vaginal microbiota and their inflammatory potential^49^ and of yeasts. This vaginal inflammation index will also be analysed as a fixed effect in mixed-effects models with pregnancy outcomes as the outcomes; these models will not include any of the infection parameters that were used to design the index.

#### Objective 3: classification and regression tree analysis for the primary outcome

We will conduct exploratory analyses to examine the combination of microorganisms that best predicts earlier gestational age at birth using classification and regression tree analysis.^50^ This method belongs to the family of decision tree machine learning algorithms and allows for non-parametric analyses of a large number of binary, categorical or continuous predictors. They are typically easy to interpret and can detect predictors with small marginal effects when there are strong interaction effects. We will make use of the predictive potential for gestational age at birth of all sexually transmitted and genital tract microorganisms, including individual bacterial taxa or bacterial groups identified by 16S rRNA gene amplicon sequencing (as binary or continuous variables) and confounding variables identified in objective 1. We will present variable importance scores and curves of marginal effects to show how prediction of the outcome changes at different levels of the exposure of each variable in the model. To avoid overfitting, we will consider bootstrap aggregating via random forests.^51^

### Data management and confidentiality

#### Data management

Each potential participant screened for eligibility is assigned a unique participant identification number, which does not include any personal identifying information. Personal identification numbers are used to link records, specimens and laboratory test results of the participants. Data are stored in a REDCap database,^30^ which is only accessible to authorised project staff. Paper records are kept in lockable fire-resistant filing cabinets. Laboratory records and journals are kept at the University of Pretoria and University of Cape Town. Forms with personal identifying information are kept separately from demographic, clinical and other data. The data manager maintains a separate, access-controlled database that links the personal identification number with identifying information. Data quality checks are conducted by study staff on-site and data administrators at the office of the Foundation for Professional Development. All study data are stored securely at the offices of the Foundation for Professional Development in East London for up to 5 years after the completion of the study or as required by the institutional review board.

#### Confidentiality

The research team is trained to adhere to guidelines on the Protection of Human Research Participants and Good Clinical Practice and fully protects the confidentiality of participants. Besides the measures described under data management, interviews are conducted in a private setting. In reports and publications, data will not be presented in a way where it could be linked to individual participants.

### Patient and public involvement

There was no involvement of patients or the public in the development of the research questions or the study methods. The research findings will be shared through open access publications and in dissemination meetings with local stakeholders, healthcare providers and communities.

## Discussion

This project is important because of its holistic approach, which considers associations between different genital tract infections, their quantity and the vaginal microbiota on earlier gestational age at birth. Many studies in this field have focused on only one or two microorganisms and few studies involve women in sub-Saharan Africa. Strengths of this study include the study setting, where the prevalence of both genital tract infections and adverse pregnancy outcomes is high, the use of ultrasound scans at enrolment for accurate assessment of gestational age and the use of state-of-the-art molecular diagnostic tests and 16S rRNA sequencing. The residual DNA from samples collected in this study will be available for future studies, including joint analyses with other studies of the influence of vaginal microbiota on adverse pregnancy outcomes.

There are limitations to the study design. First, this study involves participants from one clinic, which might limit the generalisability of the findings. Second, using gestational age at delivery as a continuous outcome instead of preterm birth as a dichotomous outcome might limit comparability with other studies. We will, however, examine the binary outcome preterm birth in secondary analyses. Third, the vaginal samples are taken in a fixed sequence at each visit, which might reduce the microorganism load of later samples. Fourth, the development of the vaginal inflammation index will use information about the inflammatory potential of microorganisms,^49^ rather than direct concentrations of inflammatory markers.

This study has the potential to generate new evidence about the role of different microorganisms in earlier gestational age at birth through analyses of the presence and quantity of individual and combinations of microorganisms, relative abundance of bacterial genera and microbiota on gestational age at birth. This study will generate new hypotheses, which can be investigated in future studies.

## Ethics and dissemination

This protocol and the informed consent forms are approved by the University of Cape Town Research Ethics Committee (reference: 676/2019), which includes activities at the University of Southern California, University of Alabama at Birmingham and Louisiana State University. Authorisation to analyse deidentified data at the University of Bern has been granted by the Canton of Bern Ethics Committee (reference: 2021-01209). Results from this study will be submitted to regional and international conferences and to open access peer-reviewed journals and preprint servers.

## Data statement

The research team will prepare datasets used in analyses, in accordance with data sharing requirements of open access journals in which manuscripts are published and in compliance with local Protection of Personal Information Act requirements. These data files will be archived with codebooks as .csv documents or R datasets and stored in REDCap. The final data files will not contain any personal identifying information of participants.

## Supplementary Material

Reviewer comments

Author's manuscript
