# Supplementary material for: Genital tract infections, the vaginal microbiome and gestational age at birth among pregnant women in South Africa: a cohort study protocol
Source: BMJ Open. 2023 Dec 28;13(12):e081562. doi: 10.1136/bmjopen-2023-081562 (PMC10759125; doi:10.1136/bmjopen-2023-081562)
Supplement: Supplementary data [file bmjopen-2023-081562supp001.pdf]

Supplemental online file – study questionnaire

Genital tract infections, the vaginal microbiome and gestational age at birth among pregnant women in South Africa: a cohort study protocol

Ranjana M S Gigi,[1 2] Mandisa M Mdingi,[2] Hyunsul Jung,[3] Shantelle Claassen Weitz,[4] Lukas Bütikofer,[5] Jeffrey D Klausner,[6] Christina A Muzny,[7] Christopher M Taylor,[8] Janneke H H M van de Wijgert*,[9] Remco P H Peters*,[2 3 4] Nicola Low*[1]

1) Institute of Social and Preventive Medicine, University of Bern, Bern, Switzerland

2) Research Unit, Foundation for Professional Development, East London, South Africa

3) Department of Medical Microbiology, University of Pretoria, Pretoria, South Africa

4) Department of Pathology, University of Cape Town, Cape Town, South Africa

5) CTU Bern, Department of Clinical Research, University of Bern, Bern, Switzerland

6) Keck School of Medicine, University of Southern California, Los Angeles, USA

7) Division of Infectious Diseases, University of Alabama at Birmingham, Birmingham, USA

8) School of Medicine, Louisiana State University, USA

9) Julius Center for Health Sciences and Primary Care, University Medical Center Utrecht, Utrecht University, Utrecht, Netherlands

Screening and Enrolment

Record ID

____

Site Information

Today's Date

____

Study Staff Name

____

Start Time

____

Please select Study Site Name

◯ Grey Gateway
◯ Duncan Village CHC
◯ Nontyatyambo CHC
◯ Gompo
◯ Ndevana

Introduction to the Study:

Note to RA:

In this section you will be introducing the study to the participant. Please make sure to execute the following steps:

1. Introduce the study

◯ Proceed

Does the participant show interest in the study

◯ Yes the participant shows interest
◯ No the participant is not interested in the study

END

The participant is not interested in the study. Thank them for their time.

Eligibility Screening

Note to RA:
The participant seems to show interest in the study. We need to determine their eligibility status. You will ask a series of questions to determine this. Please select "Proceed" to continue.

◯ Proceed

Is the participant currently living in BCM?

◯ Yes
◯ No

Is the participant 18 years or older?

◯ Yes
◯ No

Please specify the participant's date of birth

____

Calculated age

____

Is this the participant's first ANC visit?

◯ Yes
◯ No

Is the participant within the first 26 weeks of her pregnancy?

◯ Yes
◯ No

Is the participant within the first 20 weeks of her pregnancy?

◯ Yes
◯ No

Gestational weeks

____
(if unknown, enter 99)

Is the participant intending to deliver the baby at one of our collaborating MOUs?

◯ Yes
◯ No

Is the participant currently involved in any other ANC/HIV research trial?

◯ Yes
◯ No

Gigi RMS, *et al. BMJ Open* 2023; 13:e081562. doi: 10.1136/bmjopen-2023-081562

Calculated Eligibility Outcome

____
(1 = Eligible, 0 = Not Eligible)

END
The participant is not eligible for our research study

This will be the end of their participation. Please thank them for their time.

ELIGIBLE
The participant is eligible for our research study. Please select "Proceed" to start with the consenting process.

◯ Proceed

Consenting Process:

NOTE TO RA:
You will now start with the consenting process. Please make sure to do the following:

1. Read the consent form with the participant
2. Read in a language they prefer
3. Allow for questions
4. If willing to consent, sign all documents
5. Hand a signed copy (without PIN) to the patient

◯ Proceed

Did the participant provide a signed consent to participate in the research study?

◯ Yes
◯ No

Consent refusals

Reasons for refusal

☐ They have no time
☐ Scared
☐ In a different study
☐ Other

If "Other", please specify

Refusal date

____

END
Thank the participant for their time

Provided Consent

Consent date

____

Participant PIN

CONSENTED
The participant has agreed to provide consent. You will now allocate a study PIN to the participant. Please use the next available PIN on the hard copy enrollment log

◯ Proceed

Participant PIN

____

Participant PIN Verification

____

Pin match

____

PIN valid

____

ERROR
The PINs you entered did not match up

You have entered the following PINs

first pin: [participant_pin]
second pin: [participant_pin_verify]

ERROR
The PIN you entered is invalid for [site_name]

You have entered the following PINs

first pin: [participant_pin]
second pin: [participant_pin_verify]

Saving Instruction

You have completed the Screening and Enrollment process. Please make sure to check if all relevant fields have been selected and the information captured is accurate.

Once this is done, please select the "complete" option below and then select "Save & Exit".

Once you have done this you will be directed to the baseline Data.

Notes

Additional Notes

Baseline Data

Staff name

____

Today's Date

____

Start Time

____

Sociodemographics

NOTE TO RA:
You are about to start the Socio-demographics section. Please make sure to ask the questions as they appear on your tablet.

Please select "Proceed" to continue.

◯ Proceed

Sociodemographics

How would you describe yourself in terms of race?

◯ African
◯ Coloured
◯ Mixed Race
◯ White
◯ Indian
◯ No answer

What level of education did you complete?

◯ Less than Gr. 10
◯ Gr.10 or 11
◯ Gr.12
◯ Diploma
◯ Degree
◯ Refused to answer

Which best describes the type of house in which you live? Please choose one answer only:

◯ House or brick structure on a separate stand or yard or on a farm
◯ Traditional dwelling/hut/structure made of traditional materials
◯ Flat
◯ Town/cluster/semi-detached house (simplex, duplex or triplex)
◯ Unit in retirement village
◯ Dwelling/house/flat/room in backyard
◯ Informal dwelling/shack IN the backyard of a formal house
◯ Informal dwelling/shack NOT in backyard e.g. in an informal/squatter settlement or on farm
◯ Room/flatlet not in backyard but on a shared property e.g granny flat
◯ Caravan/tent
◯ Worker's hostel
◯ Other

If other, please specify.

What is the main material of your house walls? Please choose one answer only:

◯ Bricks & plaster/finished
◯ Bare brick/cement block
◯ Corrugated iron/zinc
◯ Wood
◯ Plastic
◯ Cardboard
◯ Mixture of mud and cement
◯ Wattle and daub
◯ Mud
◯ Other

If other, please specify

What is the main material of your house roof? Please choose one answer only:

◯ Tiles
◯ Corrugated iron/zinc
◯ Thatching
◯ Asbestos
◯ Plastic
◯ Cardboard
◯ Other

If other, please specify

What is your current relationship status?

◯ Married
◯ Steady partner
◯ Steady partner and Casual Partner(s)
◯ Casual Partner(s)
◯ No relationship

Do you live together with your partner?

◯ Yes
◯ No

Are you currently employed?

◯ Employed
◯ Self employed
◯ Not employed

What is your monthly personal income?

◯ None
◯ < 1000 ZAR per month
◯ 1001 - 5000 ZAR per month
◯ 5001 - 10 000 ZAR per month
◯ >10 000 ZAR per month

What is your household's main source of income?

◯ Personal income from employment \ self employment
◯ Income from partner
◯ Grants
◯ Other

Have you been outside of the Eastern Cape or country in the past 6 months?

◯ Yes
◯ No

Which provinces or country have you been to in the last 6 months?

Note to RA: please select all that apply

☐ Free State
☐ Gauteng
☐ Kwazulu-Natal
☐ Limpopo
☐ Mpumalanga
☐ Northern Cape
☐ North West
☐ Western Cape
☐ Outside South Africa

Has your partner/husband been outside of the Eastern Cape or country in the past 6 months?

◯ Yes
◯ No

Gigi RMS, *et al. BMJ Open* 2023; 13:e081562. doi: 10.1136/bmjopen-2023-081562

Which provinces or country has your partner/husband been to in the last 6 months?

Note to RA: Please select all that apply

☐ Free State
☐ Gauteng
☐ Kwazulu-Natal
☐ Limpopo
☐ Mpumalanga
☐ Northern Cape
☐ North West
☐ Western Cape
☐ Other country

What is the main source of drinking water for your household? Please choose one answer only:

◯ Piped (tap) water in dwelling
◯ Piped (tap) water on site or in yard
◯ Neighbour's tap
◯ Public or communal tap (either free or paid)
◯ Borehole on site
◯ Borehole off site/communal
◯ Rain water tank
◯ Water carrier/tanker
◯ Flowing water/stream/river
◯ Stagnant water/dam/pool
◯ Well
◯ Spring
◯ Bottled water
◯ Other

If other, please specify

What type of toilet does your household use? Please choose one answer only:

◯ Flush toilet (connected to sewage)
◯ Flush toilet (with septic tank)
◯ Chemical toilet
◯ Pit latrine with ventilation pipe
◯ Pit latrine without ventilation pipe
◯ Bucket toilet
◯ No facility/bush/field
◯ Other

If other, please specify

What is the main source of energy for cooking in your household? Please choose one answer only:

◯ Electricity from mains
◯ Electricity from generator
◯ Gas
◯ Paraffin
◯ Wood
◯ Coal
◯ Animal dung
◯ Solar energy
◯ Other

If other, please specify

Does your household have any of the following items in good working order? Read each item and indicate the presence of each

	Yes	No
Television	◯	◯
Gas or Electric stove	◯	◯
Fridge/freezer	◯	◯
Private motor vehicle in running condition	◯	◯
Bicycle	◯	◯
Bed	◯	◯
Sofa or sofa set	◯	◯
Kitchen sink	◯	◯

Do you think that you will need to borrow money to pay for healthcare during your pregnancy?

◯ Yes
◯ No

How much money did you spend coming to the clinic today (including transport costs, snacks while waiting etc.) ?

____
([RANDS])

Did you lose any money from your job because of coming to the clinic today?

◯ Yes
◯ No

If yes, how much money did you lose?

____
([RANDS])

How much time did you spend travelling to the clinic today (Hours)?

____
([HOURS])

How much time did you spend travelling to the clinic today (Minutes)?

____
([MINUTES])

Time spent travelling in minutes.

____

How much time do you normally spend waiting and seeing the doctor or nurse in a clinic such as this one (Hours)?

____
([HOURS])

How much time do you normally spend waiting and seeing the doctor or nurse in a clinic such as this one?

____
([MINUTES])

How much time do you normally spend waiting and seeing the doctor or nurse in a clinic such as this one in minutes?

____
([MINUTES])

Are you planning to wait for your results today?
(New question added @13/09/2022)

◯ Yes
◯ No

What is your main reason why you are not intending to wait today?
(New question added @13/09/2022)

◯ Have to get to work
◯ Have to get back to my kids/family
◯ Want to go to the shop
◯ Transport availability
◯ Lack of privacy
◯ Hungry
◯ No space to wait
◯ Not feeling well
◯ Boring
◯ Other

If "Other" , please specify.
(New question added @13/09/2022)

What would make you change your mind?
(New question added @13/09/2022)

Do you do any of the following activities in a lake / stream?

Note to RA: Please select multiple that apply

☐ Play
☐ Bath
☐ Wash blankets
☐ Do laundry
☐ Fish
☐ Collect water
☐ Crossing
☐ None

Behavioural Questionnaire

NOTE TO RA:
You just completed all questions related to socio-demographics. You are about to start with the Behavioral Questionnaire.

Please select "Proceed":

◯ Proceed

When was the last time you had sex?

◯ In the past week
◯ In the past month
◯ More than a month ago

Did you use a condom the last time you had sex?

◯ Yes
◯ No

Do you use a lubricant?

◯ Yes
◯ No

Can you please elaborate on the type of lubricant that you use?

____

What do you use to clean your vagina?

◯ Water only
◯ Soap and water
◯ Other household products
◯ Other

Please specify what other things you used on your vagina.

Do you do any vaginal douching?

◯ Yes
◯ No

Please specify

Do you do any form of vaginal cleansing?

◯ Yes
◯ No

Please specify

Do you use anything to clean inside your vagina?

◯ Yes
◯ No

Do you insert anything in your vagina for tightening?

◯ Yes
◯ No

Please specify

In the past 6 months, how many sexual partners did you have?

◯ One
◯ More than one

In the past 6 months, have you engaged in any of the following?
(Select ALL that apply)

☐ Vaginal sex
☐ Oral sex
☐ Anal sex

Have you recently agreed to sex even though you did not feel like to?

◯ Yes
◯ No

Note to RA: Discuss with participant counselling options

◯ Yes
◯ Participant doesn't need counselling

Please specify

In the past 6 months, have you been forced to have sex with anyone?

◯ Yes
◯ No

Note to RA: Discuss with participant counselling options

◯ Yes
◯ Participant doesn't need counselling

Please specify

In the past 6 months, have you received any benefits (money or goods) for sex?

◯ Yes
◯ No

Do you suspect your steady partner to have any other sex partners?

◯ Yes
◯ No
◯ Unsure

When did your last menstrual period start?

Note to RA: Please ask participant to give the most accurate date.

____

Just before I became pregnant.

NOTE: Please tick the statement that most applies to you:

◯ I wanted to have a baby
◯ I had mixed feelings about having a baby
◯ I did not want to have a baby

How many times have you been pregnant before your current pregnancy?

____

How many live children have you delivered?

____

Of the live births that you had, how many were normal vaginal delivery?

____

Of the live births that you had, how many were "emergency cesarean"?

____

Of the live births that you had, how many were "elective cesarean"?

____

Live birth Match

____

Note to RA: The numbers you have entered do not match. Please check again.

How many of your live birth's were premature?

____

How many of your live birth's were full term?

____

Delivery timing calc

____

Note: The numbers you have captured do not add up

Have you ever had an ectopic pregnancy?

◯ Yes
◯ No

Have you ever had a miscarriage?

◯ Yes
◯ No

Have you ever had a stillbirth?

◯ Yes
◯ No

Do you smoke cigarettes?

◯ Yes
◯ No

Have you used any of the following since you found out you were pregnant? (select multiple)
(Select ALL that apply.)

☐ Alcohol
☐ Tik
☐ Dagga
☐ Grandpa
☐ Other
☐ None

Please specify

Do you know your current HIV status?

◯ HIV negative (tested today by clinical staff)
◯ HIV positive on ART
◯ HIV positive, not on ART
◯ Don't know (never tested)
◯ Don't know (no yet tested today)

Was the participant newly diagnosed within the past week?

◯ Yes
◯ No

Can we test you for HIV today?

◯ Yes
◯ No

Unknown HIV Status:

Note to RA/ Nurse: HIV test needs to be conducted

◯ Proceed to test

HIV rapid test result:

◯ Positive
◯ Negative

HIV confirmatory test

◯ Positive
◯ Negative

Elisa blood barcode

____

Have you ever been treated for an STI in the last year?

◯ Yes, I had discharge
◯ Yes, I had ulcers
◯ Yes, I had genital warts
◯ Yes, no symptoms but notified by partner
◯ No

Does the participant have pre-existing diabetes?

◯ Yes
◯ No

Are you on treatment for your diabetes?

◯ Yes
◯ No

Does the participant have pre-existing hypertension?

◯ Yes
◯ No

Are you currently on medication for your hypertension?

◯ Yes
◯ No

Does the participant have pre-existing thyroid disease?

◯ Yes
◯ No

Is the participant taking medication for her thyroid disease?

◯ Yes
◯ No

Do you know your partner's HIV status?

◯ Yes, HIV positive on ART
◯ Yes, HIV positive but not on ART
◯ Yes, HIV negative
◯ No

You have completed the baseline questionnaire. Please make sure to log out of your REDCap account.

Once you have done this you can hand the process over to the nurse who will conduct the clinical history.

NOTES

Additional notes

Gigi RMS, *et al. BMJ Open* 2023; 13:e081562. doi: 10.1136/bmjopen-2023-081562

Physical Exam

Staff Name

____

Today's Date

____

Start time

____

You are about to administer the questions associated with the physical exam.

Please select "Proceed" to continue.

◯ Proceed

Clinical History

Do you currently have any of the following symptoms?

RA: Please select all that apply

☐ Abnormal vaginal discharge
☐ Pain during urination
☐ Lower abdominal pain
☐ Pain related to intercourse
☐ Vaginal bleeding related to intercourse
☐ Genital itchiness
☐ Any skin abnormalities
☐ None

How many days ago did your abnormal vaginal discharge start?

____

How many days ago did the pain during urination start?

____

Provide further details

Have you received treatment for these symptoms?

◯ Yes
◯ No

Where did you get the treatment from?

◯ Over the counter
◯ Healthcare facility
◯ Traditional healer

Please provide further details

If you were told you had an STI would you disclose to your partner(s)?

◯ Yes, to steady partner
◯ Yes, to casual partner(s)
◯ Yes, to all steady and casual partner(s)
◯ No

Co-Morbidities

You are about to start asking questions related to co-morbidities.

Please select "Proceed" to continue.

◯ Proceed

Did the participant screen positive for any TB symptoms?

◯ Yes
◯ No

The participant shows signs of TB. A specimen needs to be collected for further testing. Please select below to specify whether a specimen was collected successfully.

◯ Yes
◯ No

Instruction: Please record the specimen tracking number below

____

Are you on cotrimoxazole prophylaxes?

◯ Yes, on cotrimoxazole
◯ Yes, started today
◯ No

Did the participant start antiretroviral therapy today?

◯ Yes
◯ No

Specify reason for not starting

Was blood taken today for the participant's CD4 count?

◯ Yes
◯ No

Please record barcode for blood tube for CD4 count testing?

____

Is the participant's most recent CD4 count within the last 12 months available?

◯ Yes
◯ No

What was the date of the CD4 specimen collection?

____

What was the participant's most recent CD4 count?

____
(if no number listed, enter 9999)

Was blood taken today for the participant's viral load?

◯ Yes
◯ No

Please record barcode for blood tube for viral load testing?

____

Is the participant's most recent viral load within the past 12 months available?

◯ Yes
◯ No

What was the date of the viral load specimen collection?

____

What was the participant's most recent viral load?

____
(if no number listed, enter 0000)

Was blood taken today for the participant's viral load?

◯ Yes
◯ No

Please record the barcode for viral load testing?

____

Is the participant's most recent viral load available?

◯ Yes
◯ No

What was the date of the viral load specimen collection?

____

What was the participant's most recent viral load?

____

Which regimen for ART were you started on today?

◯ TLD
◯ TEE
◯ AZT/3TC/LPV
◯ Other

Which regimen for ART were you on so far?

◯ TLD
◯ TEE
◯ AZT/3TC/LPV
◯ Other

Has the regimen for ART been changed today?

◯ Yes
◯ No

To which regimen for ART has it been changed today?

◯ TLD
◯ TEE
◯ AZT/3TC/LPV
◯ Other

Please select "Proceed" to collect blood for viral load testing.

◯ Proceed

Did you successfully collect blood for viral load testing?

◯ Yes
◯ No

Please capture the barcode associated with the blood tube used for testing viral load.

____

Was a CD4 count test done?

◯ Yes
◯ Yes, but no result available
◯ Not done

Please specify last known CD4-cell count.

____

Was blood taken today for the participant's CD4 count ?

◯ Yes
◯ No

Please record the barcode for CD4 testing?

____

Is the participant's most recent CD4 available?

◯ Yes
◯ No

What was the date of the CD4 specimen collection?

____

What was the participant's most recent CD4 Count?

____

Has a syphilis test been done for the participant?

◯ Yes
◯ No

Instruction: Please conduct a rapid test for syphilis.

Specify if the participant agreed to testing / you managed to execute the test.

◯ Yes
◯ No

Which syphilis test have you used?

◯ Alere Syphilis TP (provided by FPD)
◯ HIV/Syphilis Duo (provided by FPD)
◯ No rapid test used only NHLS bloods for RPR
◯ Other please specify

Please specify

Syphilis result

◯ Positive
◯ Negative
◯ Indeterminate

Titer value 1:

____

Please collect blood for further syphilis testing and specify if the blood was collected successfully.

◯ Yes
◯ No

Treatment given

◯ Benzathine penicillin, 2.4 mU
◯ Out of stock

Please contact the study clinician and specify treatment given to participant

Please capture the barcode below.

____

Participant weight in kilograms

____

Participant height in cm

____

Participant systolic blood pressure

____

Participant diastolic blood pressure

____

How was Hemoglobin measured?

◯ Hb meter at the clinic
◯ Hb at NHLS

Please capture Hb result

____
(1 Decimal Place)

Please capture the barcode for Hb

____

Participant MUAC in cm

____
(1 Decimal Place)

Please collect participant's urine for later testing.

Ultrasound Results

You are about to start capturing information pertaining to the ultrasound results.

Please select "Proceed" to continue.

◯ Proceed

Ultra-sound scan date

____

Was the pregnancy confirmed?

◯ Yes, intra-uterine
◯ Yes, extra-uterine
◯ No

NOTE: Please refer immediately

NOTE
Due to the status of the pregnancy, the participant is no longer eligible to continue with the study. This is the end of their involvement in the study. Please thank them for their time. Also do the following:

- Save and Exit the form

- Complete a Study Note confirming termination of study participation

Please specify the number of foetus

____

You are about to capture the gestational age of the foetuses. Please select "Proceed" and then capture the number of weeks followed by days.

○ Proceed

Gestational age in weeks

____

Gestational age in days

____

Calc: Gestational age in days

____

EDD based on ultra-sound

____

Calc: Days to EDD

____

Calc assist for EDD

The number of days must be equal to 280.

____

Note to Nurse:

You did not enter either gestational age or EDD correctly.

Calc: Eligibility ________________________________

END
The participant is not eligible for our research study

This will be the end of their participation. Please thank them for their time.

STI Clinical Examination (To be done By Nurse)

You are about to capture information related to STI clinical examination.

Please select "Proceed" to continue

○ Proceed

During your examination, were there any signs of abnormal vaginal discharge?

◯ Yes
◯ No

During your examination, were there any signs of inguinal lymphadenopathy?

◯ Yes
◯ No

Are these bubo?

◯ Yes
◯ No

Note to RA: Please contact study clinician and specify treatment given to participant

During your examination, were there any signs of lower abdominal pain?

◯ Yes
◯ No

During your examination, where there any signs of scratch marks?

◯ Yes
◯ No

During your examination, where there any signs of skin conditions?

◯ Yes
◯ No

Please specify the nature of the skin conditions

During your examination, where there any other observations that need to be noted?

Urine Dipstick test results

You are about to capture the results from the dipstick testing

Please select "Proceed" to continue

◯ Proceed

Blood - Hemoglobin

◯ Negative
◯ Ca. 10
◯ Ca. 50
◯ Ca. 250/300

Blood - Erythrocytes

◯ Negative
◯ Ca. 5 -10
◯ Ca. 50
◯ Ca. 250/300

Urobilinogen

◯ Normal
◯ 2
◯ 4
◯ 8
◯ 12

Bilirubin

◯ Negative
◯ 1 plus
◯ 2 plus
◯ 3 plus
◯ Not available

Protein

◯ Negative
◯ 30
◯ 100
◯ 500

Nitrate

◯ Negative
◯ Positive

Keton

◯ Negative
◯ 1 plus
◯ 2 plus
◯ 3 plus
◯ Not available

Glucose

◯ Negative
◯ Normal
◯ 50
◯ 150
◯ 500
◯ ≥1000

pH

◯ 5
◯ 6
◯ 7
◯ 8
◯ 9
◯ Not available

SG

◯ 1.000
◯ 1.005
◯ 1.010
◯ 1.015
◯ 1.020
◯ 1.025
◯ 1.030
◯ Not available

Leucocytes

◯ Negative
◯ 25
◯ 75
◯ 500

NOTE
The participant's clinical gestational age is more than 20 weeks. They are not eligible to proceed with the study activities.

Please do the following:

1. Explain the reason for study termination
2. Complete the study electronic termination tool
3. Complete the study termination document and place in file

◯ End

NOTES

Additional Notes

You have completed capturing the information from the clinical exam. Please make sure to check that you have completed all the fields.

Please select "Complete" then "Save and Exit".

You will now proceed to collecting study specimens and randomization

Specimens and Randomization

Staff Name

____

Today's Date

____

Start time

____

Specimen Collection

NOTE
You will now start with the process of specimen collection. You will need to collect several specimens from the participant. These specimens need to be collected in the order in which they are presented here. The outcome of the randomization will have an impact on whether these specimens will be tested immediately or whether they will need to be prepared for storage.

The following specimens will need to be collected:
1. Vaginal loop to be used to prepare two slides
2. Vaginal swab to be used for STI testing
3. Vaginal swab to be used for profiling
4. Vaginal swab to be used for microbiome
5. Vaginal swab to be used for cytokine

◯ Done

NOTE
You will now start with the process of specimen collection. You will need to collect several specimens from the participant. These specimens need to be collected in the order in which they are presented here.

The following specimens will need to be collected:
1. 1 x Vaginal loop to be used to prepare two slides
2. 1 x Vaginal swab to be used for STI testing
3. 1 x Vaginal swab to be used for profiling
4. 2 x Vaginal swab to be used for microbiome
5. 1 x Vaginal swab to be used for cytokine

◯ Done

Vaginal Smear

Please specify the vaginal pH

____
(if not available, enter 99)

Gigi RMS, *et al. BMJ Open* 2023; 13:e081562. doi: 10.1136/bmjopen-2023-081562

Please select which pH strips are used to measure vaginal pH

◯ CardinalHealth pH Indicator Strips (range 3.6-6.1)
◯ pH Indicator Strips pH 0-14
◯ Natureland vaginal pH test (range 3.5-6.5)

You will need to use a single loop to collect vaginal smear on two glass slides for microscopy

◯ Done

Confirm the PIN associated with the first vaginal slide that will be used for Nugent score

◯ [participant_pin]-S1

Confirm the PIN associated with the second vaginal slide that will be used for yeast microscopy

◯ [participant_pin]-S2

Vaginal Swabs

NOTE
You will now collect four vaginal swabs. They will be used as follows:

1. STI testing (test for arms 1 and 2, store for arm 3)
2. Profiling (stored)
3. Microbiome (stored)
4, Cytokine

◯ Done

NOTE
You will now collect four vaginal swabs. They will be used as follows:

1. STI testing
2. Profiling (stored)
3. Microbiome (stored)
4, Cytokine

◯ Done

Please confirm the PIN associated with the urine for Schistosomiasis testing.
(2022/10/21 - Stopped collecting the urine specimen)

◯ [participant_pin] - UD1

Please confirm the PIN associated with the vaginal swab that will be used for STI testing.

◯ [participant_pin] - BV1

Please confirm the PIN associated with the vaginal swab that will be used for profiling.

◯ [participant_pin] - BV2

Please confirm the PIN associated with the vaginal swab that will be used for microbiome.

◯ [participant_pin] - BV3

Please confirm the PIN associated with the vaginal swab that will be used for cytokines.

◯ [participant_pin] - BV4

NOTE
You have finished the collection of the vaginal swabs. Please ensure specimens have been prepared for storage and shipment. The vaginal swab that is collected for STI testing should be kept aside following the outcome of the randomization. If the participant is in arm 1 or 2 the specimen should be used for immediate testing. If however, the participant is randomized to arm 3, you can store the specimen.

Please select "Proceed" to start the process of randomization

◯ Done

Randomization	◯ Arm 1<br>◯ Arm 2<br>◯ Arm 3

Activities Associated with "[randomization]"

NOTE
The participant has been randomized to "[randomization]". You will now need to do the following:

1. Prepare the STI swab for testing using the GeneXpert

◯ Done

NOTE:
The participant has been randomized to"[randomization]". You will now need to do the following:

1. Prepare the STI swab for testing using the GeneXpert
2. Screen for symptoms
3. Provide treatment and partner referral if positive

◯ Done

NOTE:
The participant has been randomized to "[randomization]". You will now need to do the following:

1. Screen for symptoms
2. Provide treatment and partner referral if positive

◯ Done

NOTE
You will now need to do the following:

1. Prepare the STI swab for testing using the GeneXpert

◯ Done

STI Results

	Positive	Negative
CT	◯	◯

NG	◯	◯
TV	◯	◯

NOTE: See Calculation:

The result from the STI test?

____
(0 = Negative, 1 = Positive, 2 = No result)

Date the result was obtained

____

Did the participant wait for her STI results?
(New question added @ 03/11/2022)

◯ Yes
◯ No

Symptomatic Screening Outcome Following Negative Test

The result from the GeneXpert was negative.

Was the participant reporting STI symptoms or showed symptoms during the clinical assessment?

◯ Yes
◯ No

Does the participant report any medication allergies?

◯ Yes
◯ No

Please contact study clinician before giving any treatment. Please specify discussed medication allergies and treatment plan with study clinician

The following treatment has been provided

☐ Azithromycin 1g stat dose
☐ Azithromycin 2g stat dose
☐ Ceftriaxone 250mg IM injection
☐ Ceftriaxone 1g IM injection
☐ Metronidazole 400mg bd x 1 week
☐ Metronidazole 2g stat dose
☐ Clotrimazole pessary and/or cream
☐ Trimethoprim/sulfamethoxazole 400/80 mg 2 tbl. bds for 5 days (bactrim)
☐ Ceftriaxone 500mg IM injection

Date treatment given

____

What made you change your mind about waiting for the results?
(New question added @13/09/2022)

Partner notification provided

◯ Yes, 1
◯ Yes, multiple
◯ No

Please explain why the partner notification note was not provided?

ELIGIBLE
The participant is eligible for our nested chlamydia case-control study. Please select "Proceed" to start with the consenting process.

◯ Proceed

Did the participant provide a signed consent to participate in the chlamydia case control study?

◯ Yes
◯ No

Reasons for refusal

☐ They have no time
☐ Scared
☐ In a different study
☐ Other

If "Other", please specify

Consent or refusal date

____

NOTES

Participant successfully enrolled __________________________________

Additional notes

You are done with all activities associated with "[randomization]". Please hand the tablet over to the RA to capture the remaining schedule dates.

You are done with all activities. Please hand the tablet over to the RA to capture the remaining schedule dates.

Scheduling

Scheduling of Dates Associated with [randomization].

NOTE
You are about to schedule dates associated with [randomization] participants.

Please select "Proceed".

◯ Proceed

Scheduling of Dates Associated

NOTE:
You are about to schedule dates associated with microbiome participants.

Please select "proceed"

◯ Proceed

Scheduling Dates for 3-Week ToC

NOTE: The participant tested positive and therefore we need to schedule a date, exactly 3-weeks from today to conduct a test-of-cure.

Calculator Assist

The number here must be equal to 21

____

Scheduling the 3-week ToC

NOTE:
Please schedule a date, 3 weeks from today treatment given. Please use the calculator assistance to ensure that you schedule a date exactly 21 days from today.

____

ERROR
The field does not equal to 21, please change it

Have you handed the TOC date to the participant?

◯ Yes
◯ No

Scheduling Dates Associated with ToC Reminder

Schedule date for REMINDER of 3-week ToC visit

____

Calculator Assist for scheduling ToC reminder date

The reminder phone call will be made 18 days following the treatment date. The number of days need to equal to 18.

____

ERROR
You did not enter the date correctly. The number should equal to 18. Please redo the date.

Scheduling Dates Associated with 3-Week ToC Missed Visit Date

NOTE:
You have successfully scheduled the reminder date.

Please select "proceed" to schedule the missed visit date for the 3 week ToC visit.

◯ Proceed

Schedule the date for the MISSED VISIT of the ToC visit.

This date should be 3 weeks after the date on which the participant received their test result.

____

Calculator Assist for scheduling 3-week ToC Missed Visit

The participant's time period allowed for attending a ToC will start 35 days after they received their result and will close 35 days after the date they received their result.

The number here must show 35

____

ERROR
You did not enter the date correctly. The number should equal to 35. Please redo the date.

NOTE:
You have successfully scheduled the 3-week ToC close date

Please select "proceed" to start scheduling the next visit dates

◯ Proceed

Dates Associated with reminder for the 28 Week call

NOTE:
You are about to schedule dates for the call reminder at 28 weeks.

Please select "Proceed".

◯ Proceed

Note:
Schedule the date for the 28 week call. We will contact each participant to ask the date for their 30 weeks clinic visit is.

____

Calculation assist for scheduling the 32-week reminder date.

This number must equal to 196

____

Days to call reminder

____

ERROR
The number you have entered does not match 196. Please select a different date so that the number equals to 196.

CONGRATULATIONS

You have finished scheduling all dates.

NOTES

Notes box

Gigi RMS, *et al. BMJ Open* 2023; 13:e081562. doi: 10.1136/bmjopen-2023-081562

BL specimens results

Staff

____

Date

____

Time

____

You are about to capture results of specimens collected during the baseline visit.

Please select "Proceed" to continue

◯ Proceed

Hb results received

◯ Yes
◯ No

Please capture Hb result

____

Please capture the barcode for Hb

____

Please capture the results of the sputum for TB testing

◯ MTB Negative
◯ MTB Positive Rifampicin Susceptible
◯ MTB Positive Rifampicin Resistant
◯ Not suitable
◯ Specimen missing
◯ Invalid
◯ Not applicable

Please contact participant

Please recollect specimen on participant next visit

CD4 count results received?

◯ Yes
◯ Clotted blood
◯ Missing

Please recollect blood or collect outcome from ART clinic

Please record the barcode for CD4 count testing

____

Please capture the result of the blood tube collected for CD4 count testing

____

Date sample for CD4 count was taken

____

Viral load results received?

◯ Yes
◯ Clotted blood
◯ Missing

Please recollect blood or collect outcome from ART clinic

Please capture the result of the blood tube collected for viral load testing

____

Date sample for viral load was taken

____

Please record barcode for viral load testing

____

Please capture the result of the syphilis testing

◯ RPR Negative
◯ RPR Positive
◯ RPR Indeterminate
◯ Not received

Please contact participant

____

Notes

Notes

UP Specimen Results

Staff

____

Date

____

Time

____

You are about to capture results of specimens collected during the baseline visit.

Please select "Proceed" to continue

◯ Proceed

Please capture the result for the Nugent score testing

◯ Slide reading not satisfactory
◯ Slide reading was satisfactory

____

Please capture the result for the Nugent score testing

____

Please specify if yeast was present

◯ Yes
◯ No

Please capture the result for the yeast infection testing

◯ Slide reading not satisfactory
◯ Slide reading was satisfactory

____

Please capture the result for the Yeast Infection testing

____

Please specify if candida was present

◯ Yes
◯ No

Please capture the result for the schistosomiasis

◯ Positive
◯ Negative
◯ Indeterminate

Please specify the result for the schistosomiasis

☐ Trace
☐ 1+
☐ 2+
☐ 3+
☐ 4+

____

Note:

Please notify the study clinician

Notes

Notes

Calling Reminders

Staff Name ____________________

Today's Date ____________________

Time ____________________

Opening date for Week 30-34 ____________________

Closing date for Week 30-34 ____________________

NOTE:
You are about to call a participant to remind them of a specific visit. Please make sure to do the following:

1. Obtain all relevant contact numbers for the participant on their record
2. Ensure that you have checked what the exact date is when the participant is expected to present
3. Make sure to give the participant a brief description of what will be done at the visit.
4. You will make up to 3 attempts to get hold of the participant.

Please select "Proceed"

◯ Proceed

The presentation dates are below:

TOC
Date: [baseline_arm_1][toc_3week]

WEEK 28 CALLING

Date: [baseline_arm_1][sched_28w_rem]

WEEK-32

30-34 Week Open Date: [week_28_arm_1][calling_wk30_34_open_date]

30-34 Week Close Date: [week_28_arm_1][calling_wk30_34_close_date]

30-34 Week Actual Date: [week_28_arm_1][week32_visit_date]

POST-NATAL VISIT

Visit open date: [predelivery_checki_arm_1][pd_remind_date_schedpd]

Visit close date: [predelivery_checki_arm_1][pd_close_date_schedpd]

Date of Delivery: [predelivery_checki_arm_1][pd_remind_date_delivery]

6-WEEK IMMUNIZATION VISIT

Actual Date: [predelivery_checki_arm_1][sixw_im_schedpd]

Please select the calling attempt

☐ First Attempt
☐ Second Attempt
☐ Third Attempt

Details of Calling Attempt 1

Outcome of the attempt

◯ Successful - Participant
◯ Successful - Family member
◯ Unsuccessful - Voicemail
◯ Unsuccessful - Invalid

Date of the attempt

____

Details of Calling Attempt 2

Outcome of the attempt

◯ Successful - Participant
◯ Successful - Family member
◯ Unsuccessful - Voicemail
◯ Unsuccessful - Invalid

Date of the attempt

____

Details of Calling Attempt 3

Outcome of the attempt

◯ Successful - Participant
◯ Successful - Family member
◯ Unsuccessful - Voicemail
◯ Unsuccessful - Invalid

Date of the attempt

____

Notes

Calling notes

Gigi RMS, *et al. BMJ Open* 2023; 13:e081562. doi: 10.1136/bmjopen-2023-081562

ToC Visit Activities

Staff Name ______________________

Today's date ______________________

Start time ______________________

NOTE
Did the participant present within the specified dates presented below:

Start: [baseline_arm_1][toc_reminder_date]

Actual: [baseline_arm_1][toc_3week]

End: [baseline_arm_1][arm1_toc_close_date]

◯ Yes
◯ No

The participant did not have a positive baseline STI result and therefore a ToC visit is not applicable. Activities associated with this visit will need to be captured under the "Ad-Hoc" tool

Note
You are about to start activities associated with the Test-of-Cure visit for participants in arm 1. The following activities are associated with this visit:

1. Collect 1 Loop with 2 slides
2. Collect 3 vaginal Swabs
- Test of Cure Test
- Profiling (Storage)
- Microbiome (Storage)
3. Running the Test-of-Cure
4. Collect Clinical History, Adherence and Disclosure data

Please select "Proceed"

◯ Proceed

Specimen Collection_ToC

NOTE
Please collect one vaginal loop and prepare 2 slides. Please remember to do the following:

1. Pack slides individually in their own package
2. Record the PIN on the outside of package
3. Complete the lab CRF with the matching PINs and test instructions

Select "Proceed" to confirm the PINs associated with the slides.

◯ Proceed

Did you manage to collect the vaginal loop?

◯ Yes
◯ No

Date of vaginal loop specimen collection

____

Please confirm the pin for the first vaginal swab that will be used for Nugent score

◯ [baseline_arm_1][participant_pin]-TL1

Please confirm the pin for the second vaginal swab that will be used for yeast microscopy

◯ [baseline_arm_1][participant_pin]-TL2

NOTE:
You are about the start with the process of collecting the following 3 vaginal swabs:

1. Swab to be used to conduct ToC (Immediately)
2. Swab for profiling
3. Swab to be used for microbiome

Please select "Proceed"

◯ Proceed

Please specify the vaginal pH

____

Please confirm the PIN associated with the vaginal swab that will be used for STI testing.

◯ [baseline_arm_1][participant_pin] - TCV1

Did you manage to collect the vaginal swab for profiling

◯ Yes
◯ No

Please confirm the PIN associated with the vaginal swab that will be used for profiling.

This must be stored

◯ [baseline_arm_1][participant_pin] - TCV2

Did you manage to collect the vaginal swab for microbiome testing?

◯ Yes
◯ No

Please confirm the PIN associated with the vaginal swab that will be used for microbiome.

This must be stored

◯ [baseline_arm_1][participant_pin] - TCV3

Date of specimen collection for vaginal swabs

____

NOTE
You have collected all specimens associated with this visit. Once you select the "Proceed" option below you will be directed to the start of the clinical history questionnaire. The completion of the questionnaire might take some time so it would be a good idea to start running the vaginal swab to conduct the Test of Cure in line with the below baseline results.

NG: [baseline_arm_1][sti_result_ng]

TV: [baseline_arm_1][sti_result_tv]

CT: [baseline_arm_1][sti_result_ct]

◯ Proceed

Clinical History Review

You are done trying to collect specimens. Because your were not able to collect a Vaginal Swab for STI testing you will not be able to run a test. Please proceed to completing the clinical history.

◯ Proceed

Do you currently have any of the following symptoms?
Multiple selection

☐ Abnormal vaginal discharge
☐ Pain during urination
☐ Lower abdominal pain
☐ None

When did these symptoms start for abnormal vaginal discharge?

◯ After previous visit
◯ Persistent since previous visit
◯ Recurrent since previous visit

When did these symptoms start for pain during urination?

◯ After previous visit
◯ Persistent since previous visit
◯ Recurrent since previous visit

Adherence

NOTE
The following questions pertain to adherence to the STI medication.

Select Proceed

◯ Proceed

Did you finish the whole course of treatment?

◯ Yes
◯ No

How many days did you take treatment for?

____

Did you throw up within 2 hours after taking any of the STI treatment?

◯ Yes
◯ No

Did you take any other non-chronic treatment at the time?

◯ Yes
◯ No

What type of treatment were you taking ?

NOTE
You are done with questions related to Adherence. You are about to start asking questions associated with Disclosure.

Please select "Proceed"

◯ Proceed

Disclosure

Did you have sex in the past month?

◯ Yes
◯ No

How many different male partners did you have sexual intercourse with in the past month ?

◯ 1
◯ 2
◯ More than 2 partners

Please specify how many partners?

____

What type of sex did you have with partner 1 (Husband/ Steady partner)?

☐ Vaginal
☐ Anal
☐ Oral

Did you use a condom the last time you had sex with this partner?

◯ Yes
◯ No

Did you notify him of your STI result?

◯ Yes I gave him the notification slip
◯ Yes I told him
◯ No

What was his reaction when you told him of your STI infection?

◯ Supportive
◯ Angry
◯ Violent
◯ Disengaged

How did disclosure affect your relationship?

◯ Continued as before
◯ Started using a condom
◯ He engaged with other partners
◯ He refused sex
◯ Relationship ended

Did he take the treatment?

◯ Yes
◯ No
◯ Don't know

Where did he seek treatment?

◯ Private
◯ Public
◯ Traditional

Why did you not notify this partner?

◯ I didn't feel it was necessary
◯ I am embarrassed
◯ I'm afraid he gets angry
◯ I'm afraid he gets violent
◯ I'm afraid he will end the relationship

What type of sex did you have with partner 2?

☐ Vaginal
☐ Anal
☐ Oral

Did you use a condom the last time you had sex with this partner?

◯ Yes
◯ No

Did you notify him of your STI result?

◯ Yes I gave him the notification slip
◯ Yes I told him
◯ No

What was his reaction when you told him of your STI infection?

◯ Supportive
◯ Angry
◯ Violant
◯ Disengaged

How did disclosure affect your relationship?

◯ Continued as before
◯ Started using a condom
◯ He engaged with other partners
◯ He refused sex
◯ Relationship ended

Did he take the treatment?

◯ Yes
◯ No
◯ Don't know

Where did he seek treatment?

◯ Private
◯ Public
◯ Traditional

Why did you not notify your partner?

◯ I didn't feel it was necessary
◯ I am embarrassed
◯ I'm afraid he gets angry
◯ I'm afraid he gets violent
◯ I'm afraid he will end the relationship

Did you tell anyone else of your STI infection?

◯ Yes
◯ No

Who did you tell?
(Select multiple)

☐ Family member
☐ Friend
☐ Healthcare worker
☐ Other

NOTE:
You have completed the ToC questionnaire. Please select "Proceed" to capture the outcome of the STI test.

◯ Proceed

ToC Outcome

	Positive	Negative	Did not test
CT	◯	◯	◯
NG	◯	◯	◯
TV	◯	◯	◯

NOTE: See calculation

The result from the STI result ______________ (1 = Positive, 0 = Negative)

Does the participant show any symptoms of an STI?

◯ Yes
◯ No

Please contact the study clinician and discuss treatment.

The participant tested/screened positive for an STI. Please specify the treatment that has been provided.

☐ Azithromycin 1g stat dose
☐ Azithromycin 2g stat dose
☐ Ceftriaxone 250mg IM injection
☐ Ceftriaxone 1g IM injection
☐ Metronidazole 400mg bd x 1 week
☐ Metronidazole 2g stat dose
☐ Clotrimazole pessary and/or cream
☐ Ceftriaxone 500mg IM injection

Date treatment given

____

Please specify if a partner notification has been given to the patient.

◯ Yes
◯ No

NOTE
The patient did not test positive or show any signs of an infection

Select "Proceed" to conclude visit

◯ Proceed

Notes

Additional notes

You have completed the ToC Visit Activities. Please make sure to check if all relevant fields have been selected and the information captured is accurate.

Once this is done, please select the "Complete" option below and then select "Save & Exit".

ToC specimens_result

You are about to capture the results of the first loop used for Nugent scoring.

Please select "Proceed" to continue

◯ Proceed

Was the reading satisfactory for the Nugent score?

◯ Yes
◯ No

____

Please specify the Nugent score

____

Please specify if candida was present

◯ Yes
◯ No

Additional comments

You are about to capture the results of the second loop used for smear microscopy.

Please select "Proceed" to continue

◯ Proceed

Was the reading satisfactory?

◯ Yes
◯ No

____

Please specify the Nugent score

____

Please specify if candida was present

◯ Yes
◯ No

Additional comments

Scheduling_Office

Scheduling the Dates Associated with the 32 Week Gestational Visit

The pregnancy (in days) is currently: ________________________________

NOTE
You are about the start scheduling dates associated with the 32-week visit. You will need to schedule the following associated dates:

1. Week 32 date - Actual visit

2. Week 32 reminder date

3. Week 30 date - Visit window opens

4. Week 35 date - Visit window closes

Select "Proceed" to start scheduling

◯ Proceed

Schedule the date for the 32 week gestational age, visit

Note to RA: please make sure that this date does not fall on Friday, weekend, and public holidays.

____

Calculate assist for 32-week visit

The number here must between 210 and 244.

____

Days Difference (the difference between 32 weeks & Gestational age)

____

Match ________________________________

The date you have entered does not meet the 93 day criteria. Does the intended or original date fall on a Friday weekend or public holiday?

◯ Yes
◯ No

ERROR
The numbers you have entered does not match. Please select a different date so that the numbers match.

Dates Associated with reminder for the 32 Week Gestational Age Visit

Dates associated with the 32 weeks gestational age missed visit

NOTE:
You have successfully scheduled the 32-week date.

We will need to contact the participant at least 3 days before the scheduled visit to remind them.

Select "Proceed" to schedule the reminder date for the 32 week visit.

◯ Proceed

Note:
Schedule the date for the 32 week reminder. We will contact each participant starting 3 days prior to their 32-week gestation date. That means the date scheduled here should be 3 days earlier then the scheduled date for the 32-week visit. If the date falls on a weekend choose the closest week date.

____

Calculation assist for scheduling the 32-week reminder date.

This number must be between 1 and 4.

____

ERROR
The date that you have entered is invalid. Please select a different date so that the number is less than or equal to 3.

NOTE:
You have successfully scheduled the 32-week reminder date.

Select "Proceed" to schedule the 32 week open visit date.

◯ Proceed

Schedule the date for the 32 weeks opening visit date.

Note: Participants will have from 30 weeks of gestation to present for their 32-week visit date.

____

Calculation Assist for scheduling the 32-Week opening visit date.

This number must equal to 210

____

ERROR
The number you have entered does not match 210. Please select a different date so that the number equals 210.

NOTE:
You have successfully scheduled the 32-week opening date.

Select "Proceed" to schedule the 32 weeks missed visit date.

◯ Proceed

Schedule the date for the 32 weeks missed visit date.

Note: Participants will have until 34 weeks of gestation to present for their 32-week visit date after which the visit will be closed out.

____

Calculation Assist for scheduling the 32-Week missed visit date.

This number must equal to 244

____

ERROR
The number you have entered does not match 244. Please select a different date so that the number equals to 244.

Estimated Delivery Date

You are about the schedule the Estimated Delivery Date.

Please select "proceed"

◯ Proceed

Estimated Delivery Date

____

Days difference between estimated date of delivery and gestational age

____

Calculation Assist for scheduling the Estimated Date for Delivery date.

This number must equal to [edod_calc]

____

Match ______________________________

ERROR
The number you have entered does not match. Please select a different date so that the numbers match

You have completed all the scheduling dates.

Please check that all dates entered comply with the "calculation assistance".

You are about the schedule dates associated with the following events:

1. Pre-birth check-inn

◯ Proceed

Check-In Calling at 37 Weeks

Proceed to the check-in calling date

◯ Proceed

Check-in calling date

____

Calculation Assist for check-in calling date

This number must equal to 259

____

NOTE
The date you have entered is incorrect. Please make sure that the numbers correspond.

CONGRATULATIONS

You have finished scheduling all dates.

32-Week Visit Activities

Staff name

____

Today's date

____

Start time

____

Did the participant present within the dates presented below:

30-34 Week Start Date: [week_28_arm_1][sched_32w_open_date]

30-34 Week Actual Date: [week_28_arm_1][week32_visit_date]

30-34 Week Closing Date: [week_28_arm_1][sched_32w_mv_date]

◯ Yes
◯ No

Open ad-hoc visit to capture relevant information

Specimen Collection_32-Week

NOTE
You will now start with the process of specimen collection. You will need to collect several specimens from the participant. These specimens need to be collected in the order in which they are presented here.

The following specimens will need to be collected:
1. 1 x Vaginal loop to be used to prepare two slides
2. 1 x Vaginal swab to be used for STI testing (Arm 2 and Microbiome (Empilweni): immediate testing; Arm 1 and 3: Storage)
3. 1 x Vaginal swab to be used for profiling
4. 2 x Vaginal swab to be used for microbiome
5. 1 x Vaginal swab to be used for cytokine

◯ Proceed

Vaginal Smear

Please specify the vaginal pH

____
(if not available, enter 99)

Please select which pH strips are used to measure vaginal pH

◯ CardinalHealth pH Indicator Strips (range 3.6-6.1)
◯ pH Indicator Strips pH 0-14
◯ Natureland vaginal pH test (range 3.5-6.5)

You will need to use a single loop to collect vaginal smear on two glass slides for microscopy
(if not available, enter 99)

◯ Done

Confirm PIN associated with the first Vaginal Loop to be used to test for Nugent score

◯ [baseline_arm_1][participant_pin] - WL1

Confirm PIN associated with the second Vaginal Loop to be used to test for Yeast microscopy

◯ [baseline_arm_1][participant_pin] - WL2

Vaginal Swabs

NOTE
You will now collect four vaginal swabs. They will be used as follows:

1. STI testing (Arm 2 and Microbiome (Empilweni): immediate testing; Arm 1 and 3: Storage)
2. Profiling (stored)
3. Microbiome (stored)
4, Cytokine (stored)

◯ Done

Confirm PIN associated with the vaginal swab to be used to test for STI

◯ [baseline_arm_1][participant_pin] - WV1

Please confirm PIN associated with the vaginal swab to be used for Profiling

◯ [baseline_arm_1][participant_pin] - WV2

Please confirm PIN associated with the vaginal swab to be used for microbiome

◯ [baseline_arm_1][participant_pin] - WV3

Please confirm the PIN associated with the vaginal swab that will be used for cytokines.

◯ [baseline_arm_1][participant_pin] - WV4

NOTE
The participant is in arm 2 and therefore an immediate STI test is conducted at the 32-week visit. Please prepare the swab for testing before you continue to the questionnaires.

◯ Proceed

NOTE
You are done with all specimen collection and will now proceed to administering the clinical history.

Please select "Proceed"

◯ Proceed

Gigi RMS, *et al. BMJ Open* 2023; 13:e081562. doi: 10.1136/bmjopen-2023-081562

Supplemental material BMJ Publishing Group Limited (BMJ) disclaims all liability and responsibility arising from any reliance placed on this supplemental material which has been supplied by the author(s) BMJ Open

Clinical History Review

Have you been to the clinic since the last visit with us?

◯ Yes
◯ No

What was the purpose of your visit?

☐ ANC Visit
☐ HIV/ART
☐ STI Treatment
☐ Other

Summary notes from visit

Have you used any of the following since the first study visit?
Select multiple

☐ Alcohol
☐ Tik
☐ Dagga
☐ Grandpa
☐ Other
☐ None

Please specify other drugs used?

Do you currently have any of the following symptoms?

RA: Please select all that apply

☐ Abnormal vaginal discharge
☐ Pain during urination
☐ Lower abdominal pain
☐ Pain related to intercourse
☐ Vaginal bleeding related to intercourse
☐ Genital itchiness
☐ Any skin abnormalities
☐ None

When did these symptoms start for abnormal vaginal discharge?

◯ After previous visit
◯ Persistent since previous visit
◯ Recurrent since previous visit

Gigi RMS, *et al. BMJ Open* 2023; 13:e081562. doi: 10.1136/bmjopen-2023-081562

Supplemental material BMJ Publishing Group Limited (BMJ) disclaims all liability and responsibility arising from any reliance placed on this supplemental material which has been supplied by the author(s) BMJ Open

When did these symptoms start for pain during urination?

◯ After previous visit
◯ Persistent since previous visit
◯ Recurrent since previous visit

When did these symptoms start for the lower abdominal pain?

◯ After previous visit
◯ Persistent since previous visit
◯ Recurrent since previous visit

When did these symptoms start for the pain related to intercourse?

◯ After previous visit
◯ Persistent since previous visit
◯ Recurrent since previous visit

When did these symptoms start for vaginal bleeding related to intercourse?

◯ After previous visit
◯ Persistent since previous visit
◯ Recurrent since previous visit

When did these symptoms start for genital itchiness?

◯ After previous visit
◯ Persistent since previous visit
◯ Recurrent since previous visit

Please specify any skin abnormalities

Baseline Treatment Date:
[baseline_arm_1][sti_treatment_date]

TOC Treatment Date:
[toc_arm_1_arm_1][toc_sti_treatment_date]

Did the participant receive any STI treatment at their last study visit?

◯ Yes
◯ No

Are you planning to wait for your results today?
(New question added @13/09/2022)

◯ Yes
◯ No

What is your main reason why you are not intending to wait today?
(New question added @13/09/2022)

◯ Have to get to work
◯ Have to get back to my kids/family
◯ Want to go to the shop
◯ Transport availability
◯ Lack of privacy
◯ Hungry
◯ No space to wait
◯ Not feeling well
◯ Boring
◯ Other

If "Other", please specify.
(New question added @13/09/2022)

What would make you change your mind?
(New question added @13/09/2022)

You are done with questions associated with clinical history review. You will now start with questions associated with Adherence.

◯ Proceed

Adherence

Did you finish the whole course of STI treatment

◯ Yes
◯ No

How many days did you take treatment for?

____

Did you throw up within 2 hours after taking any of the STI treatment

◯ Yes
◯ No

Did you take any other non-chronic treatment at the time

◯ Yes
◯ No

What type of treatment

You are done with questions associated with the adherence. You are about to start asking questions associated with disclosure.

◯ Proceed

Disclosure

Did you notify your partner of your STI result?

◯ Yes I gave him the notification slip
◯ Yes I told him
◯ No

What was his reaction when you told him of your STI infection

◯ Supportive
◯ Angry
◯ Violent
◯ Disengaged

How did disclosure affect your relationship?

◯ Continued as before
◯ Started using a condom
◯ He engaged with other partners
◯ He refused sex
◯ Relationship ended

Did he take treatment?

◯ Yes
◯ No
◯ I don't know

Where did he seek treatment

◯ Private
◯ Public
◯ Traditional

Why did you not notify your partner?

◯ I didn't feel it was necessary
◯ I am embarrassed
◯ I'm afraid he gets angry
◯ I'm afraid he gets violent
◯ I'm afraid he will end the relationship

Did you tell anyone else of your STI infection?

◯ Yes
◯ No

Who did you tell?

☐ Family member
☐ Friend
☐ HCW
☐ Other

Behavioral Questionnaire

NOTE TO RA:
You just completed all questions related to Disclosure. You are about to start with the Behavioral Questionnaire.

Please select "Proceed":

◯ Proceed

Did you have sex since the last visit?

◯ Yes
◯ No

How many different male partners did you have sexual intercourse with in the past month?

◯ 1
◯ 2
◯ more than 2

Were any of these new partners than the ones from the last visit

◯ Yes
◯ No

What type of sex did you have with partner 1 (Husband/ Steady partner)?

☐ Vaginal
☐ Anal
☐ Oral

Did you use a condom the last time you had sex with partner 1 (Husband/ Steady partner)?

◯ Yes
◯ No

What type of sex did you have with partner 2?

☐ Vaginal
☐ Anal
☐ Oral

Did you use a condom the last time you had sex with partner 2?

◯ Yes
◯ No

What type of sex did you have with the rest of the partners?

☐ Vaginal
☐ Anal
☐ Oral

Did you use a condom the last time you had sex with one of them?

◯ Yes
◯ No

Where are you planning to deliver?

◯ Frere
◯ CMH
◯ Nontyantyambo
◯ Empilweni
◯ Bisho
◯ Other

Please specify

You are done with the questions associated with Behavioral Questionnaire. You will now start asking questions associated with the Physical Examination.

◯ Proceed

Physical Examination

Weight of mother

____

Systolic blood pressure

____

Diastolic blood pressure

____

How was Hemoglobin measured?

◯ Hb meter at the clinic
◯ Hb at NHLS

Please capture Hb result"

____

Please capture the barcode for Hb

____

Fundal height

____

Progression of pregnancy

◯ Progressing normal
◯ Abnormality detected

Provide further details of abnormality

During your examination, were there any signs of abnormal vaginal discharge?

◯ Yes
◯ No

During your examination, were there any signs of inguinal lymphadenopathy?

◯ Yes
◯ No

Are these bubo?

◯ Yes
◯ No

Note to RA: Please contact the study clinician and specify treatment given to the participant

During your examination, were there any signs of lower abdominal pain?

◯ Yes
◯ No

During your examination, where there any signs of scratch marks?

◯ Yes
◯ No

During your examination, were there any signs of skin conditions?

◯ Yes
◯ No

Please specify the nature of the skin conditions

During your examination, were there any other observations that need to be noted?

You have completed the questions associated with the Physical Examination. You will now start capturing the results from the rapid tests.

◯ Proceed

Rapid Test Results

Do you know your current HIV status?

◯ HIV negative (tested today by clinical staff)
◯ HIV positive on ART
◯ HIV positive, not on ART
◯ Don't know (never tested)
◯ Don't know (no yet tested today)

Was the participant newly diagnosed with HIV today

◯ Yes
◯ No

Please conduct an HIV Rapid test and capture the result below

◯ Positive
◯ Negative

Please conduct a confirmatory HIV Rapid test and capture the result below

◯ Positive
◯ Negative

Did you collect a tube of blood for CD4 count?

◯ Yes
◯ No

Please record barcode for blood tube for CD4 count testing?

____

Is the participant's most recent CD4 count since Baseline available?

◯ Yes
◯ No

What was the date of the CD4 specimen collection?

____

What was the participant's most recent CD4 count?

____
(if no number listed, enter 9999)

Did you collect a tube of blood for viral load?

◯ Yes
◯ No

Please record barcode for blood tube for viral load testing?

____

Is the participant's most recent viral load since Baseline available?

◯ Yes
◯ No

What was the date of the viral load specimen collection?

____

What was the participant's most recent viral load?

____
(if no number listed, enter 0000)

Which regimen for ART were you started on today?

◯ TLD
◯ TEE
◯ AZT/3TC/LPV
◯ Other

Which regimen for ART were you on so far?

◯ TLD
◯ TEE
◯ AZT/3TC/LPV
◯ Other

Has the regimen for ART been changed today?

◯ Yes
◯ No

To which regimen for ART has it been changed today?

◯ TLD
◯ TEE
◯ AZT/3TC/LPV
◯ Other

Has a syphilis test been done for the participant?

◯ Yes
◯ No

Which syphilis test have you used?

◯ Alere Syphilis TP (provided by FPD)
◯ HIV/Syphilis Duo (provided by FPD)
◯ No rapid test used only NHLS bloods for RPR
◯ Other please specify

Please specify

Syphilis result.

◯ Positive
◯ Negative
◯ Indeterminate

Titer value 1:

____
(If RPR is non-reactive, enter 0)

Blood needs to be collected for further syphilis testing. Please confirm if blood was collected.

◯ Yes
◯ No

Collect blood for RPR and capture barcode PIN below

____

Treatment given

◯ Benzathine penicillin 2.4 MU IM weekly x3
◯ Out of stock

Please contact study clinician and specify treatment given

Please collect participant's urine for testing

Urine Dipstick test results

You are about to capture the results from the dipstick testing

Please select "Proceed" to continue

◯ Proceed

Blood - Hemoglobin

◯ Negative
◯ Ca. 10
◯ Ca. 50
◯ Ca. 250/300

Blood - Erythrocytes

◯ Negative
◯ Ca. 5 -10
◯ Ca. 50
◯ Ca. 250/300

Urobilinogen

◯ Normal
◯ 2
◯ 4
◯ 8
◯ 12

Bilirubin

◯ Negative
◯ 1 plus
◯ 2 plus
◯ 3 plus
◯ Not available

Protein

◯ Negative
◯ 30
◯ 100
◯ 500

Nitrate

◯ Negative
◯ Positive

Keton

◯ Negative
◯ 1 plus
◯ 2 plus
◯ 3 plus
◯ Not available

Glucose

◯ Negative
◯ Normal
◯ 50
◯ 150
◯ 500
◯ ≥1000

pH

◯ 5
◯ 6
◯ 7
◯ 8
◯ 9
◯ Not available

SG

◯ 1.000
◯ 1.005
◯ 1.010
◯ 1.015
◯ 1.020
◯ 1.025
◯ 1.030
◯ Not available

Leucocytes

◯ Negative
◯ 25
◯ 75
◯ 500

STI Results and Screening

The participant is in arm 2 or for Empilweni and therefore you are about to capture results for the STI testing.

◯ Proceed

STI Test Outcome

The previous STI results of the participants are:

Baseline
NG:[baseline_arm_1][sti_result_ng]

CT:[baseline_arm_1][sti_result_ct]

TV:[baseline_arm_1][sti_result_tv]

TOC
NG:[toc_arm_1_arm_1][toc_ng]

CT:[toc_arm_1_arm_1][toc_ct]

TV:[toc_arm_1_arm_1][toc_tv]

	Negative	Positive
CT	◯	◯
NG	◯	◯
TV	◯	◯

STI Calculation _ w32

____

Date the result was obtained.

____

Did the participant wait for her STI results?
(New question added @ 03/11/2022)

◯ Yes
◯ No

Was the participant reporting STI symptoms or showed symptoms during the clinical assessment?

◯ Yes
◯ No

Does the participant report any medication allergies?

◯ Yes
◯ No

Please contact the study clinician before giving any treatment. Please specify discussed medication allergies and treatment plan with the study clinician

The following treatment has been provided

☐ Azithromycin 1g stat dose
☐ Azithromycin 2g stat dose
☐ Ceftriaxone 250mg IM injection
☐ Ceftriaxone 1g IM injection
☐ Metronidazole 400mg bd x 1 week
☐ Metronidazole 2g stat dose
☐ Clotrimazole pessary and/or cream
☐ Trimethoprim/sulfamethoxazole 400/80 mg 2 tbl. bds for 5 days (bactrim)
☐ Ceftriaxone 500mg IM injection

Date treatment given

____

What made you change your mind about waiting for the results?
(New question added @13/09/2022)

Partner notification provided

◯ Yes, 1
◯ Yes, multiple
◯ No

Please explain why the partner notification note was not provided?

You have completed capturing the information from the 32 week exam. Please make sure to check that you have completed all the fields.

Please select "Complete" then "Save and Exit".

Notes

Additional notes

32W specimens results

You are about to capture the results of specimens collected during the 32-week visit

Please select "Proceed" to continue

◯ Proceed

Hb Results received

◯ Yes
◯ No

Please capture the Hb result

____

Please capture the barcode for Hb

____

Please specify whether the reading was satisfactory for the loop used for Nugent score

◯ Yes
◯ No

____

Please capture the score for the loop used for Nugent scoring

____

Please specify if candida was present for the loop used for Nugent scoring

◯ Yes
◯ No

Please specify whether the reading was satisfactory for the loop used for yeast microscopy

◯ Yes
◯ No

____

Please capture the nugent score for the loop used for yeast microscopy

____

Gigi RMS, *et al. BMJ Open* 2023; 13:e081562. doi: 10.1136/bmjopen-2023-081562

Please specify if candida was present for the loop used for yeast microscopy

◯ Yes
◯ No

Please capture the results for the blood used for Syphilis testing

◯ RPR Negative
◯ RPR Positive
◯ RPR Indeterminate

____

Please capture the result for viral load testing

____

Is the participant's most recent viral load available?

◯ Yes
◯ No

What was the date of the viral load specimen collection?

____

What was the participant's most recent viral load?

____

____

Notes

Notes

Calling Reminders_2

Staff Name ______________________

Today's Date ______________________

Time ______________________

The presentation dates are below:

TOC
Date: [baseline_arm_1][toc_3week]

WEEK 28 CALLING

Date: [baseline_arm_1][sched_28w_rem]

WEEK-32

30-34 Week Actual Date: [week_28_arm_1][week32_visit_date]

Call In Check

Week 37 Call In Check: [week_28_arm_1][cic_date]

POST-NATAL VISIT

Visit open date: [predelivery_checki_arm_1][pd_remind_date_schedpd]

Visit close date: [predelivery_checki_arm_1][pd_close_date_schedpd]

Date of Delivery: [predelivery_checki_arm_1][pd_remind_date_delivery]

6-WEEK IMMUNIZATION VISIT

Actual Date: [predelivery_checki_arm_1][sixw_im_schedpd]

NOTE:
You are about to call a participant to remind them of a specific visit. Please make sure to do the following:

1. Obtain all relevant contact numbers for the participant on their record
2. Ensure that you have checked what the exact date is when the participant is expected to present
3. Make sure to give the participant a brief description of what will be done at the visit.
4. You will make up to 3 attempts to get hold of the participant.

Please select "Proceed"

◯ Proceed

Please select the calling attempt

☐ First Attempt
☐ Second Attempt
☐ Third Attempt

Details of Calling Attempt 1

Outcome of the attempt

◯ Successful - Participant
◯ Successful - Family member
◯ Unsuccessful - Voicemail
◯ Unsuccessful - Invalid

Date of the attempt

____

Did she deliver?

◯ Yes
◯ No

Capture delivery Date

NOTE: instruct to come to the site.

____

Details of Calling Attempt 2

Outcome of the attempt

◯ Successful - Participant
◯ Successful - Family member
◯ Unsuccessful - Voicemail
◯ Unsuccessful - Invalid

Date of the attempt

____

Did she deliver?

◯ Yes
◯ No

Capture delivery Date

NOTE: instruct to come to the site.

____

Details of Calling Attempt 3

Outcome of the attempt

◯ Successful - Participant
◯ Successful - Family member
◯ Unsuccessful - Voicemail
◯ Unsuccessful - Invalid

Date of the attempt

____

Did she deliver?

◯ Yes
◯ No

Capture delivery Date

NOTE: instruct to come to the site.

____

Notes

Calling notes

Schedule calling for 38 weeks

NOTE FW: Please make sure that you call the participant once per week.

◯ Proceed

Did you schedule the 38 weeks call

◯ Yes
◯ No

Please select the calling attempt

☐ First Attempt
☐ Second Attempt
☐ Third Attempt

Details of Calling Attempt 1

Outcome of the attempt

◯ Successful - Participant
◯ Successful - Family member
◯ Unsuccessful - Voicemail
◯ Unsuccessful - Invalid

Date of the attempt

____

Did she deliver?

◯ Yes
◯ No

Details of Calling Attempt 2

Outcome of the attempt

◯ Successful - Participant
◯ Successful - Family member
◯ Unsuccessful - Voicemail
◯ Unsuccessful - Invalid

Date of the attempt

____

Did she deliver?

◯ Yes
◯ No

Details of Calling Attempt 3

Outcome of the attempt

◯ Successful - Participant
◯ Successful - Family member
◯ Unsuccessful - Voicemail
◯ Unsuccessful - Invalid

Date of the attempt

____

Did she deliver?

◯ Yes
◯ No

Schedule calling for 39 weeks

NOTE FW: Please make sure that you call the participant once per week.

◯ Proceed

Did you schedule the 39 weeks call

◯ Yes
◯ No

Please select the calling attempt

☐ First Attempt
☐ Second Attempt
☐ Third Attempt

Details of Calling Attempt 1

Outcome of the attempt

◯ Successful - Participant
◯ Successful - Family member
◯ Unsuccessful - Voicemail
◯ Unsuccessful - Invalid

Date of the attempt

____

Did she deliver?

◯ Yes
◯ No

Capture delivery Date

NOTE: instruct to come to the site.

____

Details of Calling Attempt 2

Outcome of the attempt

◯ Successful - Participant
◯ Successful - Family member
◯ Unsuccessful - Voicemail
◯ Unsuccessful - Invalid

Date of the attempt

____

Did she deliver?

◯ Yes
◯ No

Details of Calling Attempt 3

Outcome of the attempt

◯ Successful - Participant
◯ Successful - Family member
◯ Unsuccessful - Voicemail
◯ Unsuccessful - Invalid

Date of the attempt

____

Gigi RMS, *et al. BMJ Open* 2023; 13:e081562. doi: 10.1136/bmjopen-2023-081562

Did she deliver?

◯ Yes
◯ No

Calling notes

Schedule calling for 40 weeks

NOTE FW: Please make sure that you call the participant once per week.

◯ Proceed

Please select the calling attempt

☐ First Attempt
☐ Second Attempt
☐ Third Attempt

Did you schedule a call for 40 weeks

◯ Yes
◯ No

Details of Calling Attempt 1

Outcome of the attempt

◯ Successful - Participant
◯ Successful - Family member
◯ Unsuccessful - Voicemail
◯ Unsuccessful - Invalid

Date of the attempt

____

Did she deliver?

◯ Yes
◯ No

Capture delivery Date

NOTE: instruct to come to the site.

____

Details of Calling Attempt 2

Outcome of the attempt

○ Successful - Participant
○ Successful - Family member
○ Unsuccessful - Voicemail
○ Unsuccessful - Invalid

Date of the attempt

____

Did she deliver?

○ Yes
○ No

Details of Calling Attempt 3

Outcome of the attempt

○ Successful - Participant
○ Successful - Family member
○ Unsuccessful - Voicemail
○ Unsuccessful - Invalid

Date of the attempt

____

Calling notes

Did she deliver?

○ Yes
○ No

Schedule calling for 41 weeks

NOTE FW: Please make sure that you call the participant once per week.

○ Proceed

Please select the calling attempt

☐ First Attempt
☐ Second Attempt
☐ Third Attempt

Supplemental material BMJ Publishing Group Limited (BMJ) disclaims all liability and responsibility arising from any reliance placed on this supplemental material which has been supplied by the author(s) BMJ Open

Did you schedule a call for 41 weeks

◯ Yes
◯ No

Details of Calling Attempt 1

Outcome of the attempt

◯ Successful - Participant
◯ Successful - Family member
◯ Unsuccessful - Voicemail
◯ Unsuccessful - Invalid

Date of the attempt

____

Details of Calling Attempt 2

Outcome of the attempt

◯ Successful - Participant
◯ Successful - Family member
◯ Unsuccessful - Voicemail
◯ Unsuccessful - Invalid

Date of the attempt

____

Did she deliver?

◯ Yes
◯ No

Details of Calling Attempt 3

Outcome of the attempt

◯ Successful - Participant
◯ Successful - Family member
◯ Unsuccessful - Voicemail
◯ Unsuccessful - Invalid

Date of the attempt

____

Calling notes

Gigi RMS, *et al. BMJ Open* 2023; 13:e081562. doi: 10.1136/bmjopen-2023-081562

Did she deliver?

◯ Yes
◯ No

Capture delivery Date

NOTE: instruct to come to the site.

____

Schedule calling for 41 weeks (293 days)

NOTE FW: Please make sure that you call the participant once per week.

◯ Proceed

Did you schedule a call for 41 weeks

◯ Yes
◯ No

Please select the calling attempt

☐ First Attempt
☐ Second Attempt
☐ Third Attempt

Details of Calling Attempt 1

Outcome of the attempt

◯ Successful - Participant
◯ Successful - Family member
◯ Unsuccessful - Voicemail
◯ Unsuccessful - Invalid

Calling notes

Did she deliver?

◯ Yes
◯ No

Capture delivery Date

NOTE: instruct to come to the site.

____

Schedule calling for 42 weeks (296 days)

NOTE FW: Please make sure that you call the participant once per week.

◯ Proceed

Please select the calling attempt

☐ First Attempt
☐ Second Attempt
☐ Third Attempt

Did you schedule a call for 42 weeks

◯ Yes
◯ No

Details of Calling Attempt 1

Outcome of the attempt

◯ Successful - Participant
◯ Successful - Family member
◯ Unsuccessful - Voicemail
◯ Unsuccessful - Invalid

Date of the attempt

____

Did she deliver?

◯ Yes
◯ No

Details of Calling Attempt 2

Outcome of the attempt

◯ Successful - Participant
◯ Successful - Family member
◯ Unsuccessful - Voicemail
◯ Unsuccessful - Invalid

Date of the attempt

____

Did she deliver?

◯ Yes
◯ No

Details of Calling Attempt 3

Outcome of the attempt

◯ Successful - Participant
◯ Successful - Family member
◯ Unsuccessful - Voicemail
◯ Unsuccessful - Invalid

Date of the attempt

____

Outcome of the call

Did the participant deliver?

◯ Yes
◯ No

Calling notes

Did she deliver?

◯ Yes
◯ No

Capture delivery Date

NOTE: instruct to come to the site.

____

Scheduling_Post_Delivery_Activities using Updated EDD

You are about the schedule dates associated with the following events:

1. Post-Delivery Appointment
2. 6-Weeks Immunization Appointment

◯ Proceed

Post-Delivery Study Visit

You will now schedule dates associated with the post-delivery study visit.

The following dates are associated with this visit:
1. Calling reminder date / Visit opening date
2. Visit closing date

Select "Proceed" to continue

◯ Proceed

Please capture the date of delivery

____

Please schedule the date for the post-delivery reminder call.

Please note that the Delivery date is [pd_remind_date_delivery]. The reminder call will happen 1 day following delivery.

____

Calculation Assist for Post Delivery reminder call

This number must equal to: 1

____

Match

____

NOTE
The date you have entered is incorrect. Please make sure that the numbers correspond.

The post-delivery closing date.

____

Calculation Assist for Post Delivery closing date

The participant will have 14 days post-delivery to present at the clinic. The delivery date was [pd_remind_date_delivery]. This number must therefore equal to: 14

____

Match

____

NOTE
The date you have entered is incorrect. Please make sure that the numbers correspond.

Facility delivered

◯ Frere
◯ CMH
◯ Nontyantyambo
◯ Empilweni
◯ Bisho
◯ Other

Please specify the facility of delivery

____

6-Week Immunization Visit

NOTE:
In this section you will schedule all dates associated with the 6-Week Immunization Study Visit. These dates will include:

1. Calling reminder date for 6-Weeks Immunization visit
2. Scheduled date of 6-Weeks Immunization visit
3. Closing date for attending the 6-Weeks Immunization visit

Select Proceed to continue

◯ Proceed

Please schedule the date for the 6-weeks immunization reminder

____

Calculation Assist for 6-weeks immunization reminder. We will call all patients 5 weeks (35 days) following their delivery date. The updated delivery date for the participant was [calling_delivery_date_37weeks].

This number must therefore equal to: 35 and 40

____

Match

____

NOTE
The date you have entered is incorrect. Please make sure that the numbers correspond.

Please schedule the date for the 6-weeks immunization visit. This visit is scheduled to take place 6 weeks (42 days) following delivery. The updated delivery date is [calling_delivery_date_37weeks]

____

Calculation Assist for 6-weeks immunization visit

This number must equal to: 42

____

Match

____

NOTE
The date you have entered is incorrect. Please make sure that the numbers correspond.

Please schedule the date for the 6-weeks immunization visit closing date.

____

Calculation Assist for 6-Weeks Immunization visit Close Date. Mothers will have up to 8 weeks post delivery to attend this visit. This means 56 days following the delivery.

This number must equal to: 56

____

Match

____

NOTE
The date you have entered is incorrect. Please make sure that the numbers correspond.

CONGRATULATIONS

You have finished scheduling all dates.

Gigi RMS, *et al. BMJ Open* 2023; 13:e081562. doi: 10.1136/bmjopen-2023-081562

Birth Register Data

Participant PIN

[baseline_arm_1][participant_pin]

Staff name

____

Today's date

____

Start time

____

You are about to capture data retrieved from the birth registry. Please select "Proceed" to start

◯ Proceed

Delivery Details

Delivery site

◯ Frere
◯ CMH
◯ Nontyantyambo
◯ Empilweni
◯ Bisho
◯ Other

Please specify name of delivery facility

Clinic file number

____

Delivery date

____

Calculated gestational age

____
(Added @22/03/2023)

Please specify the number of babies during pregnancy

◯ 1
◯ 2
◯ 3

Outcome type for baby 1

◯ Live birth
◯ Still birth
◯ Early Neonatal Death

Outcome type for baby 2

◯ Live birth
◯ Still birth
◯ Early Neonatal Death

Outcome type for baby 3

◯ Live birth
◯ Still birth
◯ Early Neonatal Death

Type of delivery for baby 1

◯ Born before arrival
◯ Normal Vaginal Delivery
◯ Assisted Vaginal Delivery
◯ Elective Cesarean Section
◯ Emergency Cesarean Section

Type of delivery for baby 2

◯ Born before arrival
◯ Normal Vaginal Delivery
◯ Assisted Vaginal Delivery
◯ Elective Cesarean Section
◯ Emergency Cesarean Section

Type of delivery for baby 3

◯ Born before arrival
◯ Normal Vaginal Delivery
◯ Assisted Vaginal Delivery
◯ Elective Cesarean Section
◯ Emergency Cesarean Section

Please specify reason

Please specify reason

Please specify reason

Gender - Baby 1

○ Female
○ Male

Gender - Baby 2

○ Female
○ Male

Gender - Baby 3

○ Female
○ Male

Complications in labor/Delivery

	Yes	No
Induction of labour	○	○
Antepartun haemorrhage	○	○
Post Partum haemorrhage	○	○
Severe pre-eclampsia	○	○
Eclampsia	○	○
Prolonged rupture of membranes	○	○
Ruptured uterus	○	○
Sepsis	○	○
Obstructed or prolonged labour	○	○
Retained Placenta	○	○
Manual removal of placenta	○	○

Maternal outcome

○ Live
○ Death

APGAR score at 5 minutes for baby 1

____

APGAR score at 5 minutes for baby 2

____

APGAR score at 5 minutes for baby 3

____

Birth weight for baby 1 in grams

____

Birth weight for baby 2 in grams

____

Birth weight for baby 3 in grams

____

Did you breastfeed your baby/ies within 1 hour of giving birth?

◯ Yes
◯ No

Infant feeding
(New question added @15/11/2022)

◯ Exclusive Breast Feeding (EBF)
◯ Exclusive Formula Feeding (EFF)

Any birth defects to note for baby 1

◯ Yes
◯ No

Any birth defects to note for baby 2

◯ Yes
◯ No

Any birth defects to note for baby 3

◯ Yes
◯ No

Please specify

Remarks outcome

Maternal outcome

You have completed the Birth register. Please make sure to check if all relevant fields have been selected and the information captured is accurate.

Once this is done, please select the "Complete" option below and then select "Save & Exit".

Post-Natal Visit Activities

Staff name ________________________________

Today's date ________________________________

Start time ________________________________

Post-Natal Visit

The participant was scheduled to present within the two dates below. Please specify if the participant presented within this timeframe.

Visit open date: [predelivery_checki_arm_1][pd_remind_date_delivery]

Visit close date: [predelivery_checki_arm_1][pd_close_date_schedpd]

◯ Yes
◯ No

You are about to administer the questions associated with the post natal visit.

Please select "Proceed"

◯ Proceed?

Clinical History Review

Have you been to the clinic since the last visit with us?

◯ Yes
◯ No

What was the purpose of your visit?

☐ ANC Visit
☐ HIV/ART
☐ STI Treatment
☐ Other

Summary notes from the visit

Were there any abnormalities/complications since your last study visit regarding your pregnancy and delivery or did you receive any non-chronic treatment?

◯ Yes
◯ No

Please specify

Have you used any of the following since the first study visit?
Select multiple

☐ Alcohol
☐ Tik
☐ Dagga
☐ Grandpa
☐ Other
☐ None

Please specify other drugs used?

The Baseline STI results of the participants are:

NG: [baseline_arm_1][sti_result_ng]

CT: [baseline_arm_1][sti_result_ct]

TV: [baseline_arm_1][sti_result_tv]

The TOC STI results of the participants are:

NG: [toc_arm_1_arm_1][toc_ng]

CT: [toc_arm_1_arm_1][toc_ct]

TV: [toc_arm_1_arm_1][toc_tv]

The Week 32 STI results of the participants are:

CT: [3034_weeks_arm_1][w32_ct_res]

NG: [3034_weeks_arm_1][w32_ng_res]

TV: [3034_weeks_arm_1][w32_tv_res]

Did the participant receive any STI treatment at their last study visit?

◯ Yes
◯ No

Adherence

You are done with questions associated with the clinical history review. You will now start with questions associated with Adherence.

◯ Proceed

Did you finish the whole course of STI treatment?

◯ Yes
◯ No

How many days did you take treatment for?

____

Did you throw up within 2 hours after taking any of the STI treatment?

◯ Yes
◯ No

Did you take any other non-chronic treatment at the time?

◯ Yes
◯ No

What type of treatment

Disclosure

You are done with questions associated with the adherence. You are about to start asking questions associated with disclosure.

◯ Proceed

Did you notify your partner of your STI result?

◯ Yes I gave him the notification slip
◯ Yes I told him
◯ No

What was his reaction when you told him of your STI infection

◯ Supportive
◯ Angry
◯ Violent
◯ Disengaged

How did disclosure affect your relationship?

◯ Continued as before
◯ Started using a condom
◯ He engaged with other partners
◯ He refused sex
◯ Relationship ended

Did he take treatment?

◯ Yes
◯ No
◯ I don't know

Where did he seek treatment?

◯ Private
◯ Public
◯ Traditional

Why did you not notify your partner?

◯ I didn't feel it was necessary
◯ I am embarrassed
◯ I'm afraid he gets angry
◯ I'm afraid he gets violent
◯ I'm afraid he will end the relationship

Did you tell anyone else of your STI infection?

◯ Yes
◯ No

Did you tell anyone else of your STI infection?

◯ Yes
◯ No

Who did you tell?

☐ Family member
☐ Friend
☐ HCW
☐ Other

Delivery Details of Infant

Facility delivered

◯ Frere
◯ CMH
◯ Nontyantyambo
◯ Empilweni
◯ Bisho
◯ Other

Please specify facility of delivery

Date of delivery

____

Calculated gestational age

____
(Added @22/03/2023)

Please specify the number of babies during pregnancy

◯ 1
◯ 2
◯ 3

Outcome type for baby 1

◯ Live birth
◯ Still birth
◯ Early Neonatal Death

Outcome type for baby 2

◯ Live birth
◯ Still birth
◯ Early Neonatal Death

Outcome type for baby 3

◯ Live birth
◯ Still birth
◯ Early Neonatal Death

Type of delivery for baby 1

◯ Born before arrival
◯ Normal Vaginal Delivery
◯ Assisted Vaginal Delivery
◯ Elective Cesarean Section
◯ Emergency Cesarean Section

Type of delivery for baby 2

◯ Born before arrival
◯ Normal Vaginal Delivery
◯ Assisted Vaginal Delivery
◯ Elective Cesarean Section
◯ Emergency Cesarean Section

Type of delivery for baby 3

◯ Born before arrival
◯ Normal Vaginal Delivery
◯ Assisted Vaginal Delivery
◯ Elective Cesarean Section
◯ Emergency Cesarean Section

Please specify reason

Please specify reason

Please specify reason

Gender - Baby 1

◯ Female
◯ Male

Gender - Baby 2

◯ Female
◯ Male

Gender - Baby 3

◯ Female
◯ Male

Maternal outcome

◯ Live
◯ Death

Specify

APGAR score at 5 minutes for baby 1

Note to RA: Check on Road to Health

____
(if no number listed, enter 99)

APGAR score at 5 minutes for baby 2
Note to RA: Check on Road to Health

____
(if no number listed, enter 99)

APGAR score at 5 minutes for baby 3
Note to RA: Check on Road to Health

____
(if no number listed, enter 99)

Birth weight in grams for baby 1

Note to RA: Check on Road to Health

____

Birth weight in grams for baby 2

Note to RA: Check on Road to Health

____

Birth Weight in grams for baby 3

Note to RA: Check on Road to Health

____

Newborn problems

Note to RA: Check on Road to Health

☐ Birth defects
☐ Hypoxic brain injury
☐ Convulsions /fits
☐ Jaundice
☐ None

Please Specify

Was the baby exposed to HIV?
(Added @29/03/2023)

◯ Yes
◯ No

Was Nevirapine given to the baby/babies

◯ Yes
◯ No

Was birth PCR done for the baby/babies

◯ Yes
◯ No

NOTE: If not taken by birth facility please take blood for PCR.

PCR Barcode for the baby/baby 1

____

NOTE: If not taken by birth facility please take blood for PCR.

PCR Barcode for the baby/baby 2

____

NOTE: If not taken by birth facility please take blood for PCR.

PCR Barcode for the baby/baby 3

____

Result of birth PCR for baby 1

◯ Positive
◯ Negative
◯ Indeterminate
◯ Not yet available

Result of birth PCR for baby 2

◯ Positive
◯ Negative
◯ Indeterminate
◯ Not yet available

Result of birth PCR for baby 3

◯ Positive
◯ Negative
◯ Indeterminate
◯ Not yet available

Call clinician and make a note about this.

Was eye ointment given to the baby 1

◯ Yes
◯ No
◯ Don't know

Was eye ointment given to the baby 2

◯ Yes
◯ No
◯ Don't know

Was eye ointment given to the baby 3

◯ Yes
◯ No
◯ Don't know

Please specify to how many babies and which one

Gigi RMS, *et al. BMJ Open* 2023; 13:e081562. doi: 10.1136/bmjopen-2023-081562

Was the baby/babies admitted to hospital following delivery

◯ Yes
◯ No

Please specify the details about the reason for admission, number of babies admitted and which babies

Does baby 1 have any of the following symptoms?

☐ Cough
☐ Runny nose
☐ Eye discharge
☐ Sneezing
☐ None

Does baby 2 have any of the following symptoms?

☐ Cough
☐ Runny nose
☐ Eye discharge
☐ Sneezing
☐ None

Does baby 3 have any of the following symptoms

☐ Cough
☐ Runny nose
☐ Eye discharge
☐ Sneezing
☐ None

Is the baby/babies receiving any treatment at the moment

◯ Yes
◯ No

Please specify

Feeding methods

◯ Breastfeeding
◯ Formula feeding
◯ Mixed

Gigi RMS, *et al. BMJ Open* 2023; 13:e081562. doi: 10.1136/bmjopen-2023-081562

Delivery details of Mother

Is the baby present with the biological mother

◯ Yes
◯ No

Please specify

Are you currently taking any treatment

◯ Yes
◯ No

Please specify

Have you had sexual intercourse since delivery of your baby?

◯ Yes
◯ No

Do you have any of the following symptoms?

☐ Discharge
☐ Pain when urinating
☐ None

Please specify

You have completed all the questions associated with this visit. You will now start with the process of specimen collection. You will need to collect the following specimens:

From the mother you will need to collect 3 vaginal swabs:
- Vaginal Swab 1 for STI testing (Storage)
- Vaginal Swab 2 for Microbiome (Storage)
- Vaginal Swab 3 for Profiling (Storage)

From the baby you need to collect:
- Nasopharyngeal swab

- Conjunctival

Select "Proceed" to capture the information associated with these specimens

◯ Proceed

Please confirm the barcode for the vaginal swab collected for STI testing

◯ [baseline_arm_1][participant_pin]-PNV1

Please confirm the barcode for the vaginal swab collected for Microbiome

◯ [baseline_arm_1][participant_pin]-PNV3

Please confirm the Barcode for the Vaginal Swab collected for Profiling

◯ [baseline_arm_1][participant_pin]-PNV2

Please confirm the Barcode for the Nasopharyngeal swab (right nose) baby 1

◯ [baseline_arm_1][participant_pin]-PNB1N1

Please confirm the Barcode for the Nasopharyngeal swab (left nose) baby 1

◯ [baseline_arm_1][participant_pin]-PNB1N2

Please confirm the barcode for the Nasopharyngeal swab (right nose) baby 2

◯ [baseline_arm_1][participant_pin]-PNB2N1

Please confirm the barcode for the Nasopharyngeal swab (left nose) baby 2

◯ [baseline_arm_1][participant_pin]-PNB2N2

Please confirm the barcode for the Nasopharyngeal swab (right nose) baby 3

◯ [baseline_arm_1][participant_pin]-PNB3N1

Please confirm the barcode for the Nasopharyngeal swab (left nose) baby 3

◯ [baseline_arm_1][participant_pin]-PNB3N2

Please confirm the Barcode for the Nasopharyngeal swab for STI testing baby 1

◯ [baseline_arm_1][participant_pin]-PNB1N1

Please confirm the barcode for the Conjunctival swab (right eye) baby 1

◯ [baseline_arm_1][participant_pin]-PNB1C1

Please confirm the barcode for the Conjunctival swab (left eye) baby 1

◯ [baseline_arm_1][participant_pin]-PNB1C2

Please confirm the barcode for the Conjunctival swab (right eye) baby 2

◯ [baseline_arm_1][participant_pin]-PNB2C1

Please confirm the barcode for the Conjunctival swab (left eye) baby 2

◯ [baseline_arm_1][participant_pin]-PNB2C2

Please confirm the barcode for the Conjunctival swab (right eye) baby 3

◯ [baseline_arm_1][participant_pin]-PNB3C1

Please confirm the barcode for the Conjunctival swab (left eye) baby 3

◯ [baseline_arm_1][participant_pin]-PNB3C2

Please specify the vaginal pH

____

Please select which pH strips are used to measure vaginal pH

◯ CardinalHealth pH Indicator Strips (range 3.6-6.1)
◯ pH Indicator Strips pH 0-14
◯ Natureland vaginal pH test (range 3.5-6.5)

Did you give the participant the study voucher?

◯ Yes
◯ No

You have completed capturing the Post-Natal information. Please make sure to check that you have completed all the fields.

Please give the participant 6 weeks immunization visit date as per schedule.

Please select "Complete" then "Save and Exit".

Notes

Additional notes

Post-Natal specimens Results

Date

____

Staff name

____

You are about to capture the results of the specimens collected during the post natal visit

Please select "Proceed" to continue

◯ Proceed

Receive Date ________________________________

Test date (Mother) ________________________________

STI results from the mother

	Positive	Negative
CT	◯	◯
NG	◯	◯
TV	◯	◯

STI result, mother_ calc

____

Test date (Baby) ________________________________

STI Results from Baby 1

	Positive	Negative
CT (Right Nose)	◯	◯
NG (Right Nose)	◯	◯
TV (Right Nose)	◯	◯
CT (Left Nose)	◯	◯
NG (Left Nose)	◯	◯
TV (Left Nose)	◯	◯

CT (Right Eye)	◯	◯
NG (Right Eye)	◯	◯
TV (Right Eye)	◯	◯
CT (Left Eye)	◯	◯
NG (Left Eye)	◯	◯
TV (Left Eye)	◯	◯

STI Results from Baby 2

	Positive	Negative
CT - Right Nose	◯	◯
NG - Right Nose	◯	◯
TV - Right Nose	◯	◯
CT - Left Nose	◯	◯
NG Left Nose	◯	◯
TV - Left Nose	◯	◯
CT - Right Eye	◯	◯
NG - Right Eye	◯	◯
TV - Right Eye	◯	◯
CT - Left Eye	◯	◯
NG - Left Eye	◯	◯
TV - Left Eye	◯	◯

STI Results from Baby 3

	Positive	Negative
CT - Right Nose	◯	◯
NG - Right Nose	◯	◯
TV - Right Nose	◯	◯
CT - Left Nose	◯	◯
NG - Left Nose	◯	◯
TV - Left Nose	◯	◯
CT - Right Eye	◯	◯
NG - Right Eye	◯	◯
TV - Right Eye	◯	◯
CT - Left Eye	◯	◯
NG - Left Eye	◯	◯
TV - Left Eye	◯	◯

Result of birth PCR for baby 1

◯ Positive
◯ Negative
◯ Indeterminate
◯ Not yet available

Result of birth PCR for baby 2

◯ Positive
◯ Negative
◯ Indeterminate
◯ Not yet available

Result of birth PCR for baby 3

◯ Positive
◯ Negative
◯ Indeterminate
◯ Not yet available

Notes

Notes

BMJ Publishing Group Limited (BMJ) disclaims all liability and responsibility arising from any reliance placed on this supplemental material which has been supplied by the author(s)

6-Week Immunization Visit Activities

Staff name

____

Today's date

____

Time

____

Did the mother present within the specified dates below:

Start Date: [predelivery_checki_arm_1][sixweek_remind_schedpd]

Actual Date: [predelivery_checki_arm_1][sixw_im_schedpd]

End Date: [predelivery_checki_arm_1][sixw_im_close_schedpd]

◯ Yes
◯ No

You are about to administer the questions associated with 6-weeks immunization visit.

Please select "Proceed"

◯ Proceed

How many babies were delivered?

◯ 1
◯ 2
◯ 3

Was baby 1 admitted to hospital since the last study visit

◯ Yes
◯ No

Was baby 2 admitted to hospital following delivery

◯ Yes
◯ No

Was baby 3 admitted to hospital following delivery

◯ Yes
◯ No

Please specify

Does baby 1 have any of the following symptoms?

☐ Cough
☐ Runny nose
☐ Eye discharge
☐ Sneezing
☐ None

Does baby 2 have any of the following symptoms?

☐ Cough
☐ Runny nose
☐ Eye discharge
☐ Sneezing
☐ None

Does baby 3 have any of the following symptoms?

☐ Cough
☐ Runny nose
☐ Eye discharge
☐ Sneezing
☐ None

Are any of the babies receiving any treatment at the moment

Feeding methods

☐ Breastfeeding
☐ Formula feeding
☐ Mixed

Have you or the baby been to the clinic since the last visit with us?

◯ Yes
◯ No

What was the purpose of your visit?

☐ ANC Visit
☐ HIV/ART
☐ STI Treatment
☐ Other

Summary notes from the visit

Do you know your current HIV status?

☐ HIV negative (tested today by clinical staff)
☐ HIV positive on ART
☐ Known HIV positive, not on ART
☐ Newly diagnosed HIV positive (tested today by clinical staff)
☐ Don't know (never tested)
☐ Don't know (no yet tested today)

Please conduct a HIV Rapid test and capture the result below

◯ Positive
◯ Negative

Please conduct a confirmatory HIV Rapid test and capture the result below

◯ Positive
◯ Negative

HIV PCR result of baby 1

◯ Positive
◯ Negative
◯ No result

Please record barcode for blood and HIV PCR

____

HIV PCR result of baby 2

◯ Positive
◯ Negative
◯ No result

Please record barcode for blood and HIV PCR

____

HIV PCR result of baby 3

◯ Positive
◯ Negative
◯ No result

Please record barcode for blood and HIV PCR

____

NOTE
You have collected all specimens associated with this visit. Once you select the "Proceed" option below you will be

CT: [post_natal_arm_1][sti_result_ct]

NG: [post_natal_arm_1][sti_result_ng]

TV: [post_natal_arm_1][sti_result_tv]

◯ Proceed

STI result, mother_ calc

____

Does the participant report any medication allergies?

◯ Yes
◯ No

Please contact the study clinician before giving any treatment. Please specify discussed medication allergies and treatment plan with the study clinician

The following treatment has been provided

☐ Azithromycin 1g stat dose
☐ Azithromycin 2g stat dose
☐ Ceftriaxone 250mg IM injection
☐ Ceftriaxone 1g IM injection
☐ Metronidazole 400mg bd x 1 week
☐ Metronidazole 2g stat dose
☐ Clotrimazole pessary and/or cream
☐ Trimethoprim/sulfamethoxazole 400/80 mg 2 tbl. bds for 5 days (bactrim)

Date treatment given

____

Partner notification provided

◯ Yes, 1
◯ Yes, multiple
◯ No

Please explain why the partner notification note was not provided?

The mother tested positive for an STI at the Post Natal visit. You need to collect a Nasal Pharyngeal swab for baby 1. Did you manage to collect this specimen?

◯ Yes
◯ No

Please confirm the PIN for the Nasal Pharyngeal swab for baby 1.

◯ [baseline_arm_1][participant_pin]-NPB1

The mother tested positive for an STI at the Post Natal visit. You need to collect a Nasal Pharyngeal swab for baby 2. Did you manage to collect this specimen?

◯ Yes
◯ No

Please confirm the PIN for the Nasal Pharyngeal swab for baby 2 .

◯ [baseline_arm_1][participant_pin]-NPB2

The mother tested positive for an STI at the Post Natal visit. You need to collect a Nasal Pharyngeal swab for baby 3. Did you manage to collect this specimen?

◯ Yes
◯ No

Please confirm the PIN for the Nasal Pharyngeal swab for baby 3.

◯ [baseline_arm_1][participant_pin]-NPB3

You have completed capturing the Post-Natal information. Please make sure to check that you have completed all the fields.

Please select "Unverified" then "Save and Exit".

Notes

Additional notes

Adverse Outcomes

Staff Name ______________________

Today's date ______________________

Start time ______________________

Was there an adverse birth outcome?

◯ Yes
◯ No

Was there a serious adverse event

◯ Yes
◯ No

Early loss of baby

What type of early loss?

◯ Miscarriage
◯ Ectopic
◯ Termination of pregnancy
◯ Still Born

Date

____

Ectopic pregnancy

Date of surgery

____

Termination pregnancy

Date

____

Reviewed by site PI

◯ Yes
◯ No

Date Reviewed ______________________

Review Notes ____________________

Name of Reviewer ◯ Remco Peters

You have completed capturing the adverse outcomes information. Please make sure to check that you have completed all the fields.

Please select "Complete" then "Save and Exit".

Notes

Additional notes

Activities Associated with Visit

Staff Details

Staff Name

____

Today's Date

____

Start time

____

Presentation Outcome

Presentation outcome.

Did the participant present at the study site for this visit?

◯ Yes
◯ No

Activities Associated with ToC for Arm 1

You are about to facilitate activities associated with the 4-week ToC. You will need to execute the following:

1. Collect Specimens
2. Run a STI test
3. Conduct clinical history and behavioral questionnaire
4. Symptom screening if negative test
5. Treatment and partner referral if positive test

◯ Proceed

Activities Associated with 32 Week Visit

You are about to facilitate activities associated with the 32 week visit. You will need to execute the following:

1. Collect Specimens
2. Run a STI test
3. Conduct clinical history and behavioral questionnaire
4. Symptom screening if negative test
5. Treatment and partner referral if positive test

◯ Proceed

You are about to facilitate activities associated with the 32 week visit. You will need to execute the following:

1. Collect Specimens
2. Conduct clinical history and behavioral questionnaire
3. Symptom screening
4. Treatment and partner referral if positive screening

◯ Proceed

Activities Associated with the First Postnatal Visit

You are about to facilitate activities associated with the the 1st post natal visit. You will need to execute the following:

1. Determine the presentation date (Only proceed when its 14 days after the delivery date)
2. Collect pregnancy and birth outcomes data (Discharge Summary and/or Road to Health Card)
3. Conduct mother and child clinical examination and history questionnaire
4. Specimen collection for mother and child

◯ Proceed

Pregnancy and Birth Outcome Data

You are about to start with the pregnancy and birth outcome data capturing.

You can use the discharge summary and road to health as your data sources.

Select "Proceed" below to display the pregnancy and birth outcome details

◯ Proceed

Delivery date ________________________________

Mother and Baby Clinical Examination and History

You are done capturing the pregnancy and birth outcome data.

The next step is to capture the mother and baby clinical examination and history details.

Select "Proceed" below to display the questionnaire.

◯ Proceed

Scheduling the 6 week Immunization Date

Schedule a 6 week immunization.

Use the below date assist to schedule the 6 week immunization date. ________________________________

The below field must be equal to 42.

Use the date field above to ensure that the current field is equal to 42.

____

Collection of Vaginal Loops

You will need to collect a single vaginal loop that will be used to prepare two slides. Once collected you will need to prepare the slides for storage.

◯ Proceed

Date of collection of vaginal loops

____

....

Confirm the pin associated with the first vaginal loop that will be used for .....

◯ [baseline_arm_1][participant_pin]-FL1

Confirm the PIN associated with the second vaginal loop that will be used for .....

◯ [baseline_arm_1][participant_pin]-FL2

Storage of Loops

You have collected both slides. Before commencing with the rest of the specimens, please make sure to do the following:

1. Slides are individually packed in their own package
2. Record PIN on outside of package
3. Complete the lab CRF with matching PINs and test instructions

◯ Proceed

Vaginal Swab Collection

You will now collect 3 vaginal swabs. They will be used as follows:

1. STI testing (1st Specimen)
2. Profiling (2nd Specimen)
3. Microbiome (3rd Specimen)

◯ Proceed

....

Date of specimen collection for vaginal swabs

____

Confirm the PIN associated with the first vaginal swab

◯ [baseline_arm_1][participant_pin]-FV1

.....

Confirm the PIN associated with the second vaginal swab

◯ [baseline_arm_1][participant_pin]-FV2

Confirm the PIN associated with the third vaginal swab

◯ [baseline_arm_1][participant_pin]-FV3

Nasopharyngeal Swab Collection

You are about to collect the Nasopharyngeal swab on the Baby.

Collect the specimen and confirm the PIN below.

◯ [baseline_arm_1][participant_pin]-NS1

GeneXpert Testing for the First Specimen

You will now start with the testing of the first vaginal swab specimen.

Follow the below steps:
1. Ensure that the GeneXpert Machine is switched-on. Perform a quikc qulaity check on the machine.
2. Load the specimen and run the machine.
3. Conduct Clinical History and Behavioural Questionnaire

Select "Start Test" when ready to run the test.

◯ Start Test

Clinical History and Behavioural Questionnaire

You have started running the STI test.

Conduct clinical history and behavioural questionnaire. Select "Proceed" to display the questionnaire

◯ Proceed

How often have you had sex since the last time we saw you?

◯ 0
◯ 1 to 5 times a week
◯ More than 5 times a week

STI Results

	Positive	Negative
NG	◯	◯
TV	◯	◯
CT	◯	◯

The participant tested positive for an STI.

The next step is to administer treatment with the participant.

Select "Proceed" to display treatment options.

◯ Proceed

.....

The next step is to screen the patient for STI symptoms

Is the participant symptomatic?

◯ Yes
◯ No

The participant screened positive for at least a single STI symptom

The next step is to administer treatment with the participant.

Select "Proceed" to display treatment options.

◯ Proceed

Treament and Partner Notification

Select the treatment regimen you administered to the participant

◯ Azithromycin
◯ Doxycyclin
◯ Ceftriaxone
◯ Metronidazole

Did you administer partner notification treatment?

◯ Yes
◯ No

Storage Processes

You have collected all required specimens.

You can now prepare the specimens for storage, follow the below steps:
1. Ensure that each specimen has a complete Lab CRF
2. Pack the Lab CRFs in the specimen container
3. Ensure that the Lab CRF is complete and specimens are stored according to the storage requirements.

Select "Confirm" after perform tha above specimen procedures.

◯ Confirm

Notes

Additional notes

Gigi RMS, *et al. BMJ Open* 2023; 13:e081562. doi: 10.1136/bmjopen-2023-081562

Close-out

Staff member ______________________

Date ______________________

Participant ID: [baseline_arm_1][participant_pin_verify]

TERMINATION DETAILS

Date of termination ______________________

Study Time-Point
- ◯ BASELINE
- ◯ TOC
- ◯ 32 WEEKS
- ◯ POST-NATAL VISIT

Reason for termination
- ◯ End of study (study completed)
- ◯ death (participant)
- ◯ Participant refused further participation
- ◯ Participant unable to adhere to visit schedule
- ◯ Participant relocated, no follow-up planned
- ◯ Investigator decision
- ◯ unable to contact the participant
- ◯ Participant not eligible for enrollment
- ◯ Invalid ID due to duplicate screening/enrollment
- ◯ Other
- ◯ Early study closure
- ◯ End of study (adverse outcome)

Specify refusal reason/ Investigator reason ______________________

Other, Specify ______________________

....

General Comments ______________________

Ad-Hoc

Staff name

____

Please capture date of visit

____

Please summarize the purpose of the visit

Safety Protocol

Today's date	__________________
Time	__________________
Staff	__________________
Safety Protocol Issue	◯ Social Harm<br>◯ Protocol Violation<br>◯ Unanticipated Problem
Date Reported	__________________
Notes	______________________

STI Data Qc And Filing

SECTION A: STAFF DETAILS

Staff Member Name

____

Date

____

SECTION B: QUALITY ASSURANCE

Forms Received from the Field

- ☐ BQ Consent form
- ☐ Study Note
- ☐ Proof of Reimbursement
- ☐ Enrolment Log
- ☐ Expert Baseline: CT/NG
- ☐ Expert Baseline: TV
- ☐ Expert Postnatal: CT/NG
- ☐ Expert Postnatal: TV
- ☐ NHLS: CD4 Count
- ☐ NHLS: Syphilis Test
- ☐ NHLS: Viral Load
- ☐ NHLS: Baby HIV PCR
- ☐ OTHER

(Select all that you received)

Other - Specify the other form(s) received

____

QUALITY ASSURANCE: Phase 2A

Get all the participant's enrolment source documents and perform a comprehensive QC on all the source documents. After the QC is done, mark each document as "checked, properly completed" if you have no query opened on the source document.

IN CASES WHERE A QUERY IS OPENED. PLEASE CONTACT THE RESPONSIBLE DATA COLLECTOR IMMEDIATELY!

Please note that you also accept receipt of all source documents by checking them below.

	Checked, Properly Completed	Not completed, Returned to the RA
Consent Form	○	○
Study Note	○	○
Proof of Reimbursement	○	○
Enrolment Log	○	○
Expert Baseline: CT/NG	○	○

Expert Baseline: TV	○	○
Expert Postnatal: CT/NG	○	○
Expert Postnatal: TV	○	○
NHLS: CD4 Count	○	○
NHLS: Syphilis Test	○	○
NHLS: Viral Load	○	○
NHLS: Baby HIV PCR	○	○
[forms_received_oth]	○	○

Skip

Electronic Data QC

You are supposed to go through each electronic data tool and ensure the following:

1. Each Tracking Field has a data point.
2. The data is consistent
3. The data is verified with source documents

After doing the above inspection. You marked the forms as complete and locked the form.

Once all the forms are checked and properly completed.

	Checked and Completed Properly	Query Opened
1, Baseline: Screening and Enrolment	○	○
2, Baseline: Baseline data	○	○
3, STI: Physical Exam	○	○
4, STI: Specimen and Randomization	○	○
5, STI: Scheduling	○	○
6, STI: TOC Visit Activities	○	○
7, STI: Calling Reminder_2	○	○
8,STI: Scheduling post Delivery activities	○	○
9, Birth Register data	○	○
10, Post Natal Visit Activities	○	○
11, 6 week Immunization Visit Activities	○	○

.....

Date of QC

____

COMMENTS

Comments

____

SAVING INSTRUCTION

MARK THIS FORM AS COMPLET ONCE VERIFIED AND LOCK IT.

SELECT SAVE AND EXIT FORM.

Proceed to QC other source documents.

Gigi RMS, *et al. BMJ Open* 2023; 13:e081562. doi: 10.1136/bmjopen-2023-081562

Scheduling 2

Scheduling of Dates Associated with [randomization].

NOTE
You are about to schedule dates associated with [randomization] participants.

Please select "Proceed".

◯ Proceed

Scheduling of Dates Associated

NOTE:
You are about to schedule dates associated with microbiome participants.

Please select "proceed"

◯ Proceed

Scheduling Dates for 3-Week ToC

NOTE: The participant tested positive and therefore we need to schedule a date, exactly 3-weeks from today to conduct a test-of-cure.

Scheduling the 3-week ToC

NOTE:
Please schedule a date, 3 weeks from today treatment given. Please use the calculator assistance to ensure that you schedule a date exactly 21 days from today.

____

Calculator Assist

The number here must be equal to 21

____

ERROR
The field does not equal to 21, please change it

Have you handed the TOC date to the participant?

◯ Yes
◯ No

Scheduling Dates Associated with ToC Reminder

Schedule date for REMINDER of 3-week ToC visit

____

Calculator Assist for scheduling ToC reminder date

The reminder phone call will be made 18 days following the treatment date. The number of days need to equal to 18.

____

ERROR
You did not enter the date correctly. The number should equal to 18. Please redo the date.

Scheduling Dates Associated with 3-Week ToC Missed Visit Date

NOTE:
You have successfully scheduled the reminder date.

Please select "proceed" to schedule the missed visit date for the 3 week ToC visit.

○ Proceed

Schedule the date for the MISSED VISIT of the ToC visit.

This date should be 3 weeks after the date on which the participant received their test result.

____

Calculator Assist for scheduling 3-week ToC Missed Visit

The participant's time period allowed for attending a ToC will start 3 weeks after they received their result and will close 3 weeks after the date they received their result.

The number here must show 35

____

ERROR
You did not enter the date correctly. The number should equal to 35. Please redo the date.

NOTE:
You have successfully scheduled the 3-week ToC close date

Please select "proceed" to start scheduling the next visit dates

○ Proceed

Dates Associated with reminder for the 28 Week call

NOTE:
You are about to schedule dates for the call reminder at 28 weeks.

Please select "Proceed".

◯ Proceed

Note:
Schedule the date for the 28 week call. We will contact each participant to ask the date for their 30 weeks clinic visit is.

____

Calculation assist for scheduling the 32-week reminder date.

This number must equal to 196

____

Days to call reminder

____

ERROR
The number you have entered does not match 196. Please select a different date so that the number equals to 196.

Scheduling the Dates Asscoiated with the 32 Week Gestational Visit

NOTE
You are about the start scheduling dates associated with the 32 week visit. You will need to schedule the following associated dates:

1. Week 32 date
2. Week 32 reminder date
3. Week 32 missed visit date

Select "Proceed" to start scheduling

◯ Proceed

Schedule the date for the 32 week gestational age, visit

Note to RA: please make sure that this date does not fall on Friday, weekend, and public holidays.

____

Days Difference (the difference between 32 weeks & Gestational age)

____

Gigi RMS, *et al. BMJ Open* 2023; 13:e081562. doi: 10.1136/bmjopen-2023-081562

Calculate assist for 32 week visit

The number here must equal to [gest_week_calc]

____

Match

____

The date you have entered does not meet the 93 day criteria. Does the intended or original date fall on a Friday weekend or public holiday?

◯ Yes
◯ No

ERROR
The numbers you have entered does not match. Please select a different date so that the numbers match.

Dates Associated with reminder for the 32 Week Gestational Age Visit

NOTE:
You have successfully scheduled the 32 week date.

We will need to contact the participant at least 7 days before the scheduled visit to remind them.

Select "Proceed" to schedule the reminder date for the 32 week visit.

◯ Proceed

Note:
Schedule the date for the 32 week reminder. We will contact each participant starting 7 days prior to their 32-week gestation date. That means the date scheduled here should be 7 days earlier then the scheduled date for the 32-week visit.

____

Calculation assist for scheduling the 32-week reminder date.

This number must equal to 7

____

ERROR
The number you have entered does not match 7. Please select a different date so that the number equals to 7

Dates associated with the 32 week gestational age missed visit

NOTE:
You have successfully scheduled the 32 week reminder date.

Select "Proceed" to schedule the 32 week missed visit date.

◯ Proceed

Schedule the date for the 32 week missed visit date.

Note: Participants will have 3 weeks (21 days) to present for their 32 week visit date after which the visit will be closed out.

____

Calculation Assist for scheduling the 32-Week missed visit date.

This number must equal to 21

____

ERROR
The number you have entered does not match 21. Please select a different date so that the number equals to 21

Estimated Delivery Date

You are about the schedule the Estimated Delivery Date.

Please select "proceed"

◯ Proceed

Estimated Delivery Date

____

Days difference between estimated date of delivery and gestational age

____

Gigi RMS, *et al. BMJ Open* 2023; 13:e081562. doi: 10.1136/bmjopen-2023-081562

Calculation Assist for scheduling the Estimated Date for Delivery date.

This number must equal to [edod_calc]

____

Match ______________________________

ERROR
The number you have entered does not match. Please select a different date so that the numbers match

You have completed all the scheduling dates.

Please check that all dates entered comply with the "calculation assistance". Once this has been done you can select "Complete" and "Save & Exit"

NOTES

Notes box

Data Quality

Name	______________________
Time	______________________

Gigi RMS, et al. BMJ Open 2023; 13:e081562. doi: 10.1136/bmjopen-2023-081562

Updated Estimated Delivery Date

Please specify the updated delivery date

____

Gigi RMS, *et al. BMJ Open* 2023; 13:e081562. doi: 10.1136/bmjopen-2023-081562
