# Supplementary material for: Genital tract infections, the vaginal microbiome and gestational age at birth among pregnant women in South Africa: a cohort study protocol
Source: BMJ Open. 2023 Dec 28;13(12):e081562. doi: 10.1136/bmjopen-2023-081562 (PMC10759125; doi:10.1136/bmjopen-2023-081562)
Supplement: Author's manuscript [file bmjopen-2023-081562.draft_revisions.pdf]

BMJ Open is committed to open peer review. As part of this commitment we make the peer review history of every article we publish publicly available.

When an article is published we post the peer reviewers' comments and the authors' responses online. We also post the versions of the paper that were used during peer review. These are the versions that the peer review comments apply to.

The versions of the paper that follow are the versions that were submitted during the peer review process. They are not the versions of record or the final published versions. They should not be cited or distributed as the published version of this manuscript.

BMJ Open is an open access journal and the full, final, typeset and author-corrected version of record of the manuscript is available on our site with no access controls, subscription charges or pay-per-view fees (http://bmjopen.bmj.com).

If you have any questions on BMJ Open's open peer review process please email info.bmjopen@bmj.com

BMJ Open

Genital tract infections, the vaginal microbiome and gestational age at birth among pregnant women in South Africa: a cohort study protocol

Journal:	*BMJ Open*
Manuscript ID	bmjopen-2023-081562
Article Type:	Protocol
Date Submitted by the Author:	31-Oct-2023

Complete List of Authors:	Gigi, Ranjana ; University of Bern, Institute of Social and Preventive Medicine; Foundation for Professional Development, Research Unit<br>Mdingi, Mandisa ; Foundation for Professional Development, Research Unit<br>Jung, Hyunsul; University of Pretoria, Medical Microbiology<br>Claassen-Weitz, Shantelle; University of Cape Town, Department of Pathology<br>Bütikofer, Lukas; University of Bern, CTU Bern, Department of Clinical Research<br>Klausner, Jeffrey D.; University of Southern California, Department of Population and Public Health Sciences, Keck School of Medicine<br>Muzny, Christina; University of Alabama at Birmingham, Medicine/ID<br>Taylor, Christopher; Louisiana State University Health Sciences Center, Department of Microbiology, Immunology, and Parasitology<br>van de Wijgert, Janneke H.H.M.; University Medical Centre Utrecht, Julius Center for Health Sciences and Primary Care<br>Peters, Remco; Foundation for Professional Development, Research Unit; University of Pretoria, Department of Medical Microbiology<br>Low, Nicola; University of Bern, Institute of Social and Preventive Medicine

Keywords:	EPIDEMIOLOGIC STUDIES, Sexually Transmitted Disease, Diagnostic microbiology < INFECTIOUS DISEASES, Maternal medicine < OBSTETRICS, Follow-Up Studies

SCHOLARONE™
Manuscripts

Genital tract infections, the vaginal microbiome and gestational age at birth among pregnant women in South Africa: a cohort study protocol

Ranjana M S Gigi,[1 2] Mandisa M Mdingi,[2] Hyunsul Jung,[3] Shantelle Claassen Weitz,[4] Lukas Bütikofer,[5] Jeffrey D Klausner,[6] Christina A Muzny,[7] Christopher M Taylor,[8] Janneke H H M van de Wijgert*,[9] Remco P H Peters*,[2 3 4] Nicola Low*[1]

1) Institute of Social and Preventive Medicine, University of Bern, Bern, Switzerland

2) Research Unit, Foundation for Professional Development, East London, South Africa

3) Department of Medical Microbiology, University of Pretoria, Pretoria, South Africa

4) Department of Pathology, University of Cape Town, Cape Town, South Africa

5) CTU Bern, Department of Clinical Research, University of Bern, Bern, Switzerland

6) Keck School of Medicine, University of Southern California, Los Angeles, USA

7) Division of Infectious Diseases, University of Alabama at Birmingham, Birmingham, USA

8) School of Medicine, Louisiana State University, USA

9) Julius Center for Health Sciences and Primary Care, University Medical Center Utrecht, Utrecht University, Utrecht, Netherlands

Correspondence: Prof. Nicola Low (nicola.low@unibe.ch)

*Contributed equally

Word counts: Abstract, 294; main text, 3736; 2 figures, 1 supplemental online file

Keywords

Pregnancy complications, premature birth, reproductive tract infections, sexually transmitted infections, microbiota, South Africa

For peer review only - http://bmjopen.bmj.com/site/about/guidelines.xhtml

Abstract

Introduction Preterm birth complications are the most common cause of death in children under 5 years. The presence of multiple microorganisms and genital tract inflammation could be the common mechanism driving early onset of labour. South Africa has high levels of preterm birth, genital tract infections and HIV infection among pregnant women. We plan to investigate associations between the presence of multiple lower genital tract microorganisms in pregnancy and gestational age at birth.

Methods and analysis This cohort study enrols around 600 pregnant women at one public health care facility in East London, South Africa. Eligible women are ≥18 years and at <27 weeks of gestation, confirmed by ultrasound. At enrolment and 30-34 weeks of pregnancy, participants receive on-site tests for *Chlamydia trachomatis* and *Neisseria gonorrhoeae*, with treatment if test results are positive. At these visits, additional vaginal specimens are taken for: PCR detection and quantification of *Trichomonas vaginalis*, *Candida* species, *Mycoplasma genitalium, M. hominis*, *Ureaplasma urealyticum* and *U. parvum*; microscopy and Nugent scoring; and for 16S ribosomal ribonucleic acid gene sequencing and quantification. Pregnancy outcomes are collected from a post-natal visit and birth registers. The primary outcome is gestational age at birth. Statistical analyses will explore associations between specific microorganisms and gestational age at birth. To explore the association with the quantity of microorganisms, we will construct an index of microorganism load and use mixed effects regression models and classification and regression tree analysis to examine which combinations of microorganisms contribute to earlier gestational age at birth.

Ethics and dissemination This protocol has approvals from the University of Cape Town Research Ethics Committee and the Canton of Bern Ethics Committee. Results from this study will be uploaded to preprint servers, submitted to open access peer-reviewed journals and presented at regional and international conferences.

Registration to be registered on clinicaltrials.gov

For peer review only

For peer review only - http://bmjopen.bmj.com/site/about/guidelines.xhtml

Article summary, strengths and limitations of the study

- This cohort study takes a holistic approach, investigating both the presence and quantity of multiple lower genital tract microorganisms, including vaginal microbiota, in pregnancy and their associations with gestational age at birth.
- The study is set in a location where the prevalence of genital tract infections and adverse pregnancy outcomes are high, uses ultrasound scans to assess gestational age at enrolment accurately, and state-of-the-art molecular diagnostic methods.
- The study setting is limited to one research site, which may affect the generalisability of the findings.
- The use of gestational age at birth as a continuous outcome, instead of preterm birth as a dichotomous outcome, might limit comparability with other studies, but we will also examine the binary outcome preterm birth in secondary analyses.

For peer review only

For peer review only - http://bmjopen.bmj.com/site/about/guidelines.xhtml

Introduction

Preterm birth complications are the most common cause of death in children under 5 years.[1] Close to one million infants die every year because they are born preterm (before 37 completed weeks of gestation), mainly from infectious, respiratory and neurological complications, and those that survive can experience long-term morbidity.[1, 2] South Africa has a high incidence of preterm birth at around 10%,[3] around 30% of women have one or more curable sexually transmitted infections during pregnancy[4, 5] and about 30% of pregnant women are living with HIV.[6]

Microbial colonisation or infection during pregnancy, in the lower or upper genital tract, have been reported to predispose to preterm birth, as do anatomical, biochemical, endocrinological, immunological, nutritional, environmental and psychosocial factors.[7, 8] The presence of microorganisms may contribute to early onset of labour directly, through presumed ascension from the lower to the upper genital tract, or indirectly, through a pathway of inflammatory response, or a combination of both.[7, 9] Inflammation may be the common pathway, even if infection has not reached the amniotic cavity.[10]

Much of the research reporting on the role of sexually transmitted infections in pregnancy and preterm birth has focused on single infections, such as *Chlamydia trachomatis,*[11] *Neisseria gonorrhoeae,*[12] and *Trichomonas vaginalis.*[13] *Mycoplasma genitalium* is the most recently recognised sexually transmitted infection and, whilst an association with preterm birth has been reported, there are few studies with prospective data collection.[14] Bacterial vaginosis is the most common vaginal microbiota dysbiosis and is associated with adverse pregnancy outcomes, either alone, or in combination with other sexually transmitted infections.[15-17] Associations with adverse birth outcomes have also been observed for other genital mycoplasmas, *M. hominis, Ureaplasma urealyticum* and *U. parvum*.[18] For individual sexually transmitted infections, bacterial vaginosis and colonisation by other genital mycoplasmas, summary odds ratios for the association with adverse birth outcomes in meta-analyses of univariable data are generally around 1.3 to 2.0.[11-14, 16, 18] *Candida* spp. have not been found to be associated with preterm birth, but an association with more inflammatory, symptomatic yeast infection cannot be ruled out.[19] Most studies about these

For peer review only - http://bmjopen.bmj.com/site/about/guidelines.xhtml

microorganisms do not present analyses that examine the role of co-occurrence or control for confounding factors, so the presence or strength of the causal association cannot be assessed.[20] It is also important to include women living with HIV, amongst whom there are fewer studies about associations between genital tract infections and adverse birth outcomes than amongst women without HIV infection.[21, 22]

The importance of the quantity of different microorganisms as a driver of preterm birth has not been extensively studied,[23-25] but might be as, or more, relevant than their presence.[23] Together with inflammation or immune activation in the genital tract during pregnancy, organism load could be an important driver of the early onset of labour and preterm birth.[8, 23, 24, 26, 27] This calls for a holistic approach to research studies, which combines information about the presence of different microorganisms, the quantified load and the microbiome, sociodemographic factors and HIV amongst women living with infection, most of whom are receiving antiretroviral therapy. The overall aim of this study is to investigate associations between the presence of lower genital tract microorganisms in pregnancy and preterm birth and other adverse pregnancy outcomes. This will be achieved through three objectives to explore: (1) the association between the presence of specific lower genital tract microorganisms and gestational age at birth (primary outcome), as well as secondary adverse pregnancy outcomes; (2) the association between quantified load of vaginal and sexually transmitted microorganisms and gestational age at birth (primary outcome) as well as secondary adverse pregnancy outcomes; and (3) the combinations of microorganisms that are most strongly associated with earlier gestational age at birth.

Methods and analysis

STUDY DESIGN AND SETTING

This prospective closed cohort study follows women enrolled during pregnancy until after they give birth (Figure 1). The study is conducted at the antenatal clinic of one primary health care facility in Buffalo City Metropolitan Municipality, Eastern Cape Province, South Africa. This cohort study is part of a larger project, called Philani Ndiphile (meaning ‘be healthy and I will be healthy’ in isiXhosa), which includes a randomised implementation-

For peer review only - http://bmjopen.bmj.com/site/about/guidelines.xhtml

effectiveness trial of screening strategies for sexually transmitted infections in pregnancy[28] and a case-control study about persistent *C. trachomatis* infection.

PARTICIPANTS

Inclusion criteria: Pregnant women aged 18 years or older, who live in Buffalo City Metropolitan Municipality, intend to deliver in the same municipality and provide written informed consent to take part in the study. The eligible gestational age at enrolment, confirmed by ultrasound, was below 20 weeks at the start of the study in March 2021 and was increased to 27 weeks in September 2021 to increase enrolment and to align with another trial.[29]

Exclusion criteria: Participation in any other research study or inability to understand and speak a local language (English, Afrikaans, or isiXhosa).

Figure 1 Study visits and specimen collection

Abbreviations: CT, *Chlamydia trachomatis*; NG, *Neisseria gonorrhoeae*

ENROLMENT

A trained study field worker approaches all pregnant women attending an antenatal care visit at the clinic and individually informs them about the study. If a potential participant shows interest in the study, the study field worker checks for eligibility. The date of the last menstrual period is used initially to estimate gestational age. If all eligibility criteria are met, a study field worker obtains written informed consent from the participant.

STUDY PROCEDURES AND VISITS

At the enrolment visit, study field workers administer a questionnaire to record socio-demographic, behavioural and clinical information in an online Research Electronic Data Capture software (REDCap)[30] database. The study nurse examines the woman, according to the South African government standard of care.[31] As an additional procedure, a study nurse with training in obstetric ultrasound performs an abdominal ultrasound to estimate the gestational age. If this is later than the eligibility criterion, the participant is excluded from any further study activity. A study nurse collects vaginal samples (Figure 1) for on-site

For peer review only - http://bmjopen.bmj.com/site/about/guidelines.xhtml

testing for *C. trachomatis* and *N. gonorrhoeae* using the Xpert CT/NG assay on the Gene Xpert platform (Cepheid, Sunnyvale, CA, USA) and for further off-site laboratory testing (see 'Specimen collection and analysis').

If the test result for *C. trachomatis* or *N. gonorrhoeae* is positive, the woman receives immediate antibiotic treatment if still on site or is contacted by telephone and asked to return to the clinic for treatment. Antibiotic treatments are first-line regimens according to South African guidelines: for *C. trachomatis*, 1g oral azithromycin and for *N. gonorrhoeae*, 500mg intramuscular ceftriaxone (250mg until South African treatment guidelines for sexually transmitted infections changed in December 2022).[32] Women with vaginal discharge syndrome but with negative Xpert test results for *C. trachomatis* and *N. gonorrhoeae* receive empirical treatment for trichomoniasis with metronidazole 400 mg twice a day for 7 days. The study nurse gives advice to women with *C. trachomatis* or *N. gonorrhoeae* on safe disclosure of her diagnosis to her partner(s) and gives her a notification slip(s) to request her partner(s) to attend a clinic for treatment.

A follow-up visit at 30-34 weeks (third trimester visit) is scheduled at which clinical and obstetric information, as well as the same vaginal specimens, are collected and treatment given, if indicated.

A post-natal visit is scheduled for 3-6 days after giving birth, according to the South African government standard.[31] A study nurse collects information about the birth outcome and perinatal period through a questionnaire with the mother, a patient-held medical record of the baby (the Road to Health card) and/or the birth register from the public birth clinics within the study area. If the participant does not attend the post-natal visit, study staff telephone her to ask her to return to the clinic. If the participant is not able to return to the clinic, the study physician collects the information by telephone or from the birth register.

OUTCOMES

The primary outcome is gestational age at birth, measured in days, based on the ultrasound assessment at the enrolment visit. Secondary outcomes are preterm birth (<37 completed weeks of gestation), low birth weight (birth weight <2500g), miscarriage (dead foetus delivered before 28 completed weeks of pregnancy or with birth weight below 1000g) and

For peer review only - http://bmjopen.bmj.com/site/about/guidelines.xhtml

stillbirth (dead foetus delivered at or after 28 completed weeks of pregnancy or with birth weight above 1000g).[33, 34] We chose gestational age at birth as the primary outcome because, whilst the cut-off of 37 weeks is the standard definition of preterm birth, dichotomisation of a continuous variable results in a loss of statistical power.[35]

SPECIMEN COLLECTION AND ANALYSIS

DATA SOURCES AND VARIABLES

The source data are case report forms recording questionnaire data for the enrolment, third trimester and post-natal visits and forms for laboratory and specimen results, which are stored in REDCap, a secure web-based database[30] (online supplemental file), hosted by the Foundation for Professional Development, Pretoria, South Africa.

SPECIMEN COLLECTION

At the enrolment and the third trimester visits, a study nurse collects two vaginal smears using inoculation loops and air-dries them on glass slides. She then collects vaginal specimens by inserting swabs into the vagina up to a mark at 4 cm and rotating around the vaginal wall. Five swabs are collected in the following order: one Cepheid GeneXpert Xpert Vaginal/Endocervical Swab in a tube with Xpert Swab Transport Reagent (Cepheid, Sunnyvale, CA, USA); two Qiagen digene Female Swabs in a single tube with digene Specimen Transport Medium (Qiagen, Hilden, Germany); and two dry FLOQswabs (COPAN, Brescia, Italy) each in a separate sterile tube (Figure 1).

TRANSPORT AND STORAGE OF SPECIMENS

Vaginal smear glass slides are stored and transported in plastic slide carriers at room temperature. All vaginal swabs are initially stored at the clinic in a refrigerator (2-8°C with daily temperature checks). All vaginal swabs, except the Xpert swab, which is tested on-site, are transported on ice packs once a week by overnight road courier, to the laboratory at the Department of Medical Microbiology, University of Pretoria, where they are also stored in a refrigerator until DNA extraction.

For peer review only - http://bmjopen.bmj.com/site/about/guidelines.xhtml

MICROBIOLOGICAL ANALYSES

The Xpert vaginal swabs are tested on-site using the Xpert CT/NG assay (Cepheid, Sunnyvale, CA, USA) to detect *C. trachomatis* and *N. gonorrhoeae*, as per manufacturer's instructions. At the University of Pretoria, air-dried vaginal smears are heat-fixed and Gram-stained.[36] Two qualified people record the Nugent scores (0-3: normal; 4-6: intermediate; 7-10: bacterial vaginosis) and the presence of yeasts.[37] In case of discrepancies a third person assesses the slide and consensus is reached by discussion. At the University of Pretoria, one vaginal FLOQswab is used for PCRs. The genomic DNA is extracted using the High Pure PCR Template Preparation Kit (Roche Diagnostics GmbH, Mannheim, Germany) as per manufacturer's instructions. Real-time PCR assays are then performed using the LightCycler 480 Probes Master Kit (Roche Diagnostics GmbH, Mannheim, Germany) on the LightCycler 480 II instrument (Roche Diagnostics GmbH, Mannheim, Germany). Previously published primer and hydrolysis probe sequences and cycling conditions are used for detection and quantification of *M. genitalium*,[38] *M. hominis*,[39] *U. parvum*,[40] *U. Urealyticum,*[40] *T. vaginalis*[41] and *Candida* spp.[42, 43] The load for each assayed microorganism detected in vaginal swab specimens by real-time PCR or GeneXpert is obtained from the cycle threshold value.

VAGINAL MICROBIOME LABORATORY ANALYSES

The vaginal swabs stored in Qiagen digene Specimen Transport Medium will be used for DNA extraction and subsequent 16S ribosomal ribonucleic acid (rRNA) amplicon sequencing targeting the V3-V4 hypervariable regions for vaginal microbiota analyses at the Division of Medical Microbiology, University of Cape Town.

A commercial DNA extraction kit will be used and a bead-beating step included.[44] A DNA isolation control will be prepared from an unused vaginal swab specimen during this process. Two PCR rounds will be conducted to prepare amplicon libraries.[45] The aim of the first PCR round is to amplify 16S rRNA gene V3-V4 regions, using the 319F 5'-ACTCCTACGGGAGGCAGCAG-3' forward primer and 806R 5'-GGACTACHVGGGTWTCTAAT-3' reverse primer. The aim of the second PCR round is to barcode the V3-V4 amplicons by a dual-index approach, permitting multiplexing of up to 384 samples (including controls). Amplicon concentrations for all sample libraries are measured and normalised to form a mixed loading library. The libraries will be sequenced on an Illumina MiSeq instrument

For peer review only - http://bmjopen.bmj.com/site/about/guidelines.xhtml

(Illumina, San Diego, CA, USA), 2x300bp. To quantify the number of 16S rDNA copies per swab, a quantitative PCR using the same forward and reverse primers as described above will be used. Samples from enrolment and third trimester visits from the same woman will be processed in the same run.

VAGINAL MICROBIOTA BIOINFORMATICS

Raw sequencing reads will be processed using an established bioinformatics pipeline.[46] Taxonomic assignment of amplicon sequence variants (ASVs) will be done in DADA2[47] with SILVA[48] as the reference database. Vaginal microbiome composition data will be visualised in heatmaps and diagrams. For each vaginal sample, we will calculate diversity measures (alpha diversity), relative abundances and estimated concentrations of key vaginal bacteria and bacterial groups, as described.[46] We will use the entire sequencing dataset to design vaginal microbiota types by hierarchical clustering, and each sample will be assigned to one vaginal microbiota type.

SAMPLE SIZE CALCULATION

The sample size has been calculated for objective 1, with a univariable comparison between the presence of a genital tract microorganism in the mother and gestational age at birth. Figure 2 shows that, for any vaginal or sexually transmitted microorganism, or vaginal microbiota type that has a prevalence of 10% or more among all enrolled women, about 500-600 patients provides adequate power (80%) to detect a one-week difference (with standard deviation 2) in mean gestational age between the two groups using Student's t-test. Specifying an alpha of 0.83% allows for multiple hypothesis testing (6 hypotheses, using a Bonferroni correction). We enrol around 600 women and aim to have complete follow-up and outcome data on at least 550 women.

Figure 2 Sample size requirements at different levels of exposure prevalence with power of 80% and alpha 0.83% based on Student's t-test.

Legend: panel A, standard deviation 1.5; panel B, standard deviation 2.0. The curves for % exposed are symmetrical around a prevalence of 50%, i.e., curve for 10% exposed is same as that for 90% exposed.

For peer review only

STUDY TIMELINE

Enrolment began on 28 March 2021, with an estimated date for reaching the target sample size in August 2023. Follow-up of all participants until the post-natal visit is expected to be completed by March 2024.

STATISTICAL ANALYSIS

This description gives an overview of the statistical methods for each objective. A detailed statistical analysis plan will be published separately and made publicly available.

We will describe the numbers of women enrolled and available at each follow-up visit in a flow chart. We will present descriptive tables of socio-demographic, behavioural and clinical characteristics and compare women with complete follow-up with those lost to follow-up.

OBJECTIVE 1) ASSOCIATION BETWEEN SPECIFIED EXPOSURES AND PREGNANCY OUTCOMES

1a. We will examine a primary set of microorganisms as exposures, detected at either enrolment or at the third trimester visit: *M. genitalium, M. hominis*, *U. urealyticum, U. parvum, T. vaginalis* and *Candida* spp. are the microorganisms for which women did not receive diagnostic tests and treatment during study visits. We will use the mean and standard deviation for the continuous outcome (gestational age) and absolute and relative frequencies for the binary outcomes (all secondary outcomes). Gestational age at birth for each exposure will be compared using Student's t-test and a mean difference with 95% confidence intervals (continuous outcome) and Fisher's exact test and a risk difference with 95% confidence intervals (binary outcomes).

To control for confounding, multivariable regression models will be fitted to all outcomes with a set of pre-specified potential confounders (age, educational level, alcohol consumption, HIV infection, prior preterm birth) for all organisms. For analyses of *M. genitalium*, *T. vaginalis*, and *Candida* spp., we will also control for bacterial vaginosis (Nugent score 7-10). The other genital mycoplasmas can be identified from 16S rRNA amplicon sequencing in women with vaginal dysbiosis, so are sometimes considered part of bacterial vaginosis. For these organisms, we will conduct descriptive analyses, stratifying by the presence of bacterial vaginosis. For continuous confounders a linear relationship will be

For peer review only - http://bmjopen.bmj.com/site/about/guidelines.xhtml

For peer review only

assumed by default but transformations (e.g., log) or more flexible approaches (e.g., splines or fractional polynomials) will be considered if there is evidence for non-linearity. For the continuous outcome we will use linear mixed effects regression models (including data from either visit and the participant as random effect) and report the result as mean difference with 95% confidence intervals. For the binary outcome we will use logistic mixed-effects regression and report the result as odds ratios with 95% confidence intervals.

1b. Comparisons for associations with timing of detection, other microorganism exposures and birth outcomes will be considered secondary analyses. Associations between vaginal microbiota composition and pregnancy outcomes will be assessed. We will use compositional multivariable analysis methods to identify bacterial taxa that are differentially abundant between binary pregnancy outcome groups at the level of individual taxon relative abundances. We will use mixed effects models (with the individual participant as the random effect and including data from both visits) to assess associations between continuous and binary pregnancy outcome and the following fixed effects derived from the vaginal microbiota data: alpha diversity, vaginal microbiota types and absolute abundances of predefined bacterial groups.[46] These models will be adjusted for confounding as described in the previous paragraph.

OBJECTIVE 2) ASSOCIATION BETWEEN QUANTIFIED MICROORGANISM LOAD AND PREGNANCY OUTCOMES

We will investigate the hypothesis that the quantity of microorganisms with inflammatory potential is associated with gestational age at birth. For this, we will analyse the vaginal microbiota data jointly with sexually transmitted infections and *Candida* spp. diagnostic test results during pregnancy (these will be considered as additional covariates in the above-mentioned regression models). We will develop a 'vaginal inflammation index', based on quantification of the vaginal microbiota and their inflammatory potential[49] and of yeasts. This vaginal inflammation index will also be analysed as a fixed effect in mixed effects models with pregnancy outcomes as the outcomes; these models will not include any of the infection parameters that were used to design the index.

For peer review only - http://bmjopen.bmj.com/site/about/guidelines.xhtml

OBJECTIVE 3) CLASSIFICATION AND REGRESSION TREE ANALYSIS FOR THE PRIMARY OUTCOME

We will conduct exploratory analyses to examine the combination of microorganisms that best predicts earlier gestational age at birth using classification and regression tree analysis.[50] This method belongs to the family of decision tree machine learning algorithms and allow for nonparametric analyses of a large number of binary, categorical or continuous predictors. They are typically easy to interpret and can detect predictors with small marginal effects when there are strong interaction effects. We will make use of the predictive potential for gestational age at birth of all sexually transmitted and genital tract microorganisms, including individual bacterial taxa or bacterial groups identified by 16S rRNA gene amplicon sequencing (as binary or continuous variables) and confounding variables identified in objective 1. We will present variable importance scores and curves of marginal effects to show how prediction of the outcome changes at different levels of the exposure of each variable in the model. To avoid overfitting, we will consider bootstrap aggregating via random forests.[51]

DATA MANAGEMENT AND CONFIDENTIALITY

DATA MANAGEMENT

Each potential participant screened for eligibility is assigned a unique participant identification number, which does not include any personal identifying information. Personal identification numbers are used to link records, specimens and laboratory test results of the participants. Data are stored in a REDCap database,[30] which is only accessible to authorised project staff. Paper records are kept in lockable fire-resistant filing cabinets. Laboratory records and journals are kept at the University of Pretoria and University of Cape Town. Forms with personal identifying information are kept separately from demographic, clinical and other data. The data manager maintains a separate, access-controlled, database that links the personal identification number with identifying information. Data quality checks are conducted by study staff onsite and data administrators at the office of the Foundation for Professional Development. All study data are stored securely at the offices of the Foundation for Professional Development in East London for up to five years after the completion of the study or as required by the institutional review board.

For peer review only

CONFIDENTIALITY

The research team is trained to adhere to guidelines on the Protection of Human Research Participants and Good Clinical Practice and fully protects the confidentiality of participants. Besides the measures described under data management, interviews are conducted in a private setting. In reports and publications, data will not be presented in a way where it could be linked to individual participants.

PATIENT AND PUBLIC INVOLVEMENT

There was no involvement of patients or the public in the development of the research questions or the study methods. The research findings will be shared through open access publications and in dissemination meetings with local stakeholders, healthcare providers and communities.

Discussion

This project is important because of its holistic approach, which considers associations between different genital tract infections, their quantity and the vaginal microbiota on earlier gestational age at birth. Many studies in this field have focused on only one or two microorganisms and few studies involve women in sub-Saharan Africa. Strengths of this study include the study setting, where the prevalence of both genital tract infections and adverse pregnancy outcomes is high, the use of ultrasound scans at enrolment for accurate assessment of gestational age and the use of state-of-the-art molecular diagnostic tests and 16S rRNA sequencing. The residual DNA from samples collected in this study will be available for future studies, including joint analyses with other studies of the influence of vaginal microbiota on adverse pregnancy outcomes.

There are limitations to the study design. First, this study involves participants from one clinic, which might limit the generalisability of the findings. Second, using gestational age at delivery as a continuous outcome instead of preterm birth as a dichotomous outcome, might limit comparability with other studies. We will, however, examine the binary outcome preterm birth in secondary analyses. Third, the vaginal samples are taken in a fixed

For peer review only - http://bmjopen.bmj.com/site/about/guidelines.xhtml

sequence at each visit, which might reduce the microorganism load of later samples. Fourth, the development of the vaginal inflammation index will use information about the inflammatory potential of microorganisms,[49] rather than direct concentrations of inflammatory markers.

This study has the potential to generate new evidence about the role of different microorganisms in earlier gestational age at birth through analyses of the presence and quantity of individual and combinations of microorganisms, relative abundance of bacterial genera and microbiota on gestational age at birth. This study will generate new hypotheses, which can be investigated in future studies.

Ethics and dissemination

This protocol and the informed consent forms are approved by the University of Cape Town, Research Ethics Committee (Reference: 676/2019), which includes activities at the University of Southern California, University of Alabama at Birmingham and Louisiana State University. Authorisation to analyse de-identified data at the University of Bern has been granted by the Canton of Bern Ethics Committee (Reference: 2021-01209). Results from this study will be submitted to regional and international conferences and to open access peer-reviewed journals and preprint servers.

Data statement

The research team will prepare datasets used in analyses, in accordance with data sharing requirements of open access journals in which manuscripts are published and in compliance with local Protection of Personal Information Act requirements. These data files will be archived with codebooks as .csv documents or R data sets and stored in REDCap. The final data files will not contain any personal identifying information of participants.

Author contributions

For peer review only - http://bmjopen.bmj.com/site/about/guidelines.xhtml

RG, NL, JW, and RP conceived and designed the study. JK supported the study through design of the parent study of the Philani Ndiphile project. RG, NL, JW, RP, HJ, CT, CM, SC and LB contributed to the data analysis plan. RG, NL, RP, MM and HJ were involved with the implementation and management of the study. RG and MM managed the data acquisition. RG, NL, JW, RP drafted the manuscript and all authors revised it. NL, JW, RP and JK supervised the study. All authors read and approved the final manuscript.

Funding statement

This work is supported by an MD-PhD scholarship from the Swiss National Science Foundation (grant number 191225), the Swiss National Science Foundation (grant number 197831) and the US National Institutes of Health (grant number R01AI149339).

Competing interests statement

LB, SC, RG, HJ, JK, NL, MM, RP, CT, JW: no competing interests to declare.

CM has received research grant funding to her institution by Gilead Inc., Abbott Molecular, Visby, and Lupin Pharmaceuticals. She is a consultant to BioNTech, Cepheid, and BioFire Diagnostics. She has received honoraria for educational presentations and review activities from Scynexis, Visby, Abbott, Elsevier, UpToDate, and DynaMed Plus.

For peer review only

References

1. UN Inter-agency Group for Child Mortality Estimation. Levels & Trends in Child Mortality: Report 2019. New York: United Nations Children's Fund, 2019. https://www.unicef.org/reports/levels-and-trends-child-mortality-report-2019 (accessed 24 October 2023).

2. Saigal S, Doyle LW. An overview of mortality and sequelae of preterm birth from infancy to adulthood. *Lancet* 2008;371:261-9. doi: 10.1016/S0140-6736(08)60136-1

3. World Health Organization. Born too soon: decade of action on preterm birth. Geneva: World Health Organization, 2023. https://www.who.int/publications/i/item/9789240073890 (accessed 24 October 2023).

4. Joseph Davey DL, Nyemba DC, Gomba Y, et al. Prevalence and correlates of sexually transmitted infections in pregnancy in HIV-infected and- uninfected women in Cape Town, South Africa. *PLoS One* 2019;14(7):e0218349. doi: 10.1371/journal.pone.0218349

5. Moodley D, Moodley P, Sebitloane M, et al. High prevalence and incidence of asymptomatic sexually transmitted infections during pregnancy and postdelivery in KwaZulu Natal, South Africa. *Sex Transm Dis* 2015;42(1):43-7. doi: 10.1097/OLQ.0000000000000219

6. Woldesenbet SA, Lombard C, Manda S, et al. The 2019 National Antenatal Sentinel HIV Survey (ANCHSS) Key Findings. South Africa: National Department of Health, 2021. https://www.nicd.ac.za/wp-content/uploads/2019/07/Antenatal_survey-report_24July19.pdf (accessed 24 October 2023).

7. Romero R, Espinoza J, Kusanovic JP, et al. The preterm parturition syndrome. *BJOG* 2006;113(Suppl 3):17-42. doi: 10.1111/j.1471-0528.2006.01120.x

8. Hunter PJ, Awoyemi T, Ayede AI, et al. Biological and pathological mechanisms leading to the birth of a small vulnerable newborn. *Lancet* 2023;401(10389):1720-32. doi: 10.1016/S0140-6736(23)00573-1

9. Goldenberg RL, Culhane JF, Iams JD, et al. Epidemiology and causes of preterm birth. *Lancet* 2008;371:75-84. doi: 10.1016/S0140-6736(08)60074-4

10. MacIntyre DA, Sykes L, Teoh TG, et al. Prevention of preterm labour via the modulation of inflammatory pathways. *J Matern Fetal Neonatal Med* 2012;25 Suppl 1:17-20. doi: 10.3109/14767058.2012.666114

11. Tang W, Mao J, Li KT, et al. Pregnancy and fertility-related adverse outcomes associated with *Chlamydia trachomatis* infection: a global systematic review and meta-analysis. *Sex Transm Infect* 2019 doi: 10.1136/sextrans-2019-053999

For peer review only - http://bmjopen.bmj.com/site/about/guidelines.xhtml

12. Vallely LM, Egli-Gany D, Wand H, et al. Adverse pregnancy and neonatal outcomes associated with Neisseria gonorrhoeae: systematic review and meta-analysis. *Sex Transm Infect* 2021;97(2):104-11. doi: 10.1136/sextrans-2020-054653

13. Van Gerwen OT, Craig-Kuhn MC, Jones AT, et al. Trichomoniasis and adverse birth outcomes: a systematic review and meta-analysis. *BJOG* 2021;128(12):1907-15. doi: 10.1111/1471-0528.16774

14. Frenzer C, Egli-Gany D, Vallely LM, et al. Adverse pregnancy and perinatal outcomes associated with Mycoplasma genitalium: systematic review and meta-analysis. *Sex Transm Infect* 2022;98(3):222-27. doi: 10.1136/sextrans-2021-055352

15. Hillier SL, Nugent RP, Eschenbach DA, et al. Association between bacterial vaginosis and preterm delivery of a low-birth-weight infant. The Vaginal Infections and Prematurity Study Group. *N Engl J Med* 1995;333(26):1737-42. doi: 10.1056/NEJM199512283332604

16. Leitich H, Bodner-Adler B, Brunbauer M, et al. Bacterial vaginosis as a risk factor for preterm delivery: a meta-analysis. *Am J Obstet Gynecol* 2003;189(1):139-47. doi: 10.1067/mob.2003.339

17. van de Wijgert JHHM. The vaginal microbiome and sexually transmitted infections are interlinked: consequences for treatment and prevention. *PLoS Med* 2017;14(12):e1002478. doi: 10.1371/journal.pmed.1002478

18. Jonduo ME, Vallely LM, Wand H, et al. Adverse pregnancy and birth outcomes associated with Mycoplasma hominis, Ureaplasma urealyticum and Ureaplasma parvum: a systematic review and meta-analysis. *BMJ Open* 2022;12(8):e062990. doi: 10.1136/bmjopen-2022-062990

19. Gigi RMS, Buitrago-Garcia D, Taghavi K, et al. Vulvovaginal yeast infections during pregnancy and perinatal outcomes: systematic review and meta-analysis. *BMC Womens Health* 2023;23(1):116. doi: 10.1186/s12905-023-02258-7

20. Low N. *Chlamydia trachomatis* and reproductive health: what can we learn from systematic reviews of observational studies? *Sex Transm Infect* 2020;96(5):315-17. doi: 10.1136/sextrans-2019-054279

21. Moodley D, Sartorius B, Madurai S, et al. Pregnancy outcomes in association with STDs including genital HSV-2 shedding in a South African cohort study. *Sex Transm Infect* 2017;93:460-6. doi: 10.1136/sextrans-2017-053113

22. Adachi K, Klausner JD, Bristow CC, et al. Chlamydia and gonorrhea in HIV-infected pregnant women and infant HIV transmission. *Sex Transm Dis* 2015;42(10):554-65. doi: 10.1097/OLQ.0000000000000340

23. Goodfellow L, Verwijs MC, Care A, et al. Vaginal bacterial load in the second trimester is associated with early preterm birth recurrence: a nested case-control study. *BJOG* 2021;128(13):2061-72. doi: 10.1111/1471-0528.16816

For peer review only - http://bmjopen.bmj.com/site/about/guidelines.xhtml

24. Bretelle F, Rozenberg P, Pascal A, et al. High *Atopobium vaginae* and *Gardnerella vaginalis* vaginal loads are associated with preterm birth. *Clin Infect Dis* 2015;60(6):860-7. doi: 10.1093/cid/ciu966

25. Freitas AC, Bocking A, Hill JE, et al. Increased richness and diversity of the vaginal microbiota and spontaneous preterm birth. *Microbiome* 2018;6(1):117. doi: 10.1186/s40168-018-0502-8

26. Goldenberg RL, Hauth JC, Andrews WW. Intrauterine infection and preterm delivery. *N Engl J Med* 2000;342(20):1500-7. doi: 10.1056/NEJM200005183422007

27. Andrews WW, Hauth JC, Goldenberg RL, et al. Amniotic fluid interleukin-6: correlation with upper genital tract microbial colonization and gestational age in women delivered after spontaneous labor versus indicated delivery. *Am J Obstet Gynecol* 1995;173(2):606-12. doi: 10.1016/0002-9378(95)90290-2

28. Medina-Marino A, Cleary S, Muzny CA, et al. Sexually transmitted infection screening to prevent adverse birth and newborn outcomes: study protocol for a randomized-controlled hybrid-effectiveness trial. *Trials* 2022;23(1):441. doi: 10.1186/s13063-022-06400-y

29. Vallely AJ, Pomat WS, Homer C, et al. Point-of-care testing and treatment of sexually transmitted infections to improve birth outcomes in high-burden, low-income settings: Study protocol for a cluster randomized crossover trial (the WANTAIM Trial, Papua New Guinea). *Wellcome Open Res* 2019;4:53. doi: 10.12688/wellcomeopenres.15173.2

30. Harris PA, Taylor R, Thielke R, et al. Research electronic data capture (REDCap) - A metadata-driven methodology and workflow process for providing translational research informatics support. *J Biomed Inform* 2009;42(2):377-81. doi: 10.1016/j.jbi.2008.08.010

31. South African National Department of Health. Guidelines for Maternal Care in South Africa: A manual for clinics, community health centres and district hospitals. Pretoria: Department of Health, 2016. https://knowledgehub.health.gov.za/elibrary/guidelines-maternity-care-south-africa-2016 (accessed 26 October 2023).

32. Peters RPH, Garrett N, Chandiwana N, et al. Southern African HIV Clinicians Society 2022 guideline for the management of sexually transmitted infections: Moving towards best practice. *South Afr J HIV Med* 2022;23(1):1450. doi: 10.4102/sajhivmed.v23i1.1450

33. World Health Organization. ICD-10 International statistical classification of diseases and related health problems. Geneva: World Health Organization, 2016. https://icd.who.int/training/icd10training/ICD-10%20training/ICD-10_Resources/ICD-10_Volume_1.pdf (accessed 26 October 2023).

34. March of Dimes, PMNCH, Save the children, et al. Born Too Soon: The Global Action Report on Preterm Birth. Geneva: World Health Organization, 2012. https://www.who.int/publications/i/item/9789241503433 (accessed 26 October 2023).

35. Altman DG, Royston P. The cost of dichotomising continuous variables. *BMJ* 2006;332(7549):1080. doi: 10.1136/bmj.332.7549.1080

For peer review only - http://bmjopen.bmj.com/site/about/guidelines.xhtml

36. Jordan J. Laboratory Procedure Manual: Bacterial Vaginosis in Swabs. Atlanta: Center for Disease Control and Prevention, 2004. https://www.cdc.gov/nchs/data/nhanes/nhanes_03_04/l34_c_met_bv.pdf (accessed 26 October 2023).

37. Nugent RP, Krohn MA, Hillier SL. Reliability of diagnosing bacterial vaginosis is improved by a standardized method of gram stain interpretation. *J Clin Microbiol* 1991;29(2):297-301. doi: 10.1128/jcm.29.2.297-301.1991

38. Edberg A, Jurstrand M, Johansson E, et al. A comparative study of three different PCR assays for detection of Mycoplasma genitalium in urogenital specimens from men and women. *J Med Microbiol* 2008;57(Pt 3):304-09. doi: 10.1099/jmm.0.47498-0

39. Ferandon C, Peuchant O, Janis C, et al. Development of a real-time PCR targeting the yidC gene for the detection of Mycoplasma hominis and comparison with quantitative culture. *Clin Microbiol Infect* 2011;17(2):155-9. doi: 10.1111/j.1469-0691.2010.03217.x

40. Frolund M, Bjornelius E, Lidbrink P, et al. Comparison between culture and a multiplex quantitative real-time polymerase chain reaction assay detecting Ureaplasma urealyticum and U. parvum. *PLoS One* 2014;9(7):e102743. doi: 10.1371/journal.pone.0102743

41. Pillay A, Radebe F, Fehler G, et al. Comparison of a TaqMan-based real-time polymerase chain reaction with conventional tests for the detection of Trichomonas vaginalis. *Sex Transm Infect* 2007;83(2):126-9. doi: 10.1136/sti.2006.022376

42. Fujita S, Lasker BA, Lott TJ, et al. Microtitration plate enzyme immunoassay to detect PCR-amplified DNA from Candida species in blood. *J Clin Microbiol* 1995;33(4):962-7. doi: 10.1128/jcm.33.4.962-967.1995

43. Shin JH, Nolte FS, Holloway BP, et al. Rapid identification of up to three Candida species in a single reaction tube by a 5' exonuclease assay using fluorescent DNA probes. *J Clin Microbiol* 1999;37(1):165-70. doi: 10.1128/JCM.37.1.165-170.1999

44. Villette R, Autaa G, Hind S, et al. Refinement of 16S rRNA gene analysis for low biomass biospecimens. *Sci Rep* 2021;11(1):10741. doi: 10.1038/s41598-021-90226-2

45. Holm JB, Humphrys MS, Robinson CK, et al. Ultrahigh-Throughput Multiplexing and Sequencing of >500-Base-Pair Amplicon Regions on the Illumina HiSeq 2500 Platform. *mSystems* 2019;4(1) doi: 10.1128/mSystems.00029-19

46. van de Wijgert J, Verwijs MC, Agaba SK, et al. Intermittent Lactobacilli-containing Vaginal Probiotic or Metronidazole Use to Prevent Bacterial Vaginosis Recurrence: A Pilot Study Incorporating Microscopy and Sequencing. *Sci Rep* 2020;10(1):3884. doi: 10.1038/s41598-020-60671-6

47. Callahan BJ, McMurdie PJ, Rosen MJ, et al. DADA2: High-resolution sample inference from Illumina amplicon data. *Nat Methods* 2016;13(7):581-3. doi: 10.1038/nmeth.3869

For peer review only - http://bmjopen.bmj.com/site/about/guidelines.xhtml

48. Pruesse E, Quast C, Knittel K, et al. SILVA: a comprehensive online resource for quality checked and aligned ribosomal RNA sequence data compatible with ARB. *Nucleic Acids Res* 2007;35(21):7188-96. doi: 10.1093/nar/gkm864

49. Kyongo JK, Crucitti T, Menten J, et al. Cross-Sectional Analysis of Selected Genital Tract Immunological Markers and Molecular Vaginal Microbiota in Sub-Saharan African Women, with Relevance to HIV Risk and Prevention. *Clin Vaccine Immunol* 2015;22(5):526-38. doi: 10.1128/CVI.00762-14

50. Breiman L. Classification and regression trees. Monterey: Wadsworth & Brooks/Cole Advanced Books & Software 1984. doi: 10.1201/9781315139470

51. Breiman L. Random Forests. *Machine Learning* 2001;45:5-32. doi: 10.1023/A:1010933404324

For peer review only

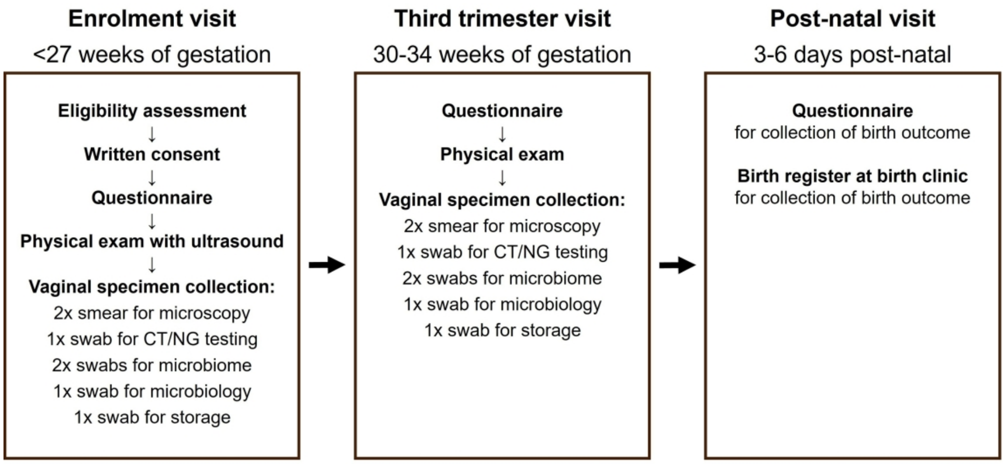

Figure 1 Study visits and specimen collection
Abbreviations: CT, Chlamydia trachomatis; NG, Neisseria gonorrhoeae.

272x125mm (300 x 300 DPI)

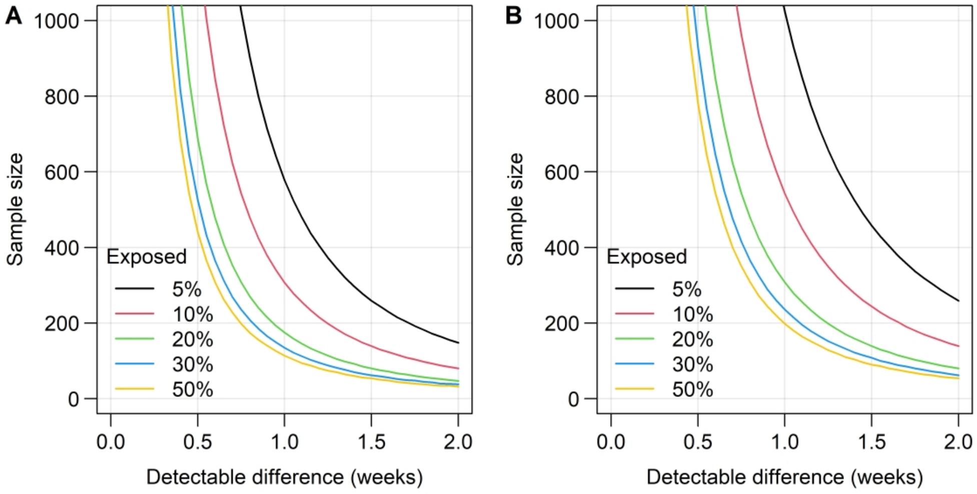

Figure 2 Sample size requirements at different levels of exposure prevalence with power of 80% and alpha 0.83% based on Student's t-test.
Legend: panel A, standard deviation 1.5; panel B, standard deviation 2.0. The curves for % exposed are symmetrical around a prevalence of 50%, i.e., curve for 10% exposed is same as that for 90% exposed.

516x258mm (236 x 236 DPI)

Supplemental online file – study questionnaire

Genital tract infections, the vaginal microbiome and gestational age at birth among pregnant women in South Africa: a cohort study protocol

Ranjana M S Gigi,[1 2] Mandisa M Mdingi,[2] Hyunsul Jung,[3] Shantelle Claassen Weitz,[4] Lukas Bütikofer,[5] Jeffrey D Klausner,[6] Christina A Muzny,[7] Christopher M Taylor,[8] Janneke H H M van de Wijgert*,[9] Remco P H Peters*,[2 3 4] Nicola Low*[1]

1) Institute of Social and Preventive Medicine, University of Bern, Bern, Switzerland

2) Research Unit, Foundation for Professional Development, East London, South Africa

3) Department of Medical Microbiology, University of Pretoria, Pretoria, South Africa

4) Department of Pathology, University of Cape Town, Cape Town, South Africa

5) CTU Bern, Department of Clinical Research, University of Bern, Bern, Switzerland

6) Keck School of Medicine, University of Southern California, Los Angeles, USA

7) Division of Infectious Diseases, University of Alabama at Birmingham, Birmingham, USA

8) School of Medicine, Louisiana State University, USA

9) Julius Center for Health Sciences and Primary Care, University Medical Center Utrecht, Utrecht University, Utrecht, Netherlands

For peer review only

S[illegible]ee[illegible]g a[illegible] [illegible]e[illegible]

Record ID ______________________

Site Information

Today's Date

____

Study Staff Name

____

Start Time

____

Please select Study Site Name

◯ Grey Gateway
◯ Duncan Village CHC
◯ Nontyatyambo CHC
◯ Gompo
◯ Ndevana

Introduction to the Study:

Note to RA:

In this section you will be introducing the study to the participant. Please make sure to execute the following steps:

1. Introduce the study

◯ Proceed

Does the participant show interest in the study

◯ Yes the participant shows interest
◯ No the participant is not interested in the study

END

The participant is not interested in the study. Thank them for their time.

For peer review only

[illegible]g[illegible] S[illegible]ee[illegible]g

Note to RA:
The participant seems to show interest in the study. We need to determine their eligibility status. You will ask a series of questions to determine this. Please select "Proceed" to continue.

◯ Proceed

Is the participant currently living in BCM?

◯ Yes
◯ No

Is the participant 18 years or older?

◯ Yes
◯ No

Please specify the participant's date of birth

____

Calculated age

____

Is this the participant's first ANC visit?

◯ Yes
◯ No

Is the participant within the first 26 weeks of her pregnancy?

◯ Yes
◯ No

Is the participant within the first 20 weeks of her pregnancy?

◯ Yes
◯ No

Gestational weeks

____
(if unknown, enter 99)

Is the participant intending to deliver the baby at one of our collaborating MOUs?

◯ Yes
◯ No

Is the participant currently involved in any other ANC/HIV research trial?

◯ Yes
◯ No

For peer review only

Calculated Eligibility Outcome

____
(1 = Eligible, 0 = Not Eligible)

END
The participant is not eligible for our research study

This will be the end of their participation. Please thank them for their time.

ELIGIBLE
The participant is eligible for our research study. Please select "Proceed" to start with the consenting process.

◯ Proceed

Consenting Process:

NOTE TO RA:
You will now start with the consenting process. Please make sure to do the following:

1. Read the consent form with the participant
2. Read in a language they prefer
3. Allow for questions
4. If willing to consent, sign all documents
5. Hand a signed copy (without PIN) to the patient

◯ Proceed

Did the participant provide a signed consent to participate in the research study?

◯ Yes
◯ No

Consent refusals

Reasons for refusal

☐ They have no time
☐ Scared
☐ In a different study
☐ Other

If "Other", please specify

For peer review only - http://bmjopen.bmj.com/site/about/guidelines.xhtml

Refusal date

____

END
Thank the participant for their time

Provided Consent

Consent date

____

Participant PIN

CONSENTED
The participant has agreed to provide consent. You will now allocate a study PIN to the participant. Please use the next available PIN on the hard copy enrollment log

◯ Proceed

Participant PIN

____

Participant PIN Verification

____

Pin match

____

PIN valid

____

ERROR
The PINs you entered did not match up

You have entered the following PINs

first pin: [participant_pin]
second pin: [participant_pin_verify]

ERROR
The PIN you entered is invalid for [site_name]

You have entered the following PINs

first pin: [participant_pin]
second pin: [participant_pin_verify]

For peer review only

Sa[illegible]g I[illegible]

You have completed the Screening and Enrollment process. Please make sure to check if all relevant fields have been selected and the information captured is accurate.

Once this is done, please select the "complete" option below and then select "Save & Exit".

Once you have done this you will be directed to the baseline Data.

Notes

Additional Notes

For peer review only

[illegible]

Staff name ______________________

Today's Date ______________________

Start Time ______________________

Sociodemographics

NOTE TO RA:
You are about to start the Socio-demographics section. Please make sure to ask the questions as they appear on your tablet.

Please select "Proceed" to continue.

◯ Proceed

Sociodemographics

How would you describe yourself in terms of race?

◯ African
◯ Coloured
◯ Mixed Race
◯ White
◯ Indian
◯ No answer

What level of education did you complete?

◯ Less than Gr. 10
◯ Gr.10 or 11
◯ Gr.12
◯ Diploma
◯ Degree
◯ Refused to answer

For peer review only

Which best describes the type of house in which you live? Please choose one answer only:

◯ House or brick structure on a separate stand or yard or on a farm
◯ Traditional dwelling/hut/structure made of traditional materials
◯ Flat
◯ Town/cluster/semi-detached house (simplex, duplex or triplex)
◯ Unit in retirement village
◯ Dwelling/house/flat/room in backyard
◯ Informal dwelling/shack IN the backyard of a formal house
◯ Informal dwelling/shack NOT in backyard e.g. in an informal/squatter settlement or on farm
◯ Room/flatlet not in backyard but on a shared property e.g granny flat
◯ Caravan/tent
◯ Worker's hostel
◯ Other

If other, please specify.

What is the main material of your house walls? Please choose one answer only:

◯ Bricks & plaster/finished
◯ Bare brick/cement block
◯ Corrugated iron/zinc
◯ Wood
◯ Plastic
◯ Cardboard
◯ Mixture of mud and cement
◯ Wattle and daub
◯ Mud
◯ Other

If other, please specify

What is the main material of your house roof? Please choose one answer only:

◯ Tiles
◯ Corrugated iron/zinc
◯ Thatching
◯ Asbestos
◯ Plastic
◯ Cardboard
◯ Other

If other, please specify

For peer review only

What is your current relationship status?

◯ Married
◯ Steady partner
◯ Steady partner and Casual Partner(s)
◯ Casual Partner(s)
◯ No relationship

Do you live together with your partner?

◯ Yes
◯ No

Are you currently employed?

◯ Employed
◯ Self employed
◯ Not employed

What is your monthly personal income?

◯ None
◯ < 1000 ZAR per month
◯ 1001 - 5000 ZAR per month
◯ 5001 - 10 000 ZAR per month
◯ >10 000 ZAR per month

What is your household's main source of income?

◯ Personal income from employment \ self employment
◯ Income from partner
◯ Grants
◯ Other

Have you been outside of the Eastern Cape or country in the past 6 months?

◯ Yes
◯ No

Which provinces or country have you been to in the last 6 months?

Note to RA: please select all that apply

☐ Free State
☐ Gauteng
☐ Kwazulu-Natal
☐ Limpopo
☐ Mpumalanga
☐ Northern Cape
☐ North West
☐ Western Cape
☐ Outside South Africa

Has your partner/husband been outside of the Eastern Cape or country in the past 6 months?

◯ Yes
◯ No

For peer review only

Which provinces or country has your partner/husband been to in the last 6 months?

Note to RA: Please select all that apply

☐ Free State
☐ Gauteng
☐ Kwazulu-Natal
☐ Limpopo
☐ Mpumalanga
☐ Northern Cape
☐ North West
☐ Western Cape
☐ Other country

What is the main source of drinking water for your household? Please choose one answer only:

◯ Piped (tap) water in dwelling
◯ Piped (tap) water on site or in yard
◯ Neighbour's tap
◯ Public or communal tap (either free or paid)
◯ Borehole on site
◯ Borehole off site/communal
◯ Rain water tank
◯ Water carrier/tanker
◯ Flowing water/stream/river
◯ Stagnant water/dam/pool
◯ Well
◯ Spring
◯ Bottled water
◯ Other

If other, please specify

What type of toilet does your household use? Please choose one answer only:

◯ Flush toilet (connected to sewage)
◯ Flush toilet (with septic tank)
◯ Chemical toilet
◯ Pit latrine with ventilation pipe
◯ Pit latrine without ventilation pipe
◯ Bucket toilet
◯ No facility/bush/field
◯ Other

If other, please specify

For peer review only

What is the main source of energy for cooking in your household? Please choose one answer only:

◯ Electricity from mains
◯ Electricity from generator
◯ Gas
◯ Paraffin
◯ Wood
◯ Coal
◯ Animal dung
◯ Solar energy
◯ Other

If other, please specify

Does your household have any of the following items in good working order? Read each item and indicate the presence of each

	Yes	No
Television	◯	◯
Gas or Electric stove	◯	◯
Fridge/freezer	◯	◯
Private motor vehicle in running condition	◯	◯
Bicycle	◯	◯
Bed	◯	◯
Sofa or sofa set	◯	◯
Kitchen sink	◯	◯

Do you think that you will need to borrow money to pay for healthcare during your pregnancy?

◯ Yes
◯ No

How much money did you spend coming to the clinic today (including transport costs, snacks while waiting etc.) ?

____
([RANDS])

Did you lose any money from your job because of coming to the clinic today?

◯ Yes
◯ No

If yes, how much money did you lose?

____
([RANDS])

How much time did you spend travelling to the clinic today (Hours)?

____
([HOURS])

For peer review only

How much time did you spend travelling to the clinic today (Minutes)?

____
([MINUTES])

Time spent travelling in minutes.

____

How much time do you normally spend waiting and seeing the doctor or nurse in a clinic such as this one (Hours)?

____
([HOURS])

How much time do you normally spend waiting and seeing the doctor or nurse in a clinic such as this one?

____
([MINUTES])

How much time do you normally spend waiting and seeing the doctor or nurse in a clinic such as this one in minutes?

____
([MINUTES])

Are you planning to wait for your results today?
(New question added @13/09/2022)

◯ Yes
◯ No

What is your main reason why you are not intending to wait today?
(New question added @13/09/2022)

◯ Have to get to work
◯ Have to get back to my kids/family
◯ Want to go to the shop
◯ Transport availability
◯ Lack of privacy
◯ Hungry
◯ No space to wait
◯ Not feeling well
◯ Boring
◯ Other

If "Other" , please specify.
(New question added @13/09/2022)

What would make you change your mind?
(New question added @13/09/2022)

For peer review only

Do you do any of the following activities in a lake / stream?

Note to RA: Please select multiple that apply

☐ Play
☐ Bath
☐ Wash blankets
☐ Do laundry
☐ Fish
☐ Collect water
☐ Crossing
☐ None

Behavioural Questionnaire

NOTE TO RA:
You just completed all questions related to socio-demographics. You are about to start with the Behavioral Questionnaire.

Please select "Proceed":

◯ Proceed

When was the last time you had sex?

◯ In the past week
◯ In the past month
◯ More than a month ago

Did you use a condom the last time you had sex?

◯ Yes
◯ No

Do you use a lubricant?

◯ Yes
◯ No

Can you please elaborate on the type of lubricant that you use?

____

What do you use to clean your vagina?

◯ Water only
◯ Soap and water
◯ Other household products
◯ Other

Please specify what other things you used on your vagina.

For peer review only

Do you do any vaginal douching?

◯ Yes
◯ No

Please specify

Do you do any form of vaginal cleansing?

◯ Yes
◯ No

Please specify

Do you use anything to clean inside your vagina?

◯ Yes
◯ No

Do you insert anything in your vagina for tightening?

◯ Yes
◯ No

Please specify

In the past 6 months, how many sexual partners did you have?

◯ One
◯ More than one

In the past 6 months, have you engaged in any of the following?
(Select ALL that apply)

☐ Vaginal sex
☐ Oral sex
☐ Anal sex

Have you recently agreed to sex even though you did not feel like to?

◯ Yes
◯ No

For peer review only

Note to RA: Discuss with participant counselling options

◯ Yes
◯ Participant doesn't need counselling

Please specify

In the past 6 months, have you been forced to have sex with anyone?

◯ Yes
◯ No

Note to RA: Discuss with participant counselling options

◯ Yes
◯ Participant doesn't need counselling

Please specify

In the past 6 months, have you received any benefits (money or goods) for sex?

◯ Yes
◯ No

Do you suspect your steady partner to have any other sex partners?

◯ Yes
◯ No
◯ Unsure

When did your last menstrual period start?

Note to RA: Please ask participant to give the most accurate date.

____

Just before I became pregnant.

NOTE: Please tick the statement that most applies to you:

◯ I wanted to have a baby
◯ I had mixed feelings about having a baby
◯ I did not want to have a baby

How many times have you been pregnant before your current pregnancy?

____

How many live children have you delivered?

____

For peer review only

For peer review only - http://bmjopen.bmj.com/site/about/guidelines.xhtml

Of the live births that you had, how many were normal vaginal delivery?

____

Of the live births that you had, how many were "emergency cesarean"?

____

Of the live births that you had, how many were "elective cesarean"?

____

Live birth Match

____

Note to RA: The numbers you have entered do not match. Please check again.

How many of your live birth's were premature?

____

How many of your live birth's were full term?

____

Delivery timing calc

____

Note: The numbers you have captured do not add up

Have you ever had an ectopic pregnancy?

◯ Yes
◯ No

Have you ever had a miscarriage?

◯ Yes
◯ No

Have you ever had a stillbirth?

◯ Yes
◯ No

For peer review only

Do you smoke cigarettes?

◯ Yes
◯ No

Have you used any of the following since you found out you were pregnant? (select multiple)
(Select ALL that apply.)

☐ Alcohol
☐ Tik
☐ Dagga
☐ Grandpa
☐ Other
☐ None

Please specify

Do you know your current HIV status?

◯ HIV negative (tested today by clinical staff)
◯ HIV positive on ART
◯ HIV positive, not on ART
◯ Don't know (never tested)
◯ Don't know (no yet tested today)

Was the participant newly diagnosed within the past week?

◯ Yes
◯ No

Can we test you for HIV today?

◯ Yes
◯ No

Unknown HIV Status:

Note to RA/ Nurse: HIV test needs to be conducted

◯ Proceed to test

HIV rapid test result:

◯ Positive
◯ Negative

HIV confirmatory test

◯ Positive
◯ Negative

Elisa blood barcode

____

For peer review only

For peer review only - http://bmjopen.bmj.com/site/about/guidelines.xhtml

Have you ever been treated for an STI in the last year?

◯ Yes, I had discharge
◯ Yes, I had ulcers
◯ Yes, I had genital warts
◯ Yes, no symptoms but notified by partner
◯ No

Does the participant have pre-existing diabetes?

◯ Yes
◯ No

Are you on treatment for your diabetes?

◯ Yes
◯ No

Does the participant have pre-existing hypertension?

◯ Yes
◯ No

Are you currently on medication for your hypertension?

◯ Yes
◯ No

Does the participant have pre-existing thyroid disease?

◯ Yes
◯ No

Is the participant taking medication for her thyroid disease?

◯ Yes
◯ No

Do you know your partner's HIV status?

◯ Yes, HIV positive on ART
◯ Yes, HIV positive but not on ART
◯ Yes, HIV negative
◯ No

You have completed the baseline questionnaire. Please make sure to log out of your REDCap account.

Once you have done this you can hand the process over to the nurse who will conduct the clinical history.

For peer review only

□□T□S

Additional notes

For peer review only

P[illegible]a[illegible] [illegible]a[illegible]

Staff Name __________________________________

Today's Date __________________________________

Start time __________________________________

You are about to administer the questions associated with the physical exam.

Please select "Proceed" to continue.

◯ Proceed

Clinical History

Do you currently have any of the following symptoms?

RA: Please select all that apply

☐ Abnormal vaginal discharge
☐ Pain during urination
☐ Lower abdominal pain
☐ Pain related to intercourse
☐ Vaginal bleeding related to intercourse
☐ Genital itchiness
☐ Any skin abnormalities
☐ None

How many days ago did your abnormal vaginal discharge start?

____

How many days ago did the pain during urination start?

____

Provide further details

Have you received treatment for these symptoms?

◯ Yes
◯ No

For peer review only - http://bmjopen.bmj.com/site/about/guidelines.xhtml

Where did you get the treatment from?

◯ Over the counter
◯ Healthcare facility
◯ Traditional healer

Please provide further details

If you were told you had an STI would you disclose to your partner(s)?

◯ Yes, to steady partner
◯ Yes, to casual partner(s)
◯ Yes, to all steady and casual partner(s)
◯ No

Co-Morbidities

You are about to start asking questions related to co-morbidities.

Please select "Proceed" to continue.

◯ Proceed

Did the participant screen positive for any TB symptoms?

◯ Yes
◯ No

The participant shows signs of TB. A specimen needs to be collected for further testing. Please select below to specify whether a specimen was collected successfully.

◯ Yes
◯ No

Instruction: Please record the specimen tracking number below

____

Are you on cotrimoxazole prophylaxes?

◯ Yes, on cotrimoxazole
◯ Yes, started today
◯ No

Did the participant start antiretroviral therapy today?

◯ Yes
◯ No

Specify reason for not starting

For peer review only

Was blood taken today for the participant's CD4 count?

◯ Yes
◯ No

Please record barcode for blood tube for CD4 count testing?

____

Is the participant's most recent CD4 count within the last 12 months available?

◯ Yes
◯ No

What was the date of the CD4 specimen collection?

____

What was the participant's most recent CD4 count?

____
(if no number listed, enter 9999)

Was blood taken today for the participant's viral load?

◯ Yes
◯ No

Please record barcode for blood tube for viral load testing?

____

Is the participant's most recent viral load within the past 12 months available?

◯ Yes
◯ No

What was the date of the viral load specimen collection?

____

What was the participant's most recent viral load?

____
(if no number listed, enter 0000)

Was blood taken today for the participant's viral load?

◯ Yes
◯ No

Please record the barcode for viral load testing?

____

For peer review only

For peer review only - http://bmjopen.bmj.com/site/about/guidelines.xhtml

Is the participant's most recent viral load available?

◯ Yes
◯ No

What was the date of the viral load specimen collection?

____

What was the participant's most recent viral load?

____

Which regimen for ART were you started on today?

◯ TLD
◯ TEE
◯ AZT/3TC/LPV
◯ Other

Which regimen for ART were you on so far?

◯ TLD
◯ TEE
◯ AZT/3TC/LPV
◯ Other

Has the regimen for ART been changed today?

◯ Yes
◯ No

To which regimen for ART has it been changed today?

◯ TLD
◯ TEE
◯ AZT/3TC/LPV
◯ Other

Please select "Proceed" to collect blood for viral load testing.

◯ Proceed

Did you successfully collect blood for viral load testing?

◯ Yes
◯ No

Please capture the barcode associated with the blood tube used for testing viral load.

____

For peer review only

Was a CD4 count test done?

◯ Yes
◯ Yes, but no result available
◯ Not done

Please specify last known CD4-cell count.

____

Was blood taken today for the participant's CD4 count ?

◯ Yes
◯ No

Please record the barcode for CD4 testing?

____

Is the participant's most recent CD4 available?

◯ Yes
◯ No

What was the date of the CD4 specimen collection?

____

What was the participant's most recent CD4 Count?

____

Has a syphilis test been done for the participant?

◯ Yes
◯ No

Instruction: Please conduct a rapid test for syphilis.

Specify if the participant agreed to testing / you managed to execute the test.

◯ Yes
◯ No

Which syphilis test have you used?

◯ Alere Syphilis TP (provided by FPD)
◯ HIV/Syphilis Duo (provided by FPD)
◯ No rapid test used only NHLS bloods for RPR
◯ Other please specify

Please specify

For peer review only

Syphilis result

◯ Positive
◯ Negative
◯ Indeterminate

Titer value 1:

____

Please collect blood for further syphilis testing and specify if the blood was collected successfully.

◯ Yes
◯ No

Treatment given

◯ Benzathine penicillin, 2.4 mU
◯ Out of stock

Please contact the study clinician and specify treatment given to participant

Please capture the barcode below.

____

Participant weight in kilograms

____

Participant height in cm

____

Participant systolic blood pressure

____

Participant diastolic blood pressure

____

How was Hemoglobin measured?

◯ Hb meter at the clinic
◯ Hb at NHLS

Please capture Hb result

____
(1 Decimal Place)

For peer review only

For peer review only - http://bmjopen.bmj.com/site/about/guidelines.xhtml

Please capture the barcode for Hb

____

Participant MUAC in cm

____
(1 Decimal Place)

Please collect participant's urine for later testing.

Ultrasound Results

You are about to start capturing information pertaining to the ultrasound results.

Please select "Proceed" to continue.

◯ Proceed

Ultra-sound scan date

____

Was the pregnancy confirmed?

◯ Yes, intra-uterine
◯ Yes, extra-uterine
◯ No

NOTE: Please refer immediately

NOTE
Due to the status of the pregnancy, the participant is no longer eligible to continue with the study. This is the end of their involvement in the study. Please thank them for their time. Also do the following:

- Save and Exit the form

- Complete a Study Note confirming termination of study participation

Please specify the number of foetus

____

For peer review only - http://bmjopen.bmj.com/site/about/guidelines.xhtml

You are about to capture the gestational age of the foetuses. Please select "Proceed" and then capture the number of weeks followed by days.

◯ Proceed

Gestational age in weeks

____

Gestational age in days

____

Calc: Gestational age in days

____

EDD based on ultra-sound

____

Calc: Days to EDD

____

Calc assist for EDD

The number of days must be equal to 280.

____

Note to Nurse:

You did not enter either gestational age or EDD correctly.

Calc: Eligibility

____

END
The participant is not eligible for our research study

This will be the end of their participation. Please thank them for their time.

STI Clinical Examination (To be done By Nurse)

You are about to capture information related to STI clinical examination.

Please select "Proceed" to continue

◯ Proceed

For peer review only

During your examination, were there any signs of abnormal vaginal discharge?

◯ Yes
◯ No

During your examination, were there any signs of inguinal lymphadenopathy?

◯ Yes
◯ No

Are these bubo?

◯ Yes
◯ No

Note to RA: Please contact study clinician and specify treatment given to participant

During your examination, were there any signs of lower abdominal pain?

◯ Yes
◯ No

During your examination, where there any signs of scratch marks?

◯ Yes
◯ No

During your examination, where there any signs of skin conditions?

◯ Yes
◯ No

Please specify the nature of the skin conditions

During your examination, where there any other observations that need to be noted?

For peer review only

[illegible]

You are about to capture the results from the dipstick testing

Please select "Proceed" to continue

◯ Proceed

Blood - Hemoglobin

◯ Negative
◯ Ca. 10
◯ Ca. 50
◯ Ca. 250/300

Blood - Erythrocytes

◯ Negative
◯ Ca. 5 -10
◯ Ca. 50
◯ Ca. 250/300

Urobilinogen

◯ Normal
◯ 2
◯ 4
◯ 8
◯ 12

Bilirubin

◯ Negative
◯ 1 plus
◯ 2 plus
◯ 3 plus
◯ Not available

Protein

◯ Negative
◯ 30
◯ 100
◯ 500

Nitrate

◯ Negative
◯ Positive

For peer review only

Keton

◯ Negative
◯ 1 plus
◯ 2 plus
◯ 3 plus
◯ Not available

Glucose

◯ Negative
◯ Normal
◯ 50
◯ 150
◯ 500
◯ ≥1000

pH

◯ 5
◯ 6
◯ 7
◯ 8
◯ 9
◯ Not available

SG

◯ 1.000
◯ 1.005
◯ 1.010
◯ 1.015
◯ 1.020
◯ 1.025
◯ 1.030
◯ Not available

Leucocytes

◯ Negative
◯ 25
◯ 75
◯ 500

NOTE
The participant's clinical gestational age is more than 20 weeks. They are not eligible to proceed with the study activities.

Please do the following:

1. Explain the reason for study termination
2. Complete the study electronic termination tool
3. Complete the study termination document and place in file

◯ End

For peer review only

□□*T*□*S*

Additional Notes

You have completed capturing the information from the clinical exam. Please make sure to check that you have completed all the fields.

Please select "Complete" then "Save and Exit".

You will now proceed to collecting study specimens and randomization

For peer review only

S□e□□□e□□ a□□ Ra□□□□□□a□□□□

Staff Name __________________________________

Today's Date __________________________________

Start time __________________________________

Specimen Collection

NOTE
You will now start with the process of specimen collection. You will need to collect several specimens from the participant. These specimens need to be collected in the order in which they are presented here. The outcome of the randomization will have an impact on whether these specimens will be tested immediately or whether they will need to be prepared for storage.

The following specimens will need to be collected:
1. Vaginal loop to be used to prepare two slides
2. Vaginal swab to be used for STI testing
3. Vaginal swab to be used for profiling
4. Vaginal swab to be used for microbiome
5. Vaginal swab to be used for cytokine

◯ Done

NOTE
You will now start with the process of specimen collection. You will need to collect several specimens from the participant. These specimens need to be collected in the order in which they are presented here.

The following specimens will need to be collected:
1. 1 x Vaginal loop to be used to prepare two slides
2. 1 x Vaginal swab to be used for STI testing
3. 1 x Vaginal swab to be used for profiling
4. 2 x Vaginal swab to be used for microbiome
5. 1 x Vaginal swab to be used for cytokine

◯ Done

Vaginal Smear

Please specify the vaginal pH

____
(if not available, enter 99)

For peer review only

Please select which pH strips are used to measure vaginal pH

◯ CardinalHealth pH Indicator Strips (range 3.6-6.1)
◯ pH Indicator Strips pH 0-14
◯ Natureland vaginal pH test (range 3.5-6.5)

You will need to use a single loop to collect vaginal smear on two glass slides for microscopy

◯ Done

Confirm the PIN associated with the first vaginal slide that will be used for Nugent score

◯ [participant_pin]-S1

Confirm the PIN associated with the second vaginal slide that will be used for yeast microscopy

◯ [participant_pin]-S2

Vaginal Swabs

NOTE
You will now collect four vaginal swabs. They will be used as follows:

1. STI testing (test for arms 1 and 2, store for arm 3)
2. Profiling (stored)
3. Microbiome (stored)
4, Cytokine

◯ Done

NOTE
You will now collect four vaginal swabs. They will be used as follows:

1. STI testing
2. Profiling (stored)
3. Microbiome (stored)
4, Cytokine

◯ Done

Please confirm the PIN associated with the urine for Schistosomiasis testing.
(2022/10/21 - Stopped collecting the urine specimen)

◯ [participant_pin] - UD1

Please confirm the PIN associated with the vaginal swab that will be used for STI testing.

◯ [participant_pin] - BV1

Please confirm the PIN associated with the vaginal swab that will be used for profiling.

◯ [participant_pin] - BV2

Please confirm the PIN associated with the vaginal swab that will be used for microbiome.

◯ [participant_pin] - BV3

For peer review only

Please confirm the PIN associated with the vaginal swab that will be used for cytokines.

◯ [participant_pin] - BV4

NOTE
You have finished the collection of the vaginal swabs. Please ensure specimens have been prepared for storage and shipment. The vaginal swab that is collected for STI testing should be kept aside following the outcome of the randomization. If the participant is in arm 1 or 2 the specimen should be used for immediate testing. If however, the participant is randomized to arm 3, you can store the specimen.

Please select "Proceed" to start the process of randomization

◯ Done

Randomization	◯ Arm 1<br>◯ Arm 2<br>◯ Arm 3

Activities Associated with "[randomization]"

NOTE
The participant has been randomized to "[randomization]". You will now need to do the following:

1. Prepare the STI swab for testing using the GeneXpert

◯ Done

NOTE:
The participant has been randomized to"[randomization]". You will now need to do the following:

1. Prepare the STI swab for testing using the GeneXpert
2. Screen for symptoms
3. Provide treatment and partner referral if positive

◯ Done

NOTE:
The participant has been randomized to "[randomization]". You will now need to do the following:

1. Screen for symptoms
2. Provide treatment and partner referral if positive

◯ Done

NOTE
You will now need to do the following:

1. Prepare the STI swab for testing using the GeneXpert

◯ Done

STI Results

	Positive	Negative
CT	◯	◯

For peer review only

NG	◯	◯
TV	◯	◯

NOTE: See Calculation:

The result from the STI test?

____
(0 = Negative, 1 = Positive, 2 = No result)

Date the result was obtained

____

Did the participant wait for her STI results?
(New question added @ 03/11/2022)

◯ Yes
◯ No

Symptomatic Screening Outcome Following Negative Test

The result from the GeneXpert was negative.

Was the participant reporting STI symptoms or showed symptoms during the clinical assessment?

◯ Yes
◯ No

Does the participant report any medication allergies?

◯ Yes
◯ No

Please contact study clinician before giving any treatment. Please specify discussed medication allergies and treatment plan with study clinician

The following treatment has been provided

☐ Azithromycin 1g stat dose
☐ Azithromycin 2g stat dose
☐ Ceftriaxone 250mg IM injection
☐ Ceftriaxone 1g IM injection
☐ Metronidazole 400mg bd x 1 week
☐ Metronidazole 2g stat dose
☐ Clotrimazole pessary and/or cream
☐ Trimethoprim/sulfamethoxazole 400/80 mg 2 tbl. bds for 5 days (bactrim)
☐ Ceftriaxone 500mg IM injection

Date treatment given

____

For peer review only

What made you change your mind about waiting for the results?
(New question added @13/09/2022)

Partner notification provided

◯ Yes, 1
◯ Yes, multiple
◯ No

Please explain why the partner notification note was not provided?

ELIGIBLE
The participant is eligible for our nested chlamydia case-control study. Please select "Proceed" to start with the consenting process.

◯ Proceed

Did the participant provide a signed consent to participate in the chlamydia case control study?

◯ Yes
◯ No

Reasons for refusal

☐ They have no time
☐ Scared
☐ In a different study
☐ Other

If "Other", please specify

Consent or refusal date

____

NOTES

Participant successfully enrolled __________________________________

Additional notes

For peer review only

You are done with all activities associated with "[randomization]". Please hand the tablet over to the RA to capture the remaining schedule dates.

You are done with all activities. Please hand the tablet over to the RA to capture the remaining schedule dates.

For peer review only

S□□e□□□□□g

Scheduling of Dates Associated with [randomization].

NOTE
You are about to schedule dates associated with [randomization] participants.

Please select "Proceed".

◯ Proceed

Scheduling of Dates Associated

NOTE:
You are about to schedule dates associated with microbiome participants.

Please select "proceed"

◯ Proceed

Scheduling Dates for 3-Week ToC

NOTE: The participant tested positive and therefore we need to schedule a date, exactly 3-weeks from today to conduct a test-of-cure.

Calculator Assist

The number here must be equal to 21

____

Scheduling the 3-week ToC

NOTE:
Please schedule a date, 3 weeks from today treatment given. Please use the calculator assistance to ensure that you schedule a date exactly 21 days from today.

____

ERROR
The field does not equal to 21, please change it

Have you handed the TOC date to the participant?

◯ Yes
◯ No

Scheduling Dates Associated with ToC Reminder

Schedule date for REMINDER of 3-week ToC visit

____

For peer review only

Calculator Assist for scheduling ToC reminder date

The reminder phone call will be made 18 days following the treatment date. The number of days need to equal to 18.

____

ERROR
You did not enter the date correctly. The number should equal to 18. Please redo the date.

Scheduling Dates Associated with 3-Week ToC Missed Visit Date

NOTE:
You have successfully scheduled the reminder date.

Please select "proceed" to schedule the missed visit date for the 3 week ToC visit.

◯ Proceed

Schedule the date for the MISSED VISIT of the ToC visit.

This date should be 3 weeks after the date on which the participant received their test result.

____

Calculator Assist for scheduling 3-week ToC Missed Visit

The participant's time period allowed for attending a ToC will start 35 days after they received their result and will close 35 days after the date they received their result.

The number here must show 35

____

ERROR
You did not enter the date correctly. The number should equal to 35. Please redo the date.

NOTE:
You have successfully scheduled the 3-week ToC close date

Please select "proceed" to start scheduling the next visit dates

◯ Proceed

Dates Associated with reminder for the 28 Week call

NOTE:
You are about to schedule dates for the call reminder at 28 weeks.

Please select "Proceed".

◯ Proceed

Note:
Schedule the date for the 28 week call. We will contact each participant to ask the date for their 30 weeks clinic visit is.

____

For peer review only - http://bmjopen.bmj.com/site/about/guidelines.xhtml

Calculation assist for scheduling the 32-week reminder date.

This number must equal to 196

____

Days to call reminder

____

ERROR
The number you have entered does not match 196. Please select a different date so that the number equals to 196.

CONGRATULATIONS

You have finished scheduling all dates.

NOTES

Notes box

For peer review only

[illegible]

Staff

____

Date

____

Time

____

You are about to capture results of specimens collected during the baseline visit.

Please select "Proceed" to continue

◯ Proceed

Hb results received

◯ Yes
◯ No

Please capture Hb result

____

Please capture the barcode for Hb

____

Please capture the results of the sputum for TB testing

◯ MTB Negative
◯ MTB Positive Rifampicin Susceptible
◯ MTB Positive Rifampicin Resistant
◯ Not suitable
◯ Specimen missing
◯ Invalid
◯ Not applicable

Please contact participant

Please recollect specimen on participant next visit

CD4 count results received?

◯ Yes
◯ Clotted blood
◯ Missing

Please recollect blood or collect outcome from ART clinic

Please record the barcode for CD4 count testing

____

For peer review only

Please capture the result of the blood tube collected for CD4 count testing

____

Date sample for CD4 count was taken

____

Viral load results received?

◯ Yes
◯ Clotted blood
◯ Missing

Please recollect blood or collect outcome from ART clinic

Please capture the result of the blood tube collected for viral load testing

____

Date sample for viral load was taken

____

Please record barcode for viral load testing

____

Please capture the result of the syphilis testing

◯ RPR Negative
◯ RPR Positive
◯ RPR Indeterminate
◯ Not received

Please contact participant

____

Notes

Notes

For peer review only

For peer review only - http://bmjopen.bmj.com/site/about/guidelines.xhtml

□P S□e□□□e□ Re□□□□□

Staff ______________________

Date ______________________

Time ______________________

You are about to capture results of specimens collected during the baseline visit.

Please select "Proceed" to continue

◯ Proceed

Please capture the result for the Nugent score testing

◯ Slide reading not satisfactory
◯ Slide reading was satisfactory

____

Please capture the result for the Nugent score testing

____

Please specify if yeast was present

◯ Yes
◯ No

Please capture the result for the yeast infection testing

◯ Slide reading not satisfactory
◯ Slide reading was satisfactory

____

Please capture the result for the Yeast Infection testing

____

Please specify if candida was present

◯ Yes
◯ No

For peer review only

Please capture the result for the schistosomiasis

◯ Positive
◯ Negative
◯ Indeterminate

Please specify the result for the schistosomiasis

☐ Trace
☐ 1+
☐ 2+
☐ 3+
☐ 4+

____

Note:

Please notify the study clinician

Notes

Notes

For peer review only

□a□□□□g Re□□□□e□□

Staff Name ________________________________

Today's Date ________________________________

Time ________________________________

Opening date for Week 30-34 ________________________________

Closing date for Week 30-34 ________________________________

NOTE:
You are about to call a participant to remind them of a specific visit. Please make sure to do the following:

1. Obtain all relevant contact numbers for the participant on their record
2. Ensure that you have checked what the exact date is when the participant is expected to present
3. Make sure to give the participant a brief description of what will be done at the visit.
4. You will make up to 3 attempts to get hold of the participant.

Please select "Proceed"

◯ Proceed

The presentation dates are below:

TOC
Date: [baseline_arm_1][toc_3week]

WEEK 28 CALLING

Date: [baseline_arm_1][sched_28w_rem]

WEEK-32

30-34 Week Open Date: [week_28_arm_1][calling_wk30_34_open_date]

30-34 Week Close Date: [week_28_arm_1][calling_wk30_34_close_date]

30-34 Week Actual Date: [week_28_arm_1][week32_visit_date]

POST-NATAL VISIT

Visit open date: [predelivery_checki_arm_1][pd_remind_date_schedpd]

Visit close date: [predelivery_checki_arm_1][pd_close_date_schedpd]

Date of Delivery: [predelivery_checki_arm_1][pd_remind_date_delivery]

For peer review only

6-WEEK IMMUNIZATION VISIT

Actual Date: [predelivery_checki_arm_1][sixw_im_schedpd]

Please select the calling attempt

☐ First Attempt
☐ Second Attempt
☐ Third Attempt

Details of Calling Attempt 1

Outcome of the attempt

◯ Successful - Participant
◯ Successful - Family member
◯ Unsuccessful - Voicemail
◯ Unsuccessful - Invalid

Date of the attempt

____

Details of Calling Attempt 2

Outcome of the attempt

◯ Successful - Participant
◯ Successful - Family member
◯ Unsuccessful - Voicemail
◯ Unsuccessful - Invalid

Date of the attempt

____

Details of Calling Attempt 3

Outcome of the attempt

◯ Successful - Participant
◯ Successful - Family member
◯ Unsuccessful - Voicemail
◯ Unsuccessful - Invalid

Date of the attempt

____

For peer review only

e

Calling notes

For peer review only

[illegible]

Staff Name ______________________

Today's date ______________________

Start time ______________________

NOTE
Did the participant present within the specified dates presented below:

Start: [baseline_arm_1][toc_reminder_date]

Actual: [baseline_arm_1][toc_3week]

End: [baseline_arm_1][arm1_toc_close_date]

◯ Yes
◯ No

The participant did not have a positive baseline STI result and therefore a ToC visit is not applicable. Activities associated with this visit will need to be captured under the "Ad-Hoc" tool

Note
You are about to start activities associated with the Test-of-Cure visit for participants in arm 1. The following activities are associated with this visit:

1. Collect 1 Loop with 2 slides
2. Collect 3 vaginal Swabs
- Test of Cure Test
- Profiling (Storage)
- Microbiome (Storage)
3. Running the Test-of-Cure
4. Collect Clinical History, Adherence and Disclosure data

Please select "Proceed"

◯ Proceed

Specimen Collection_ToC

NOTE
Please collect one vaginal loop and prepare 2 slides. Please remember to do the following:

1. Pack slides individually in their own package
2. Record the PIN on the outside of package
3. Complete the lab CRF with the matching PINs and test instructions

Select "Proceed" to confirm the PINs associated with the slides.

◯ Proceed

For peer review only

Did you manage to collect the vaginal loop?

◯ Yes
◯ No

Date of vaginal loop specimen collection

____

Please confirm the pin for the first vaginal swab that will be used for Nugent score

◯ [baseline_arm_1][participant_pin]-TL1

Please confirm the pin for the second vaginal swab that will be used for yeast microscopy

◯ [baseline_arm_1][participant_pin]-TL2

NOTE:
You are about the start with the process of collecting the following 3 vaginal swabs:

1. Swab to be used to conduct ToC (Immediately)
2. Swab for profiling
3. Swab to be used for microbiome

Please select "Proceed"

◯ Proceed

Please specify the vaginal pH

____

Please confirm the PIN associated with the vaginal swab that will be used for STI testing.

◯ [baseline_arm_1][participant_pin] - TCV1

Did you manage to collect the vaginal swab for profiling

◯ Yes
◯ No

Please confirm the PIN associated with the vaginal swab that will be used for profiling.

This must be stored

◯ [baseline_arm_1][participant_pin] - TCV2

Did you manage to collect the vaginal swab for microbiome testing?

◯ Yes
◯ No

Please confirm the PIN associated with the vaginal swab that will be used for microbiome.

This must be stored

◯ [baseline_arm_1][participant_pin] - TCV3

For peer review only - http://bmjopen.bmj.com/site/about/guidelines.xhtml

Date of specimen collection for vaginal swabs

____

NOTE
You have collected all specimens associated with this visit. Once you select the "Proceed" option below you will be directed to the start of the clinical history questionnaire. The completion of the questionnaire might take some time so it would be a good idea to start running the vaginal swab to conduct the Test of Cure in line with the below baseline results.

NG: [baseline_arm_1][sti_result_ng]

TV: [baseline_arm_1][sti_result_tv]

CT: [baseline_arm_1][sti_result_ct]

○ Proceed

Clinical History Review

You are done trying to collect specimens. Because your were not able to collect a Vaginal Swab for STI testing you will not be able to run a test. Please proceed to completing the clinical history.

○ Proceed

Do you currently have any of the following symptoms?
Multiple selection

☐ Abnormal vaginal discharge
☐ Pain during urination
☐ Lower abdominal pain
☐ None

When did these symptoms start for abnormal vaginal discharge?

○ After previous visit
○ Persistent since previous visit
○ Recurrent since previous visit

When did these symptoms start for pain during urination?

○ After previous visit
○ Persistent since previous visit
○ Recurrent since previous visit

For peer review only - http://bmjopen.bmj.com/site/about/guidelines.xhtml

[illegible]

NOTE
The following questions pertain to adherence to the STI medication.

Select Proceed

◯ Proceed

Did you finish the whole course of treatment?

◯ Yes
◯ No

How many days did you take treatment for?

____

Did you throw up within 2 hours after taking any of the STI treatment?

◯ Yes
◯ No

Did you take any other non-chronic treatment at the time?

◯ Yes
◯ No

What type of treatment were you taking ?

NOTE
You are done with questions related to Adherence. You are about to start asking questions associated with Disclosure.

Please select "Proceed"

◯ Proceed

Disclosure

Did you have sex in the past month?

◯ Yes
◯ No

For peer review only

How many different male partners did you have sexual intercourse with in the past month ?

◯ 1
◯ 2
◯ More than 2 partners

Please specify how many partners?

____

What type of sex did you have with partner 1 (Husband/ Steady partner)?

☐ Vaginal
☐ Anal
☐ Oral

Did you use a condom the last time you had sex with this partner?

◯ Yes
◯ No

Did you notify him of your STI result?

◯ Yes I gave him the notification slip
◯ Yes I told him
◯ No

What was his reaction when you told him of your STI infection?

◯ Supportive
◯ Angry
◯ Violent
◯ Disengaged

How did disclosure affect your relationship?

◯ Continued as before
◯ Started using a condom
◯ He engaged with other partners
◯ He refused sex
◯ Relationship ended

Did he take the treatment?

◯ Yes
◯ No
◯ Don't know

Where did he seek treatment?

◯ Private
◯ Public
◯ Traditional

For peer review only

Why did you not notify this partner?

◯ I didn't feel it was necessary
◯ I am embarrassed
◯ I'm afraid he gets angry
◯ I'm afraid he gets violent
◯ I'm afraid he will end the relationship

What type of sex did you have with partner 2?

☐ Vaginal
☐ Anal
☐ Oral

Did you use a condom the last time you had sex with this partner?

◯ Yes
◯ No

Did you notify him of your STI result?

◯ Yes I gave him the notification slip
◯ Yes I told him
◯ No

What was his reaction when you told him of your STI infection?

◯ Supportive
◯ Angry
◯ Violant
◯ Disengaged

How did disclosure affect your relationship?

◯ Continued as before
◯ Started using a condom
◯ He engaged with other partners
◯ He refused sex
◯ Relationship ended

Did he take the treatment?

◯ Yes
◯ No
◯ Don't know

Where did he seek treatment?

◯ Private
◯ Public
◯ Traditional

For peer review only

Why did you not notify your partner?

◯ I didn't feel it was necessary
◯ I am embarrassed
◯ I'm afraid he gets angry
◯ I'm afraid he gets violent
◯ I'm afraid he will end the relationship

Did you tell anyone else of your STI infection?

◯ Yes
◯ No

Who did you tell?
(Select multiple)

☐ Family member
☐ Friend
☐ Healthcare worker
☐ Other

NOTE:
You have completed the ToC questionnaire. Please select "Proceed" to capture the outcome of the STI test.

◯ Proceed

ToC Outcome

	Positive	Negative	Did not test
CT	◯	◯	◯
NG	◯	◯	◯
TV	◯	◯	◯

NOTE: See calculation

The result from the STI result ________________ (1 = Positive, 0 = Negative)

Does the participant show any symptoms of an STI?

◯ Yes
◯ No

Please contact the study clinician and discuss treatment.

For peer review only

The participant tested/screened positive for an STI. Please specify the treatment that has been provided.

☐ Azithromycin 1g stat dose
☐ Azithromycin 2g stat dose
☐ Ceftriaxone 250mg IM injection
☐ Ceftriaxone 1g IM injection
☐ Metronidazole 400mg bd x 1 week
☐ Metronidazole 2g stat dose
☐ Clotrimazole pessary and/or cream
☐ Ceftriaxone 500mg IM injection

Date treatment given

____

Please specify if a partner notification has been given to the patient.

◯ Yes
◯ No

NOTE
The patient did not test positive or show any signs of an infection

Select "Proceed" to conclude visit

◯ Proceed

Notes

Additional notes

You have completed the ToC Visit Activities. Please make sure to check if all relevant fields have been selected and the information captured is accurate.

Once this is done, please select the "Complete" option below and then select "Save & Exit".

For peer review only

[illegible]

You are about to capture the results of the first loop used for Nugent scoring.

Please select "Proceed" to continue

◯ Proceed

Was the reading satisfactory for the Nugent score?

◯ Yes
◯ No

____

Please specify the Nugent score

____

Please specify if candida was present

◯ Yes
◯ No

Additional comments

You are about to capture the results of the second loop used for smear microscopy.

Please select "Proceed" to continue

◯ Proceed

Was the reading satisfactory?

◯ Yes
◯ No

____

Please specify the Nugent score

____

For peer review only

Please specify if candida was present

◯ Yes
◯ No

Additional comments

For peer review only

S[illegible]e[illegible]g_[illegible]e

Scheduling the Dates Associated with the 32 Week Gestational Visit

The pregnancy (in days) is currently:

____

NOTE
You are about the start scheduling dates associated with the 32-week visit. You will need to schedule the following associated dates:

1. Week 32 date - Actual visit

2. Week 32 reminder date

3. Week 30 date - Visit window opens

4. Week 35 date - Visit window closes

Select "Proceed" to start scheduling

◯ Proceed

Schedule the date for the 32 week gestational age, visit

Note to RA: please make sure that this date does not fall on Friday, weekend, and public holidays.

____

Calculate assist for 32-week visit

The number here must between 210 and 244.

____

Days Difference (the difference between 32 weeks & Gestational age)

____

Match

____

The date you have entered does not meet the 93 day criteria. Does the intended or original date fall on a Friday weekend or public holiday?

◯ Yes
◯ No

ERROR
The numbers you have entered does not match. Please select a different date so that the numbers match.

Dates Associated with reminder for the 32 Week Gestational Age Visit

For peer review only

□a□e□ a□□□□□a□e□ □□□□ □□e 32 □ee□□ ge□□a□□□□a□ age □□□□e□ □□□□□

NOTE:
You have successfully scheduled the 32-week date.

We will need to contact the participant at least 3 days before the scheduled visit to remind them.

Select "Proceed" to schedule the reminder date for the 32 week visit.

◯ Proceed

Note:
Schedule the date for the 32 week reminder. We will contact each participant starting 3 days prior to their 32-week gestation date. That means the date scheduled here should be 3 days earlier then the scheduled date for the 32-week visit. If the date falls on a weekend choose the closest week date.

____

Calculation assist for scheduling the 32-week reminder date.

This number must be between 1 and 4.

____

ERROR
The date that you have entered is invalid. Please select a different date so that the number is less than or equal to 3.

NOTE:
You have successfully scheduled the 32-week reminder date.

Select "Proceed" to schedule the 32 week open visit date.

◯ Proceed

Schedule the date for the 32 weeks opening visit date.

Note: Participants will have from 30 weeks of gestation to present for their 32-week visit date.

____

Calculation Assist for scheduling the 32-Week opening visit date.

This number must equal to 210

____

ERROR
The number you have entered does not match 210. Please select a different date so that the number equals 210.

NOTE:
You have successfully scheduled the 32-week opening date.

Select "Proceed" to schedule the 32 weeks missed visit date.

◯ Proceed

For peer review only

Schedule the date for the 32 weeks missed visit date.

Note: Participants will have until 34 weeks of gestation to present for their 32-week visit date after which the visit will be closed out.

____

Calculation Assist for scheduling the 32-Week missed visit date.

This number must equal to 244

____

ERROR
The number you have entered does not match 244. Please select a different date so that the number equals to 244.

Estimated Delivery Date

You are about the schedule the Estimated Delivery Date.

Please select "proceed"

◯ Proceed

Estimated Delivery Date

____

Days difference between estimated date of delivery and gestational age

____

Calculation Assist for scheduling the Estimated Date for Delivery date.

This number must equal to [edod_calc]

____

Match ________________________________

ERROR
The number you have entered does not match. Please select a different date so that the numbers match

You have completed all the scheduling dates.

Please check that all dates entered comply with the "calculation assistance".

For peer review only - http://bmjopen.bmj.com/site/about/guidelines.xhtml

You are about the schedule dates associated with the following events:

1. Pre-birth check-inn

◯ Proceed

Check-In Calling at 37 Weeks

Proceed to the check-in calling date

◯ Proceed

Check-in calling date

____

Calculation Assist for check-in calling date

This number must equal to 259

____

NOTE
The date you have entered is incorrect. Please make sure that the numbers correspond.

CONGRATULATIONS

You have finished scheduling all dates.

For peer review only

32[illegible] ee[illegible] [illegible] [illegible]e[illegible]

Staff name	__________________________________
Today's date	__________________________________
Start time	__________________________________

Did the participant present within the dates presented below:

30-34 Week Start Date: [week_28_arm_1][sched_32w_open_date]

30-34 Week Actual Date: [week_28_arm_1][week32_visit_date]

30-34 Week Closing Date: [week_28_arm_1][sched_32w_mv_date]

◯ Yes
◯ No

Open ad-hoc visit to capture relevant information

Specimen Collection_32-Week

NOTE
You will now start with the process of specimen collection. You will need to collect several specimens from the participant. These specimens need to be collected in the order in which they are presented here.

The following specimens will need to be collected:
1. 1 x Vaginal loop to be used to prepare two slides
2. 1 x Vaginal swab to be used for STI testing (Arm 2 and Microbiome (Empilweni): immediate testing; Arm 1 and 3: Storage)
3. 1 x Vaginal swab to be used for profiling
4. 2 x Vaginal swab to be used for microbiome
5. 1 x Vaginal swab to be used for cytokine

◯ Proceed

Vaginal Smear

Please specify the vaginal pH

____
(if not available, enter 99)

For peer review only

Please select which pH strips are used to measure vaginal pH

◯ CardinalHealth pH Indicator Strips (range 3.6-6.1)
◯ pH Indicator Strips pH 0-14
◯ Natureland vaginal pH test (range 3.5-6.5)

You will need to use a single loop to collect vaginal smear on two glass slides for microscopy (if not available, enter 99)

◯ Done

Confirm PIN associated with the first Vaginal Loop to be used to test for Nugent score

◯ [baseline_arm_1][participant_pin] - WL1

Confirm PIN associated with the second Vaginal Loop to be used to test for Yeast microscopy

◯ [baseline_arm_1][participant_pin] - WL2

Vaginal Swabs

NOTE
You will now collect four vaginal swabs. They will be used as follows:

1. STI testing (Arm 2 and Microbiome (Empilweni): immediate testing; Arm 1 and 3: Storage)
2. Profiling (stored)
3. Microbiome (stored)
4, Cytokine (stored)

◯ Done

Confirm PIN associated with the vaginal swab to be used to test for STI

◯ [baseline_arm_1][participant_pin] - WV1

Please confirm PIN associated with the vaginal swab to be used for Profiling

◯ [baseline_arm_1][participant_pin] - WV2

Please confirm PIN associated with the vaginal swab to be used for microbiome

◯ [baseline_arm_1][participant_pin] - WV3

Please confirm the PIN associated with the vaginal swab that will be used for cytokines.

◯ [baseline_arm_1][participant_pin] - WV4

NOTE
The participant is in arm 2 and therefore an immediate STI test is conducted at the 32-week visit. Please prepare the swab for testing before you continue to the questionnaires.

◯ Proceed

NOTE
You are done with all specimen collection and will now proceed to administering the clinical history.

Please select "Proceed"

◯ Proceed

For peer review only

[illegible]

Have you been to the clinic since the last visit with us?

◯ Yes
◯ No

What was the purpose of your visit?

☐ ANC Visit
☐ HIV/ART
☐ STI Treatment
☐ Other

Summary notes from visit

Have you used any of the following since the first study visit?
Select multiple

☐ Alcohol
☐ Tik
☐ Dagga
☐ Grandpa
☐ Other
☐ None

Please specify other drugs used?

Do you currently have any of the following symptoms?

RA: Please select all that apply

☐ Abnormal vaginal discharge
☐ Pain during urination
☐ Lower abdominal pain
☐ Pain related to intercourse
☐ Vaginal bleeding related to intercourse
☐ Genital itchiness
☐ Any skin abnormalities
☐ None

When did these symptoms start for abnormal vaginal discharge?

◯ After previous visit
◯ Persistent since previous visit
◯ Recurrent since previous visit

For peer review only

When did these symptoms start for pain during urination?

◯ After previous visit
◯ Persistent since previous visit
◯ Recurrent since previous visit

When did these symptoms start for the lower abdominal pain?

◯ After previous visit
◯ Persistent since previous visit
◯ Recurrent since previous visit

When did these symptoms start for the pain related to intercourse?

◯ After previous visit
◯ Persistent since previous visit
◯ Recurrent since previous visit

When did these symptoms start for vaginal bleeding related to intercourse?

◯ After previous visit
◯ Persistent since previous visit
◯ Recurrent since previous visit

When did these symptoms start for genital itchiness?

◯ After previous visit
◯ Persistent since previous visit
◯ Recurrent since previous visit

Please specify any skin abnormalities

Baseline Treatment Date:
[baseline_arm_1][sti_treatment_date]

TOC Treatment Date:
[toc_arm_1_arm_1][toc_sti_treatment_date]

Did the participant receive any STI treatment at their last study visit?

◯ Yes
◯ No

Are you planning to wait for your results today?
(New question added @13/09/2022)

◯ Yes
◯ No

For peer review only

What is your main reason why you are not intending to wait today?
(New question added @13/09/2022)

◯ Have to get to work
◯ Have to get back to my kids/family
◯ Want to go to the shop
◯ Transport availability
◯ Lack of privacy
◯ Hungry
◯ No space to wait
◯ Not feeling well
◯ Boring
◯ Other

If "Other", please specify.
(New question added @13/09/2022)

What would make you change your mind?
(New question added @13/09/2022)

You are done with questions associated with clinical history review. You will now start with questions associated with Adherence.

◯ Proceed

Adherence

Did you finish the whole course of STI treatment

◯ Yes
◯ No

How many days did you take treatment for?

____

Did you throw up within 2 hours after taking any of the STI treatment

◯ Yes
◯ No

Did you take any other non-chronic treatment at the time

◯ Yes
◯ No

What type of treatment

For peer review only

You are done with questions associated with the adherence. You are about to start asking questions associated with disclosure.

◯ Proceed

Disclosure

Did you notify your partner of your STI result?

◯ Yes I gave him the notification slip
◯ Yes I told him
◯ No

What was his reaction when you told him of your STI infection

◯ Supportive
◯ Angry
◯ Violent
◯ Disengaged

How did disclosure affect your relationship?

◯ Continued as before
◯ Started using a condom
◯ He engaged with other partners
◯ He refused sex
◯ Relationship ended

Did he take treatment?

◯ Yes
◯ No
◯ I don't know

Where did he seek treatment

◯ Private
◯ Public
◯ Traditional

Why did you not notify your partner?

◯ I didn't feel it was necessary
◯ I am embarrassed
◯ I'm afraid he gets angry
◯ I'm afraid he gets violent
◯ I'm afraid he will end the relationship

Did you tell anyone else of your STI infection?

◯ Yes
◯ No

For peer review only

Who did you tell?

☐ Family member
☐ Friend
☐ HCW
☐ Other

Behavioral Questionnaire

NOTE TO RA:
You just completed all questions related to Disclosure. You are about to start with the Behavioral Questionnaire.

Please select "Proceed":

◯ Proceed

Did you have sex since the last visit?

◯ Yes
◯ No

How many different male partners did you have sexual intercourse with in the past month?

◯ 1
◯ 2
◯ more than 2

Were any of these new partners than the ones from the last visit

◯ Yes
◯ No

What type of sex did you have with partner 1 (Husband/ Steady partner)?

☐ Vaginal
☐ Anal
☐ Oral

Did you use a condom the last time you had sex with partner 1 (Husband/ Steady partner)?

◯ Yes
◯ No

What type of sex did you have with partner 2?

☐ Vaginal
☐ Anal
☐ Oral

Did you use a condom the last time you had sex with partner 2?

◯ Yes
◯ No

For peer review only

What type of sex did you have with the rest of the partners?

☐ Vaginal
☐ Anal
☐ Oral

Did you use a condom the last time you had sex with one of them?

◯ Yes
◯ No

Where are you planning to deliver?

◯ Frere
◯ CMH
◯ Nontyantyambo
◯ Empilweni
◯ Bisho
◯ Other

Please specify

You are done with the questions associated with Behavioral Questionnaire. You will now start asking questions associated with the Physical Examination.

◯ Proceed

Physical Examination

Weight of mother

____

Systolic blood pressure

____

Diastolic blood pressure

____

How was Hemoglobin measured?

◯ Hb meter at the clinic
◯ Hb at NHLS

Please capture Hb result"

____

For peer review only - http://bmjopen.bmj.com/site/about/guidelines.xhtml

Please capture the barcode for Hb

____

Fundal height

____

Progression of pregnancy

◯ Progressing normal
◯ Abnormality detected

Provide further details of abnormality

During your examination, were there any signs of abnormal vaginal discharge?

◯ Yes
◯ No

During your examination, were there any signs of inguinal lymphadenopathy?

◯ Yes
◯ No

Are these bubo?

◯ Yes
◯ No

Note to RA: Please contact the study clinician and specify treatment given to the participant

During your examination, were there any signs of lower abdominal pain?

◯ Yes
◯ No

During your examination, where there any signs of scratch marks?

◯ Yes
◯ No

During your examination, were there any signs of skin conditions?

◯ Yes
◯ No

Please specify the nature of the skin conditions

For peer review only - http://bmjopen.bmj.com/site/about/guidelines.xhtml

During your examination, were there any other observations that need to be noted?

You have completed the questions associated with the Physical Examination. You will now start capturing the results from the rapid tests.

◯ Proceed

Rapid Test Results

Do you know your current HIV status?

◯ HIV negative (tested today by clinical staff)
◯ HIV positive on ART
◯ HIV positive, not on ART
◯ Don't know (never tested)
◯ Don't know (no yet tested today)

Was the participant newly diagnosed with HIV today

◯ Yes
◯ No

Please conduct an HIV Rapid test and capture the result below

◯ Positive
◯ Negative

Please conduct a confirmatory HIV Rapid test and capture the result below

◯ Positive
◯ Negative

Did you collect a tube of blood for CD4 count?

◯ Yes
◯ No

Please record barcode for blood tube for CD4 count testing?

____

Is the participant's most recent CD4 count since Baseline available?

◯ Yes
◯ No

What was the date of the CD4 specimen collection?

____

For peer review only

What was the participant's most recent CD4 count?

____
(if no number listed, enter 9999)

Did you collect a tube of blood for viral load?

◯ Yes
◯ No

Please record barcode for blood tube for viral load testing?

____

Is the participant's most recent viral load since Baseline available?

◯ Yes
◯ No

What was the date of the viral load specimen collection?

____

What was the participant's most recent viral load?

____
(if no number listed, enter 0000)

Which regimen for ART were you started on today?

◯ TLD
◯ TEE
◯ AZT/3TC/LPV
◯ Other

Which regimen for ART were you on so far?

◯ TLD
◯ TEE
◯ AZT/3TC/LPV
◯ Other

Has the regimen for ART been changed today?

◯ Yes
◯ No

To which regimen for ART has it been changed today?

◯ TLD
◯ TEE
◯ AZT/3TC/LPV
◯ Other

For peer review only

Has a syphilis test been done for the participant?

◯ Yes
◯ No

Which syphilis test have you used?

◯ Alere Syphilis TP (provided by FPD)
◯ HIV/Syphilis Duo (provided by FPD)
◯ No rapid test used only NHLS bloods for RPR
◯ Other please specify

Please specify

Syphilis result.

◯ Positive
◯ Negative
◯ Indeterminate

Titer value 1:

____
(If RPR is non-reactive, enter 0)

Blood needs to be collected for further syphilis testing. Please confirm if blood was collected.

◯ Yes
◯ No

Collect blood for RPR and capture barcode PIN below

____

Treatment given

◯ Benzathine penicillin 2.4 MU IM weekly x3
◯ Out of stock

Please contact study clinician and specify treatment given

Please collect participant's urine for testing

For peer review only

[illegible]

You are about to capture the results from the dipstick testing

Please select "Proceed" to continue

◯ Proceed

Blood - Hemoglobin

◯ Negative
◯ Ca. 10
◯ Ca. 50
◯ Ca. 250/300

Blood - Erythrocytes

◯ Negative
◯ Ca. 5 -10
◯ Ca. 50
◯ Ca. 250/300

Urobilinogen

◯ Normal
◯ 2
◯ 4
◯ 8
◯ 12

Bilirubin

◯ Negative
◯ 1 plus
◯ 2 plus
◯ 3 plus
◯ Not available

Protein

◯ Negative
◯ 30
◯ 100
◯ 500

Nitrate

◯ Negative
◯ Positive

For peer review only

Keton

◯ Negative
◯ 1 plus
◯ 2 plus
◯ 3 plus
◯ Not available

Glucose

◯ Negative
◯ Normal
◯ 50
◯ 150
◯ 500
◯ ≥1000

pH

◯ 5
◯ 6
◯ 7
◯ 8
◯ 9
◯ Not available

SG

◯ 1.000
◯ 1.005
◯ 1.010
◯ 1.015
◯ 1.020
◯ 1.025
◯ 1.030
◯ Not available

Leucocytes

◯ Negative
◯ 25
◯ 75
◯ 500

STI Results and Screening

For peer review only

The participant is in arm 2 or for Empilweni and therefore you are about to capture results for the STI testing.

◯ Proceed

STI Test Outcome

The previous STI results of the participants are:

Baseline
NG:[baseline_arm_1][sti_result_ng]

CT:[baseline_arm_1][sti_result_ct]

TV:[baseline_arm_1][sti_result_tv]

TOC
NG:[toc_arm_1_arm_1][toc_ng]

CT:[toc_arm_1_arm_1][toc_ct]

TV:[toc_arm_1_arm_1][toc_tv]

	Negative	Positive
CT	◯	◯
NG	◯	◯
TV	◯	◯

STI Calculation _ w32

____

Date the result was obtained.

____

Did the participant wait for her STI results?
(New question added @ 03/11/2022)

◯ Yes
◯ No

Was the participant reporting STI symptoms or showed symptoms during the clinical assessment?

◯ Yes
◯ No

Does the participant report any medication allergies?

◯ Yes
◯ No

For peer review only

Please contact the study clinician before giving any treatment. Please specify discussed medication allergies and treatment plan with the study clinician

The following treatment has been provided

☐ Azithromycin 1g stat dose
☐ Azithromycin 2g stat dose
☐ Ceftriaxone 250mg IM injection
☐ Ceftriaxone 1g IM injection
☐ Metronidazole 400mg bd x 1 week
☐ Metronidazole 2g stat dose
☐ Clotrimazole pessary and/or cream
☐ Trimethoprim/sulfamethoxazole 400/80 mg 2 tbl. bds for 5 days (bactrim)
☐ Ceftriaxone 500mg IM injection

Date treatment given

____

What made you change your mind about waiting for the results?
(New question added @13/09/2022)

Partner notification provided

◯ Yes, 1
◯ Yes, multiple
◯ No

Please explain why the partner notification note was not provided?

You have completed capturing the information from the 32 week exam. Please make sure to check that you have completed all the fields.

Please select "Complete" then "Save and Exit".

Notes

Additional notes

For peer review only

32[illegible] [illegible]

You are about to capture the results of specimens collected during the 32-week visit

Please select "Proceed" to continue

◯ Proceed

Hb Results received

◯ Yes
◯ No

Please capture the Hb result

____

Please capture the barcode for Hb

____

Please specify whether the reading was satisfactory for the loop used for Nugent score

◯ Yes
◯ No

____

Please capture the score for the loop used for Nugent scoring

____

Please specify if candida was present for the loop used for Nugent scoring

◯ Yes
◯ No

Please specify whether the reading was satisfactory for the loop used for yeast microscopy

◯ Yes
◯ No

____

Please capture the nugent score for the loop used for yeast microscopy

____

For peer review only

Please specify if candida was present for the loop used for yeast microscopy

◯ Yes
◯ No

Please capture the results for the blood used for Syphilis testing

◯ RPR Negative
◯ RPR Positive
◯ RPR Indeterminate

____

Please capture the result for viral load testing

____

Is the participant's most recent viral load available?

◯ Yes
◯ No

What was the date of the viral load specimen collection?

____

What was the participant's most recent viral load?

____

____

Notes

Notes

For peer review only

□a□□□□g Re□□□□e□□_2

Staff Name ______________________________

Today's Date ______________________________

Time ______________________________

The presentation dates are below:

TOC
Date: [baseline_arm_1][toc_3week]

WEEK 28 CALLING

Date: [baseline_arm_1][sched_28w_rem]

WEEK-32

30-34 Week Actual Date: [week_28_arm_1][week32_visit_date]

Call In Check

Week 37 Call In Check: [week_28_arm_1][cic_date]

POST-NATAL VISIT

Visit open date: [predelivery_checki_arm_1][pd_remind_date_schedpd]

Visit close date: [predelivery_checki_arm_1][pd_close_date_schedpd]

Date of Delivery: [predelivery_checki_arm_1][pd_remind_date_delivery]

6-WEEK IMMUNIZATION VISIT

Actual Date: [predelivery_checki_arm_1][sixw_im_schedpd]

NOTE:
You are about to call a participant to remind them of a specific visit. Please make sure to do the following:

1. Obtain all relevant contact numbers for the participant on their record
2. Ensure that you have checked what the exact date is when the participant is expected to present
3. Make sure to give the participant a brief description of what will be done at the visit.
4. You will make up to 3 attempts to get hold of the participant.

Please select "Proceed"

◯ Proceed

For peer review only

For peer review only - http://bmjopen.bmj.com/site/about/guidelines.xhtml

Please select the calling attempt

☐ First Attempt
☐ Second Attempt
☐ Third Attempt

Details of Calling Attempt 1

Outcome of the attempt

◯ Successful - Participant
◯ Successful - Family member
◯ Unsuccessful - Voicemail
◯ Unsuccessful - Invalid

Date of the attempt

____

Did she deliver?

◯ Yes
◯ No

Capture delivery Date

NOTE: instruct to come to the site.

____

Details of Calling Attempt 2

Outcome of the attempt

◯ Successful - Participant
◯ Successful - Family member
◯ Unsuccessful - Voicemail
◯ Unsuccessful - Invalid

Date of the attempt

____

For peer review only

Did she deliver?

◯ Yes
◯ No

Capture delivery Date

NOTE: instruct to come to the site.

____

Details of Calling Attempt 3

Outcome of the attempt

◯ Successful - Participant
◯ Successful - Family member
◯ Unsuccessful - Voicemail
◯ Unsuccessful - Invalid

Date of the attempt

____

Did she deliver?

◯ Yes
◯ No

Capture delivery Date

NOTE: instruct to come to the site.

____

Notes

Calling notes

For peer review only - http://bmjopen.bmj.com/site/about/guidelines.xhtml

S[illegible]e[illegible]e [illegible]a[illegible]g [illegible] 38 [illegible]ee[illegible]

NOTE FW: Please make sure that you call the participant once per week.

◯ Proceed

Did you schedule the 38 weeks call

◯ Yes
◯ No

Please select the calling attempt

☐ First Attempt
☐ Second Attempt
☐ Third Attempt

Details of Calling Attempt 1

Outcome of the attempt

◯ Successful - Participant
◯ Successful - Family member
◯ Unsuccessful - Voicemail
◯ Unsuccessful - Invalid

Date of the attempt

____

Did she deliver?

◯ Yes
◯ No

Details of Calling Attempt 2

Outcome of the attempt

◯ Successful - Participant
◯ Successful - Family member
◯ Unsuccessful - Voicemail
◯ Unsuccessful - Invalid

Date of the attempt

____

For peer review only

Did she deliver?

◯ Yes
◯ No

Details of Calling Attempt 3

Outcome of the attempt

◯ Successful - Participant
◯ Successful - Family member
◯ Unsuccessful - Voicemail
◯ Unsuccessful - Invalid

Date of the attempt

____

Did she deliver?

◯ Yes
◯ No

Schedule calling for 39 weeks

NOTE FW: Please make sure that you call the participant once per week.

◯ Proceed

Did you schedule the 39 weeks call

◯ Yes
◯ No

Please select the calling attempt

☐ First Attempt
☐ Second Attempt
☐ Third Attempt

For peer review only

[illegible]e[illegible]a[illegible] [illegible]a[illegible]g [illegible]e[illegible] 1

Outcome of the attempt

◯ Successful - Participant
◯ Successful - Family member
◯ Unsuccessful - Voicemail
◯ Unsuccessful - Invalid

Date of the attempt

____

Did she deliver?

◯ Yes
◯ No

Capture delivery Date

NOTE: instruct to come to the site.

____

For peer review only

Details of Calling Attempt 2

Outcome of the attempt

◯ Successful - Participant
◯ Successful - Family member
◯ Unsuccessful - Voicemail
◯ Unsuccessful - Invalid

Date of the attempt

____

Did she deliver?

◯ Yes
◯ No

Details of Calling Attempt 3

Outcome of the attempt

◯ Successful - Participant
◯ Successful - Family member
◯ Unsuccessful - Voicemail
◯ Unsuccessful - Invalid

Date of the attempt

____

Did she deliver?

◯ Yes
◯ No

Calling notes

Schedule calling for 40 weeks

NOTE FW: Please make sure that you call the participant once per week.

◯ Proceed

Please select the calling attempt

☐ First Attempt
☐ Second Attempt
☐ Third Attempt

Did you schedule a call for 40 weeks

◯ Yes
◯ No

Details of Calling Attempt 1

Outcome of the attempt

◯ Successful - Participant
◯ Successful - Family member
◯ Unsuccessful - Voicemail
◯ Unsuccessful - Invalid

Date of the attempt

____

Did she deliver?

◯ Yes
◯ No

Capture delivery Date

NOTE: instruct to come to the site.

____

For peer review only

□e□a□□□ □□□a□□□□g □□□e□□□ 2

Outcome of the attempt

◯ Successful - Participant
◯ Successful - Family member
◯ Unsuccessful - Voicemail
◯ Unsuccessful - Invalid

Date of the attempt

____

Did she deliver?

◯ Yes
◯ No

Details of Calling Attempt 3

Outcome of the attempt

◯ Successful - Participant
◯ Successful - Family member
◯ Unsuccessful - Voicemail
◯ Unsuccessful - Invalid

Date of the attempt

____

Calling notes

Did she deliver?

◯ Yes
◯ No

Schedule calling for 41 weeks

NOTE FW: Please make sure that you call the participant once per week.

◯ Proceed

Please select the calling attempt

☐ First Attempt
☐ Second Attempt
☐ Third Attempt

For peer review only

Did you schedule a call for 41 weeks

◯ Yes
◯ No

Details of Calling Attempt 1

Outcome of the attempt

◯ Successful - Participant
◯ Successful - Family member
◯ Unsuccessful - Voicemail
◯ Unsuccessful - Invalid

Date of the attempt

____

Details of Calling Attempt 2

Outcome of the attempt

◯ Successful - Participant
◯ Successful - Family member
◯ Unsuccessful - Voicemail
◯ Unsuccessful - Invalid

Date of the attempt

____

Did she deliver?

◯ Yes
◯ No

Details of Calling Attempt 3

Outcome of the attempt

◯ Successful - Participant
◯ Successful - Family member
◯ Unsuccessful - Voicemail
◯ Unsuccessful - Invalid

Date of the attempt

____

Calling notes

For peer review only

Did she deliver?

◯ Yes
◯ No

Capture delivery Date

NOTE: instruct to come to the site.

____

Schedule calling for 41 weeks (293 days)

NOTE FW: Please make sure that you call the participant once per week.

◯ Proceed

Did you schedule a call for 41 weeks

◯ Yes
◯ No

Please select the calling attempt

☐ First Attempt
☐ Second Attempt
☐ Third Attempt

Details of Calling Attempt 1

Outcome of the attempt

◯ Successful - Participant
◯ Successful - Family member
◯ Unsuccessful - Voicemail
◯ Unsuccessful - Invalid

Calling notes

Did she deliver?

◯ Yes
◯ No

Capture delivery Date

NOTE: instruct to come to the site.

____

For peer review only

S[illegible]e[illegible]e [illegible]a[illegible]g [illegible] 42 [illegible]ee[illegible] [illegible]296 [illegible]a[illegible]

NOTE FW: Please make sure that you call the participant once per week.

◯ Proceed

Please select the calling attempt

☐ First Attempt
☐ Second Attempt
☐ Third Attempt

Did you schedule a call for 42 weeks

◯ Yes
◯ No

Details of Calling Attempt 1

Outcome of the attempt

◯ Successful - Participant
◯ Successful - Family member
◯ Unsuccessful - Voicemail
◯ Unsuccessful - Invalid

Date of the attempt

____

Did she deliver?

◯ Yes
◯ No

Details of Calling Attempt 2

Outcome of the attempt

◯ Successful - Participant
◯ Successful - Family member
◯ Unsuccessful - Voicemail
◯ Unsuccessful - Invalid

Date of the attempt

____

For peer review

Did she deliver?

◯ Yes
◯ No

Details of Calling Attempt 3

Outcome of the attempt

◯ Successful - Participant
◯ Successful - Family member
◯ Unsuccessful - Voicemail
◯ Unsuccessful - Invalid

Date of the attempt

____

Outcome of the call

Did the participant deliver?

◯ Yes
◯ No

Calling notes

Did she deliver?

◯ Yes
◯ No

Capture delivery Date

NOTE: instruct to come to the site.

____

For peer review only

[illegible]

You are about the schedule dates associated with the following events:

1. Post-Delivery Appointment
2. 6-Weeks Immunization Appointment

◯ Proceed

Post-Delivery Study Visit

You will now schedule dates associated with the post-delivery study visit.

The following dates are associated with this visit:
1. Calling reminder date / Visit opening date
2. Visit closing date

Select "Proceed" to continue

◯ Proceed

Please capture the date of delivery

____

Please schedule the date for the post-delivery reminder call.

Please note that the Delivery date is [pd_remind_date_delivery]. The reminder call will happen 1 day following delivery.

____

Calculation Assist for Post Delivery reminder call

This number must equal to: 1

____

Match

____

NOTE
The date you have entered is incorrect. Please make sure that the numbers correspond.

For peer review only

The post-delivery closing date.

____

Calculation Assist for Post Delivery closing date

The participant will have 14 days post-delivery to present at the clinic. The delivery date was [pd_remind_date_delivery]. This number must therefore equal to: 14

____

Match

____

NOTE
The date you have entered is incorrect. Please make sure that the numbers correspond.

Facility delivered

- ◯ Frere
- ◯ CMH
- ◯ Nontyantyambo
- ◯ Empilweni
- ◯ Bisho
- ◯ Other

Please specify the facility of delivery

____

6-Week Immunization Visit

NOTE:
In this section you will schedule all dates associated with the 6-Week Immunization Study Visit. These dates will include:

1. Calling reminder date for 6-Weeks Immunization visit
2. Scheduled date of 6-Weeks Immunization visit
3. Closing date for attending the 6-Weeks Immunization visit

Select Proceed to continue

◯ Proceed

Please schedule the date for the 6-weeks immunization reminder

____

For peer review only - http://bmjopen.bmj.com/site/about/guidelines.xhtml

Calculation Assist for 6-weeks immunization reminder. We will call all patients 5 weeks (35 days) following their delivery date. The updated delivery date for the participant was [calling_delivery_date_37weeks].

This number must therefore equal to: 35 and 40

____

Match

____

NOTE
The date you have entered is incorrect. Please make sure that the numbers correspond.

Please schedule the date for the 6-weeks immunization visit. This visit is scheduled to take place 6 weeks (42 days) following delivery. The updated delivery date is [calling_delivery_date_37weeks]

____

Calculation Assist for 6-weeks immunization visit

This number must equal to: 42

____

Match

____

NOTE
The date you have entered is incorrect. Please make sure that the numbers correspond.

Please schedule the date for the 6-weeks immunization visit closing date.

____

Calculation Assist for 6-Weeks Immunization visit Close Date. Mothers will have up to 8 weeks post delivery to attend this visit. This means 56 days following the delivery.

This number must equal to: 56

____

Match

____

For peer review only

NOTE
The date you have entered is incorrect. Please make sure that the numbers correspond.

CONGRATULATIONS

You have finished scheduling all dates.

For peer review only

[illegible] Reg[illegible]e[illegible] [illegible]a[illegible]a

Participant PIN

[baseline_arm_1][participant_pin]

Staff name

____

Today's date

____

Start time

____

You are about to capture data retrieved from the birth registry. Please select "Proceed" to start

◯ Proceed

Delivery Details

Delivery site

◯ Frere
◯ CMH
◯ Nontyantyambo
◯ Empilweni
◯ Bisho
◯ Other

Please specify name of delivery facility

Clinic file number

____

Delivery date

____

Calculated gestational age

____
(Added @22/03/2023)

For peer review only

Please specify the number of babies during pregnancy

◯ 1
◯ 2
◯ 3

Outcome type for baby 1

◯ Live birth
◯ Still birth
◯ Early Neonatal Death

Outcome type for baby 2

◯ Live birth
◯ Still birth
◯ Early Neonatal Death

Outcome type for baby 3

◯ Live birth
◯ Still birth
◯ Early Neonatal Death

Type of delivery for baby 1

◯ Born before arrival
◯ Normal Vaginal Delivery
◯ Assisted Vaginal Delivery
◯ Elective Cesarean Section
◯ Emergency Cesarean Section

Type of delivery for baby 2

◯ Born before arrival
◯ Normal Vaginal Delivery
◯ Assisted Vaginal Delivery
◯ Elective Cesarean Section
◯ Emergency Cesarean Section

Type of delivery for baby 3

◯ Born before arrival
◯ Normal Vaginal Delivery
◯ Assisted Vaginal Delivery
◯ Elective Cesarean Section
◯ Emergency Cesarean Section

Please specify reason

Please specify reason

For peer review only

For peer review only - http://bmjopen.bmj.com/site/about/guidelines.xhtml

Please specify reason

Gender - Baby 1

◯ Female
◯ Male

Gender - Baby 2

◯ Female
◯ Male

Gender - Baby 3

◯ Female
◯ Male

Complications in labor/Delivery

	Yes	No
Induction of labour	◯	◯
Antepartun haemorrhage	◯	◯
Post Partum haemorrhage	◯	◯
Severe pre-eclampsia	◯	◯
Eclampsia	◯	◯
Prolonged rupture of membranes	◯	◯
Ruptured uterus	◯	◯
Sepsis	◯	◯
Obstructed or prolonged labour	◯	◯
Retained Placenta	◯	◯
Manual removal of placenta	◯	◯

Maternal outcome

◯ Live
◯ Death

APGAR score at 5 minutes for baby 1

____

APGAR score at 5 minutes for baby 2

____

APGAR score at 5 minutes for baby 3

____

For peer review only

Birth weight for baby 1 in grams

____

Birth weight for baby 2 in grams

____

Birth weight for baby 3 in grams

____

Did you breastfeed your baby/ies within 1 hour of giving birth?

◯ Yes
◯ No

Infant feeding
(New question added @15/11/2022)

◯ Exclusive Breast Feeding (EBF)
◯ Exclusive Formula Feeding (EFF)

Any birth defects to note for baby 1

◯ Yes
◯ No

Any birth defects to note for baby 2

◯ Yes
◯ No

Any birth defects to note for baby 3

◯ Yes
◯ No

Please specify

Remarks outcome

Maternal outcome

For peer review only

You have completed the Birth register. Please make sure to check if all relevant fields have been selected and the information captured is accurate.

Once this is done, please select the "Complete" option below and then select "Save & Exit".

For peer review only

[illegible]

Staff name ________________________

Today's date ________________________

Start time ________________________

Post-Natal Visit

The participant was scheduled to present within the two dates below. Please specify if the participant presented within this timeframe.

Visit open date: [predelivery_checki_arm_1][pd_remind_date_delivery]

Visit close date: [predelivery_checki_arm_1][pd_close_date_schedpd]

◯ Yes
◯ No

You are about to administer the questions associated with the post natal visit.

Please select "Proceed"

◯ Proceed?

Clinical History Review

Have you been to the clinic since the last visit with us?

◯ Yes
◯ No

What was the purpose of your visit?

☐ ANC Visit
☐ HIV/ART
☐ STI Treatment
☐ Other

Summary notes from the visit

For peer review only

Were there any abnormalities/complications since your last study visit regarding your pregnancy and delivery or did you receive any non-chronic treatment?

◯ Yes
◯ No

Please specify

Have you used any of the following since the first study visit?
Select multiple

☐ Alcohol
☐ Tik
☐ Dagga
☐ Grandpa
☐ Other
☐ None

Please specify other drugs used?

The Baseline STI results of the participants are:

NG: [baseline_arm_1][sti_result_ng]

CT: [baseline_arm_1][sti_result_ct]

TV: [baseline_arm_1][sti_result_tv]

The TOC STI results of the participants are:

NG: [toc_arm_1_arm_1][toc_ng]

CT: [toc_arm_1_arm_1][toc_ct]

TV: [toc_arm_1_arm_1][toc_tv]

The Week 32 STI results of the participants are:

CT: [3034_weeks_arm_1][w32_ct_res]

NG: [3034_weeks_arm_1][w32_ng_res]

TV: [3034_weeks_arm_1][w32_tv_res]

Did the participant receive any STI treatment at their last study visit?

◯ Yes
◯ No

For peer review only

For peer review only - http://bmjopen.bmj.com/site/about/guidelines.xhtml

[illegible]

You are done with questions associated with the clinical history review. You will now start with questions associated with Adherence.

◯ Proceed

Did you finish the whole course of STI treatment?

◯ Yes
◯ No

How many days did you take treatment for?

____

Did you throw up within 2 hours after taking any of the STI treatment?

◯ Yes
◯ No

Did you take any other non-chronic treatment at the time?

◯ Yes
◯ No

What type of treatment

Disclosure

You are done with questions associated with the adherence. You are about to start asking questions associated with disclosure.

◯ Proceed

Did you notify your partner of your STI result?

◯ Yes I gave him the notification slip
◯ Yes I told him
◯ No

What was his reaction when you told him of your STI infection

◯ Supportive
◯ Angry
◯ Violent
◯ Disengaged

For peer review only

How did disclosure affect your relationship?

◯ Continued as before
◯ Started using a condom
◯ He engaged with other partners
◯ He refused sex
◯ Relationship ended

Did he take treatment?

◯ Yes
◯ No
◯ I don't know

Where did he seek treatment?

◯ Private
◯ Public
◯ Traditional

Why did you not notify your partner?

◯ I didn't feel it was necessary
◯ I am embarrassed
◯ I'm afraid he gets angry
◯ I'm afraid he gets violent
◯ I'm afraid he will end the relationship

Did you tell anyone else of your STI infection?

◯ Yes
◯ No

Did you tell anyone else of your STI infection?

◯ Yes
◯ No

Who did you tell?

☐ Family member
☐ Friend
☐ HCW
☐ Other

Delivery Details of Infant

Facility delivered

◯ Frere
◯ CMH
◯ Nontyantyambo
◯ Empilweni
◯ Bisho
◯ Other

Please specify facility of delivery

For peer review only

Date of delivery

____

Calculated gestational age

____
(Added @22/03/2023)

Please specify the number of babies during pregnancy

◯ 1
◯ 2
◯ 3

Outcome type for baby 1

◯ Live birth
◯ Still birth
◯ Early Neonatal Death

Outcome type for baby 2

◯ Live birth
◯ Still birth
◯ Early Neonatal Death

Outcome type for baby 3

◯ Live birth
◯ Still birth
◯ Early Neonatal Death

Type of delivery for baby 1

◯ Born before arrival
◯ Normal Vaginal Delivery
◯ Assisted Vaginal Delivery
◯ Elective Cesarean Section
◯ Emergency Cesarean Section

Type of delivery for baby 2

◯ Born before arrival
◯ Normal Vaginal Delivery
◯ Assisted Vaginal Delivery
◯ Elective Cesarean Section
◯ Emergency Cesarean Section

Type of delivery for baby 3

◯ Born before arrival
◯ Normal Vaginal Delivery
◯ Assisted Vaginal Delivery
◯ Elective Cesarean Section
◯ Emergency Cesarean Section

Please specify reason

For peer review only

Please specify reason

Please specify reason

Gender - Baby 1

◯ Female
◯ Male

Gender - Baby 2

◯ Female
◯ Male

Gender - Baby 3

◯ Female
◯ Male

Maternal outcome

◯ Live
◯ Death

Specify

APGAR score at 5 minutes for baby 1

Note to RA: Check on Road to Health

____
(if no number listed, enter 99)

APGAR score at 5 minutes for baby 2
Note to RA: Check on Road to Health

____
(if no number listed, enter 99)

APGAR score at 5 minutes for baby 3
Note to RA: Check on Road to Health

____
(if no number listed, enter 99)

For peer review only

Birth weight in grams for baby 1

Note to RA: Check on Road to Health

____

Birth weight in grams for baby 2

Note to RA: Check on Road to Health

____

Birth Weight in grams for baby 3

Note to RA: Check on Road to Health

____

Newborn problems

Note to RA: Check on Road to Health

☐ Birth defects
☐ Hypoxic brain injury
☐ Convulsions /fits
☐ Jaundice
☐ None

Please Specify

Was the baby exposed to HIV?
(Added @29/03/2023)

◯ Yes
◯ No

Was Nevirapine given to the baby/babies

◯ Yes
◯ No

Was birth PCR done for the baby/babies

◯ Yes
◯ No

NOTE: If not taken by birth facility please take blood for PCR.

PCR Barcode for the baby/baby 1

____

For peer review only

NOTE: If not taken by birth facility please take blood for PCR.

PCR Barcode for the baby/baby 2

____

NOTE: If not taken by birth facility please take blood for PCR.

PCR Barcode for the baby/baby 3

____

Result of birth PCR for baby 1

◯ Positive
◯ Negative
◯ Indeterminate
◯ Not yet available

Result of birth PCR for baby 2

◯ Positive
◯ Negative
◯ Indeterminate
◯ Not yet available

Result of birth PCR for baby 3

◯ Positive
◯ Negative
◯ Indeterminate
◯ Not yet available

Call clinician and make a note about this.

Was eye ointment given to the baby 1

◯ Yes
◯ No
◯ Don't know

Was eye ointment given to the baby 2

◯ Yes
◯ No
◯ Don't know

Was eye ointment given to the baby 3

◯ Yes
◯ No
◯ Don't know

Please specify to how many babies and which one

For peer review only

Was the baby/babies admitted to hospital following delivery

◯ Yes
◯ No

Please specify the details about the reason for admission, number of babies admitted and which babies

Does baby 1 have any of the following symptoms?

☐ Cough
☐ Runny nose
☐ Eye discharge
☐ Sneezing
☐ None

Does baby 2 have any of the following symptoms?

☐ Cough
☐ Runny nose
☐ Eye discharge
☐ Sneezing
☐ None

Does baby 3 have any of the following symptoms

☐ Cough
☐ Runny nose
☐ Eye discharge
☐ Sneezing
☐ None

Is the baby/babies receiving any treatment at the moment

◯ Yes
◯ No

Please specify

Feeding methods

◯ Breastfeeding
◯ Formula feeding
◯ Mixed

For peer review only

[illegible]

Is the baby present with the biological mother

◯ Yes
◯ No

Please specify

Are you currently taking any treatment

◯ Yes
◯ No

Please specify

Have you had sexual intercourse since delivery of your baby?

◯ Yes
◯ No

Do you have any of the following symptoms?

☐ Discharge
☐ Pain when urinating
☐ None

Please specify

You have completed all the questions associated with this visit. You will now start with the process of specimen collection. You will need to collect the following specimens:

From the mother you will need to collect 3 vaginal swabs:
- Vaginal Swab 1 for STI testing (Storage)
- Vaginal Swab 2 for Microbiome (Storage)
- Vaginal Swab 3 for Profiling (Storage)

From the baby you need to collect:
- Nasopharyngeal swab

- Conjunctival

Select "Proceed" to capture the information associated with these specimens

◯ Proceed

For peer review only
For peer review only - http://bmjopen.bmj.com/site/about/guidelines.xhtml

Please confirm the barcode for the vaginal swab collected for STI testing

◯ [baseline_arm_1][participant_pin]-PNV1

Please confirm the barcode for the vaginal swab collected for Microbiome

◯ [baseline_arm_1][participant_pin]-PNV3

Please confirm the Barcode for the Vaginal Swab collected for Profiling

◯ [baseline_arm_1][participant_pin]-PNV2

Please confirm the Barcode for the Nasopharyngeal swab (right nose) baby 1

◯ [baseline_arm_1][participant_pin]-PNB1N1

Please confirm the Barcode for the Nasopharyngeal swab (left nose) baby 1

◯ [baseline_arm_1][participant_pin]-PNB1N2

Please confirm the barcode for the Nasopharyngeal swab (right nose) baby 2

◯ [baseline_arm_1][participant_pin]-PNB2N1

Please confirm the barcode for the Nasopharyngeal swab (left nose) baby 2

◯ [baseline_arm_1][participant_pin]-PNB2N2

Please confirm the barcode for the Nasopharyngeal swab (right nose) baby 3

◯ [baseline_arm_1][participant_pin]-PNB3N1

Please confirm the barcode for the Nasopharyngeal swab (left nose) baby 3

◯ [baseline_arm_1][participant_pin]-PNB3N2

Please confirm the Barcode for the Nasopharyngeal swab for STI testing baby 1

◯ [baseline_arm_1][participant_pin]-PNB1N1

Please confirm the barcode for the Conjunctival swab (right eye) baby 1

◯ [baseline_arm_1][participant_pin]-PNB1C1

Please confirm the barcode for the Conjunctival swab (left eye) baby 1

◯ [baseline_arm_1][participant_pin]-PNB1C2

Please confirm the barcode for the Conjunctival swab (right eye) baby 2

◯ [baseline_arm_1][participant_pin]-PNB2C1

Please confirm the barcode for the Conjunctival swab (left eye) baby 2

◯ [baseline_arm_1][participant_pin]-PNB2C2

For peer review only

Please confirm the barcode for the Conjunctival swab (right eye) baby 3

◯ [baseline_arm_1][participant_pin]-PNB3C1

Please confirm the barcode for the Conjunctival swab (left eye) baby 3

◯ [baseline_arm_1][participant_pin]-PNB3C2

Please specify the vaginal pH

____

Please select which pH strips are used to measure vaginal pH

◯ CardinalHealth pH Indicator Strips (range 3.6-6.1)
◯ pH Indicator Strips pH 0-14
◯ Natureland vaginal pH test (range 3.5-6.5)

Did you give the participant the study voucher?

◯ Yes
◯ No

You have completed capturing the Post-Natal information. Please make sure to check that you have completed all the fields.

Please give the participant 6 weeks immunization visit date as per schedule.

Please select "Complete" then "Save and Exit".

Notes

Additional notes

For peer review only

[illegible]

Date

____

Staff name

____

You are about to capture the results of the specimens collected during the post natal visit

Please select "Proceed" to continue

◯ Proceed

Receive Date ____________________________

Test date (Mother) ____________________________

STI results from the mother

	Positive	Negative
CT	◯	◯
NG	◯	◯
TV	◯	◯

STI result, mother_ calc

____

Test date (Baby) ____________________________

STI Results from Baby 1

	Positive	Negative
CT (Right Nose)	◯	◯
NG (Right Nose)	◯	◯
TV (Right Nose)	◯	◯
CT (Left Nose)	◯	◯
NG (Left Nose)	◯	◯
TV (Left Nose)	◯	◯

For peer review only

CT (Right Eye)	◯	◯
NG (Right Eye)	◯	◯
TV (Right Eye)	◯	◯
CT (Left Eye)	◯	◯
NG (Left Eye)	◯	◯
TV (Left Eye)	◯	◯

STI Results from Baby 2

	Positive	Negative
CT - Right Nose	◯	◯
NG - Right Nose	◯	◯
TV - Right Nose	◯	◯
CT - Left Nose	◯	◯
NG Left Nose	◯	◯
TV - Left Nose	◯	◯
CT - Right Eye	◯	◯
NG - Right Eye	◯	◯
TV - Right Eye	◯	◯
CT - Left Eye	◯	◯
NG - Left Eye	◯	◯
TV - Left Eye	◯	◯

STI Results from Baby 3

	Positive	Negative
CT - Right Nose	◯	◯
NG - Right Nose	◯	◯
TV - Right Nose	◯	◯
CT - Left Nose	◯	◯
NG - Left Nose	◯	◯
TV - Left Nose	◯	◯
CT - Right Eye	◯	◯
NG - Right Eye	◯	◯
TV - Right Eye	◯	◯
CT - Left Eye	◯	◯
NG - Left Eye	◯	◯
TV - Left Eye	◯	◯

Result of birth PCR for baby 1

◯ Positive
◯ Negative
◯ Indeterminate
◯ Not yet available

For peer review only

Result of birth PCR for baby 2

- ◯ Positive
- ◯ Negative
- ◯ Indeterminate
- ◯ Not yet available

Result of birth PCR for baby 3

- ◯ Positive
- ◯ Negative
- ◯ Indeterminate
- ◯ Not yet available

Notes

Notes

For peer review only

6[illegible] ee[illegible] I[illegible]a[illegible] [illegible] [illegible]e[illegible]

Staff name ______________________________

Today's date ______________________________

Time ______________________________

Did the mother present within the specified dates below:

Start Date: [predelivery_checki_arm_1][sixweek_remind_schedpd]

Actual Date: [predelivery_checki_arm_1][sixw_im_schedpd]

End Date: [predelivery_checki_arm_1][sixw_im_close_schedpd]

◯ Yes
◯ No

You are about to administer the questions associated with 6-weeks immunization visit.

Please select "Proceed"

◯ Proceed

How many babies were delivered?

◯ 1
◯ 2
◯ 3

Was baby 1 admitted to hospital since the last study visit

◯ Yes
◯ No

Was baby 2 admitted to hospital following delivery

◯ Yes
◯ No

Was baby 3 admitted to hospital following delivery

◯ Yes
◯ No

Please specify

For peer review only

Does baby 1 have any of the following symptoms?

☐ Cough
☐ Runny nose
☐ Eye discharge
☐ Sneezing
☐ None

Does baby 2 have any of the following symptoms?

☐ Cough
☐ Runny nose
☐ Eye discharge
☐ Sneezing
☐ None

Does baby 3 have any of the following symptoms?

☐ Cough
☐ Runny nose
☐ Eye discharge
☐ Sneezing
☐ None

Are any of the babies receiving any treatment at the moment

Feeding methods

☐ Breastfeeding
☐ Formula feeding
☐ Mixed

Have you or the baby been to the clinic since the last visit with us?

◯ Yes
◯ No

What was the purpose of your visit?

☐ ANC Visit
☐ HIV/ART
☐ STI Treatment
☐ Other

Summary notes from the visit

For peer review only

Do you know your current HIV status?

☐ HIV negative (tested today by clinical staff)
☐ HIV positive on ART
☐ Known HIV positive, not on ART
☐ Newly diagnosed HIV positive (tested today by clinical staff)
☐ Don't know (never tested)
☐ Don't know (no yet tested today)

Please conduct a HIV Rapid test and capture the result below

◯ Positive
◯ Negative

Please conduct a confirmatory HIV Rapid test and capture the result below

◯ Positive
◯ Negative

HIV PCR result of baby 1

◯ Positive
◯ Negative
◯ No result

Please record barcode for blood and HIV PCR

____

HIV PCR result of baby 2

◯ Positive
◯ Negative
◯ No result

Please record barcode for blood and HIV PCR

____

HIV PCR result of baby 3

◯ Positive
◯ Negative
◯ No result

Please record barcode for blood and HIV PCR

____

NOTE
You have collected all specimens associated with this visit. Once you select the "Proceed" option below you will be

CT: [post_natal_arm_1][sti_result_ct]

NG: [post_natal_arm_1][sti_result_ng]

TV: [post_natal_arm_1][sti_result_tv]

◯ Proceed

For peer review only

STI result, mother_ calc

____

Does the participant report any medication allergies?

◯ Yes
◯ No

Please contact the study clinician before giving any treatment. Please specify discussed medication allergies and treatment plan with the study clinician

The following treatment has been provided

☐ Azithromycin 1g stat dose
☐ Azithromycin 2g stat dose
☐ Ceftriaxone 250mg IM injection
☐ Ceftriaxone 1g IM injection
☐ Metronidazole 400mg bd x 1 week
☐ Metronidazole 2g stat dose
☐ Clotrimazole pessary and/or cream
☐ Trimethoprim/sulfamethoxazole 400/80 mg 2 tbl. bds for 5 days (bactrim)

Date treatment given

____

Partner notification provided

◯ Yes, 1
◯ Yes, multiple
◯ No

Please explain why the partner notification note was not provided?

The mother tested positive for an STI at the Post Natal visit. You need to collect a Nasal Pharyngeal swab for baby 1. Did you manage to collect this specimen?

◯ Yes
◯ No

Please confirm the PIN for the Nasal Pharyngeal swab for baby 1.

◯ [baseline_arm_1][participant_pin]-NPB1

For peer review only

The mother tested positive for an STI at the Post Natal visit. You need to collect a Nasal Pharyngeal swab for baby 2. Did you manage to collect this specimen?

◯ Yes
◯ No

Please confirm the PIN for the Nasal Pharyngeal swab for baby 2 .

◯ [baseline_arm_1][participant_pin]-NPB2

The mother tested positive for an STI at the Post Natal visit. You need to collect a Nasal Pharyngeal swab for baby 3. Did you manage to collect this specimen?

◯ Yes
◯ No

Please confirm the PIN for the Nasal Pharyngeal swab for baby 3.

◯ [baseline_arm_1][participant_pin]-NPB3

You have completed capturing the Post-Natal information. Please make sure to check that you have completed all the fields.

Please select "Unverified" then "Save and Exit".

Notes

Additional notes

For peer review only

[illegible]

Staff Name ____________________

Today's date ____________________

Start time ____________________

Was there an adverse birth outcome?

◯ Yes
◯ No

Was there a serious adverse event ◯ Yes ◯ No

Early loss of baby

What type of early loss?

◯ Miscarriage
◯ Ectopic
◯ Termination of pregnancy
◯ Still Born

Date

____

Ectopic pregnancy

Date of surgery

____

Termination pregnancy

Date

____

Reviewed by site PI ◯ Yes ◯ No

Date Reviewed ____________________

For peer review only

Review Notes

Name of Reviewer ◯ Remco Peters

You have completed capturing the adverse outcomes information. Please make sure to check that you have completed all the fields.

Please select "Complete" then "Save and Exit".

Notes

Additional notes

For peer review only

□□□□□□□□e□ □□□□□□a□e□ □□□□ □□□□□

S□a□□□ e□a□□□

Staff Name

____

Today's Date

____

Start time

____

Presentation Outcome

Presentation outcome.

Did the participant present at the study site for this visit?

◯ Yes
◯ No

Activities Associated with ToC for Arm 1

You are about to facilitate activities associated with the 4-week ToC. You will need to execute the following:

1. Collect Specimens
2. Run a STI test
3. Conduct clinical history and behavioral questionnaire
4. Symptom screening if negative test
5. Treatment and partner referral if positive test

◯ Proceed

Activities Associated with 32 Week Visit

You are about to facilitate activities associated with the 32 week visit. You will need to execute the following:

1. Collect Specimens
2. Run a STI test
3. Conduct clinical history and behavioral questionnaire
4. Symptom screening if negative test
5. Treatment and partner referral if positive test

◯ Proceed

You are about to facilitate activities associated with the 32 week visit. You will need to execute the following:

1. Collect Specimens
2. Conduct clinical history and behavioral questionnaire
3. Symptom screening
4. Treatment and partner referral if positive screening

◯ Proceed

For peer review only

[illegible]

You are about to facilitate activities associated with the the 1st post natal visit. You will need to execute the following:

1. Determine the presentation date (Only proceed when its 14 days after the delivery date)
2. Collect pregnancy and birth outcomes data (Discharge Summary and/or Road to Health Card)
3. Conduct mother and child clinical examination and history questionnaire
4. Specimen collection for mother and child

◯ Proceed

Pregnancy and Birth Outcome Data

You are about to start with the pregnancy and birth outcome data capturing.

You can use the discharge summary and road to health as your data sources.

Select "Proceed" below to display the pregnancy and birth outcome details

◯ Proceed

Delivery date ______________________________

Mother and Baby Clinical Examination and History

You are done capturing the pregnancy and birth outcome data.

The next step is to capture the mother and baby clinical examination and history details.

Select "Proceed" below to display the questionnaire.

◯ Proceed

Scheduling the 6 week Immunization Date

Schedule a 6 week immunization. ______________________________

Use the below date assist to schedule the 6 week immunization date.

The below field must be equal to 42.

Use the date field above to ensure that the current field is equal to 42.

____

For peer review only

[illegible]

You will need to collect a single vaginal loop that will be used to prepare two slides. Once collected you will need to prepare the slides for storage.

◯ Proceed

Date of collection of vaginal loops

____

....

Confirm the pin associated with the first vaginal loop that will be used for .....

◯ [baseline_arm_1][participant_pin]-FL1

Confirm the PIN associated with the second vaginal loop that will be used for .....

◯ [baseline_arm_1][participant_pin]-FL2

Storage of Loops

You have collected both slides. Before commencing with the rest of the specimens, please make sure to do the following:

1. Slides are individually packed in their own package
2. Record PIN on outside of package
3. Complete the lab CRF with matching PINs and test instructions

◯ Proceed

Vaginal Swab Collection

You will now collect 3 vaginal swabs. They will be used as follows:

1. STI testing (1st Specimen)
2. Profiling (2nd Specimen)
3. Microbiome (3rd Specimen)

◯ Proceed

....

Date of specimen collection for vaginal swabs

____

Confirm the PIN associated with the first vaginal swab

◯ [baseline_arm_1][participant_pin]-FV1

For peer review only

For peer review only - http://bmjopen.bmj.com/site/about/guidelines.xhtml

Confirm the PIN associated with the second vaginal swab

◯ [baseline_arm_1][participant_pin]-FV2

Confirm the PIN associated with the third vaginal swab

◯ [baseline_arm_1][participant_pin]-FV3

Nasopharyngeal Swab Collection

You are about to collect the Nasopharyngeal swab on the Baby.

Collect the specimen and confirm the PIN below.

◯ [baseline_arm_1][participant_pin]-NS1

GeneXpert Testing for the First Specimen

You will now start with the testing of the first vaginal swab specimen.

Follow the below steps:
1. Ensure that the GeneXpert Machine is switched-on. Perform a quikc qulaity check on the machine.
2. Load the specimen and run the machine.
3. Conduct Clinical History and Behavioural Questionnaire

Select "Start Test" when ready to run the test.

◯ Start Test

Clinical History and Behavioural Questionnaire

You have started running the STI test.

Conduct clinical history and behavioural questionnaire. Select "Proceed" to display the questionnaire

◯ Proceed

How often have you had sex since the last time we saw you?

◯ 0
◯ 1 to 5 times a week
◯ More than 5 times a week

STI Results

	Positive	Negative
NG	◯	◯
TV	◯	◯
CT	◯	◯

For peer review only

The participant tested positive for an STI.

The next step is to administer treatment with the participant.

Select "Proceed" to display treatment options.

◯ Proceed

.....

The next step is to screen the patient for STI symptoms

Is the participant symptomatic?

◯ Yes
◯ No

The participant screened positive for at least a single STI symptom

The next step is to administer treatment with the participant.

Select "Proceed" to display treatment options.

◯ Proceed

Treament and Partner Notification

Select the treatment regimen you administered to the participant

◯ Azithromycin
◯ Doxycyclin
◯ Ceftriaxone
◯ Metronidazole

Did you administer partner notification treatment?

◯ Yes
◯ No

Storage Processes

You have collected all required specimens.

You can now prepare the specimens for storage, follow the below steps:
1. Ensure that each specimen has a complete Lab CRF
2. Pack the Lab CRFs in the specimen container
3. Ensure that the Lab CRF is complete and specimens are stored according to the storage requirements.

Select "Confirm" after perform tha above specimen procedures.

◯ Confirm

For peer review only

[illegible]

Additional notes

For peer review only

□□□□e□□□

Staff member ______________________

Date ______________________

Participant ID: [baseline_arm_1][participant_pin_verify]

TERMINATION DETAILS

Date of termination ______________________

Study Time-Point
- ◯ BASELINE
- ◯ TOC
- ◯ 32 WEEKS
- ◯ POST-NATAL VISIT

Reason for termination
- ◯ End of study (study completed)
- ◯ death (participant)
- ◯ Participant refused further participation
- ◯ Participant unable to adhere to visit schedule
- ◯ Participant relocated, no follow-up planned
- ◯ Investigator decision
- ◯ unable to contact the participant
- ◯ Participant not eligible for enrollment
- ◯ Invalid ID due to duplicate screening/enrollment
- ◯ Other
- ◯ Early study closure
- ◯ End of study (adverse outcome)

Specify refusal reason/ Investigator reason ______________________

Other, Specify ______________________

....

General Comments ______________________

For peer review only

□□□□ □□

Staff name

____

Please capture date of visit

____

Please summarize the purpose of the visit

For peer review only

Sa[illegible]e[illegible] P[illegible]

Today's date	______________
Time	______________
Staff	______________
Safety Protocol Issue	◯ Social Harm<br>◯ Protocol Violation<br>◯ Unanticipated Problem
Date Reported	______________
Notes	______________

For peer review only

STI [illegible]a[illegible]a [illegible] [illegible] [illegible]g

S[illegible]TI[illegible] [illegible] ST[illegible] [illegible]T[illegible]I[illegible]S

Staff Member Name ________________________

Date ________________________

SECTION B: QUALITY ASSURANCE

Forms Received from the Field

☐ BQ Consent form
☐ Study Note
☐ Proof of Reimbursement
☐ Enrolment Log
☐ Expert Baseline: CT/NG
☐ Expert Baseline: TV
☐ Expert Postnatal: CT/NG
☐ Expert Postnatal: TV
☐ NHLS: CD4 Count
☐ NHLS: Syphilis Test
☐ NHLS: Viral Load
☐ NHLS: Baby HIV PCR
☐ OTHER
(Select all that you received)

Other - Specify the other form(s) received ________________________

QUALITY ASSURANCE: Phase 2A

Get all the participant's enrolment source documents and perform a comprehensive QC on all the source documents. After the QC is done, mark each document as "checked, properly completed" if you have no query opened on the source document.

IN CASES WHERE A QUERY IS OPENED. PLEASE CONTACT THE RESPONSIBLE DATA COLLECTOR IMMEDIATELY[illegible]

Please note that you also accept receipt of all source documents by checking them below.

	Checked, Properly Completed	Not completed, Returned to the RA
Consent Form	○	○
Study Note	○	○
Proof of Reimbursement	○	○
Enrolment Log	○	○
Expert Baseline: CT/NG	○	○

For peer review only - http://bmjopen.bmj.com/site/about/guidelines.xhtml

Expert Baseline: TV	◯	◯
Expert Postnatal: CT/NG	◯	◯
Expert Postnatal: TV	◯	◯
NHLS: CD4 Count	◯	◯
NHLS: Syphilis Test	◯	◯
NHLS: Viral Load	◯	◯
NHLS: Baby HIV PCR	◯	◯
[forms_received_oth]	◯	◯

Skip

Electronic Data QC

You are supposed to go through each electronic data tool and ensure the following:

1. Each Tracking Field has a data point.
2. The data is consistent
3. The data is verified with source documents

After doing the above inspection. You marked the forms as complete and locked the form.

Once all the forms are checked and properly completed.

	Checked and Completed Properly	Query Opened
1, Baseline: Screening and Enrolment	◯	◯
2, Baseline: Baseline data	◯	◯
3, STI: Physical Exam	◯	◯
4, STI: Specimen and Randomization	◯	◯
5, STI: Scheduling	◯	◯
6, STI: TOC Visit Activities	◯	◯
7, STI: Calling Reminder_2	◯	◯
8,STI: Scheduling post Delivery activities	◯	◯
9, Birth Register data	◯	◯
10, Post Natal Visit Activities	◯	◯
11, 6 week Immunization Visit Activities	◯	◯

review only

Date of QC

COMMENTS

Comments

SAVING INSTRUCTION

MARK THIS FORM AS COMPLET ONCE VERIFIED AND LOCK IT.

SELECT SAVE AND EXIT FORM.

Proceed to QC other source documents.

For peer review only

S[illegible]e[illegible]g 2

Scheduling of Dates Associated with [randomization].

NOTE
You are about to schedule dates associated with [randomization] participants.

Please select "Proceed".

◯ Proceed

Scheduling of Dates Associated

NOTE:
You are about to schedule dates associated with microbiome participants.

Please select "proceed"

◯ Proceed

For peer review only

Scheduling Dates for 3-Week ToC

NOTE: The participant tested positive and therefore we need to schedule a date, exactly 3-weeks from today to conduct a test-of-cure.

Scheduling the 3-week ToC

NOTE:
Please schedule a date, 3 weeks from today treatment given. Please use the calculator assistance to ensure that you schedule a date exactly 21 days from today.

____

Calculator Assist

The number here must be equal to 21

____

ERROR
The field does not equal to 21, please change it

Have you handed the TOC date to the participant?

◯ Yes
◯ No

Scheduling Dates Associated with ToC Reminder

Schedule date for REMINDER of 3-week ToC visit

____

Calculator Assist for scheduling ToC reminder date

The reminder phone call will be made 18 days following the treatment date. The number of days need to equal to 18.

____

ERROR
You did not enter the date correctly. The number should equal to 18. Please redo the date.

Scheduling Dates Associated with 3-Week ToC Missed Visit Date

NOTE:
You have successfully scheduled the reminder date.

Please select "proceed" to schedule the missed visit date for the 3 week ToC visit.

◯ Proceed

Schedule the date for the MISSED VISIT of the ToC visit.

This date should be 3 weeks after the date on which the participant received their test result.

____

Calculator Assist for scheduling 3-week ToC Missed Visit

The participant's time period allowed for attending a ToC will start 3 weeks after they received their result and will close 3 weeks after the date they received their result.

The number here must show 35

____

ERROR
You did not enter the date correctly. The number should equal to 35. Please redo the date.

NOTE:
You have successfully scheduled the 3-week ToC close date

Please select "proceed" to start scheduling the next visit dates

◯ Proceed

For peer review only

Dates Associated with reminder for the 28 Week call

NOTE:
You are about to schedule dates for the call reminder at 28 weeks.

Please select "Proceed".

◯ Proceed

Note:
Schedule the date for the 28 week call. We will contact each participant to ask the date for their 30 weeks clinic visit is.

____

Calculation assist for scheduling the 32-week reminder date.

This number must equal to 196

____

Days to call reminder

____

ERROR
The number you have entered does not match 196. Please select a different date so that the number equals to 196.

Scheduling the Dates Asscoiated with the 32 Week Gestational Visit

NOTE
You are about the start scheduling dates associated with the 32 week visit. You will need to schedule the following associated dates:

1. Week 32 date
2. Week 32 reminder date
3. Week 32 missed visit date

Select "Proceed" to start scheduling

◯ Proceed

Schedule the date for the 32 week gestational age, visit

Note to RA: please make sure that this date does not fall on Friday, weekend, and public holidays.

____

Days Difference (the difference between 32 weeks & Gestational age)

____

For peer review only

Calculate assist for 32 week visit

The number here must equal to [gest_week_calc]

____

Match

____

The date you have entered does not meet the 93 day criteria. Does the intended or original date fall on a Friday weekend or public holiday?

◯ Yes
◯ No

ERROR
The numbers you have entered does not match. Please select a different date so that the numbers match.

Dates Associated with reminder for the 32 Week Gestational Age Visit

NOTE:
You have successfully scheduled the 32 week date.

We will need to contact the participant at least 7 days before the scheduled visit to remind them.

Select "Proceed" to schedule the reminder date for the 32 week visit.

◯ Proceed

Note:
Schedule the date for the 32 week reminder. We will contact each participant starting 7 days prior to their 32-week gestation date. That means the date scheduled here should be 7 days earlier then the scheduled date for the 32-week visit.

____

Calculation assist for scheduling the 32-week reminder date.

This number must equal to 7

____

ERROR
The number you have entered does not match 7. Please select a different date so that the number equals to 7

For peer review only - http://bmjopen.bmj.com/site/about/guidelines.xhtml

[illegible]

[illegible]

NOTE:
You have successfully scheduled the 32 week reminder date.

Select "Proceed" to schedule the 32 week missed visit date.

◯ Proceed

Schedule the date for the 32 week missed visit date.

Note: Participants will have 3 weeks (21 days) to present for their 32 week visit date after which the visit will be closed out.

____

Calculation Assist for scheduling the 32-Week missed visit date.

This number must equal to 21

____

ERROR
The number you have entered does not match 21. Please select a different date so that the number equals to 21

Estimated Delivery Date

You are about the schedule the Estimated Delivery Date.

Please select "proceed"

◯ Proceed

Estimated Delivery Date

____

Days difference between estimated date of delivery and gestational age

____

For peer review only

Calculation Assist for scheduling the Estimated Date for Delivery date.

This number must equal to [edod_calc]

____

Match ______________________________

ERROR
The number you have entered does not match. Please select a different date so that the numbers match

You have completed all the scheduling dates.

Please check that all dates entered comply with the "calculation assistance". Once this has been done you can select "Complete" and "Save & Exit"

NOTES

Notes box

For peer review only

[illegible]

Name ______________________

Time ______________________

For peer review only

[illegible]

Please specify the updated delivery date

For peer review only

Genital tract infections, the vaginal microbiome and gestational age at birth among pregnant women in South Africa: a cohort study protocol

Journal:	*BMJ Open*
Manuscript ID	bmjopen-2023-081562.R1
Article Type:	Protocol
Date Submitted by the Author:	28-Nov-2023
Complete List of Authors:	Gigi, Ranjana ; University of Bern, Institute of Social and Preventive Medicine; Foundation for Professional Development, Research Unit<br>Mdingi, Mandisa ; Foundation for Professional Development, Research Unit<br>Jung, Hyunsul; University of Pretoria, Medical Microbiology<br>Claassen-Weitz, Shantelle; University of Cape Town, Department of Pathology<br>Bütikofer, Lukas; University of Bern, CTU Bern, Department of Clinical Research<br>Klausner, Jeffrey D.; University of Southern California, Department of Population and Public Health Sciences, Keck School of Medicine<br>Muzny, Christina; University of Alabama at Birmingham, Division of Infectious Diseases<br>Taylor, Christopher; Louisiana State University Health Sciences Center, Department of Microbiology, Immunology, and Parasitology<br>van de Wijgert, Janneke H.H.M.; Utrecht University, Julius Center for Health Sciences and Primary Care, University Medical Center Utrecht<br>Peters, Remco; Foundation for Professional Development, Research Unit; University of Pretoria, Department of Medical Microbiology<br>Low, Nicola; University of Bern, Institute of Social and Preventive Medicine
**Primary Subject Heading**:	Epidemiology
Secondary Subject Heading:	Obstetrics and gynaecology, Infectious diseases, HIV/AIDS
Keywords:	EPIDEMIOLOGIC STUDIES, Sexually Transmitted Disease, Diagnostic microbiology < INFECTIOUS DISEASES, Maternal medicine < OBSTETRICS, Follow-Up Studies

SCHOLARONE™
Manuscripts

Genital tract infections, the vaginal microbiome and gestational age at birth among pregnant women in South Africa: a cohort study protocol

Ranjana M S Gigi,[1 2] Mandisa M Mdingi,[2] Hyunsul Jung,[3] Shantelle Claassen-Weitz,[4] Lukas Bütikofer,[5] Jeffrey D Klausner,[6] Christina A Muzny,[7] Christopher M Taylor,[8] Janneke H H M van de Wijgert*,[9] Remco P H Peters*,[2 3 4] Nicola Low*[1]

1) Institute of Social and Preventive Medicine, University of Bern, Bern, Switzerland

2) Research Unit, Foundation for Professional Development, East London, South Africa

3) Department of Medical Microbiology, University of Pretoria, Pretoria, South Africa

4) Department of Pathology, University of Cape Town, Cape Town, South Africa

5) CTU Bern, Department of Clinical Research, University of Bern, Bern, Switzerland

6) Keck School of Medicine, University of Southern California, Los Angeles, USA

7) Division of Infectious Diseases, University of Alabama at Birmingham, Birmingham, USA

8) School of Medicine, Louisiana State University, USA

9) Julius Center for Health Sciences and Primary Care, University Medical Center Utrecht, Utrecht University, Utrecht, Netherlands

Correspondence: Prof. Nicola Low (nicola.low@unibe.ch)

*Contributed equally

Word counts: Abstract, 294; main text, 3736; 2 figures, 1 supplemental online file

Keywords

Pregnancy complications, premature birth, reproductive tract infections, sexually transmitted infections, microbiota, South Africa

For peer review only

Abstract

Introduction Preterm birth complications are the most common cause of death in children under 5 years. The presence of multiple microorganisms and genital tract inflammation could be the common mechanism driving early onset of labour. South Africa has high levels of preterm birth, genital tract infections and HIV infection among pregnant women. We plan to investigate associations between the presence of multiple lower genital tract microorganisms in pregnancy and gestational age at birth.

Methods and analysis This cohort study enrols around 600 pregnant women at one public health care facility in East London, South Africa. Eligible women are ≥18 years and at <27 weeks of gestation, confirmed by ultrasound. At enrolment and 30-34 weeks of pregnancy, participants receive on-site tests for *Chlamydia trachomatis* and *Neisseria gonorrhoeae*, with treatment if test results are positive. At these visits, additional vaginal specimens are taken for: PCR detection and quantification of *Trichomonas vaginalis*, *Candida* species, *Mycoplasma genitalium, M. hominis*, *Ureaplasma urealyticum* and *U. parvum*; microscopy and Nugent scoring; and for 16S ribosomal ribonucleic acid gene sequencing and quantification. Pregnancy outcomes are collected from a post-natal visit and birth registers. The primary outcome is gestational age at birth. Statistical analyses will explore associations between specific microorganisms and gestational age at birth. To explore the association with the quantity of microorganisms, we will construct an index of microorganism load and use mixed effects regression models and classification and regression tree analysis to examine which combinations of microorganisms contribute to earlier gestational age at birth.

Ethics and dissemination This protocol has approvals from the University of Cape Town Research Ethics Committee and the Canton of Bern Ethics Committee. Results from this study will be uploaded to preprint servers, submitted to open access peer-reviewed journals and presented at regional and international conferences.

Registration ClinicalTrials.gov Identifier: NCT06131749

For peer review only

For peer review only - http://bmjopen.bmj.com/site/about/guidelines.xhtml

Article summary, strengths and limitations of the study

- This cohort study takes a holistic approach, investigating both the presence and quantity of multiple lower genital tract microorganisms, including vaginal microbiota, in pregnancy and their associations with gestational age at birth.
- The study is set in a location where the prevalence of genital tract infections and adverse pregnancy outcomes are high, uses ultrasound scans to assess gestational age at enrolment accurately, and state-of-the-art molecular diagnostic methods.
- The study setting is limited to one research site, which may affect the generalisability of the findings.
- The use of gestational age at birth as a continuous outcome, instead of preterm birth as a dichotomous outcome, might limit comparability with other studies, but we will also examine the binary outcome preterm birth in secondary analyses.

For peer review only

For peer review only - http://bmjopen.bmj.com/site/about/guidelines.xhtml

Introduction

Preterm birth complications are the most common cause of death in children under 5 years.[1] Close to one million infants die every year because they are born preterm (before 37 completed weeks of gestation), mainly from infectious, respiratory and neurological complications, and those that survive can experience long-term morbidity.[1], [2] South Africa has a high incidence of preterm birth at around 10%,[3] around 30% of women have one or more curable sexually transmitted infections during pregnancy [4], [5] and about 30% of pregnant women are living with HIV. [6]

Microbial colonisation or infection during pregnancy, in the lower or upper genital tract, have been reported to predispose to preterm birth, as do anatomical, biochemical, endocrinological, immunological, nutritional, environmental and psychosocial factors.[7], [8] The presence of microorganisms may contribute to early onset of labour directly, through presumed ascension from the lower to the upper genital tract, or indirectly, through a pathway of inflammatory response, or a combination of both.[7], [9] Inflammation may be the common pathway, even if infection has not reached the amniotic cavity. [10]

Much of the research reporting on the role of sexually transmitted infections in pregnancy and preterm birth has focused on single infections, such as *Chlamydia trachomatis,* [11] *Neisseria gonorrhoeae,* [12] and *Trichomonas vaginalis.* [13] *Mycoplasma genitalium* is the most recently recognised sexually transmitted infection and, whilst an association with preterm birth has been reported, there are few studies with prospective data collection. [14] Bacterial vaginosis is the most common vaginal microbiota dysbiosis and is associated with adverse pregnancy outcomes, either alone, or in combination with other sexually transmitted infections. [15-17] Associations with adverse birth outcomes have also been observed for other genital mycoplasmas, *M. hominis, Ureaplasma urealyticum* and *U. parvum*. [18] For individual sexually transmitted infections, bacterial vaginosis and colonisation by other genital mycoplasmas, summary odds ratios for the association with adverse birth outcomes in meta-analyses of univariable data are generally around 1.3 to 2.0. [11-14, 16, 18] *Candida* spp. have not been found to be associated with preterm birth, but an association with more inflammatory, symptomatic yeast infection cannot be ruled out. [19] Most studies about these microorganisms do not present analyses that examine the role of co-occurrence or control for confounding factors, so the presence or strength of the causal association cannot be assessed. [20] It is also important to include women living with HIV, amongst whom there are fewer studies about associations between genital tract infections and adverse birth outcomes than amongst women without HIV infection. [21-22]

The importance of the quantity of different microorganisms as a driver of preterm birth has not been extensively studied, [23-25] but might be as, or more, relevant than their presence. [23] Together with inflammation or immune activation in the genital tract during pregnancy, organism load could be an important driver of the early onset of labour and preterm birth.[8,23,24,26,27] This calls for a holistic approach to research studies, which combines information about the presence of different microorganisms, the quantified load and the microbiome, sociodemographic factors and HIV amongst women living with infection, most of whom are receiving antiretroviral therapy. The overall aim of this study is to investigate associations between the presence of lower genital tract microorganisms in pregnancy and preterm birth and other adverse pregnancy outcomes. This will be achieved through three objectives to explore: (1) the association between the presence of specific lower genital tract microorganisms and gestational age at birth (primary outcome), as well as

For peer review only - http://bmjopen.bmj.com/site/about/guidelines.xhtml

secondary adverse pregnancy outcomes; (2) the association between quantified load of vaginal and sexually transmitted microorganisms and gestational age at birth (primary outcome) as well as secondary adverse pregnancy outcomes; and (3) the combinations of microorganisms that are most strongly associated with earlier gestational age at birth.

Methods and analysis

STUDY DESIGN AND SETTING

This prospective closed cohort study follows women enrolled during pregnancy until after they give birth (Figure 1). The study is conducted at the antenatal clinic of one primary health care facility in Buffalo City Metropolitan Municipality, Eastern Cape Province, South Africa. This cohort study is part of a larger project, called Philani Ndiphile (meaning 'be healthy and I will be healthy' in isiXhosa), which includes a randomised implementation-effectiveness trial of screening strategies for sexually transmitted infections in pregnancy [28]and a case-control study about persistent *C. trachomatis* infection.

PARTICIPANTS

Inclusion criteria: Pregnant women aged 18 years or older, who live in Buffalo City Metropolitan Municipality, intend to deliver in the same municipality and provide written informed consent to take part in the study. The eligible gestational age at enrolment, confirmed by ultrasound, was below 20 weeks at the start of the study in March 2021 and was increased to 27 weeks in September 2021 to increase enrolment and to align with another trial. [29]

Exclusion criteria: Participation in any other research study or inability to understand and speak a local language (English, Afrikaans, or isiXhosa).

Figure 1 Study visits and specimen collection
Abbreviations: CT, *Chlamydia trachomatis*; NG, *Neisseria gonorrhoeae*

ENROLMENT

A trained study field worker approaches all pregnant women attending an antenatal care visit at the clinic and individually informs them about the study. If a potential participant shows interest in the study, the study field worker checks for eligibility. The date of the last menstrual period is used initially to estimate gestational age. If all eligibility criteria are met, a study field worker obtains written informed consent from the participant.

STUDY PROCEDURES AND VISITS

At the enrolment visit, study field workers administer a questionnaire to record socio-demographic, behavioural and clinical information in an online Research Electronic Data Capture software (REDCap) [30] database. The study nurse examines the woman, according to the South African government standard of care. [31] As an additional procedure, a study nurse with training in obstetric ultrasound performs an abdominal ultrasound to estimate the gestational age. If this is later than the eligibility criterion, the participant is excluded from any further study activity. A study nurse collects vaginal samples (Figure 1) for on-site testing for *C. trachomatis* and *N. gonorrhoeae*

For peer review only - http://bmjopen.bmj.com/site/about/guidelines.xhtml

using the Xpert CT/NG assay on the Gene Xpert platform (Cepheid, Sunnyvale, CA, USA) and for further off-site laboratory testing (see 'Specimen collection and analysis').

If the test result for *C. trachomatis* or *N. gonorrhoeae* is positive, the woman receives immediate antibiotic treatment if still on site or is contacted by telephone and asked to return to the clinic for treatment. Antibiotic treatments are first-line regimens according to South African guidelines: for *C. trachomatis*, 1g oral azithromycin and for *N. gonorrhoeae*, 500mg intramuscular ceftriaxone (250mg until South African treatment guidelines for sexually transmitted infections changed in December 2022). [32] Women with vaginal discharge syndrome but with negative Xpert test results for *C. trachomatis* and *N. gonorrhoeae* receive empirical treatment for trichomoniasis with metronidazole 400 mg twice a day for 7 days. The study nurse gives advice to women with *C. trachomatis* or *N. gonorrhoeae* on safe disclosure of her diagnosis to her partner(s) and gives her a notification slip(s) to request her partner(s) to attend a clinic for treatment.

A follow-up visit at 30-34 weeks (third trimester visit) is scheduled at which clinical and obstetric information, as well as the same vaginal specimens, are collected and treatment given, if indicated.

A post-natal visit is scheduled for 3-6 days after giving birth, according to the South African government standard. [31] A study nurse collects information about the birth outcome and perinatal period through a questionnaire with the mother, a patient-held medical record of the baby (the Road to Health card) and/or the birth register from the public birth clinics within the study area. If the participant does not attend the post-natal visit, study staff telephone her to ask her to return to the clinic. If the participant is not able to return to the clinic, the study physician collects the information by telephone or from the birth register.

OUTCOMES

The primary outcome is gestational age at birth, measured in days, based on the ultrasound assessment at the enrolment visit. Secondary outcomes are preterm birth (<37 completed weeks of gestation), low birth weight (birth weight <2500g), miscarriage (dead foetus delivered before 28 completed weeks of pregnancy or with birth weight below 1000g) and stillbirth (dead foetus delivered at or after 28 completed weeks of pregnancy or with birth weight above 1000g). [33,34] We chose gestational age at birth as the primary outcome because, whilst the cut-off of 37 weeks is the standard definition of preterm birth, dichotomisation of a continuous variable results in a loss of statistical power. [35]

For peer review only

SPECIMEN COLLECTION AND ANALYSIS

DATA SOURCES AND VARIABLES

The source data are case report forms recording questionnaire data for the enrolment, third trimester and post-natal visits and forms for laboratory and specimen results, which are stored in REDCap, a secure web-based database [30] (online supplemental file), hosted by the Foundation for Professional Development, Pretoria, South Africa.

SPECIMEN COLLECTION

At the enrolment and the third trimester visits, a study nurse collects two vaginal smears using inoculation loops and air-dries them on glass slides. She then collects vaginal specimens by inserting swabs into the vagina up to a mark at 4 cm and rotating around the vaginal wall. Five swabs are collected in the following order: one Cepheid GeneXpert Xpert Vaginal/Endocervical Swab in a tube

For peer review only - http://bmjopen.bmj.com/site/about/guidelines.xhtml

with Xpert Swab Transport Reagent (Cepheid, Sunnyvale, CA, USA); two Qiagen digene Female Swabs in a single tube with digene Specimen Transport Medium (Qiagen, Hilden, Germany); and two dry FLOQswabs (COPAN, Brescia, Italy) each in a separate sterile tube (Figure 1).

TRANSPORT AND STORAGE OF SPECIMENS

Vaginal smear glass slides are stored and transported in plastic slide carriers at room temperature. All vaginal swabs are initially stored at the clinic in a refrigerator (2-8°C with daily temperature checks). All vaginal swabs, except the Xpert swab, which is tested on-site, are transported on ice packs once a week by overnight road courier, to the laboratory at the Department of Medical Microbiology, University of Pretoria, where they are also stored in a refrigerator until DNA extraction.

MICROBIOLOGICAL ANALYSES

The Xpert vaginal swabs are tested on-site using the Xpert CT/NG assay (Cepheid, Sunnyvale, CA, USA) to detect *C. trachomatis* and *N. gonorrhoeae*, as per manufacturer's instructions. At the University of Pretoria, air-dried vaginal smears are heat-fixed and Gram-stained. [36] Two qualified people record the Nugent scores (0-3: normal; 4-6: intermediate; 7-10: bacterial vaginosis) and the presence of yeasts. [37] In case of discrepancies a third person assesses the slide and consensus is reached by discussion. At the University of Pretoria, one vaginal FLOQswab is used for PCRs. The genomic DNA is extracted using the High Pure PCR Template Preparation Kit (Roche Diagnostics GmbH, Mannheim, Germany) as per manufacturer's instructions. Real-time PCR assays are then performed using the LightCycler 480 Probes Master Kit (Roche Diagnostics GmbH, Mannheim, Germany) on the LightCycler 480 II instrument (Roche Diagnostics GmbH, Mannheim, Germany). Previously published primer and hydrolysis probe sequences and cycling conditions are used for detection and quantification of *M. genitalium*, [38] *M. hominis*, [39] *U. parvum*, [40] *U. Urealyticum,* [40] *T. vaginalis* [41] and *Candida* spp. [42,43] The load for each assayed microorganism detected in vaginal swab specimens by real-time PCR or GeneXpert is obtained from the cycle threshold value.

VAGINAL MICROBIOME LABORATORY ANALYSES

The vaginal swabs stored in Qiagen digene Specimen Transport Medium will be used for DNA extraction and subsequent 16S ribosomal ribonucleic acid (rRNA) amplicon sequencing targeting the V3-V4 hypervariable regions for vaginal microbiota analyses at the Division of Medical Microbiology, University of Cape Town.

A commercial DNA extraction kit will be used and a bead-beating step included. [44] A DNA isolation control will be prepared from an unused vaginal swab specimen during this process. Two PCR rounds will be conducted to prepare amplicon libraries.[45] The aim of the first PCR round is to amplify 16S rRNA gene V3-V4 regions, using the 319F 5'-ACTCCTACGGGAGGCAGCAG-3' forward primer and 806R 5'-GGACTACHVGGGTWTCTAAT-3' reverse primer. The aim of the second PCR round is to barcode the V3-V4 amplicons by a dual-index approach, permitting multiplexing of up to 384 samples (including controls). Amplicon concentrations for all sample libraries are measured and normalised to form a mixed loading library. The libraries will be sequenced on an Illumina MiSeq instrument (Illumina, San Diego, CA, USA), 2x300bp. To quantify the number of 16S rDNA copies per swab, a quantitative PCR using the same forward and reverse primers as described above will be used. Samples from enrolment and third trimester visits from the same woman will be processed in the same run.

For peer review only - http://bmjopen.bmj.com/site/about/guidelines.xhtml

VAGINAL MICROBIOTA BIOINFORMATICS

Raw sequencing reads will be processed using an established bioinformatics pipeline.[46] Taxonomic assignment of amplicon sequence variants (ASVs) will be done in DADA2[47] with SILVA[48] as the reference database. Vaginal microbiome composition data will be visualised in heatmaps and diagrams. For each vaginal sample, we will calculate diversity measures (alpha diversity), relative abundances and estimated concentrations of key vaginal bacteria and bacterial groups, as described.[46] We will use the entire sequencing dataset to design vaginal microbiota types by hierarchical clustering, and each sample will be assigned to one vaginal microbiota type.

SAMPLE SIZE CALCULATION

The sample size has been calculated for objective 1, with a univariable comparison between the presence of a genital tract microorganism in the mother and gestational age at birth. Figure 2 shows that, for any vaginal or sexually transmitted microorganism, or vaginal microbiota type that has a prevalence of 10% or more among all enrolled women, about 500-600 patients provides adequate power (80%) to detect a one-week difference (with standard deviation 2) in mean gestational age between the two groups using Student's t-test. Specifying an alpha of 0.83% allows for multiple hypothesis testing (6 hypotheses, using a Bonferroni correction). We enrol around 600 women and aim to have complete follow-up and outcome data on at least 550 women.

Figure 2 Sample size requirements at different levels of exposure prevalence with power of 80% and alpha 0.83% based on Student's t-test.

Legend: panel A, standard deviation 1.5; panel B, standard deviation 2.0. The curves for % exposed are symmetrical around a prevalence of 50%, i.e., curve for 10% exposed is same as that for 90% exposed.

STUDY TIMELINE

Enrolment began on 28 March 2021, with an estimated date for reaching the target sample size in August 2023. Follow-up of all participants until the post-natal visit is expected to be completed by March 2024.

STATISTICAL ANALYSIS

This description gives an overview of the statistical methods for each objective. A detailed statistical analysis plan will be published separately and made publicly available.

We will describe the numbers of women enrolled and available at each follow-up visit in a flow chart. We will present descriptive tables of socio-demographic, behavioural and clinical characteristics and compare women with complete follow-up with those lost to follow-up.

OBJECTIVE 1) ASSOCIATION BETWEEN SPECIFIED EXPOSURES AND PREGNANCY OUTCOMES

1a. We will examine a primary set of microorganisms as exposures, detected at either enrolment or at the third trimester visit: *M. genitalium, M. hominis*, *U. urealyticum, U. parvum, T. vaginalis* and *Candida* spp. are the microorganisms for which women did not receive diagnostic tests and treatment during study visits. We will use the mean and standard deviation for the continuous outcome (gestational age) and absolute and relative frequencies for the binary outcomes (all secondary outcomes). Gestational age at birth for each exposure will be compared using Student's t-test and a mean difference with 95% confidence intervals (continuous outcome) and Fisher's exact test and a risk difference with 95% confidence intervals (binary outcomes).

For peer review only - http://bmjopen.bmj.com/site/about/guidelines.xhtml

To control for confounding, multivariable regression models will be fitted to all outcomes with a set of pre-specified potential confounders (age, educational level, alcohol consumption, HIV infection, prior preterm birth) for all organisms. For analyses of *M. genitalium*, *T. vaginalis*, and *Candida* spp., we will also control for bacterial vaginosis (Nugent score 7-10). The other genital mycoplasmas can be identified from 16S rRNA amplicon sequencing in women with vaginal dysbiosis, so are sometimes considered part of bacterial vaginosis. For these organisms, we will conduct descriptive analyses, stratifying by the presence of bacterial vaginosis. For continuous confounders a linear relationship will be assumed by default but transformations (e.g., log) or more flexible approaches (e.g., splines or fractional polynomials) will be considered if there is evidence for non-linearity. For the continuous outcome we will use linear mixed effects regression models (including data from either visit and the participant as random effect) and report the result as mean difference with 95% confidence intervals. For the binary outcome we will use logistic mixed-effects regression and report the result as odds ratios with 95% confidence intervals.

1b. Comparisons for associations with timing of detection, other microorganism exposures and birth outcomes will be considered secondary analyses. Associations between vaginal microbiota composition and pregnancy outcomes will be assessed. We will use compositional multivariable analysis methods to identify bacterial taxa that are differentially abundant between binary pregnancy outcome groups at the level of individual taxon relative abundances. We will use mixed effects models (with the individual participant as the random effect and including data from both visits) to assess associations between continuous and binary pregnancy outcome and the following fixed effects derived from the vaginal microbiota data: alpha diversity, vaginal microbiota types and absolute abundances of predefined bacterial groups. [46] These models will be adjusted for confounding as described in the previous paragraph.

OBJECTIVE 2) ASSOCIATION BETWEEN QUANTIFIED MICROORGANISM LOAD AND PREGNANCY OUTCOMES

We will investigate the hypothesis that the quantity of microorganisms with inflammatory potential is associated with gestational age at birth. For this, we will analyse the vaginal microbiota data jointly with sexually transmitted infections and *Candida* spp. diagnostic test results during pregnancy (these will be considered as additional covariates in the above-mentioned regression models). We will develop a ‘vaginal inflammation index’, based on quantification of the vaginal microbiota and their inflammatory potential [49] and of yeasts. This vaginal inflammation index will also be analysed as a fixed effect in mixed effects models with pregnancy outcomes as the outcomes; these models will not include any of the infection parameters that were used to design the index.

OBJECTIVE 3) CLASSIFICATION AND REGRESSION TREE ANALYSIS FOR THE PRIMARY OUTCOME

We will conduct exploratory analyses to examine the combination of microorganisms that best predicts earlier gestational age at birth using classification and regression tree analysis. [50] This method belongs to the family of decision tree machine learning algorithms and allow for nonparametric analyses of a large number of binary, categorical or continuous predictors. They are typically easy to interpret and can detect predictors with small marginal effects when there are strong interaction effects. We will make use of the predictive potential for gestational age at birth of all sexually transmitted and genital tract microorganisms, including individual bacterial taxa or bacterial groups identified by 16S rRNA gene amplicon sequencing (as binary or continuous variables) and confounding variables identified in objective 1. We will present variable importance scores and curves of marginal effects to show how prediction of the outcome changes at different

For peer review only - http://bmjopen.bmj.com/site/about/guidelines.xhtml

levels of the exposure of each variable in the model. To avoid overfitting, we will consider bootstrap aggregating via random forests. [51]

DATA MANAGEMENT AND CONFIDENTIALITY

DATA MANAGEMENT

Each potential participant screened for eligibility is assigned a unique participant identification number, which does not include any personal identifying information. Personal identification numbers are used to link records, specimens and laboratory test results of the participants. Data are stored in a REDCap database, [30] which is only accessible to authorised project staff. Paper records are kept in lockable fire-resistant filing cabinets. Laboratory records and journals are kept at the University of Pretoria and University of Cape Town. Forms with personal identifying information are kept separately from demographic, clinical and other data. The data manager maintains a separate, access-controlled, database that links the personal identification number with identifying information. Data quality checks are conducted by study staff onsite and data administrators at the office of the Foundation for Professional Development. All study data are stored securely at the offices of the Foundation for Professional Development in East London for up to five years after the completion of the study or as required by the institutional review board.

CONFIDENTIALITY

The research team is trained to adhere to guidelines on the Protection of Human Research Participants and Good Clinical Practice and fully protects the confidentiality of participants. Besides the measures described under data management, interviews are conducted in a private setting. In reports and publications, data will not be presented in a way where it could be linked to individual participants.

PATIENT AND PUBLIC INVOLVEMENT

There was no involvement of patients or the public in the development of the research questions or the study methods. The research findings will be shared through open access publications and in dissemination meetings with local stakeholders, healthcare providers and communities.

Discussion

This project is important because of its holistic approach, which considers associations between different genital tract infections, their quantity and the vaginal microbiota on earlier gestational age at birth. Many studies in this field have focused on only one or two microorganisms and few studies involve women in sub-Saharan Africa. Strengths of this study include the study setting, where the prevalence of both genital tract infections and adverse pregnancy outcomes is high, the use of ultrasound scans at enrolment for accurate assessment of gestational age and the use of state-of-the-art molecular diagnostic tests and 16S rRNA sequencing. The residual DNA from samples collected in this study will be available for future studies, including joint analyses with other studies of the influence of vaginal microbiota on adverse pregnancy outcomes.

There are limitations to the study design. First, this study involves participants from one clinic, which might limit the generalisability of the findings. Second, using gestational age at delivery as a

For peer review only - http://bmjopen.bmj.com/site/about/guidelines.xhtml

For peer review only

continuous outcome instead of preterm birth as a dichotomous outcome, might limit comparability with other studies. We will, however, examine the binary outcome preterm birth in secondary analyses. Third, the vaginal samples are taken in a fixed sequence at each visit, which might reduce the microorganism load of later samples. Fourth, the development of the vaginal inflammation index will use information about the inflammatory potential of microorganisms, [49] rather than direct concentrations of inflammatory markers.

This study has the potential to generate new evidence about the role of different microorganisms in earlier gestational age at birth through analyses of the presence and quantity of individual and combinations of microorganisms, relative abundance of bacterial genera and microbiota on gestational age at birth. This study will generate new hypotheses, which can be investigated in future studies.

Ethics and dissemination

This protocol and the informed consent forms are approved by the University of Cape Town, Research Ethics Committee (Reference: 676/2019), which includes activities at the University of Southern California, University of Alabama at Birmingham and Louisiana State University. Authorisation to analyse de-identified data at the University of Bern has been granted by the Canton of Bern Ethics Committee (Reference: 2021-01209). Results from this study will be submitted to regional and international conferences and to open access peer-reviewed journals and preprint servers.

Data statement

The research team will prepare datasets used in analyses, in accordance with data sharing requirements of open access journals in which manuscripts are published and in compliance with local Protection of Personal Information Act requirements. These data files will be archived with codebooks as .csv documents or R data sets and stored in REDCap. The final data files will not contain any personal identifying information of participants.

Author contributions

RG, NL, JW, and RP conceived and designed the study. JK supported the study through design of the parent study of the Philani Ndiphile project. RG, NL, JW, RP, HJ, CT, CM, SC and LB contributed to the data analysis plan. RG, NL, RP, MM and HJ were involved with the implementation and management of the study. RG and MM managed the data acquisition. RG, NL, JW, RP drafted the manuscript and all authors revised it. NL, JW, RP and JK supervised the study. All authors read and approved the final manuscript.

Funding statement

For peer review only

This work is supported by an MD-PhD scholarship from the Swiss National Science Foundation (grant number 191225), the Swiss National Science Foundation (grant number 197831) and the US National Institutes of Health (grant number R01AI149339).

Competing interests statement

LB, SC, RG, HJ, JK, NL, MM, RP, CT, JW: no competing interests to declare.

CM has received research grant funding to her institution by Gilead Inc., Abbott Molecular, Visby, and Lupin Pharmaceuticals. She is a consultant to BioNTech, Cepheid, and BioFire Diagnostics. She has received honoraria for educational presentations and review activities from Scynexis, Visby, Abbott, Elsevier, UpToDate, and DynaMed Plus.

For peer review only

References

1. UN Inter-agency Group for Child Mortality Estimation. Levels & Trends in Child Mortality: Report 2019. New York: United Nations Children's Fund, 2019. https://www.unicef.org/reports/levels-and-trends-child-mortality-report-2019 (accessed 24 October 2023).

2. Saigal S, Doyle LW. An overview of mortality and sequelae of preterm birth from infancy to adulthood. *Lancet* 2008;371:261-9. doi: 10.1016/S0140-6736(08)60136-1

3. World Health Organization. Born too soon: decade of action on preterm birth. Geneva: World Health Organization, 2023. https://www.who.int/publications/i/item/9789240073890 (accessed 24 October 2023).

4. Joseph Davey DL, Nyemba DC, Gomba Y, et al. Prevalence and correlates of sexually transmitted infections in pregnancy in HIV-infected and- uninfected women in Cape Town, South Africa. *PLoS One* 2019;14(7):e0218349. doi: 10.1371/journal.pone.0218349

5. Moodley D, Moodley P, Sebitloane M, et al. High prevalence and incidence of asymptomatic sexually transmitted infections during pregnancy and postdelivery in KwaZulu Natal, South Africa. *Sex Transm Dis* 2015;42(1):43-7. doi: 10.1097/OLQ.0000000000000219

6. Woldesenbet SA, Lombard C, Manda S, et al. The 2019 National Antenatal Sentinel HIV Survey (ANCHSS) Key Findings. South Africa: National Department of Health, 2021. https://www.nicd.ac.za/wp-content/uploads/2019/07/Antenatal_survey-report_24July19.pdf (accessed 24 October 2023).

7. Romero R, Espinoza J, Kusanovic JP, et al. The preterm parturition syndrome. *BJOG* 2006;113(Suppl 3):17-42. doi: 10.1111/j.1471-0528.2006.01120.x

8. Hunter PJ, Awoyemi T, Ayede AI, et al. Biological and pathological mechanisms leading to the birth of a small vulnerable newborn. *Lancet* 2023;401(10389):1720-32. doi: 10.1016/S0140-6736(23)00573-1

9. Goldenberg RL, Culhane JF, Iams JD, et al. Epidemiology and causes of preterm birth. *Lancet* 2008;371:75-84. doi: 10.1016/S0140-6736(08)60074-4

10. MacIntyre DA, Sykes L, Teoh TG, et al. Prevention of preterm labour via the modulation of inflammatory pathways. *J Matern Fetal Neonatal Med* 2012;25 Suppl 1:17-20. doi: 10.3109/14767058.2012.666114

11. Tang W, Mao J, Li KT, et al. Pregnancy and fertility-related adverse outcomes associated with *Chlamydia trachomatis* infection: a global systematic review and meta-analysis. *Sex Transm Infect* 2019 doi: 10.1136/sextrans-2019-053999

12. Vallely LM, Egli-Gany D, Wand H, et al. Adverse pregnancy and neonatal outcomes associated with Neisseria gonorrhoeae: systematic review and meta-analysis. *Sex Transm Infect* 2021;97(2):104-11. doi: 10.1136/sextrans-2020-054653

13. Van Gerwen OT, Craig-Kuhn MC, Jones AT, et al. Trichomoniasis and adverse birth outcomes: a systematic review and meta-analysis. *BJOG* 2021;128(12):1907-15. doi: 10.1111/1471-0528.16774

For peer review only - http://bmjopen.bmj.com/site/about/guidelines.xhtml

14. Frenzer C, Egli-Gany D, Vallely LM, et al. Adverse pregnancy and perinatal outcomes associated with Mycoplasma genitalium: systematic review and meta-analysis. *Sex Transm Infect* 2022;98(3):222-27. doi: 10.1136/sextrans-2021-055352

15. Hillier SL, Nugent RP, Eschenbach DA, et al. Association between bacterial vaginosis and preterm delivery of a low-birth-weight infant. The Vaginal Infections and Prematurity Study Group. *N Engl J Med* 1995;333(26):1737-42. doi: 10.1056/NEJM199512283332604

16. Leitich H, Bodner-Adler B, Brunbauer M, et al. Bacterial vaginosis as a risk factor for preterm delivery: a meta-analysis. *Am J Obstet Gynecol* 2003;189(1):139-47. doi: 10.1067/mob.2003.339

17. van de Wijgert JHHM. The vaginal microbiome and sexually transmitted infections are interlinked: consequences for treatment and prevention. *PLoS Med* 2017;14(12):e1002478. doi: 10.1371/journal.pmed.1002478

18. Jonduo ME, Vallely LM, Wand H, et al. Adverse pregnancy and birth outcomes associated with Mycoplasma hominis, Ureaplasma urealyticum and Ureaplasma parvum: a systematic review and meta-analysis. *BMJ Open* 2022;12(8):e062990. doi: 10.1136/bmjopen-2022-062990

19. Gigi RMS, Buitrago-Garcia D, Taghavi K, et al. Vulvovaginal yeast infections during pregnancy and perinatal outcomes: systematic review and meta-analysis. *BMC Womens Health* 2023;23(1):116. doi: 10.1186/s12905-023-02258-7

20. Low N. *Chlamydia trachomatis* and reproductive health: what can we learn from systematic reviews of observational studies? *Sex Transm Infect* 2020;96(5):315-17. doi: 10.1136/sextrans-2019-054279

21. Moodley D, Sartorius B, Madurai S, et al. Pregnancy outcomes in association with STDs including genital HSV-2 shedding in a South African cohort study. *Sex Transm Infect* 2017;93:460-6. doi: 10.1136/sextrans-2017-053113

22. Adachi K, Klausner JD, Bristow CC, et al. Chlamydia and gonorrhea in HIV-infected pregnant women and infant HIV transmission. *Sex Transm Dis* 2015;42(10):554-65. doi: 10.1097/OLQ.0000000000000340

23. Goodfellow L, Verwijs MC, Care A, et al. Vaginal bacterial load in the second trimester is associated with early preterm birth recurrence: a nested case-control study. *BJOG* 2021;128(13):2061-72. doi: 10.1111/1471-0528.16816

24. Bretelle F, Rozenberg P, Pascal A, et al. High *Atopobium vaginae* and *Gardnerella vaginalis* vaginal loads are associated with preterm birth. *Clin Infect Dis* 2015;60(6):860-7. doi: 10.1093/cid/ciu966

25. Freitas AC, Bocking A, Hill JE, et al. Increased richness and diversity of the vaginal microbiota and spontaneous preterm birth. *Microbiome* 2018;6(1):117. doi: 10.1186/s40168-018-0502-8

26. Goldenberg RL, Hauth JC, Andrews WW. Intrauterine infection and preterm delivery. *N Engl J Med* 2000;342(20):1500-7. doi: 10.1056/NEJM200005183422007

27. Andrews WW, Hauth JC, Goldenberg RL, et al. Amniotic fluid interleukin-6: correlation with upper genital tract microbial colonization and gestational age in women delivered after spontaneous labor versus indicated delivery. *Am J Obstet Gynecol* 1995;173(2):606-12. doi: 10.1016/0002-9378(95)90290-2

For peer review only - http://bmjopen.bmj.com/site/about/guidelines.xhtml

28. Medina-Marino A, Cleary S, Muzny CA, et al. Sexually transmitted infection screening to prevent adverse birth and newborn outcomes: study protocol for a randomized-controlled hybrid-effectiveness trial. *Trials* 2022;23(1):441. doi: 10.1186/s13063-022-06400-y

29. Vallely AJ, Pomat WS, Homer C, et al. Point-of-care testing and treatment of sexually transmitted infections to improve birth outcomes in high-burden, low-income settings: Study protocol for a cluster randomized crossover trial (the WANTAIM Trial, Papua New Guinea). *Wellcome Open Res* 2019;4:53. doi: 10.12688/wellcomeopenres.15173.2

30. Harris PA, Taylor R, Thielke R, et al. Research electronic data capture (REDCap) - A metadata-driven methodology and workflow process for providing translational research informatics support. *J Biomed Inform* 2009;42(2):377-81. doi: 10.1016/j.jbi.2008.08.010

31. South African National Department of Health. Guidelines for Maternal Care in South Africa: A manual for clinics, community health centres and district hospitals. Pretoria: Department of Health, 2016. https://knowledgehub.health.gov.za/elibrary/guidelines-maternity-care-south-africa-2016 (accessed 26 October 2023).

32. Peters RPH, Garrett N, Chandiwana N, et al. Southern African HIV Clinicians Society 2022 guideline for the management of sexually transmitted infections: Moving towards best practice. *South Afr J HIV Med* 2022;23(1):1450. doi: 10.4102/sajhivmed.v23i1.1450

33. World Health Organization. ICD-10 International statistical classification of diseases and related health problems. Geneva: World Health Organization, 2016. https://icd.who.int/training/icd10training/ICD-10%20training/ICD-10_Resources/ICD-10_Volume_1.pdf (accessed 26 October 2023).

34. March of Dimes, PMNCH, Save the children, et al. Born Too Soon: The Global Action Report on Preterm Birth. Geneva: World Health Organization, 2012. https://www.who.int/publications/i/item/9789241503433 (accessed 26 October 2023).

35. Altman DG, Royston P. The cost of dichotomising continuous variables. *BMJ* 2006;332(7549):1080. doi: 10.1136/bmj.332.7549.1080

36. Jordan J. Laboratory Procedure Manual: Bacterial Vaginosis in Swabs. Atlanta: Center for Disease Control and Prevention, 2004. https://www.cdc.gov/nchs/data/nhanes/nhanes_03_04/l34_c_met_bv.pdf (accessed 26 October 2023).

37. Nugent RP, Krohn MA, Hillier SL. Reliability of diagnosing bacterial vaginosis is improved by a standardized method of gram stain interpretation. *J Clin Microbiol* 1991;29(2):297-301. doi: 10.1128/jcm.29.2.297-301.1991

38. Edberg A, Jurstrand M, Johansson E, et al. A comparative study of three different PCR assays for detection of Mycoplasma genitalium in urogenital specimens from men and women. *J Med Microbiol* 2008;57(Pt 3):304-09. doi: 10.1099/jmm.0.47498-0

39. Ferandon C, Peuchant O, Janis C, et al. Development of a real-time PCR targeting the yidC gene for the detection of Mycoplasma hominis and comparison with quantitative culture. *Clin Microbiol Infect* 2011;17(2):155-9. doi: 10.1111/j.1469-0691.2010.03217.x

For peer review only

40. Frolund M, Bjornelius E, Lidbrink P, et al. Comparison between culture and a multiplex quantitative real-time polymerase chain reaction assay detecting Ureaplasma urealyticum and U. parvum. *PLoS One* 2014;9(7):e102743. doi: 10.1371/journal.pone.0102743

41. Pillay A, Radebe F, Fehler G, et al. Comparison of a TaqMan-based real-time polymerase chain reaction with conventional tests for the detection of Trichomonas vaginalis. *Sex Transm Infect* 2007;83(2):126-9. doi: 10.1136/sti.2006.022376

42. Fujita S, Lasker BA, Lott TJ, et al. Microtitration plate enzyme immunoassay to detect PCR-amplified DNA from Candida species in blood. *J Clin Microbiol* 1995;33(4):962-7. doi: 10.1128/jcm.33.4.962-967.1995

43. Shin JH, Nolte FS, Holloway BP, et al. Rapid identification of up to three Candida species in a single reaction tube by a 5' exonuclease assay using fluorescent DNA probes. *J Clin Microbiol* 1999;37(1):165-70. doi: 10.1128/JCM.37.1.165-170.1999

44. Villette R, Autaa G, Hind S, et al. Refinement of 16S rRNA gene analysis for low biomass biospecimens. *Sci Rep* 2021;11(1):10741. doi: 10.1038/s41598-021-90226-2

45. Holm JB, Humphrys MS, Robinson CK, et al. Ultrahigh-Throughput Multiplexing and Sequencing of >500-Base-Pair Amplicon Regions on the Illumina HiSeq 2500 Platform. *mSystems* 2019;4(1) doi: 10.1128/mSystems.00029-19

46. van de Wijgert J, Verwijs MC, Agaba SK, et al. Intermittent Lactobacilli-containing Vaginal Probiotic or Metronidazole Use to Prevent Bacterial Vaginosis Recurrence: A Pilot Study Incorporating Microscopy and Sequencing. *Sci Rep* 2020;10(1):3884. doi: 10.1038/s41598-020-60671-6

47. Callahan BJ, McMurdie PJ, Rosen MJ, et al. DADA2: High-resolution sample inference from Illumina amplicon data. *Nat Methods* 2016;13(7):581-3. doi: 10.1038/nmeth.3869

48. Pruesse E, Quast C, Knittel K, et al. SILVA: a comprehensive online resource for quality checked and aligned ribosomal RNA sequence data compatible with ARB. *Nucleic Acids Res* 2007;35(21):7188-96. doi: 10.1093/nar/gkm864

49. Kyongo JK, Crucitti T, Menten J, et al. Cross-Sectional Analysis of Selected Genital Tract Immunological Markers and Molecular Vaginal Microbiota in Sub-Saharan African Women, with Relevance to HIV Risk and Prevention. *Clin Vaccine Immunol* 2015;22(5):526-38. doi: 10.1128/CVI.00762-14

50. Breiman L. Classification and regression trees. Monterey: Wadsworth & Brooks/Cole Advanced Books & Software 1984. doi: 10.1201/9781315139470

51. Breiman L. Random Forests. *Machine Learning* 2001;45:5-32. doi: 10.1023/A:1010933404324

For peer review only

For peer review only - http://bmjopen.bmj.com/site/about/guidelines.xhtml

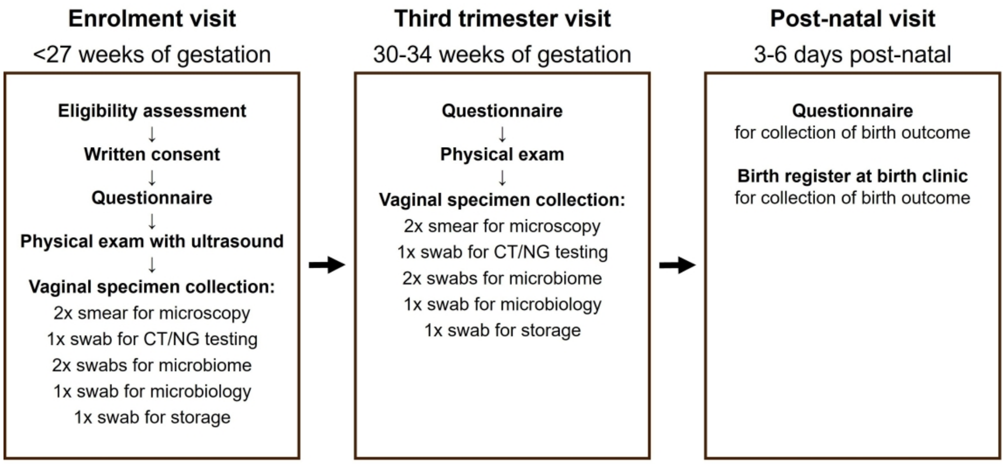

Figure 1 Study visits and specimen collection
Abbreviations: CT, Chlamydia trachomatis; NG, Neisseria gonorrhoeae.

272x125mm (330 x 330 DPI)

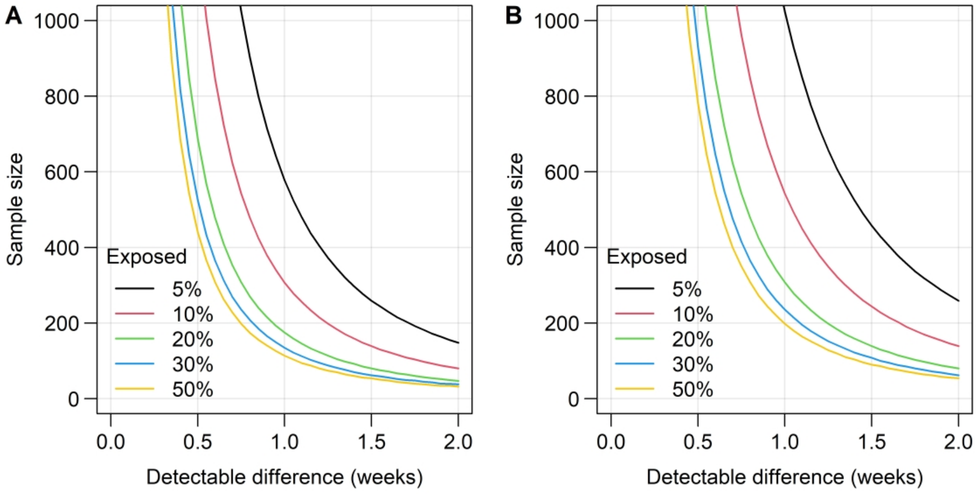

Figure 2 Sample size requirements at different levels of exposure prevalence with power of 80% and alpha 0.83% based on Student's t-test.
Legend: panel A, standard deviation 1.5; panel B, standard deviation 2.0. The curves for % exposed are symmetrical around a prevalence of 50%, i.e., curve for 10% exposed is same as that for 90% exposed.

516x258mm (236 x 236 DPI)

Supplemental online file – study questionnaire

Genital tract infections, the vaginal microbiome and gestational age at birth among pregnant women in South Africa: a cohort study protocol

Ranjana M S Gigi,[1 2] Mandisa M Mdingi,[2] Hyunsul Jung,[3] Shantelle Claassen Weitz,[4] Lukas Bütikofer,[5] Jeffrey D Klausner,[6] Christina A Muzny,[7] Christopher M Taylor,[8] Janneke H H M van de Wijgert*,[9] Remco P H Peters*,[2 3 4] Nicola Low*[1]

1) Institute of Social and Preventive Medicine, University of Bern, Bern, Switzerland

2) Research Unit, Foundation for Professional Development, East London, South Africa

3) Department of Medical Microbiology, University of Pretoria, Pretoria, South Africa

4) Department of Pathology, University of Cape Town, Cape Town, South Africa

5) CTU Bern, Department of Clinical Research, University of Bern, Bern, Switzerland

6) Keck School of Medicine, University of Southern California, Los Angeles, USA

7) Division of Infectious Diseases, University of Alabama at Birmingham, Birmingham, USA

8) School of Medicine, Louisiana State University, USA

9) Julius Center for Health Sciences and Primary Care, University Medical Center Utrecht, Utrecht University, Utrecht, Netherlands

For peer review only

Screening and Enrolment

Record ID

Site Information

Today's Date

Study Staff Name

Start Time

Please select Study Site Name

◯ Grey Gateway
◯ Duncan Village CHC
◯ Nontyatyambo CHC
◯ Gompo
◯ Ndevana

Introduction to the Study:

Note to RA:

In this section you will be introducing the study to the participant. Please make sure to execute the following steps:

1. Introduce the study

◯ Proceed

Does the participant show interest in the study

◯ Yes the participant shows interest
◯ No the participant is not interested in the study

END

The participant is not interested in the study. Thank them for their time.

For peer review only

Eligibility Screening

Note to RA:
The participant seems to show interest in the study. We need to determine their eligibility status. You will ask a series of questions to determine this. Please select "Proceed" to continue.

◯ Proceed

Is the participant currently living in BCM?

◯ Yes
◯ No

Is the participant 18 years or older?

◯ Yes
◯ No

Please specify the participant's date of birth ______________________

Calculated age ______________________

Is this the participant's first ANC visit?

◯ Yes
◯ No

Is the participant within the first 26 weeks of her pregnancy?

◯ Yes
◯ No

Is the participant within the first 20 weeks of her pregnancy?

◯ Yes
◯ No

Gestational weeks ______________________
(if unknown, enter 99)

Is the participant intending to deliver the baby at one of our collaborating MOUs?

◯ Yes
◯ No

Is the participant currently involved in any other ANC/HIV research trial?

◯ Yes
◯ No

For peer review only

Calculated Eligibility Outcome

____
(1 = Eligible, 0 = Not Eligible)

END
The participant is not eligible for our research study

This will be the end of their participation. Please thank them for their time.

ELIGIBLE
The participant is eligible for our research study. Please select "Proceed" to start with the consenting process.

◯ Proceed

Consenting Process:

NOTE TO RA:
You will now start with the consenting process. Please make sure to do the following:

1. Read the consent form with the participant
2. Read in a language they prefer
3. Allow for questions
4. If willing to consent, sign all documents
5. Hand a signed copy (without PIN) to the patient

◯ Proceed

Did the participant provide a signed consent to participate in the research study?

◯ Yes
◯ No

Consent refusals

Reasons for refusal

☐ They have no time
☐ Scared
☐ In a different study
☐ Other

If "Other", please specify

For peer review only - http://bmjopen.bmj.com/site/about/guidelines.xhtml

Refusal date

END
Thank the participant for their time

Provided Consent

Consent date

Participant PIN

CONSENTED
The participant has agreed to provide consent. You will now allocate a study PIN to the participant. Please use the next available PIN on the hard copy enrollment log

◯ Proceed

Participant PIN

Participant PIN Verification

Pin match

PIN valid

ERROR
The PINs you entered did not match up

You have entered the following PINs

first pin: [participant_pin]
second pin: [participant_pin_verify]

ERROR
The PIN you entered is invalid for [site_name]

You have entered the following PINs

first pin: [participant_pin]
second pin: [participant_pin_verify]

For peer review only - http://bmjopen.bmj.com/site/about/guidelines.xhtml

Saving Instruction

You have completed the Screening and Enrollment process. Please make sure to check if all relevant fields have been selected and the information captured is accurate.

Once this is done, please select the "complete" option below and then select "Save & Exit".

Once you have done this you will be directed to the baseline Data.

Notes

Additional Notes

For peer review only

Baseline Data

Staff name ______________________

Today's Date ______________________

Start Time ______________________

Sociodemographics

NOTE TO RA:
You are about to start the Socio-demographics section. Please make sure to ask the questions as they appear on your tablet.

Please select "Proceed" to continue.

◯ Proceed

Sociodemographics

How would you describe yourself in terms of race?

◯ African
◯ Coloured
◯ Mixed Race
◯ White
◯ Indian
◯ No answer

What level of education did you complete?

◯ Less than Gr. 10
◯ Gr.10 or 11
◯ Gr.12
◯ Diploma
◯ Degree
◯ Refused to answer

For peer review only

Which best describes the type of house in which you live? Please choose one answer only:

- ◯ House or brick structure on a separate stand or yard or on a farm
- ◯ Traditional dwelling/hut/structure made of traditional materials
- ◯ Flat
- ◯ Town/cluster/semi-detached house (simplex, duplex or triplex)
- ◯ Unit in retirement village
- ◯ Dwelling/house/flat/room in backyard
- ◯ Informal dwelling/shack IN the backyard of a formal house
- ◯ Informal dwelling/shack NOT in backyard e.g. in an informal/squatter settlement or on farm
- ◯ Room/flatlet not in backyard but on a shared property e.g granny flat
- ◯ Caravan/tent
- ◯ Worker's hostel
- ◯ Other

If other, please specify.

What is the main material of your house walls? Please choose one answer only:

- ◯ Bricks & plaster/finished
- ◯ Bare brick/cement block
- ◯ Corrugated iron/zinc
- ◯ Wood
- ◯ Plastic
- ◯ Cardboard
- ◯ Mixture of mud and cement
- ◯ Wattle and daub
- ◯ Mud
- ◯ Other

If other, please specify

What is the main material of your house roof? Please choose one answer only:

- ◯ Tiles
- ◯ Corrugated iron/zinc
- ◯ Thatching
- ◯ Asbestos
- ◯ Plastic
- ◯ Cardboard
- ◯ Other

If other, please specify

For peer review only

What is your current relationship status?

◯ Married
◯ Steady partner
◯ Steady partner and Casual Partner(s)
◯ Casual Partner(s)
◯ No relationship

Do you live together with your partner?

◯ Yes
◯ No

Are you currently employed?

◯ Employed
◯ Self employed
◯ Not employed

What is your monthly personal income?

◯ None
◯ < 1000 ZAR per month
◯ 1001 - 5000 ZAR per month
◯ 5001 - 10 000 ZAR per month
◯ >10 000 ZAR per month

What is your household's main source of income?

◯ Personal income from employment \ self employment
◯ Income from partner
◯ Grants
◯ Other

Have you been outside of the Eastern Cape or country in the past 6 months?

◯ Yes
◯ No

Which provinces or country have you been to in the last 6 months?

Note to RA: please select all that apply

☐ Free State
☐ Gauteng
☐ Kwazulu-Natal
☐ Limpopo
☐ Mpumalanga
☐ Northern Cape
☐ North West
☐ Western Cape
☐ Outside South Africa

Has your partner/husband been outside of the Eastern Cape or country in the past 6 months?

◯ Yes
◯ No

For peer review only

Which provinces or country has your partner/husband been to in the last 6 months?

Note to RA: Please select all that apply

☐ Free State
☐ Gauteng
☐ Kwazulu-Natal
☐ Limpopo
☐ Mpumalanga
☐ Northern Cape
☐ North West
☐ Western Cape
☐ Other country

What is the main source of drinking water for your household? Please choose one answer only:

◯ Piped (tap) water in dwelling
◯ Piped (tap) water on site or in yard
◯ Neighbour's tap
◯ Public or communal tap (either free or paid)
◯ Borehole on site
◯ Borehole off site/communal
◯ Rain water tank
◯ Water carrier/tanker
◯ Flowing water/stream/river
◯ Stagnant water/dam/pool
◯ Well
◯ Spring
◯ Bottled water
◯ Other

If other, please specify

What type of toilet does your household use? Please choose one answer only:

◯ Flush toilet (connected to sewage)
◯ Flush toilet (with septic tank)
◯ Chemical toilet
◯ Pit latrine with ventilation pipe
◯ Pit latrine without ventilation pipe
◯ Bucket toilet
◯ No facility/bush/field
◯ Other

If other, please specify

For peer review only

What is the main source of energy for cooking in your household? Please choose one answer only:

◯ Electricity from mains
◯ Electricity from generator
◯ Gas
◯ Paraffin
◯ Wood
◯ Coal
◯ Animal dung
◯ Solar energy
◯ Other

If other, please specify

Does your household have any of the following items in good working order? Read each item and indicate the presence of each

	Yes	No
Television	◯	◯
Gas or Electric stove	◯	◯
Fridge/freezer	◯	◯
Private motor vehicle in running condition	◯	◯
Bicycle	◯	◯
Bed	◯	◯
Sofa or sofa set	◯	◯
Kitchen sink	◯	◯

Do you think that you will need to borrow money to pay for healthcare during your pregnancy?

◯ Yes
◯ No

How much money did you spend coming to the clinic today (including transport costs, snacks while waiting etc.) ?

____
([RANDS])

Did you lose any money from your job because of coming to the clinic today?

◯ Yes
◯ No

If yes, how much money did you lose?

____
([RANDS])

How much time did you spend travelling to the clinic today (Hours)?

____
([HOURS])

For peer review only - http://bmjopen.bmj.com/site/about/guidelines.xhtml

How much time did you spend travelling to the clinic today (Minutes)?

____
([MINUTES])

Time spent travelling in minutes.

____

How much time do you normally spend waiting and seeing the doctor or nurse in a clinic such as this one (Hours)?

____
([HOURS])

How much time do you normally spend waiting and seeing the doctor or nurse in a clinic such as this one?

____
([MINUTES])

How much time do you normally spend waiting and seeing the doctor or nurse in a clinic such as this one in minutes?

____
([MINUTES])

Are you planning to wait for your results today?
(New question added @13/09/2022)

◯ Yes
◯ No

What is your main reason why you are not intending to wait today?
(New question added @13/09/2022)

◯ Have to get to work
◯ Have to get back to my kids/family
◯ Want to go to the shop
◯ Transport availability
◯ Lack of privacy
◯ Hungry
◯ No space to wait
◯ Not feeling well
◯ Boring
◯ Other

If "Other" , please specify.
(New question added @13/09/2022)

What would make you change your mind?
(New question added @13/09/2022)

For peer review only

Do you do any of the following activities in a lake / stream?

Note to RA: Please select multiple that apply

☐ Play
☐ Bath
☐ Wash blankets
☐ Do laundry
☐ Fish
☐ Collect water
☐ Crossing
☐ None

Behavioural Questionnaire

NOTE TO RA:
You just completed all questions related to socio-demographics. You are about to start with the Behavioral Questionnaire.

Please select "Proceed":

◯ Proceed

When was the last time you had sex?

◯ In the past week
◯ In the past month
◯ More than a month ago

Did you use a condom the last time you had sex?

◯ Yes
◯ No

Do you use a lubricant?

◯ Yes
◯ No

Can you please elaborate on the type of lubricant that you use?

____

What do you use to clean your vagina?

◯ Water only
◯ Soap and water
◯ Other household products
◯ Other

Please specify what other things you used on your vagina.

For peer review only

Do you do any vaginal douching?

◯ Yes
◯ No

Please specify

Do you do any form of vaginal cleansing?

◯ Yes
◯ No

Please specify

Do you use anything to clean inside your vagina?

◯ Yes
◯ No

Do you insert anything in your vagina for tightening?

◯ Yes
◯ No

Please specify

In the past 6 months, how many sexual partners did you have?

◯ One
◯ More than one

In the past 6 months, have you engaged in any of the following?
(Select ALL that apply)

☐ Vaginal sex
☐ Oral sex
☐ Anal sex

Have you recently agreed to sex even though you did not feel like to?

◯ Yes
◯ No

For peer review only

Note to RA: Discuss with participant counselling options

◯ Yes
◯ Participant doesn't need counselling

Please specify

In the past 6 months, have you been forced to have sex with anyone?

◯ Yes
◯ No

Note to RA: Discuss with participant counselling options

◯ Yes
◯ Participant doesn't need counselling

Please specify

In the past 6 months, have you received any benefits (money or goods) for sex?

◯ Yes
◯ No

Do you suspect your steady partner to have any other sex partners?

◯ Yes
◯ No
◯ Unsure

When did your last menstrual period start?

Note to RA: Please ask participant to give the most accurate date.

____

Just before I became pregnant.

NOTE: Please tick the statement that most applies to you:

◯ I wanted to have a baby
◯ I had mixed feelings about having a baby
◯ I did not want to have a baby

How many times have you been pregnant before your current pregnancy?

____

How many live children have you delivered?

____

For peer review only

Of the live births that you had, how many were normal vaginal delivery?

____

Of the live births that you had, how many were "emergency cesarean"?

____

Of the live births that you had, how many were "elective cesarean"?

____

Live birth Match

____

Note to RA: The numbers you have entered do not match. Please check again.

How many of your live birth's were premature?

____

How many of your live birth's were full term?

____

Delivery timing calc

____

Note: The numbers you have captured do not add up

Have you ever had an ectopic pregnancy?

◯ Yes
◯ No

Have you ever had a miscarriage?

◯ Yes
◯ No

Have you ever had a stillbirth?

◯ Yes
◯ No

For peer review only

Do you smoke cigarettes?

◯ Yes
◯ No

Have you used any of the following since you found out you were pregnant? (select multiple)
(Select ALL that apply.)

☐ Alcohol
☐ Tik
☐ Dagga
☐ Grandpa
☐ Other
☐ None

Please specify

Do you know your current HIV status?

◯ HIV negative (tested today by clinical staff)
◯ HIV positive on ART
◯ HIV positive, not on ART
◯ Don't know (never tested)
◯ Don't know (no yet tested today)

Was the participant newly diagnosed within the past week?

◯ Yes
◯ No

Can we test you for HIV today?

◯ Yes
◯ No

Unknown HIV Status:

Note to RA/ Nurse: HIV test needs to be conducted

◯ Proceed to test

HIV rapid test result:

◯ Positive
◯ Negative

HIV confirmatory test

◯ Positive
◯ Negative

Elisa blood barcode

____

For peer review only - http://bmjopen.bmj.com/site/about/guidelines.xhtml

Have you ever been treated for an STI in the last year?

◯ Yes, I had discharge
◯ Yes, I had ulcers
◯ Yes, I had genital warts
◯ Yes, no symptoms but notified by partner
◯ No

Does the participant have pre-existing diabetes?

◯ Yes
◯ No

Are you on treatment for your diabetes?

◯ Yes
◯ No

Does the participant have pre-existing hypertension?

◯ Yes
◯ No

Are you currently on medication for your hypertension?

◯ Yes
◯ No

Does the participant have pre-existing thyroid disease?

◯ Yes
◯ No

Is the participant taking medication for her thyroid disease?

◯ Yes
◯ No

Do you know your partner's HIV status?

◯ Yes, HIV positive on ART
◯ Yes, HIV positive but not on ART
◯ Yes, HIV negative
◯ No

You have completed the baseline questionnaire. Please make sure to log out of your REDCap account.

Once you have done this you can hand the process over to the nurse who will conduct the clinical history.

For peer review only

NOTES

Additional notes

For peer review only

Physical Exam

Staff Name ____________________

Today's Date ____________________

Start time ____________________

You are about to administer the questions associated with the physical exam.

Please select "Proceed" to continue.

◯ Proceed

Clinical History

Do you currently have any of the following symptoms?

RA: Please select all that apply

- ☐ Abnormal vaginal discharge
- ☐ Pain during urination
- ☐ Lower abdominal pain
- ☐ Pain related to intercourse
- ☐ Vaginal bleeding related to intercourse
- ☐ Genital itchiness
- ☐ Any skin abnormalities
- ☐ None

How many days ago did your abnormal vaginal discharge start?

____

How many days ago did the pain during urination start?

____

Provide further details

Have you received treatment for these symptoms?

◯ Yes
◯ No

For peer review only

Where did you get the treatment from?

◯ Over the counter
◯ Healthcare facility
◯ Traditional healer

Please provide further details

If you were told you had an STI would you disclose to your partner(s)?

◯ Yes, to steady partner
◯ Yes, to casual partner(s)
◯ Yes, to all steady and casual partner(s)
◯ No

Co-Morbidities

You are about to start asking questions related to co-morbidities.

Please select "Proceed" to continue.

◯ Proceed

Did the participant screen positive for any TB symptoms?

◯ Yes
◯ No

The participant shows signs of TB. A specimen needs to be collected for further testing. Please select below to specify whether a specimen was collected successfully.

◯ Yes
◯ No

Instruction: Please record the specimen tracking number below

____

Are you on cotrimoxazole prophylaxes?

◯ Yes, on cotrimoxazole
◯ Yes, started today
◯ No

Did the participant start antiretroviral therapy today?

◯ Yes
◯ No

Specify reason for not starting

For peer review only

Was blood taken today for the participant's CD4 count?

◯ Yes
◯ No

Please record barcode for blood tube for CD4 count testing?

____

Is the participant's most recent CD4 count within the last 12 months available?

◯ Yes
◯ No

What was the date of the CD4 specimen collection?

____

What was the participant's most recent CD4 count?

____
(if no number listed, enter 9999)

Was blood taken today for the participant's viral load?

◯ Yes
◯ No

Please record barcode for blood tube for viral load testing?

____

Is the participant's most recent viral load within the past 12 months available?

◯ Yes
◯ No

What was the date of the viral load specimen collection?

____

What was the participant's most recent viral load?

____
(if no number listed, enter 0000)

Was blood taken today for the participant's viral load?

◯ Yes
◯ No

Please record the barcode for viral load testing?

____

For peer review only

Is the participant's most recent viral load available?

◯ Yes
◯ No

What was the date of the viral load specimen collection?

____

What was the participant's most recent viral load?

____

Which regimen for ART were you started on today?

◯ TLD
◯ TEE
◯ AZT/3TC/LPV
◯ Other

Which regimen for ART were you on so far?

◯ TLD
◯ TEE
◯ AZT/3TC/LPV
◯ Other

Has the regimen for ART been changed today?

◯ Yes
◯ No

To which regimen for ART has it been changed today?

◯ TLD
◯ TEE
◯ AZT/3TC/LPV
◯ Other

Please select "Proceed" to collect blood for viral load testing.

◯ Proceed

Did you successfully collect blood for viral load testing?

◯ Yes
◯ No

Please capture the barcode associated with the blood tube used for testing viral load.

____

For peer review only

Was a CD4 count test done?

◯ Yes
◯ Yes, but no result available
◯ Not done

Please specify last known CD4-cell count.

____

Was blood taken today for the participant's CD4 count ?

◯ Yes
◯ No

Please record the barcode for CD4 testing?

____

Is the participant's most recent CD4 available?

◯ Yes
◯ No

What was the date of the CD4 specimen collection?

____

What was the participant's most recent CD4 Count?

____

Has a syphilis test been done for the participant?

◯ Yes
◯ No

Instruction: Please conduct a rapid test for syphilis.

Specify if the participant agreed to testing / you managed to execute the test.

◯ Yes
◯ No

Which syphilis test have you used?

◯ Alere Syphilis TP (provided by FPD)
◯ HIV/Syphilis Duo (provided by FPD)
◯ No rapid test used only NHLS bloods for RPR
◯ Other please specify

Please specify

For peer review only

Syphilis result

◯ Positive
◯ Negative
◯ Indeterminate

Titer value 1:

____

Please collect blood for further syphilis testing and specify if the blood was collected successfully.

◯ Yes
◯ No

Treatment given

◯ Benzathine penicillin, 2.4 mU
◯ Out of stock

Please contact the study clinician and specify treatment given to participant

Please capture the barcode below.

____

Participant weight in kilograms

____

Participant height in cm

____

Participant systolic blood pressure

____

Participant diastolic blood pressure

____

How was Hemoglobin measured?

◯ Hb meter at the clinic
◯ Hb at NHLS

Please capture Hb result

____
(1 Decimal Place)

For peer review only - http://bmjopen.bmj.com/site/about/guidelines.xhtml

Please capture the barcode for Hb

____

Participant MUAC in cm

____
(1 Decimal Place)

Please collect participant's urine for later testing.

Ultrasound Results

You are about to start capturing information pertaining to the ultrasound results.

Please select "Proceed" to continue.

◯ Proceed

Ultra-sound scan date

____

Was the pregnancy confirmed?

◯ Yes, intra-uterine
◯ Yes, extra-uterine
◯ No

NOTE: Please refer immediately

NOTE
Due to the status of the pregnancy, the participant is no longer eligible to continue with the study. This is the end of their involvement in the study. Please thank them for their time. Also do the following:

- Save and Exit the form

- Complete a Study Note confirming termination of study participation

Please specify the number of foetus

____

For peer review only - http://bmjopen.bmj.com/site/about/guidelines.xhtml

You are about to capture the gestational age of the foetuses. Please select "Proceed" and then capture the number of weeks followed by days.

◯ Proceed

Gestational age in weeks

____

Gestational age in days

____

Calc: Gestational age in days

____

EDD based on ultra-sound

____

Calc: Days to EDD

____

Calc assist for EDD

The number of days must be equal to 280.

____

Note to Nurse:

You did not enter either gestational age or EDD correctly.

Calc: Eligibility __________________________________

END
The participant is not eligible for our research study

This will be the end of their participation. Please thank them for their time.

STI Clinical Examination (To be done By Nurse)

You are about to capture information related to STI clinical examination.

Please select "Proceed" to continue

◯ Proceed

For peer review only

During your examination, were there any signs of abnormal vaginal discharge?

◯ Yes
◯ No

During your examination, were there any signs of inguinal lymphadenopathy?

◯ Yes
◯ No

Are these bubo?

◯ Yes
◯ No

Note to RA: Please contact study clinician and specify treatment given to participant

During your examination, were there any signs of lower abdominal pain?

◯ Yes
◯ No

During your examination, where there any signs of scratch marks?

◯ Yes
◯ No

During your examination, where there any signs of skin conditions?

◯ Yes
◯ No

Please specify the nature of the skin conditions

During your examination, where there any other observations that need to be noted?

For peer review only

For peer review only - http://bmjopen.bmj.com/site/about/guidelines.xhtml

Urine Dipstick test results

You are about to capture the results from the dipstick testing

Please select "Proceed" to continue

◯ Proceed

Blood - Hemoglobin

◯ Negative
◯ Ca. 10
◯ Ca. 50
◯ Ca. 250/300

Blood - Erythrocytes

◯ Negative
◯ Ca. 5 -10
◯ Ca. 50
◯ Ca. 250/300

Urobilinogen

◯ Normal
◯ 2
◯ 4
◯ 8
◯ 12

Bilirubin

◯ Negative
◯ 1 plus
◯ 2 plus
◯ 3 plus
◯ Not available

Protein

◯ Negative
◯ 30
◯ 100
◯ 500

Nitrate

◯ Negative
◯ Positive

For peer review only

Keton

◯ Negative
◯ 1 plus
◯ 2 plus
◯ 3 plus
◯ Not available

Glucose

◯ Negative
◯ Normal
◯ 50
◯ 150
◯ 500
◯ ≥1000

pH

◯ 5
◯ 6
◯ 7
◯ 8
◯ 9
◯ Not available

SG

◯ 1.000
◯ 1.005
◯ 1.010
◯ 1.015
◯ 1.020
◯ 1.025
◯ 1.030
◯ Not available

Leucocytes

◯ Negative
◯ 25
◯ 75
◯ 500

NOTE
The participant's clinical gestational age is more than 20 weeks. They are not eligible to proceed with the study activities.

Please do the following:

1. Explain the reason for study termination
2. Complete the study electronic termination tool
3. Complete the study termination document and place in file

◯ End

For peer review only

NOTES

Additional Notes

You have completed capturing the information from the clinical exam. Please make sure to check that you have completed all the fields.

Please select "Complete" then "Save and Exit".

You will now proceed to collecting study specimens and randomization

For peer review only

Specimens and Randomization

Staff Name ______________________

Today's Date ______________________

Start time ______________________

Specimen Collection

NOTE
You will now start with the process of specimen collection. You will need to collect several specimens from the participant. These specimens need to be collected in the order in which they are presented here. The outcome of the randomization will have an impact on whether these specimens will be tested immediately or whether they will need to be prepared for storage.

The following specimens will need to be collected:
1. Vaginal loop to be used to prepare two slides
2. Vaginal swab to be used for STI testing
3. Vaginal swab to be used for profiling
4. Vaginal swab to be used for microbiome
5. Vaginal swab to be used for cytokine

◯ Done

NOTE
You will now start with the process of specimen collection. You will need to collect several specimens from the participant. These specimens need to be collected in the order in which they are presented here.

The following specimens will need to be collected:
1. 1 x Vaginal loop to be used to prepare two slides
2. 1 x Vaginal swab to be used for STI testing
3. 1 x Vaginal swab to be used for profiling
4. 2 x Vaginal swab to be used for microbiome
5. 1 x Vaginal swab to be used for cytokine

◯ Done

Vaginal Smear

Please specify the vaginal pH

____
(if not available, enter 99)

For peer review only

Please select which pH strips are used to measure vaginal pH

◯ CardinalHealth pH Indicator Strips (range 3.6-6.1)
◯ pH Indicator Strips pH 0-14
◯ Natureland vaginal pH test (range 3.5-6.5)

You will need to use a single loop to collect vaginal smear on two glass slides for microscopy

◯ Done

Confirm the PIN associated with the first vaginal slide that will be used for Nugent score

◯ [participant_pin]-S1

Confirm the PIN associated with the second vaginal slide that will be used for yeast microscopy

◯ [participant_pin]-S2

Vaginal Swabs

NOTE
You will now collect four vaginal swabs. They will be used as follows:

1. STI testing (test for arms 1 and 2, store for arm 3)
2. Profiling (stored)
3. Microbiome (stored)
4, Cytokine

◯ Done

NOTE
You will now collect four vaginal swabs. They will be used as follows:

1. STI testing
2. Profiling (stored)
3. Microbiome (stored)
4, Cytokine

◯ Done

Please confirm the PIN associated with the urine for Schistosomiasis testing.
(2022/10/21 - Stopped collecting the urine specimen)

◯ [participant_pin] - UD1

Please confirm the PIN associated with the vaginal swab that will be used for STI testing.

◯ [participant_pin] - BV1

Please confirm the PIN associated with the vaginal swab that will be used for profiling.

◯ [participant_pin] - BV2

Please confirm the PIN associated with the vaginal swab that will be used for microbiome.

◯ [participant_pin] - BV3

For peer review only

Please confirm the PIN associated with the vaginal swab that will be used for cytokines.

◯ [participant_pin] - BV4

NOTE
You have finished the collection of the vaginal swabs. Please ensure specimens have been prepared for storage and shipment. The vaginal swab that is collected for STI testing should be kept aside following the outcome of the randomization. If the participant is in arm 1 or 2 the specimen should be used for immediate testing. If however, the participant is randomized to arm 3, you can store the specimen.

Please select "Proceed" to start the process of randomization

◯ Done

Randomization

◯ Arm 1
◯ Arm 2
◯ Arm 3

Activities Associated with "[randomization]"

NOTE
The participant has been randomized to "[randomization]". You will now need to do the following:

1. Prepare the STI swab for testing using the GeneXpert

◯ Done

NOTE:
The participant has been randomized to"[randomization]". You will now need to do the following:

1. Prepare the STI swab for testing using the GeneXpert
2. Screen for symptoms
3. Provide treatment and partner referral if positive

◯ Done

NOTE:
The participant has been randomized to "[randomization]". You will now need to do the following:

1. Screen for symptoms
2. Provide treatment and partner referral if positive

◯ Done

NOTE
You will now need to do the following:

1. Prepare the STI swab for testing using the GeneXpert

◯ Done

STI Results

	Positive	Negative
CT	◯	◯

For peer review only

NG	◯	◯
TV	◯	◯

NOTE: See Calculation:

The result from the STI test?

____
(0 = Negative, 1 = Positive, 2 = No result)

Date the result was obtained

____

Did the participant wait for her STI results?
(New question added @ 03/11/2022)

◯ Yes
◯ No

Symptomatic Screening Outcome Following Negative Test

The result from the GeneXpert was negative.

Was the participant reporting STI symptoms or showed symptoms during the clinical assessment?

◯ Yes
◯ No

Does the participant report any medication allergies?

◯ Yes
◯ No

Please contact study clinician before giving any treatment. Please specify discussed medication allergies and treatment plan with study clinician

The following treatment has been provided

☐ Azithromycin 1g stat dose
☐ Azithromycin 2g stat dose
☐ Ceftriaxone 250mg IM injection
☐ Ceftriaxone 1g IM injection
☐ Metronidazole 400mg bd x 1 week
☐ Metronidazole 2g stat dose
☐ Clotrimazole pessary and/or cream
☐ Trimethoprim/sulfamethoxazole 400/80 mg 2 tbl. bds for 5 days (bactrim)
☐ Ceftriaxone 500mg IM injection

Date treatment given

____

For peer review only

For peer review only - http://bmjopen.bmj.com/site/about/guidelines.xhtml

What made you change your mind about waiting for the results?
(New question added @13/09/2022)

Partner notification provided

◯ Yes, 1
◯ Yes, multiple
◯ No

Please explain why the partner notification note was not provided?

ELIGIBLE
The participant is eligible for our nested chlamydia case-control study. Please select "Proceed" to start with the consenting process.

◯ Proceed

Did the participant provide a signed consent to participate in the chlamydia case control study?

◯ Yes
◯ No

Reasons for refusal

☐ They have no time
☐ Scared
☐ In a different study
☐ Other

If "Other", please specify

Consent or refusal date

____

NOTES

Participant successfully enrolled

____

Additional notes

For peer review only

You are done with all activities associated with "[randomization]". Please hand the tablet over to the RA to capture the remaining schedule dates.

You are done with all activities. Please hand the tablet over to the RA to capture the remaining schedule dates.

For peer review only

Scheduling

Scheduling of Dates Associated with [randomization].

NOTE
You are about to schedule dates associated with [randomization] participants.

Please select "Proceed".

◯ Proceed

Scheduling of Dates Associated

NOTE:
You are about to schedule dates associated with microbiome participants.

Please select "proceed"

◯ Proceed

Scheduling Dates for 3-Week ToC

NOTE: The participant tested positive and therefore we need to schedule a date, exactly 3-weeks from today to conduct a test-of-cure.

Calculator Assist

The number here must be equal to 21

____

Scheduling the 3-week ToC

NOTE:
Please schedule a date, 3 weeks from today treatment given. Please use the calculator assistance to ensure that you schedule a date exactly 21 days from today.

____

ERROR
The field does not equal to 21, please change it

Have you handed the TOC date to the participant?

◯ Yes
◯ No

Scheduling Dates Associated with ToC Reminder

Schedule date for REMINDER of 3-week ToC visit

____

For peer review only

Calculator Assist for scheduling ToC reminder date

The reminder phone call will be made 18 days following the treatment date. The number of days need to equal to 18.

____

ERROR
You did not enter the date correctly. The number should equal to 18. Please redo the date.

Scheduling Dates Associated with 3-Week ToC Missed Visit Date

NOTE:
You have successfully scheduled the reminder date.

Please select "proceed" to schedule the missed visit date for the 3 week ToC visit.

◯ Proceed

Schedule the date for the MISSED VISIT of the ToC visit.

This date should be 3 weeks after the date on which the participant received their test result.

____

Calculator Assist for scheduling 3-week ToC Missed Visit

The participant's time period allowed for attending a ToC will start 35 days after they received their result and will close 35 days after the date they received their result.

The number here must show 35

____

ERROR
You did not enter the date correctly. The number should equal to 35. Please redo the date.

NOTE:
You have successfully scheduled the 3-week ToC close date

Please select "proceed" to start scheduling the next visit dates

◯ Proceed

Dates Associated with reminder for the 28 Week call

NOTE:
You are about to schedule dates for the call reminder at 28 weeks.

Please select "Proceed".

◯ Proceed

Note:
Schedule the date for the 28 week call. We will contact each participant to ask the date for their 30 weeks clinic visit is.

____

For peer review only

Calculation assist for scheduling the 32-week reminder date.

This number must equal to 196

____

Days to call reminder

____

ERROR
The number you have entered does not match 196. Please select a different date so that the number equals to 196.

CONGRATULATIONS

You have finished scheduling all dates.

NOTES

Notes box

For peer review only

BL specimens results

Staff

____

Date

____

Time

____

You are about to capture results of specimens collected during the baseline visit.

Please select "Proceed" to continue

◯ Proceed

Hb results received

◯ Yes
◯ No

Please capture Hb result

____

Please capture the barcode for Hb

____

Please capture the results of the sputum for TB testing

◯ MTB Negative
◯ MTB Positive Rifampicin Susceptible
◯ MTB Positive Rifampicin Resistant
◯ Not suitable
◯ Specimen missing
◯ Invalid
◯ Not applicable

Please contact participant

Please recollect specimen on participant next visit

CD4 count results received?

◯ Yes
◯ Clotted blood
◯ Missing

Please recollect blood or collect outcome from ART clinic

Please record the barcode for CD4 count testing

____

For peer review only

Please capture the result of the blood tube collected for CD4 count testing

____

Date sample for CD4 count was taken

____

Viral load results received?

◯ Yes
◯ Clotted blood
◯ Missing

Please recollect blood or collect outcome from ART clinic

Please capture the result of the blood tube collected for viral load testing

____

Date sample for viral load was taken

____

Please record barcode for viral load testing

____

Please capture the result of the syphilis testing

◯ RPR Negative
◯ RPR Positive
◯ RPR Indeterminate
◯ Not received

Please contact participant

____

Notes

Notes

For peer review only

UP Specimen Results

Staff ______________________________

Date ______________________________

Time ______________________________

You are about to capture results of specimens collected during the baseline visit.

Please select "Proceed" to continue

◯ Proceed

Please capture the result for the Nugent score testing

◯ Slide reading not satisfactory
◯ Slide reading was satisfactory

____

Please capture the result for the Nugent score testing

____

Please specify if yeast was present

◯ Yes
◯ No

Please capture the result for the yeast infection testing

◯ Slide reading not satisfactory
◯ Slide reading was satisfactory

____

Please capture the result for the Yeast Infection testing

____

Please specify if candida was present

◯ Yes
◯ No

For peer review only

Please capture the result for the schistosomiasis

◯ Positive
◯ Negative
◯ Indeterminate

Please specify the result for the schistosomiasis

☐ Trace
☐ 1+
☐ 2+
☐ 3+
☐ 4+

____

Note:

Please notify the study clinician

Notes

Notes

For peer review only

Calling Reminders

Staff Name ______________________

Today's Date ______________________

Time ______________________

Opening date for Week 30-34 ______________________

Closing date for Week 30-34 ______________________

NOTE:
You are about to call a participant to remind them of a specific visit. Please make sure to do the following:

1. Obtain all relevant contact numbers for the participant on their record
2. Ensure that you have checked what the exact date is when the participant is expected to present
3. Make sure to give the participant a brief description of what will be done at the visit.
4. You will make up to 3 attempts to get hold of the participant.

Please select "Proceed"

◯ Proceed

The presentation dates are below:

TOC
Date: [baseline_arm_1][toc_3week]

WEEK 28 CALLING

Date: [baseline_arm_1][sched_28w_rem]

WEEK-32

30-34 Week Open Date: [week_28_arm_1][calling_wk30_34_open_date]

30-34 Week Close Date: [week_28_arm_1][calling_wk30_34_close_date]

30-34 Week Actual Date: [week_28_arm_1][week32_visit_date]

POST-NATAL VISIT

Visit open date: [predelivery_checki_arm_1][pd_remind_date_schedpd]

Visit close date: [predelivery_checki_arm_1][pd_close_date_schedpd]

Date of Delivery: [predelivery_checki_arm_1][pd_remind_date_delivery]

For peer review only

For peer review only - http://bmjopen.bmj.com/site/about/guidelines.xhtml

6-WEEK IMMUNIZATION VISIT

Actual Date: [predelivery_checki_arm_1][sixw_im_schedpd]

Please select the calling attempt

☐ First Attempt
☐ Second Attempt
☐ Third Attempt

Details of Calling Attempt 1

Outcome of the attempt

◯ Successful - Participant
◯ Successful - Family member
◯ Unsuccessful - Voicemail
◯ Unsuccessful - Invalid

Date of the attempt

____

Details of Calling Attempt 2

Outcome of the attempt

◯ Successful - Participant
◯ Successful - Family member
◯ Unsuccessful - Voicemail
◯ Unsuccessful - Invalid

Date of the attempt

____

Details of Calling Attempt 3

Outcome of the attempt

◯ Successful - Participant
◯ Successful - Family member
◯ Unsuccessful - Voicemail
◯ Unsuccessful - Invalid

Date of the attempt

____

For peer review only

Notes

Calling notes

For peer review only

ToC Visit Activities

Staff Name ______________________

Today's date ______________________

Start time ______________________

NOTE
Did the participant present within the specified dates presented below:

Start: [baseline_arm_1][toc_reminder_date]

Actual: [baseline_arm_1][toc_3week]

End: [baseline_arm_1][arm1_toc_close_date]

◯ Yes
◯ No

The participant did not have a positive baseline STI result and therefore a ToC visit is not applicable. Activities associated with this visit will need to be captured under the "Ad-Hoc" tool

Note
You are about to start activities associated with the Test-of-Cure visit for participants in arm 1. The following activities are associated with this visit:

1. Collect 1 Loop with 2 slides
2. Collect 3 vaginal Swabs
- Test of Cure Test
- Profiling (Storage)
- Microbiome (Storage)
3. Running the Test-of-Cure
4. Collect Clinical History, Adherence and Disclosure data

Please select "Proceed"

◯ Proceed

Specimen Collection_ToC

NOTE
Please collect one vaginal loop and prepare 2 slides. Please remember to do the following:

1. Pack slides individually in their own package
2. Record the PIN on the outside of package
3. Complete the lab CRF with the matching PINs and test instructions

Select "Proceed" to confirm the PINs associated with the slides.

◯ Proceed

For peer review only - http://bmjopen.bmj.com/site/about/guidelines.xhtml

Did you manage to collect the vaginal loop?

◯ Yes
◯ No

Date of vaginal loop specimen collection

____

Please confirm the pin for the first vaginal swab that will be used for Nugent score

◯ [baseline_arm_1][participant_pin]-TL1

Please confirm the pin for the second vaginal swab that will be used for yeast microscopy

◯ [baseline_arm_1][participant_pin]-TL2

NOTE:
You are about the start with the process of collecting the following 3 vaginal swabs:

1. Swab to be used to conduct ToC (Immediately)
2. Swab for profiling
3. Swab to be used for microbiome

Please select "Proceed"

◯ Proceed

Please specify the vaginal pH

____

Please confirm the PIN associated with the vaginal swab that will be used for STI testing.

◯ [baseline_arm_1][participant_pin] - TCV1

Did you manage to collect the vaginal swab for profiling

◯ Yes
◯ No

Please confirm the PIN associated with the vaginal swab that will be used for profiling.

This must be stored

◯ [baseline_arm_1][participant_pin] - TCV2

Did you manage to collect the vaginal swab for microbiome testing?

◯ Yes
◯ No

Please confirm the PIN associated with the vaginal swab that will be used for microbiome.

This must be stored

◯ [baseline_arm_1][participant_pin] - TCV3

For peer review only

Date of specimen collection for vaginal swabs

____

NOTE
You have collected all specimens associated with this visit. Once you select the "Proceed" option below you will be directed to the start of the clinical history questionnaire. The completion of the questionnaire might take some time so it would be a good idea to start running the vaginal swab to conduct the Test of Cure in line with the below baseline results.

NG: [baseline_arm_1][sti_result_ng]

TV: [baseline_arm_1][sti_result_tv]

CT: [baseline_arm_1][sti_result_ct]

◯ Proceed

Clinical History Review

You are done trying to collect specimens. Because your were not able to collect a Vaginal Swab for STI testing you will not be able to run a test. Please proceed to completing the clinical history.

◯ Proceed

Do you currently have any of the following symptoms?
Multiple selection

☐ Abnormal vaginal discharge
☐ Pain during urination
☐ Lower abdominal pain
☐ None

When did these symptoms start for abnormal vaginal discharge?

◯ After previous visit
◯ Persistent since previous visit
◯ Recurrent since previous visit

When did these symptoms start for pain during urination?

◯ After previous visit
◯ Persistent since previous visit
◯ Recurrent since previous visit

For peer review only - http://bmjopen.bmj.com/site/about/guidelines.xhtml

Adherence

NOTE
The following questions pertain to adherence to the STI medication.

Select Proceed

◯ Proceed

Did you finish the whole course of treatment?

◯ Yes
◯ No

How many days did you take treatment for?

____

Did you throw up within 2 hours after taking any of the STI treatment?

◯ Yes
◯ No

Did you take any other non-chronic treatment at the time?

◯ Yes
◯ No

What type of treatment were you taking ?

NOTE
You are done with questions related to Adherence. You are about to start asking questions associated with Disclosure.

Please select "Proceed"

◯ Proceed

Disclosure

Did you have sex in the past month?

◯ Yes
◯ No

For peer review only - http://bmjopen.bmj.com/site/about/guidelines.xhtml

How many different male partners did you have sexual intercourse with in the past month ?

◯ 1
◯ 2
◯ More than 2 partners

Please specify how many partners?

____

What type of sex did you have with partner 1 (Husband/ Steady partner)?

☐ Vaginal
☐ Anal
☐ Oral

Did you use a condom the last time you had sex with this partner?

◯ Yes
◯ No

Did you notify him of your STI result?

◯ Yes I gave him the notification slip
◯ Yes I told him
◯ No

What was his reaction when you told him of your STI infection?

◯ Supportive
◯ Angry
◯ Violent
◯ Disengaged

How did disclosure affect your relationship?

◯ Continued as before
◯ Started using a condom
◯ He engaged with other partners
◯ He refused sex
◯ Relationship ended

Did he take the treatment?

◯ Yes
◯ No
◯ Don't know

Where did he seek treatment?

◯ Private
◯ Public
◯ Traditional

For peer review only

Why did you not notify this partner?

◯ I didn't feel it was necessary
◯ I am embarrassed
◯ I'm afraid he gets angry
◯ I'm afraid he gets violent
◯ I'm afraid he will end the relationship

What type of sex did you have with partner 2?

☐ Vaginal
☐ Anal
☐ Oral

Did you use a condom the last time you had sex with this partner?

◯ Yes
◯ No

Did you notify him of your STI result?

◯ Yes I gave him the notification slip
◯ Yes I told him
◯ No

What was his reaction when you told him of your STI infection?

◯ Supportive
◯ Angry
◯ Violant
◯ Disengaged

How did disclosure affect your relationship?

◯ Continued as before
◯ Started using a condom
◯ He engaged with other partners
◯ He refused sex
◯ Relationship ended

Did he take the treatment?

◯ Yes
◯ No
◯ Don't know

Where did he seek treatment?

◯ Private
◯ Public
◯ Traditional

For peer review only

Why did you not notify your partner?

◯ I didn't feel it was necessary
◯ I am embarrassed
◯ I'm afraid he gets angry
◯ I'm afraid he gets violent
◯ I'm afraid he will end the relationship

Did you tell anyone else of your STI infection?

◯ Yes
◯ No

Who did you tell?
(Select multiple)

☐ Family member
☐ Friend
☐ Healthcare worker
☐ Other

NOTE:
You have completed the ToC questionnaire. Please select "Proceed" to capture the outcome of the STI test.

◯ Proceed

ToC Outcome

	Positive	Negative	Did not test
CT	◯	◯	◯
NG	◯	◯	◯
TV	◯	◯	◯

NOTE: See calculation

The result from the STI result ________________ (1 = Positive, 0 = Negative)

Does the participant show any symptoms of an STI?

◯ Yes
◯ No

Please contact the study clinician and discuss treatment.

For peer review only

The participant tested/screened positive for an STI. Please specify the treatment that has been provided.

☐ Azithromycin 1g stat dose
☐ Azithromycin 2g stat dose
☐ Ceftriaxone 250mg IM injection
☐ Ceftriaxone 1g IM injection
☐ Metronidazole 400mg bd x 1 week
☐ Metronidazole 2g stat dose
☐ Clotrimazole pessary and/or cream
☐ Ceftriaxone 500mg IM injection

Date treatment given

____

Please specify if a partner notification has been given to the patient.

◯ Yes
◯ No

NOTE
The patient did not test positive or show any signs of an infection

Select "Proceed" to conclude visit

◯ Proceed

Notes

Additional notes

You have completed the ToC Visit Activities. Please make sure to check if all relevant fields have been selected and the information captured is accurate.

Once this is done, please select the "Complete" option below and then select "Save & Exit".

For peer review only

ToC specimens_result

You are about to capture the results of the first loop used for Nugent scoring.

Please select "Proceed" to continue

◯ Proceed

Was the reading satisfactory for the Nugent score?

◯ Yes
◯ No

____

Please specify the Nugent score

____

Please specify if candida was present

◯ Yes
◯ No

Additional comments

You are about to capture the results of the second loop used for smear microscopy.

Please select "Proceed" to continue

◯ Proceed

Was the reading satisfactory?

◯ Yes
◯ No

____

Please specify the Nugent score

____

For peer review only

Please specify if candida was present

◯ Yes
◯ No

Additional comments

For peer review only

Scheduling_Office

Scheduling the Dates Associated with the 32 Week Gestational Visit

The pregnancy (in days) is currently: ______________________

NOTE
You are about the start scheduling dates associated with the 32-week visit. You will need to schedule the following associated dates:

1. Week 32 date - Actual visit

2. Week 32 reminder date

3. Week 30 date - Visit window opens

4. Week 35 date - Visit window closes

Select "Proceed" to start scheduling

◯ Proceed

Schedule the date for the 32 week gestational age, visit

Note to RA: please make sure that this date does not fall on Friday, weekend, and public holidays.

____

Calculate assist for 32-week visit

The number here must between 210 and 244.

____

Days Difference (the difference between 32 weeks & Gestational age)

____

Match ______________________

The date you have entered does not meet the 93 day criteria. Does the intended or original date fall on a Friday weekend or public holiday?

◯ Yes
◯ No

ERROR
The numbers you have entered does not match. Please select a different date so that the numbers match.

Dates Associated with reminder for the 32 Week Gestational Age Visit

For peer review only - http://bmjopen.bmj.com/site/about/guidelines.xhtml

Dates associated with the 32 weeks gestational age missed visit

NOTE:
You have successfully scheduled the 32-week date.

We will need to contact the participant at least 3 days before the scheduled visit to remind them.

Select "Proceed" to schedule the reminder date for the 32 week visit.

◯ Proceed

Note:
Schedule the date for the 32 week reminder. We will contact each participant starting 3 days prior to their 32-week gestation date. That means the date scheduled here should be 3 days earlier then the scheduled date for the 32-week visit. If the date falls on a weekend choose the closest week date.

____

Calculation assist for scheduling the 32-week reminder date.

This number must be between 1 and 4.

____

ERROR
The date that you have entered is invalid. Please select a different date so that the number is less than or equal to 3.

NOTE:
You have successfully scheduled the 32-week reminder date.

Select "Proceed" to schedule the 32 week open visit date.

◯ Proceed

Schedule the date for the 32 weeks opening visit date.

Note: Participants will have from 30 weeks of gestation to present for their 32-week visit date.

____

Calculation Assist for scheduling the 32-Week opening visit date.

This number must equal to 210

____

ERROR
The number you have entered does not match 210. Please select a different date so that the number equals 210.

NOTE:
You have successfully scheduled the 32-week opening date.

Select "Proceed" to schedule the 32 weeks missed visit date.

◯ Proceed

For peer review only

Schedule the date for the 32 weeks missed visit date.

Note: Participants will have until 34 weeks of gestation to present for their 32-week visit date after which the visit will be closed out.

____

Calculation Assist for scheduling the 32-Week missed visit date.

This number must equal to 244

____

ERROR
The number you have entered does not match 244. Please select a different date so that the number equals to 244.

Estimated Delivery Date

You are about the schedule the Estimated Delivery Date.

Please select "proceed"

◯ Proceed

Estimated Delivery Date

____

Days difference between estimated date of delivery and gestational age

____

Calculation Assist for scheduling the Estimated Date for Delivery date.

This number must equal to [edod_calc]

____

Match ________________________________

ERROR
The number you have entered does not match. Please select a different date so that the numbers match

You have completed all the scheduling dates.

Please check that all dates entered comply with the "calculation assistance".

For peer review only

You are about the schedule dates associated with the following events:

1. Pre-birth check-inn

◯ Proceed

Check-In Calling at 37 Weeks

Proceed to the check-in calling date

◯ Proceed

Check-in calling date

____

Calculation Assist for check-in calling date

This number must equal to 259

____

NOTE
The date you have entered is incorrect. Please make sure that the numbers correspond.

CONGRATULATIONS

You have finished scheduling all dates.

For peer review only

32-Week Visit Activities

Staff name

____

Today's date

____

Start time

____

Did the participant present within the dates presented below:

30-34 Week Start Date: [week_28_arm_1][sched_32w_open_date]

30-34 Week Actual Date: [week_28_arm_1][week32_visit_date]

30-34 Week Closing Date: [week_28_arm_1][sched_32w_mv_date]

◯ Yes
◯ No

Open ad-hoc visit to capture relevant information

Specimen Collection_32-Week

NOTE
You will now start with the process of specimen collection. You will need to collect several specimens from the participant. These specimens need to be collected in the order in which they are presented here.

The following specimens will need to be collected:
1. 1 x Vaginal loop to be used to prepare two slides
2. 1 x Vaginal swab to be used for STI testing (Arm 2 and Microbiome (Empilweni): immediate testing; Arm 1 and 3: Storage)
3. 1 x Vaginal swab to be used for profiling
4. 2 x Vaginal swab to be used for microbiome
5. 1 x Vaginal swab to be used for cytokine

◯ Proceed

Vaginal Smear

Please specify the vaginal pH

____
(if not available, enter 99)

For peer review only

Please select which pH strips are used to measure vaginal pH

◯ CardinalHealth pH Indicator Strips (range 3.6-6.1)
◯ pH Indicator Strips pH 0-14
◯ Natureland vaginal pH test (range 3.5-6.5)

You will need to use a single loop to collect vaginal smear on two glass slides for microscopy (if not available, enter 99)

◯ Done

Confirm PIN associated with the first Vaginal Loop to be used to test for Nugent score

◯ [baseline_arm_1][participant_pin] - WL1

Confirm PIN associated with the second Vaginal Loop to be used to test for Yeast microscopy

◯ [baseline_arm_1][participant_pin] - WL2

Vaginal Swabs

NOTE
You will now collect four vaginal swabs. They will be used as follows:

1. STI testing (Arm 2 and Microbiome (Empilweni): immediate testing; Arm 1 and 3: Storage)
2. Profiling (stored)
3. Microbiome (stored)
4, Cytokine (stored)

◯ Done

Confirm PIN associated with the vaginal swab to be used to test for STI

◯ [baseline_arm_1][participant_pin] - WV1

Please confirm PIN associated with the vaginal swab to be used for Profiling

◯ [baseline_arm_1][participant_pin] - WV2

Please confirm PIN associated with the vaginal swab to be used for microbiome

◯ [baseline_arm_1][participant_pin] - WV3

Please confirm the PIN associated with the vaginal swab that will be used for cytokines.

◯ [baseline_arm_1][participant_pin] - WV4

NOTE
The participant is in arm 2 and therefore an immediate STI test is conducted at the 32-week visit. Please prepare the swab for testing before you continue to the questionnaires.

◯ Proceed

NOTE
You are done with all specimen collection and will now proceed to administering the clinical history.

Please select "Proceed"

◯ Proceed

For peer review only - http://bmjopen.bmj.com/site/about/guidelines.xhtml

Clinical History Review

Have you been to the clinic since the last visit with us?

◯ Yes
◯ No

What was the purpose of your visit?

☐ ANC Visit
☐ HIV/ART
☐ STI Treatment
☐ Other

Summary notes from visit

Have you used any of the following since the first study visit?
Select multiple

☐ Alcohol
☐ Tik
☐ Dagga
☐ Grandpa
☐ Other
☐ None

Please specify other drugs used?

Do you currently have any of the following symptoms?

RA: Please select all that apply

☐ Abnormal vaginal discharge
☐ Pain during urination
☐ Lower abdominal pain
☐ Pain related to intercourse
☐ Vaginal bleeding related to intercourse
☐ Genital itchiness
☐ Any skin abnormalities
☐ None

When did these symptoms start for abnormal vaginal discharge?

◯ After previous visit
◯ Persistent since previous visit
◯ Recurrent since previous visit

For peer review only

When did these symptoms start for pain during urination?

◯ After previous visit
◯ Persistent since previous visit
◯ Recurrent since previous visit

When did these symptoms start for the lower abdominal pain?

◯ After previous visit
◯ Persistent since previous visit
◯ Recurrent since previous visit

When did these symptoms start for the pain related to intercourse?

◯ After previous visit
◯ Persistent since previous visit
◯ Recurrent since previous visit

When did these symptoms start for vaginal bleeding related to intercourse?

◯ After previous visit
◯ Persistent since previous visit
◯ Recurrent since previous visit

When did these symptoms start for genital itchiness?

◯ After previous visit
◯ Persistent since previous visit
◯ Recurrent since previous visit

Please specify any skin abnormalities

Baseline Treatment Date:
[baseline_arm_1][sti_treatment_date]

TOC Treatment Date:
[toc_arm_1_arm_1][toc_sti_treatment_date]

Did the participant receive any STI treatment at their last study visit?

◯ Yes
◯ No

Are you planning to wait for your results today?
(New question added @13/09/2022)

◯ Yes
◯ No

For peer review only

What is your main reason why you are not intending to wait today?
(New question added @13/09/2022)

- ◯ Have to get to work
- ◯ Have to get back to my kids/family
- ◯ Want to go to the shop
- ◯ Transport availability
- ◯ Lack of privacy
- ◯ Hungry
- ◯ No space to wait
- ◯ Not feeling well
- ◯ Boring
- ◯ Other

If "Other", please specify.
(New question added @13/09/2022)

What would make you change your mind?
(New question added @13/09/2022)

You are done with questions associated with clinical history review. You will now start with questions associated with Adherence.

- ◯ Proceed

Adherence

Did you finish the whole course of STI treatment

- ◯ Yes
- ◯ No

How many days did you take treatment for?

____

Did you throw up within 2 hours after taking any of the STI treatment

- ◯ Yes
- ◯ No

Did you take any other non-chronic treatment at the time

- ◯ Yes
- ◯ No

What type of treatment

For peer review only

You are done with questions associated with the adherence. You are about to start asking questions associated with disclosure.

◯ Proceed

Disclosure

Did you notify your partner of your STI result?

◯ Yes I gave him the notification slip
◯ Yes I told him
◯ No

What was his reaction when you told him of your STI infection

◯ Supportive
◯ Angry
◯ Violent
◯ Disengaged

How did disclosure affect your relationship?

◯ Continued as before
◯ Started using a condom
◯ He engaged with other partners
◯ He refused sex
◯ Relationship ended

Did he take treatment?

◯ Yes
◯ No
◯ I don't know

Where did he seek treatment

◯ Private
◯ Public
◯ Traditional

Why did you not notify your partner?

◯ I didn't feel it was necessary
◯ I am embarrassed
◯ I'm afraid he gets angry
◯ I'm afraid he gets violent
◯ I'm afraid he will end the relationship

Did you tell anyone else of your STI infection?

◯ Yes
◯ No

For peer review only

Who did you tell?

☐ Family member
☐ Friend
☐ HCW
☐ Other

Behavioral Questionnaire

NOTE TO RA:
You just completed all questions related to Disclosure. You are about to start with the Behavioral Questionnaire.

Please select "Proceed":

◯ Proceed

Did you have sex since the last visit?

◯ Yes
◯ No

How many different male partners did you have sexual intercourse with in the past month?

◯ 1
◯ 2
◯ more than 2

Were any of these new partners than the ones from the last visit

◯ Yes
◯ No

What type of sex did you have with partner 1 (Husband/ Steady partner)?

☐ Vaginal
☐ Anal
☐ Oral

Did you use a condom the last time you had sex with partner 1 (Husband/ Steady partner)?

◯ Yes
◯ No

What type of sex did you have with partner 2?

☐ Vaginal
☐ Anal
☐ Oral

Did you use a condom the last time you had sex with partner 2?

◯ Yes
◯ No

For peer review only

What type of sex did you have with the rest of the partners?

☐ Vaginal
☐ Anal
☐ Oral

Did you use a condom the last time you had sex with one of them?

◯ Yes
◯ No

Where are you planning to deliver?

◯ Frere
◯ CMH
◯ Nontyantyambo
◯ Empilweni
◯ Bisho
◯ Other

Please specify

You are done with the questions associated with Behavioral Questionnaire. You will now start asking questions associated with the Physical Examination.

◯ Proceed

Physical Examination

Weight of mother

____

Systolic blood pressure

____

Diastolic blood pressure

____

How was Hemoglobin measured?

◯ Hb meter at the clinic
◯ Hb at NHLS

Please capture Hb result"

____

For peer review only

Please capture the barcode for Hb

Fundal height

Progression of pregnancy

◯ Progressing normal
◯ Abnormality detected

Provide further details of abnormality

During your examination, were there any signs of abnormal vaginal discharge?

◯ Yes
◯ No

During your examination, were there any signs of inguinal lymphadenopathy?

◯ Yes
◯ No

Are these bubo?

◯ Yes
◯ No

Note to RA: Please contact the study clinician and specify treatment given to the participant

During your examination, were there any signs of lower abdominal pain?

◯ Yes
◯ No

During your examination, where there any signs of scratch marks?

◯ Yes
◯ No

During your examination, were there any signs of skin conditions?

◯ Yes
◯ No

Please specify the nature of the skin conditions

For peer review only

For peer review only - http://bmjopen.bmj.com/site/about/guidelines.xhtml

During your examination, were there any other observations that need to be noted?

You have completed the questions associated with the Physical Examination. You will now start capturing the results from the rapid tests.

◯ Proceed

Rapid Test Results

Do you know your current HIV status?

◯ HIV negative (tested today by clinical staff)
◯ HIV positive on ART
◯ HIV positive, not on ART
◯ Don't know (never tested)
◯ Don't know (no yet tested today)

Was the participant newly diagnosed with HIV today

◯ Yes
◯ No

Please conduct an HIV Rapid test and capture the result below

◯ Positive
◯ Negative

Please conduct a confirmatory HIV Rapid test and capture the result below

◯ Positive
◯ Negative

Did you collect a tube of blood for CD4 count?

◯ Yes
◯ No

Please record barcode for blood tube for CD4 count testing?

____

Is the participant's most recent CD4 count since Baseline available?

◯ Yes
◯ No

What was the date of the CD4 specimen collection?

____

For peer review only - http://bmjopen.bmj.com/site/about/guidelines.xhtml

What was the participant's most recent CD4 count?

____
(if no number listed, enter 9999)

Did you collect a tube of blood for viral load?

◯ Yes
◯ No

Please record barcode for blood tube for viral load testing?

____

Is the participant's most recent viral load since Baseline available?

◯ Yes
◯ No

What was the date of the viral load specimen collection?

____

What was the participant's most recent viral load?

____
(if no number listed, enter 0000)

Which regimen for ART were you started on today?

◯ TLD
◯ TEE
◯ AZT/3TC/LPV
◯ Other

Which regimen for ART were you on so far?

◯ TLD
◯ TEE
◯ AZT/3TC/LPV
◯ Other

Has the regimen for ART been changed today?

◯ Yes
◯ No

To which regimen for ART has it been changed today?

◯ TLD
◯ TEE
◯ AZT/3TC/LPV
◯ Other

For peer review only

Has a syphilis test been done for the participant?

◯ Yes
◯ No

Which syphilis test have you used?

◯ Alere Syphilis TP (provided by FPD)
◯ HIV/Syphilis Duo (provided by FPD)
◯ No rapid test used only NHLS bloods for RPR
◯ Other please specify

Please specify

Syphilis result.

◯ Positive
◯ Negative
◯ Indeterminate

Titer value 1:

____
(If RPR is non-reactive, enter 0)

Blood needs to be collected for further syphilis testing. Please confirm if blood was collected.

◯ Yes
◯ No

Collect blood for RPR and capture barcode PIN below

____

Treatment given

◯ Benzathine penicillin 2.4 MU IM weekly x3
◯ Out of stock

Please contact study clinician and specify treatment given

Please collect participant's urine for testing

For peer review only

Urine Dipstick test results

You are about to capture the results from the dipstick testing

Please select "Proceed" to continue

◯ Proceed

Blood - Hemoglobin

◯ Negative
◯ Ca. 10
◯ Ca. 50
◯ Ca. 250/300

Blood - Erythrocytes

◯ Negative
◯ Ca. 5 -10
◯ Ca. 50
◯ Ca. 250/300

Urobilinogen

◯ Normal
◯ 2
◯ 4
◯ 8
◯ 12

Bilirubin

◯ Negative
◯ 1 plus
◯ 2 plus
◯ 3 plus
◯ Not available

Protein

◯ Negative
◯ 30
◯ 100
◯ 500

Nitrate

◯ Negative
◯ Positive

For peer review only

Keton

◯ Negative
◯ 1 plus
◯ 2 plus
◯ 3 plus
◯ Not available

Glucose

◯ Negative
◯ Normal
◯ 50
◯ 150
◯ 500
◯ ≥1000

pH

◯ 5
◯ 6
◯ 7
◯ 8
◯ 9
◯ Not available

SG

◯ 1.000
◯ 1.005
◯ 1.010
◯ 1.015
◯ 1.020
◯ 1.025
◯ 1.030
◯ Not available

Leucocytes

◯ Negative
◯ 25
◯ 75
◯ 500

STI Results and Screening

For peer review only

The participant is in arm 2 or for Empilweni and therefore you are about to capture results for the STI testing.

◯ Proceed

STI Test Outcome

The previous STI results of the participants are:

Baseline
NG:[baseline_arm_1][sti_result_ng]

CT:[baseline_arm_1][sti_result_ct]

TV:[baseline_arm_1][sti_result_tv]

TOC
NG:[toc_arm_1_arm_1][toc_ng]

CT:[toc_arm_1_arm_1][toc_ct]

TV:[toc_arm_1_arm_1][toc_tv]

	Negative	Positive
CT	◯	◯
NG	◯	◯
TV	◯	◯

STI Calculation _ w32

____

Date the result was obtained.

____

Did the participant wait for her STI results?
(New question added @ 03/11/2022)

◯ Yes
◯ No

Was the participant reporting STI symptoms or showed symptoms during the clinical assessment?

◯ Yes
◯ No

Does the participant report any medication allergies?

◯ Yes
◯ No

For peer review only

Please contact the study clinician before giving any treatment. Please specify discussed medication allergies and treatment plan with the study clinician

The following treatment has been provided

- ☐ Azithromycin 1g stat dose
- ☐ Azithromycin 2g stat dose
- ☐ Ceftriaxone 250mg IM injection
- ☐ Ceftriaxone 1g IM injection
- ☐ Metronidazole 400mg bd x 1 week
- ☐ Metronidazole 2g stat dose
- ☐ Clotrimazole pessary and/or cream
- ☐ Trimethoprim/sulfamethoxazole 400/80 mg 2 tbl. bds for 5 days (bactrim)
- ☐ Ceftriaxone 500mg IM injection

Date treatment given

____

What made you change your mind about waiting for the results? (New question added @13/09/2022)

Partner notification provided

- ◯ Yes, 1
- ◯ Yes, multiple
- ◯ No

Please explain why the partner notification note was not provided?

You have completed capturing the information from the 32 week exam. Please make sure to check that you have completed all the fields.

Please select "Complete" then "Save and Exit".

Notes

Additional notes

For peer review only

32W specimens results

You are about to capture the results of specimens collected during the 32-week visit

Please select "Proceed" to continue

◯ Proceed

Hb Results received

◯ Yes
◯ No

Please capture the Hb result

____

Please capture the barcode for Hb

____

Please specify whether the reading was satisfactory for the loop used for Nugent score

◯ Yes
◯ No

____

Please capture the score for the loop used for Nugent scoring

____

Please specify if candida was present for the loop used for Nugent scoring

◯ Yes
◯ No

Please specify whether the reading was satisfactory for the loop used for yeast microscopy

◯ Yes
◯ No

____

Please capture the nugent score for the loop used for yeast microscopy

____

For peer review only

Please specify if candida was present for the loop used for yeast microscopy

◯ Yes
◯ No

Please capture the results for the blood used for Syphilis testing

◯ RPR Negative
◯ RPR Positive
◯ RPR Indeterminate

____

Please capture the result for viral load testing

____

Is the participant's most recent viral load available?

◯ Yes
◯ No

What was the date of the viral load specimen collection?

____

What was the participant's most recent viral load?

____

____

Notes

Notes

For peer review only

Calling Reminders_2

Staff Name ______________________

Today's Date ______________________

Time ______________________

The presentation dates are below:

TOC
Date: [baseline_arm_1][toc_3week]

WEEK 28 CALLING

Date: [baseline_arm_1][sched_28w_rem]

WEEK-32

30-34 Week Actual Date: [week_28_arm_1][week32_visit_date]

Call In Check

Week 37 Call In Check: [week_28_arm_1][cic_date]

POST-NATAL VISIT

Visit open date: [predelivery_checki_arm_1][pd_remind_date_schedpd]

Visit close date: [predelivery_checki_arm_1][pd_close_date_schedpd]

Date of Delivery: [predelivery_checki_arm_1][pd_remind_date_delivery]

6-WEEK IMMUNIZATION VISIT

Actual Date: [predelivery_checki_arm_1][sixw_im_schedpd]

NOTE:
You are about to call a participant to remind them of a specific visit. Please make sure to do the following:

1. Obtain all relevant contact numbers for the participant on their record
2. Ensure that you have checked what the exact date is when the participant is expected to present
3. Make sure to give the participant a brief description of what will be done at the visit.
4. You will make up to 3 attempts to get hold of the participant.

Please select "Proceed"

◯ Proceed

For peer review only - http://bmjopen.bmj.com/site/about/guidelines.xhtml

For peer review only

Please select the calling attempt

- ☐ First Attempt
- ☐ Second Attempt
- ☐ Third Attempt

Details of Calling Attempt 1

Outcome of the attempt

- ◯ Successful - Participant
- ◯ Successful - Family member
- ◯ Unsuccessful - Voicemail
- ◯ Unsuccessful - Invalid

Date of the attempt

____

Did she deliver?

- ◯ Yes
- ◯ No

Capture delivery Date

NOTE: instruct to come to the site.

____

Details of Calling Attempt 2

Outcome of the attempt

- ◯ Successful - Participant
- ◯ Successful - Family member
- ◯ Unsuccessful - Voicemail
- ◯ Unsuccessful - Invalid

Date of the attempt

____

For peer review only - http://bmjopen.bmj.com/site/about/guidelines.xhtml

Did she deliver?

◯ Yes
◯ No

Capture delivery Date

NOTE: instruct to come to the site.

____

Details of Calling Attempt 3

Outcome of the attempt

◯ Successful - Participant
◯ Successful - Family member
◯ Unsuccessful - Voicemail
◯ Unsuccessful - Invalid

Date of the attempt

____

Did she deliver?

◯ Yes
◯ No

Capture delivery Date

NOTE: instruct to come to the site.

____

Notes

Calling notes

For peer review only

Schedule calling for 38 weeks

NOTE FW: Please make sure that you call the participant once per week.

◯ Proceed

Did you schedule the 38 weeks call

◯ Yes
◯ No

Please select the calling attempt

☐ First Attempt
☐ Second Attempt
☐ Third Attempt

Details of Calling Attempt 1

Outcome of the attempt

◯ Successful - Participant
◯ Successful - Family member
◯ Unsuccessful - Voicemail
◯ Unsuccessful - Invalid

Date of the attempt

____

Did she deliver?

◯ Yes
◯ No

Details of Calling Attempt 2

Outcome of the attempt

◯ Successful - Participant
◯ Successful - Family member
◯ Unsuccessful - Voicemail
◯ Unsuccessful - Invalid

Date of the attempt

____

For peer review only

Did she deliver?

◯ Yes
◯ No

Details of Calling Attempt 3

Outcome of the attempt

◯ Successful - Participant
◯ Successful - Family member
◯ Unsuccessful - Voicemail
◯ Unsuccessful - Invalid

Date of the attempt

____

Did she deliver?

◯ Yes
◯ No

Schedule calling for 39 weeks

NOTE FW: Please make sure that you call the participant once per week.

◯ Proceed

Did you schedule the 39 weeks call

◯ Yes
◯ No

Please select the calling attempt

☐ First Attempt
☐ Second Attempt
☐ Third Attempt

For peer review only

Details of Calling Attempt 1

Outcome of the attempt

◯ Successful - Participant
◯ Successful - Family member
◯ Unsuccessful - Voicemail
◯ Unsuccessful - Invalid

Date of the attempt

____

Did she deliver?

◯ Yes
◯ No

Capture delivery Date

NOTE: instruct to come to the site.

____

Details of Calling Attempt 2

Outcome of the attempt

◯ Successful - Participant
◯ Successful - Family member
◯ Unsuccessful - Voicemail
◯ Unsuccessful - Invalid

Date of the attempt

____

Did she deliver?

◯ Yes
◯ No

Details of Calling Attempt 3

Outcome of the attempt

◯ Successful - Participant
◯ Successful - Family member
◯ Unsuccessful - Voicemail
◯ Unsuccessful - Invalid

Date of the attempt

____

For peer review only

For peer review only - http://bmjopen.bmj.com/site/about/guidelines.xhtml

Did she deliver?

◯ Yes
◯ No

Calling notes

Schedule calling for 40 weeks

NOTE FW: Please make sure that you call the participant once per week.

◯ Proceed

Please select the calling attempt

☐ First Attempt
☐ Second Attempt
☐ Third Attempt

Did you schedule a call for 40 weeks

◯ Yes
◯ No

Details of Calling Attempt 1

Outcome of the attempt

◯ Successful - Participant
◯ Successful - Family member
◯ Unsuccessful - Voicemail
◯ Unsuccessful - Invalid

Date of the attempt

____

Did she deliver?

◯ Yes
◯ No

Capture delivery Date

NOTE: instruct to come to the site.

____

For peer review only

Details of Calling Attempt 2

Outcome of the attempt

◯ Successful - Participant
◯ Successful - Family member
◯ Unsuccessful - Voicemail
◯ Unsuccessful - Invalid

Date of the attempt

____

Did she deliver?

◯ Yes
◯ No

Details of Calling Attempt 3

Outcome of the attempt

◯ Successful - Participant
◯ Successful - Family member
◯ Unsuccessful - Voicemail
◯ Unsuccessful - Invalid

Date of the attempt

____

Calling notes

Did she deliver?

◯ Yes
◯ No

Schedule calling for 41 weeks

NOTE FW: Please make sure that you call the participant once per week.

◯ Proceed

Please select the calling attempt

☐ First Attempt
☐ Second Attempt
☐ Third Attempt

For peer review only

Did you schedule a call for 41 weeks

◯ Yes
◯ No

Details of Calling Attempt 1

Outcome of the attempt

◯ Successful - Participant
◯ Successful - Family member
◯ Unsuccessful - Voicemail
◯ Unsuccessful - Invalid

Date of the attempt

____

Details of Calling Attempt 2

Outcome of the attempt

◯ Successful - Participant
◯ Successful - Family member
◯ Unsuccessful - Voicemail
◯ Unsuccessful - Invalid

Date of the attempt

____

Did she deliver?

◯ Yes
◯ No

Details of Calling Attempt 3

Outcome of the attempt

◯ Successful - Participant
◯ Successful - Family member
◯ Unsuccessful - Voicemail
◯ Unsuccessful - Invalid

Date of the attempt

____

Calling notes

For peer review only

Did she deliver?

◯ Yes
◯ No

Capture delivery Date

NOTE: instruct to come to the site.

____

Schedule calling for 41 weeks (293 days)

NOTE FW: Please make sure that you call the participant once per week.

◯ Proceed

Did you schedule a call for 41 weeks

◯ Yes
◯ No

Please select the calling attempt

☐ First Attempt
☐ Second Attempt
☐ Third Attempt

Details of Calling Attempt 1

Outcome of the attempt

◯ Successful - Participant
◯ Successful - Family member
◯ Unsuccessful - Voicemail
◯ Unsuccessful - Invalid

Calling notes

Did she deliver?

◯ Yes
◯ No

Capture delivery Date

NOTE: instruct to come to the site.

____

For peer review only

Schedule calling for 42 weeks (296 days)

NOTE FW: Please make sure that you call the participant once per week.

◯ Proceed

Please select the calling attempt

☐ First Attempt
☐ Second Attempt
☐ Third Attempt

Did you schedule a call for 42 weeks

◯ Yes
◯ No

Details of Calling Attempt 1

Outcome of the attempt

◯ Successful - Participant
◯ Successful - Family member
◯ Unsuccessful - Voicemail
◯ Unsuccessful - Invalid

Date of the attempt

____

Did she deliver?

◯ Yes
◯ No

Details of Calling Attempt 2

Outcome of the attempt

◯ Successful - Participant
◯ Successful - Family member
◯ Unsuccessful - Voicemail
◯ Unsuccessful - Invalid

Date of the attempt

____

For peer review

Did she deliver?

◯ Yes
◯ No

Details of Calling Attempt 3

Outcome of the attempt

◯ Successful - Participant
◯ Successful - Family member
◯ Unsuccessful - Voicemail
◯ Unsuccessful - Invalid

Date of the attempt

____

Outcome of the call

Did the participant deliver?

◯ Yes
◯ No

Calling notes

Did she deliver?

◯ Yes
◯ No

Capture delivery Date

NOTE: instruct to come to the site.

____

For peer review only

Scheduling_Post_Delivery_Activities using Updated EDD

You are about the schedule dates associated with the following events:

1. Post-Delivery Appointment
2. 6-Weeks Immunization Appointment

◯ Proceed

Post-Delivery Study Visit

You will now schedule dates associated with the post-delivery study visit.

The following dates are associated with this visit:
1. Calling reminder date / Visit opening date
2. Visit closing date

Select "Proceed" to continue

◯ Proceed

Please capture the date of delivery

____

Please schedule the date for the post-delivery reminder call.

Please note that the Delivery date is [pd_remind_date_delivery]. The reminder call will happen 1 day following delivery.

____

Calculation Assist for Post Delivery reminder call

This number must equal to: 1

____

Match

____

NOTE
The date you have entered is incorrect. Please make sure that the numbers correspond.

For peer review only - http://bmjopen.bmj.com/site/about/guidelines.xhtml

The post-delivery closing date.

____

Calculation Assist for Post Delivery closing date

The participant will have 14 days post-delivery to present at the clinic. The delivery date was [pd_remind_date_delivery]. This number must therefore equal to: 14

____

Match

____

NOTE
The date you have entered is incorrect. Please make sure that the numbers correspond.

Facility delivered

◯ Frere
◯ CMH
◯ Nontyantyambo
◯ Empilweni
◯ Bisho
◯ Other

Please specify the facility of delivery

____

6-Week Immunization Visit

NOTE:
In this section you will schedule all dates associated with the 6-Week Immunization Study Visit. These dates will include:

1. Calling reminder date for 6-Weeks Immunization visit
2. Scheduled date of 6-Weeks Immunization visit
3. Closing date for attending the 6-Weeks Immunization visit

Select Proceed to continue

◯ Proceed

Please schedule the date for the 6-weeks immunization reminder

____

For peer review only - http://bmjopen.bmj.com/site/about/guidelines.xhtml

Calculation Assist for 6-weeks immunization reminder. We will call all patients 5 weeks (35 days) following their delivery date. The updated delivery date for the participant was [calling_delivery_date_37weeks].

This number must therefore equal to: 35 and 40

____

Match

____

NOTE
The date you have entered is incorrect. Please make sure that the numbers correspond.

Please schedule the date for the 6-weeks immunization visit. This visit is scheduled to take place 6 weeks (42 days) following delivery. The updated delivery date is [calling_delivery_date_37weeks]

____

Calculation Assist for 6-weeks immunization visit

This number must equal to: 42

____

Match

____

NOTE
The date you have entered is incorrect. Please make sure that the numbers correspond.

Please schedule the date for the 6-weeks immunization visit closing date.

____

Calculation Assist for 6-Weeks Immunization visit Close Date. Mothers will have up to 8 weeks post delivery to attend this visit. This means 56 days following the delivery.

This number must equal to: 56

____

Match

____

For peer review only

NOTE
The date you have entered is incorrect. Please make sure that the numbers correspond.

CONGRATULATIONS

You have finished scheduling all dates.

For peer review only

Birth Register Data

Participant PIN

[baseline_arm_1][participant_pin]

Staff name

____

Today's date

____

Start time

____

You are about to capture data retrieved from the birth registry. Please select "Proceed" to start

◯ Proceed

Delivery Details

Delivery site

◯ Frere
◯ CMH
◯ Nontyantyambo
◯ Empilweni
◯ Bisho
◯ Other

Please specify name of delivery facility

Clinic file number

____

Delivery date

____

Calculated gestational age

____
(Added @22/03/2023)

For peer review only

Please specify the number of babies during pregnancy

◯ 1
◯ 2
◯ 3

Outcome type for baby 1

◯ Live birth
◯ Still birth
◯ Early Neonatal Death

Outcome type for baby 2

◯ Live birth
◯ Still birth
◯ Early Neonatal Death

Outcome type for baby 3

◯ Live birth
◯ Still birth
◯ Early Neonatal Death

Type of delivery for baby 1

◯ Born before arrival
◯ Normal Vaginal Delivery
◯ Assisted Vaginal Delivery
◯ Elective Cesarean Section
◯ Emergency Cesarean Section

Type of delivery for baby 2

◯ Born before arrival
◯ Normal Vaginal Delivery
◯ Assisted Vaginal Delivery
◯ Elective Cesarean Section
◯ Emergency Cesarean Section

Type of delivery for baby 3

◯ Born before arrival
◯ Normal Vaginal Delivery
◯ Assisted Vaginal Delivery
◯ Elective Cesarean Section
◯ Emergency Cesarean Section

Please specify reason

Please specify reason

For peer review only

Please specify reason

Gender - Baby 1

◯ Female
◯ Male

Gender - Baby 2

◯ Female
◯ Male

Gender - Baby 3

◯ Female
◯ Male

Complications in labor/Delivery

	Yes	No
Induction of labour	◯	◯
Antepartun haemorrhage	◯	◯
Post Partum haemorrhage	◯	◯
Severe pre-eclampsia	◯	◯
Eclampsia	◯	◯
Prolonged rupture of membranes	◯	◯
Ruptured uterus	◯	◯
Sepsis	◯	◯
Obstructed or prolonged labour	◯	◯
Retained Placenta	◯	◯
Manual removal of placenta	◯	◯

Maternal outcome

◯ Live
◯ Death

APGAR score at 5 minutes for baby 1

____

APGAR score at 5 minutes for baby 2

____

APGAR score at 5 minutes for baby 3

____

For peer review only

For peer review only - http://bmjopen.bmj.com/site/about/guidelines.xhtml

Birth weight for baby 1 in grams

____

Birth weight for baby 2 in grams

____

Birth weight for baby 3 in grams

____

Did you breastfeed your baby/ies within 1 hour of giving birth?

◯ Yes
◯ No

Infant feeding
(New question added @15/11/2022)

◯ Exclusive Breast Feeding (EBF)
◯ Exclusive Formula Feeding (EFF)

Any birth defects to note for baby 1

◯ Yes
◯ No

Any birth defects to note for baby 2

◯ Yes
◯ No

Any birth defects to note for baby 3

◯ Yes
◯ No

Please specify

Remarks outcome

Maternal outcome

For peer review only - http://bmjopen.bmj.com/site/about/guidelines.xhtml

You have completed the Birth register. Please make sure to check if all relevant fields have been selected and the information captured is accurate.

Once this is done, please select the "Complete" option below and then select "Save & Exit".

For peer review only

Post-Natal Visit Activities

Staff name ______________________

Today's date ______________________

Start time ______________________

Post-Natal Visit

The participant was scheduled to present within the two dates below. Please specify if the participant presented within this timeframe.

Visit open date: [predelivery_checki_arm_1][pd_remind_date_delivery]

Visit close date: [predelivery_checki_arm_1][pd_close_date_schedpd]

◯ Yes
◯ No

You are about to administer the questions associated with the post natal visit.

Please select "Proceed"

◯ Proceed?

Clinical History Review

Have you been to the clinic since the last visit with us?

◯ Yes
◯ No

What was the purpose of your visit?

☐ ANC Visit
☐ HIV/ART
☐ STI Treatment
☐ Other

Summary notes from the visit

For peer review only

Were there any abnormalities/complications since your last study visit regarding your pregnancy and delivery or did you receive any non-chronic treatment?

◯ Yes
◯ No

Please specify

Have you used any of the following since the first study visit?
Select multiple

☐ Alcohol
☐ Tik
☐ Dagga
☐ Grandpa
☐ Other
☐ None

Please specify other drugs used?

The Baseline STI results of the participants are:

NG: [baseline_arm_1][sti_result_ng]

CT: [baseline_arm_1][sti_result_ct]

TV: [baseline_arm_1][sti_result_tv]

The TOC STI results of the participants are:

NG: [toc_arm_1_arm_1][toc_ng]

CT: [toc_arm_1_arm_1][toc_ct]

TV: [toc_arm_1_arm_1][toc_tv]

The Week 32 STI results of the participants are:

CT: [3034_weeks_arm_1][w32_ct_res]

NG: [3034_weeks_arm_1][w32_ng_res]

TV: [3034_weeks_arm_1][w32_tv_res]

Did the participant receive any STI treatment at their last study visit?

◯ Yes
◯ No

For peer review only

Adherence

You are done with questions associated with the clinical history review. You will now start with questions associated with Adherence.

◯ Proceed

Did you finish the whole course of STI treatment?

◯ Yes
◯ No

How many days did you take treatment for?

____

Did you throw up within 2 hours after taking any of the STI treatment?

◯ Yes
◯ No

Did you take any other non-chronic treatment at the time?

◯ Yes
◯ No

What type of treatment

Disclosure

You are done with questions associated with the adherence. You are about to start asking questions associated with disclosure.

◯ Proceed

Did you notify your partner of your STI result?

◯ Yes I gave him the notification slip
◯ Yes I told him
◯ No

What was his reaction when you told him of your STI infection

◯ Supportive
◯ Angry
◯ Violent
◯ Disengaged

For peer review only

How did disclosure affect your relationship?

◯ Continued as before
◯ Started using a condom
◯ He engaged with other partners
◯ He refused sex
◯ Relationship ended

Did he take treatment?

◯ Yes
◯ No
◯ I don't know

Where did he seek treatment?

◯ Private
◯ Public
◯ Traditional

Why did you not notify your partner?

◯ I didn't feel it was necessary
◯ I am embarrassed
◯ I'm afraid he gets angry
◯ I'm afraid he gets violent
◯ I'm afraid he will end the relationship

Did you tell anyone else of your STI infection?

◯ Yes
◯ No

Did you tell anyone else of your STI infection?

◯ Yes
◯ No

Who did you tell?

☐ Family member
☐ Friend
☐ HCW
☐ Other

Delivery Details of Infant

Facility delivered

◯ Frere
◯ CMH
◯ Nontyantyambo
◯ Empilweni
◯ Bisho
◯ Other

Please specify facility of delivery

For peer review only

Date of delivery

____

Calculated gestational age

____
(Added @22/03/2023)

Please specify the number of babies during pregnancy

◯ 1
◯ 2
◯ 3

Outcome type for baby 1

◯ Live birth
◯ Still birth
◯ Early Neonatal Death

Outcome type for baby 2

◯ Live birth
◯ Still birth
◯ Early Neonatal Death

Outcome type for baby 3

◯ Live birth
◯ Still birth
◯ Early Neonatal Death

Type of delivery for baby 1

◯ Born before arrival
◯ Normal Vaginal Delivery
◯ Assisted Vaginal Delivery
◯ Elective Cesarean Section
◯ Emergency Cesarean Section

Type of delivery for baby 2

◯ Born before arrival
◯ Normal Vaginal Delivery
◯ Assisted Vaginal Delivery
◯ Elective Cesarean Section
◯ Emergency Cesarean Section

Type of delivery for baby 3

◯ Born before arrival
◯ Normal Vaginal Delivery
◯ Assisted Vaginal Delivery
◯ Elective Cesarean Section
◯ Emergency Cesarean Section

Please specify reason

For peer review only

For peer review only - http://bmjopen.bmj.com/site/about/guidelines.xhtml

Please specify reason

Please specify reason

Gender - Baby 1

◯ Female
◯ Male

Gender - Baby 2

◯ Female
◯ Male

Gender - Baby 3

◯ Female
◯ Male

Maternal outcome

◯ Live
◯ Death

Specify

APGAR score at 5 minutes for baby 1

Note to RA: Check on Road to Health

____
(if no number listed, enter 99)

APGAR score at 5 minutes for baby 2
Note to RA: Check on Road to Health

____
(if no number listed, enter 99)

APGAR score at 5 minutes for baby 3
Note to RA: Check on Road to Health

____
(if no number listed, enter 99)

For peer review only

Birth weight in grams for baby 1

Note to RA: Check on Road to Health

____

Birth weight in grams for baby 2

Note to RA: Check on Road to Health

____

Birth Weight in grams for baby 3

Note to RA: Check on Road to Health

____

Newborn problems

Note to RA: Check on Road to Health

☐ Birth defects
☐ Hypoxic brain injury
☐ Convulsions /fits
☐ Jaundice
☐ None

Please Specify

Was the baby exposed to HIV?
(Added @29/03/2023)

◯ Yes
◯ No

Was Nevirapine given to the baby/babies

◯ Yes
◯ No

Was birth PCR done for the baby/babies

◯ Yes
◯ No

NOTE: If not taken by birth facility please take blood for PCR.

PCR Barcode for the baby/baby 1

____

For peer review only

NOTE: If not taken by birth facility please take blood for PCR.

PCR Barcode for the baby/baby 2

____

NOTE: If not taken by birth facility please take blood for PCR.

PCR Barcode for the baby/baby 3

____

Result of birth PCR for baby 1

◯ Positive
◯ Negative
◯ Indeterminate
◯ Not yet available

Result of birth PCR for baby 2

◯ Positive
◯ Negative
◯ Indeterminate
◯ Not yet available

Result of birth PCR for baby 3

◯ Positive
◯ Negative
◯ Indeterminate
◯ Not yet available

Call clinician and make a note about this.

Was eye ointment given to the baby 1

◯ Yes
◯ No
◯ Don't know

Was eye ointment given to the baby 2

◯ Yes
◯ No
◯ Don't know

Was eye ointment given to the baby 3

◯ Yes
◯ No
◯ Don't know

Please specify to how many babies and which one

For peer review only

For peer review only - http://bmjopen.bmj.com/site/about/guidelines.xhtml

Was the baby/babies admitted to hospital following delivery

◯ Yes
◯ No

Please specify the details about the reason for admission, number of babies admitted and which babies

Does baby 1 have any of the following symptoms?

☐ Cough
☐ Runny nose
☐ Eye discharge
☐ Sneezing
☐ None

Does baby 2 have any of the following symptoms?

☐ Cough
☐ Runny nose
☐ Eye discharge
☐ Sneezing
☐ None

Does baby 3 have any of the following symptoms

☐ Cough
☐ Runny nose
☐ Eye discharge
☐ Sneezing
☐ None

Is the baby/babies receiving any treatment at the moment

◯ Yes
◯ No

Please specify

Feeding methods

◯ Breastfeeding
◯ Formula feeding
◯ Mixed

For peer review only

Delivery details of Mother

Is the baby present with the biological mother

◯ Yes
◯ No

Please specify

Are you currently taking any treatment

◯ Yes
◯ No

Please specify

Have you had sexual intercourse since delivery of your baby?

◯ Yes
◯ No

Do you have any of the following symptoms?

☐ Discharge
☐ Pain when urinating
☐ None

Please specify

You have completed all the questions associated with this visit. You will now start with the process of specimen collection. You will need to collect the following specimens:

From the mother you will need to collect 3 vaginal swabs:
- Vaginal Swab 1 for STI testing (Storage)
- Vaginal Swab 2 for Microbiome (Storage)
- Vaginal Swab 3 for Profiling (Storage)

From the baby you need to collect:
- Nasopharyngeal swab

- Conjunctival

Select "Proceed" to capture the information associated with these specimens

◯ Proceed

For peer review only

For peer review only - http://bmjopen.bmj.com/site/about/guidelines.xhtml

Please confirm the barcode for the vaginal swab collected for STI testing

◯ [baseline_arm_1][participant_pin]-PNV1

Please confirm the barcode for the vaginal swab collected for Microbiome

◯ [baseline_arm_1][participant_pin]-PNV3

Please confirm the Barcode for the Vaginal Swab collected for Profiling

◯ [baseline_arm_1][participant_pin]-PNV2

Please confirm the Barcode for the Nasopharyngeal swab (right nose) baby 1

◯ [baseline_arm_1][participant_pin]-PNB1N1

Please confirm the Barcode for the Nasopharyngeal swab (left nose) baby 1

◯ [baseline_arm_1][participant_pin]-PNB1N2

Please confirm the barcode for the Nasopharyngeal swab (right nose) baby 2

◯ [baseline_arm_1][participant_pin]-PNB2N1

Please confirm the barcode for the Nasopharyngeal swab (left nose) baby 2

◯ [baseline_arm_1][participant_pin]-PNB2N2

Please confirm the barcode for the Nasopharyngeal swab (right nose) baby 3

◯ [baseline_arm_1][participant_pin]-PNB3N1

Please confirm the barcode for the Nasopharyngeal swab (left nose) baby 3

◯ [baseline_arm_1][participant_pin]-PNB3N2

Please confirm the Barcode for the Nasopharyngeal swab for STI testing baby 1

◯ [baseline_arm_1][participant_pin]-PNB1N1

Please confirm the barcode for the Conjunctival swab (right eye) baby 1

◯ [baseline_arm_1][participant_pin]-PNB1C1

Please confirm the barcode for the Conjunctival swab (left eye) baby 1

◯ [baseline_arm_1][participant_pin]-PNB1C2

Please confirm the barcode for the Conjunctival swab (right eye) baby 2

◯ [baseline_arm_1][participant_pin]-PNB2C1

Please confirm the barcode for the Conjunctival swab (left eye) baby 2

◯ [baseline_arm_1][participant_pin]-PNB2C2

For peer review only

Please confirm the barcode for the Conjunctival swab (right eye) baby 3

◯ [baseline_arm_1][participant_pin]-PNB3C1

Please confirm the barcode for the Conjunctival swab (left eye) baby 3

◯ [baseline_arm_1][participant_pin]-PNB3C2

Please specify the vaginal pH

____

Please select which pH strips are used to measure vaginal pH

◯ CardinalHealth pH Indicator Strips (range 3.6-6.1)
◯ pH Indicator Strips pH 0-14
◯ Natureland vaginal pH test (range 3.5-6.5)

Did you give the participant the study voucher?

◯ Yes
◯ No

You have completed capturing the Post-Natal information. Please make sure to check that you have completed all the fields.

Please give the participant 6 weeks immunization visit date as per schedule.

Please select "Complete" then "Save and Exit".

Notes

Additional notes

For peer review only

Post-Natal specimens Results

Date

____

Staff name

____

You are about to capture the results of the specimens collected during the post natal visit

Please select "Proceed" to continue

◯ Proceed

Receive Date ________________________

Test date (Mother) ________________________

STI results from the mother

	Positive	Negative
CT	◯	◯
NG	◯	◯
TV	◯	◯

STI result, mother_ calc

____

Test date (Baby) ________________________

STI Results from Baby 1

	Positive	Negative
CT (Right Nose)	◯	◯
NG (Right Nose)	◯	◯
TV (Right Nose)	◯	◯
CT (Left Nose)	◯	◯
NG (Left Nose)	◯	◯
TV (Left Nose)	◯	◯

For peer review only

CT (Right Eye)	◯	◯
NG (Right Eye)	◯	◯
TV (Right Eye)	◯	◯
CT (Left Eye)	◯	◯
NG (Left Eye)	◯	◯
TV (Left Eye)	◯	◯

STI Results from Baby 2

	Positive	Negative
CT - Right Nose	◯	◯
NG - Right Nose	◯	◯
TV - Right Nose	◯	◯
CT - Left Nose	◯	◯
NG Left Nose	◯	◯
TV - Left Nose	◯	◯
CT - Right Eye	◯	◯
NG - Right Eye	◯	◯
TV - Right Eye	◯	◯
CT - Left Eye	◯	◯
NG - Left Eye	◯	◯
TV - Left Eye	◯	◯

STI Results from Baby 3

	Positive	Negative
CT - Right Nose	◯	◯
NG - Right Nose	◯	◯
TV - Right Nose	◯	◯
CT - Left Nose	◯	◯
NG - Left Nose	◯	◯
TV - Left Nose	◯	◯
CT - Right Eye	◯	◯
NG - Right Eye	◯	◯
TV - Right Eye	◯	◯
CT - Left Eye	◯	◯
NG - Left Eye	◯	◯
TV - Left Eye	◯	◯

Result of birth PCR for baby 1

◯ Positive
◯ Negative
◯ Indeterminate
◯ Not yet available

For peer review only

Result of birth PCR for baby 2

◯ Positive
◯ Negative
◯ Indeterminate
◯ Not yet available

Result of birth PCR for baby 3

◯ Positive
◯ Negative
◯ Indeterminate
◯ Not yet available

Notes

Notes

For peer review only

6-Week Immunization Visit Activities

Staff name ________________________

Today's date ________________________

Time ________________________

Did the mother present within the specified dates below:

Start Date: [predelivery_checki_arm_1][sixweek_remind_schedpd]

Actual Date: [predelivery_checki_arm_1][sixw_im_schedpd]

End Date: [predelivery_checki_arm_1][sixw_im_close_schedpd]

◯ Yes
◯ No

You are about to administer the questions associated with 6-weeks immunization visit.

Please select "Proceed"

◯ Proceed

How many babies were delivered?

◯ 1
◯ 2
◯ 3

Was baby 1 admitted to hospital since the last study visit

◯ Yes
◯ No

Was baby 2 admitted to hospital following delivery

◯ Yes
◯ No

Was baby 3 admitted to hospital following delivery

◯ Yes
◯ No

Please specify

For peer review only

Does baby 1 have any of the following symptoms?

- ☐ Cough
- ☐ Runny nose
- ☐ Eye discharge
- ☐ Sneezing
- ☐ None

Does baby 2 have any of the following symptoms?

- ☐ Cough
- ☐ Runny nose
- ☐ Eye discharge
- ☐ Sneezing
- ☐ None

Does baby 3 have any of the following symptoms?

- ☐ Cough
- ☐ Runny nose
- ☐ Eye discharge
- ☐ Sneezing
- ☐ None

Are any of the babies receiving any treatment at the moment

Feeding methods

- ☐ Breastfeeding
- ☐ Formula feeding
- ☐ Mixed

Have you or the baby been to the clinic since the last visit with us?

- ◯ Yes
- ◯ No

What was the purpose of your visit?

- ☐ ANC Visit
- ☐ HIV/ART
- ☐ STI Treatment
- ☐ Other

Summary notes from the visit

For peer review only

Do you know your current HIV status?

☐ HIV negative (tested today by clinical staff)
☐ HIV positive on ART
☐ Known HIV positive, not on ART
☐ Newly diagnosed HIV positive (tested today by clinical staff)
☐ Don't know (never tested)
☐ Don't know (no yet tested today)

Please conduct a HIV Rapid test and capture the result below

◯ Positive
◯ Negative

Please conduct a confirmatory HIV Rapid test and capture the result below

◯ Positive
◯ Negative

HIV PCR result of baby 1

◯ Positive
◯ Negative
◯ No result

Please record barcode for blood and HIV PCR

____

HIV PCR result of baby 2

◯ Positive
◯ Negative
◯ No result

Please record barcode for blood and HIV PCR

____

HIV PCR result of baby 3

◯ Positive
◯ Negative
◯ No result

Please record barcode for blood and HIV PCR

____

NOTE
You have collected all specimens associated with this visit. Once you select the "Proceed" option below you will be

CT: [post_natal_arm_1][sti_result_ct]

NG: [post_natal_arm_1][sti_result_ng]

TV: [post_natal_arm_1][sti_result_tv]

◯ Proceed

For peer review only - http://bmjopen.bmj.com/site/about/guidelines.xhtml

For peer review only

STI result, mother_ calc

____

Does the participant report any medication allergies?

◯ Yes
◯ No

Please contact the study clinician before giving any treatment. Please specify discussed medication allergies and treatment plan with the study clinician

The following treatment has been provided

☐ Azithromycin 1g stat dose
☐ Azithromycin 2g stat dose
☐ Ceftriaxone 250mg IM injection
☐ Ceftriaxone 1g IM injection
☐ Metronidazole 400mg bd x 1 week
☐ Metronidazole 2g stat dose
☐ Clotrimazole pessary and/or cream
☐ Trimethoprim/sulfamethoxazole 400/80 mg 2 tbl. bds for 5 days (bactrim)

Date treatment given

____

Partner notification provided

◯ Yes, 1
◯ Yes, multiple
◯ No

Please explain why the partner notification note was not provided?

The mother tested positive for an STI at the Post Natal visit. You need to collect a Nasal Pharyngeal swab for baby 1. Did you manage to collect this specimen?

◯ Yes
◯ No

Please confirm the PIN for the Nasal Pharyngeal swab for baby 1.

◯ [baseline_arm_1][participant_pin]-NPB1

For peer review only

The mother tested positive for an STI at the Post Natal visit. You need to collect a Nasal Pharyngeal swab for baby 2. Did you manage to collect this specimen?

◯ Yes
◯ No

Please confirm the PIN for the Nasal Pharyngeal swab for baby 2 .

◯ [baseline_arm_1][participant_pin]-NPB2

The mother tested positive for an STI at the Post Natal visit. You need to collect a Nasal Pharyngeal swab for baby 3. Did you manage to collect this specimen?

◯ Yes
◯ No

Please confirm the PIN for the Nasal Pharyngeal swab for baby 3.

◯ [baseline_arm_1][participant_pin]-NPB3

You have completed capturing the Post-Natal information. Please make sure to check that you have completed all the fields.

Please select "Unverified" then "Save and Exit".

Notes

Additional notes

For peer review only

Adverse Outcomes

Staff Name ______________________

Today's date ______________________

Start time ______________________

Was there an adverse birth outcome?

◯ Yes
◯ No

Was there a serious adverse event ◯ Yes ◯ No

Early loss of baby

What type of early loss?

◯ Miscarriage
◯ Ectopic
◯ Termination of pregnancy
◯ Still Born

Date

____

Ectopic pregnancy

Date of surgery

____

Termination pregnancy

Date

____

Reviewed by site PI ◯ Yes ◯ No

Date Reviewed ______________________

For peer review only

Review Notes	________________
Name of Reviewer	◯ Remco Peters

You have completed capturing the adverse outcomes information. Please make sure to check that you have completed all the fields.

Please select "Complete" then "Save and Exit".

Notes

Additional notes

For peer review only

Activities Associated with Visit

Staff Details

Staff Name ______________________

Today's Date ______________________

Start time ______________________

Presentation Outcome

Presentation outcome.

Did the participant present at the study site for this visit?

◯ Yes
◯ No

Activities Associated with ToC for Arm 1

You are about to facilitate activities associated with the 4-week ToC. You will need to execute the following:

1. Collect Specimens
2. Run a STI test
3. Conduct clinical history and behavioral questionnaire
4. Symptom screening if negative test
5. Treatment and partner referral if positive test

◯ Proceed

Activities Associated with 32 Week Visit

You are about to facilitate activities associated with the 32 week visit. You will need to execute the following:

1. Collect Specimens
2. Run a STI test
3. Conduct clinical history and behavioral questionnaire
4. Symptom screening if negative test
5. Treatment and partner referral if positive test

◯ Proceed

You are about to facilitate activities associated with the 32 week visit. You will need to execute the following:

1. Collect Specimens
2. Conduct clinical history and behavioral questionnaire
3. Symptom screening
4. Treatment and partner referral if positive screening

◯ Proceed

For peer review only - http://bmjopen.bmj.com/site/about/guidelines.xhtml

Activities Associated with the First Postnatal Visit

You are about to facilitate activities associated with the the 1st post natal visit. You will need to execute the following:

1. Determine the presentation date (Only proceed when its 14 days after the delivery date)
2. Collect pregnancy and birth outcomes data (Discharge Summary and/or Road to Health Card)
3. Conduct mother and child clinical examination and history questionnaire
4. Specimen collection for mother and child

◯ Proceed

Pregnancy and Birth Outcome Data

You are about to start with the pregnancy and birth outcome data capturing.

You can use the discharge summary and road to health as your data sources.

Select "Proceed" below to display the pregnancy and birth outcome details

◯ Proceed

Delivery date ________________________________

Mother and Baby Clinical Examination and History

You are done capturing the pregnancy and birth outcome data.

The next step is to capture the mother and baby clinical examination and history details.

Select "Proceed" below to display the questionnaire.

◯ Proceed

Scheduling the 6 week Immunization Date

Schedule a 6 week immunization. ________________________________

Use the below date assist to schedule the 6 week immunization date.

The below field must be equal to 42.

Use the date field above to ensure that the current field is equal to 42.

____

For peer review only

Collection of Vaginal Loops

You will need to collect a single vaginal loop that will be used to prepare two slides. Once collected you will need to prepare the slides for storage.

◯ Proceed

Date of collection of vaginal loops

____

....

Confirm the pin associated with the first vaginal loop that will be used for .....

◯ [baseline_arm_1][participant_pin]-FL1

Confirm the PIN associated with the second vaginal loop that will be used for .....

◯ [baseline_arm_1][participant_pin]-FL2

Storage of Loops

You have collected both slides. Before commencing with the rest of the specimens, please make sure to do the following:

1. Slides are individually packed in their own package
2. Record PIN on outside of package
3. Complete the lab CRF with matching PINs and test instructions

◯ Proceed

Vaginal Swab Collection

You will now collect 3 vaginal swabs. They will be used as follows:

1. STI testing (1st Specimen)
2. Profiling (2nd Specimen)
3. Microbiome (3rd Specimen)

◯ Proceed

....

Date of specimen collection for vaginal swabs

____

Confirm the PIN associated with the first vaginal swab

◯ [baseline_arm_1][participant_pin]-FV1

For peer review only - http://bmjopen.bmj.com/site/about/guidelines.xhtml

.....

Confirm the PIN associated with the second vaginal swab

◯ [baseline_arm_1][participant_pin]-FV2

Confirm the PIN associated with the third vaginal swab

◯ [baseline_arm_1][participant_pin]-FV3

Nasopharyngeal Swab Collection

You are about to collect the Nasopharyngeal swab on the Baby.

Collect the specimen and confirm the PIN below.

◯ [baseline_arm_1][participant_pin]-NS1

GeneXpert Testing for the First Specimen

You will now start with the testing of the first vaginal swab specimen.

Follow the below steps:
1. Ensure that the GeneXpert Machine is switched-on. Perform a quikc qulaity check on the machine.
2. Load the specimen and run the machine.
3. Conduct Clinical History and Behavioural Questionnaire

Select "Start Test" when ready to run the test.

◯ Start Test

Clinical History and Behavioural Questionnaire

You have started running the STI test.

Conduct clinical history and behavioural questionnaire. Select "Proceed" to display the questionnaire

◯ Proceed

How often have you had sex since the last time we saw you?

◯ 0
◯ 1 to 5 times a week
◯ More than 5 times a week

STI Results

	Positive	Negative
NG	◯	◯
TV	◯	◯
CT	◯	◯

For peer review only - http://bmjopen.bmj.com/site/about/guidelines.xhtml

The participant tested positive for an STI.

The next step is to administer treatment with the participant.

Select "Proceed" to display treatment options.

◯ Proceed

.....

The next step is to screen the patient for STI symptoms

Is the participant symptomatic?

◯ Yes
◯ No

The participant screened positive for at least a single STI symptom

The next step is to administer treatment with the participant.

Select "Proceed" to display treatment options.

◯ Proceed

Treament and Partner Notification

Select the treatment regimen you administered to the participant

◯ Azithromycin
◯ Doxycyclin
◯ Ceftriaxone
◯ Metronidazole

Did you administer partner notification treatment?

◯ Yes
◯ No

Storage Processes

You have collected all required specimens.

You can now prepare the specimens for storage, follow the below steps:
1. Ensure that each specimen has a complete Lab CRF
2. Pack the Lab CRFs in the specimen container
3. Ensure that the Lab CRF is complete and specimens are stored according to the storage requirements.

Select "Confirm" after perform tha above specimen procedures.

◯ Confirm

For peer review only

Notes

Additional notes

For peer review only

Close-out

Staff member ____________________

Date ____________________

Participant ID: [baseline_arm_1][participant_pin_verify]

TERMINATION DETAILS

Date of termination ____________________

Study Time-Point
- ◯ BASELINE
- ◯ TOC
- ◯ 32 WEEKS
- ◯ POST-NATAL VISIT

Reason for termination
- ◯ End of study (study completed)
- ◯ death (participant)
- ◯ Participant refused further participation
- ◯ Participant unable to adhere to visit schedule
- ◯ Participant relocated, no follow-up planned
- ◯ Investigator decision
- ◯ unable to contact the participant
- ◯ Participant not eligible for enrollment
- ◯ Invalid ID due to duplicate screening/enrollment
- ◯ Other
- ◯ Early study closure
- ◯ End of study (adverse outcome)

Specify refusal reason/ Investigator reason ____________________

Other, Specify ____________________

....

General Comments ____________________

For peer review only

Ad-Hoc

Staff name

____

Please capture date of visit

____

Please summarize the purpose of the visit

For peer review only

Safety Protocol

Today's date ______________________

Time ______________________

Staff ______________________

Safety Protocol Issue
- ◯ Social Harm
- ◯ Protocol Violation
- ◯ Unanticipated Problem

Date Reported ______________________

Notes ______________________

For peer review only

STI Data Qc And Filing

SECTION A: STAFF DETAILS

Staff Member Name ________________________________

Date ________________________________

SECTION B: QUALITY ASSURANCE

Forms Received from the Field

- ☐ BQ Consent form
- ☐ Study Note
- ☐ Proof of Reimbursement
- ☐ Enrolment Log
- ☐ Expert Baseline: CT/NG
- ☐ Expert Baseline: TV
- ☐ Expert Postnatal: CT/NG
- ☐ Expert Postnatal: TV
- ☐ NHLS: CD4 Count
- ☐ NHLS: Syphilis Test
- ☐ NHLS: Viral Load
- ☐ NHLS: Baby HIV PCR
- ☐ OTHER

(Select all that you received)

Other - Specify the other form(s) received ________________________________

QUALITY ASSURANCE: Phase 2A

Get all the participant's enrolment source documents and perform a comprehensive QC on all the source documents. After the QC is done, mark each document as "checked, properly completed" if you have no query opened on the source document.

IN CASES WHERE A QUERY IS OPENED. PLEASE CONTACT THE RESPONSIBLE DATA COLLECTOR IMMEDIATELY!

Please note that you also accept receipt of all source documents by checking them below.

	Checked, Properly Completed	Not completed, Returned to the RA
Consent Form	○	○
Study Note	○	○
Proof of Reimbursement	○	○
Enrolment Log	○	○
Expert Baseline: CT/NG	○	○

For peer review only - http://bmjopen.bmj.com/site/about/guidelines.xhtml

Expert Baseline: TV	○	○
Expert Postnatal: CT/NG	○	○
Expert Postnatal: TV	○	○
NHLS: CD4 Count	○	○
NHLS: Syphilis Test	○	○
NHLS: Viral Load	○	○
NHLS: Baby HIV PCR	○	○
[forms_received_oth]	○	○

Skip

Electronic Data QC

You are supposed to go through each electronic data tool and ensure the following:

1. Each Tracking Field has a data point.
2. The data is consistent
3. The data is verified with source documents

After doing the above inspection. You marked the forms as complete and locked the form.

Once all the forms are checked and properly completed.

	Checked and Completed Properly	Query Opened
1, Baseline: Screening and Enrolment	○	○
2, Baseline: Baseline data	○	○
3, STI: Physical Exam	○	○
4, STI: Specimen and Randomization	○	○
5, STI: Scheduling	○	○
6, STI: TOC Visit Activities	○	○
7, STI: Calling Reminder_2	○	○
8,STI: Scheduling post Delivery activities	○	○
9, Birth Register data	○	○
10, Post Natal Visit Activities	○	○
11, 6 week Immunization Visit Activities	○	○

view only

.....

Date of QC

____

COMMENTS

Comments

____

SAVING INSTRUCTION

MARK THIS FORM AS COMPLET ONCE VERIFIED AND LOCK IT.

SELECT SAVE AND EXIT FORM.

Proceed to QC other source documents.

For peer review only

Scheduling 2

Scheduling of Dates Associated with [randomization].

NOTE
You are about to schedule dates associated with [randomization] participants.

Please select "Proceed".

◯ Proceed

Scheduling of Dates Associated

NOTE:
You are about to schedule dates associated with microbiome participants.

Please select "proceed"

◯ Proceed

Scheduling Dates for 3-Week ToC

NOTE: The participant tested positive and therefore we need to schedule a date, exactly 3-weeks from today to conduct a test-of-cure.

Scheduling the 3-week ToC

NOTE:
Please schedule a date, 3 weeks from today treatment given. Please use the calculator assistance to ensure that you schedule a date exactly 21 days from today.

____

Calculator Assist

The number here must be equal to 21

____

ERROR
The field does not equal to 21, please change it

Have you handed the TOC date to the participant?

◯ Yes
◯ No

Scheduling Dates Associated with ToC Reminder

For peer review only

Schedule date for REMINDER of 3-week ToC visit

____

Calculator Assist for scheduling ToC reminder date

The reminder phone call will be made 18 days following the treatment date. The number of days need to equal to 18.

____

ERROR
You did not enter the date correctly. The number should equal to 18. Please redo the date.

Scheduling Dates Associated with 3-Week ToC Missed Visit Date

NOTE:
You have successfully scheduled the reminder date.

Please select "proceed" to schedule the missed visit date for the 3 week ToC visit.

◯ Proceed

Schedule the date for the MISSED VISIT of the ToC visit.

This date should be 3 weeks after the date on which the participant received their test result.

____

Calculator Assist for scheduling 3-week ToC Missed Visit

The participant's time period allowed for attending a ToC will start 3 weeks after they received their result and will close 3 weeks after the date they received their result.

The number here must show 35

____

ERROR
You did not enter the date correctly. The number should equal to 35. Please redo the date.

NOTE:
You have successfully scheduled the 3-week ToC close date

Please select "proceed" to start scheduling the next visit dates

◯ Proceed

For peer review only - http://bmjopen.bmj.com/site/about/guidelines.xhtml

Dates Associated with reminder for the 28 Week call

NOTE:
You are about to schedule dates for the call reminder at 28 weeks.

Please select "Proceed".

◯ Proceed

Note:
Schedule the date for the 28 week call. We will contact each participant to ask the date for their 30 weeks clinic visit is.

____

Calculation assist for scheduling the 32-week reminder date.

This number must equal to 196

____

Days to call reminder

____

ERROR
The number you have entered does not match 196. Please select a different date so that the number equals to 196.

Scheduling the Dates Asscoiated with the 32 Week Gestational Visit

NOTE
You are about the start scheduling dates associated with the 32 week visit. You will need to schedule the following associated dates:

1. Week 32 date
2. Week 32 reminder date
3. Week 32 missed visit date

Select "Proceed" to start scheduling

◯ Proceed

Schedule the date for the 32 week gestational age, visit

Note to RA: please make sure that this date does not fall on Friday, weekend, and public holidays.

____

Days Difference (the difference between 32 weeks & Gestational age)

____

For peer review only

Calculate assist for 32 week visit

The number here must equal to [gest_week_calc]

____

Match

____

The date you have entered does not meet the 93 day criteria. Does the intended or original date fall on a Friday weekend or public holiday?

◯ Yes
◯ No

ERROR
The numbers you have entered does not match. Please select a different date so that the numbers match.

Dates Associated with reminder for the 32 Week Gestational Age Visit

NOTE:
You have successfully scheduled the 32 week date.

We will need to contact the participant at least 7 days before the scheduled visit to remind them.

Select "Proceed" to schedule the reminder date for the 32 week visit.

◯ Proceed

Note:
Schedule the date for the 32 week reminder. We will contact each participant starting 7 days prior to their 32-week gestation date. That means the date scheduled here should be 7 days earlier then the scheduled date for the 32-week visit.

____

Calculation assist for scheduling the 32-week reminder date.

This number must equal to 7

____

ERROR
The number you have entered does not match 7. Please select a different date so that the number equals to 7

For peer review only

Dates associated with the 32 week gestational age missed visit

NOTE:
You have successfully scheduled the 32 week reminder date.

Select "Proceed" to schedule the 32 week missed visit date.

◯ Proceed

Schedule the date for the 32 week missed visit date.

Note: Participants will have 3 weeks (21 days) to present for their 32 week visit date after which the visit will be closed out.

____

Calculation Assist for scheduling the 32-Week missed visit date.

This number must equal to 21

____

ERROR
The number you have entered does not match 21. Please select a different date so that the number equals to 21

Estimated Delivery Date

You are about the schedule the Estimated Delivery Date.

Please select "proceed"

◯ Proceed

Estimated Delivery Date

____

Days difference between estimated date of delivery and gestational age

____

For peer review only

Calculation Assist for scheduling the Estimated Date for Delivery date.

This number must equal to [edod_calc]

____

Match

____

ERROR
The number you have entered does not match. Please select a different date so that the numbers match

You have completed all the scheduling dates.

Please check that all dates entered comply with the "calculation assistance". Once this has been done you can select "Complete" and "Save & Exit"

NOTES

Notes box

For peer review only

Data Quality

Name ______________________

Time ______________________

For peer review only

Updated Estimated Delivery Date

Please specify the updated delivery date

____

For peer review only
