# Supplementary material for: Genital tract infections, the vaginal microbiome and gestational age at birth among pregnant women in South Africa: a cohort study protocol
Source: BMJ Open. 2023 Dec 28;13(12):e081562. doi: 10.1136/bmjopen-2023-081562 (PMC10759125; doi:10.1136/bmjopen-2023-081562)
Supplement: Reviewer comments [file bmjopen-2023-081562.reviewer_comments.pdf]

PEER REVIEW HISTORY

BMJ Open publishes all reviews undertaken for accepted manuscripts. Reviewers are asked to complete a checklist review form (**http://bmjopen.bmj.com/site/about/resources/checklist.pdf**) and are provided with free text boxes to elaborate on their assessment. These free text comments are reproduced below.

ARTICLE DETAILS

**TITLE (PROVISIONAL)**	Genital tract infections, the vaginal microbiome and gestational age at birth among pregnant women in South Africa: a cohort study protocol
**AUTHORS**	Gigi, Ranjana; Mdingi, Mandisa; Jung, Hyunsul; Claassen-Weitz, Shantelle; Bütikofer, Lukas; Klausner, Jeffrey D.; Muzny, Christina; Taylor, Christopher; van de Wijgert, Janneke H.H.M.; Peters, Remco; Low, Nicola

This article was not externally reviewed at BMJ Open. Protocols that have been independently assessed prior to submission to BMJ Open are usually fast-tracked to publication on the grounds that further substantial changes will not be possible. This independent assessment will usually be external, independent review for both a substantial grant award from a non-commercial or government funder and ethics approval.
